# Radical cascade reaction of alkynes with *N*-fluoroarylsulfonimides and alcohols

**DOI:** 10.1038/ncomms8011

**Published:** 2015-04-22

**Authors:** Guangfan Zheng, Yan Li, Jingjie Han, Tao Xiong, Qian Zhang

**Affiliations:** 1 Department of Chemistry, Northeast Normal University, Changchun 130024, China

## Abstract

Cascade reactions initiated by radical addition to alkynes are synthetically very attractive because they enable access to highly complex molecular skeletons in only few synthetic steps under usually mild conditions. Here we report a general radical cascade reaction of alkynes, *N*-fluoroarylsulfonimides and alcohols, enabling the efficient synthesis of important α-amino-α-aryl ketones from readily available starting materials via a single operation. During this process, the highly regioselective nitrogen-centred radical addition to internal and terminal alkynes generating vinyl radicals and the next explicit migration of aryl group from the nitrogen source lead the following efficient desulfonylation, oxygenation, and semi-pinacol rearrangement. In addition, the semi-pinacol rearrangement precursors, α-alkyloxyl-α,α-diaryl imines, could also be efficiently obtained under milder conditions. This methodology might open a new entry for designing intermolecular radical cascade reaction of alkynes.

## Abstract (web summary)

Cascade and multi-component reactions allow highly functionalized molecules to be built from simpler precursors. Here, the authors report a three-component coupling giving α-amino-α-aryl ketones, initiated by the regioselective addition of nitrogen radicals to alkynes.

## Graphical abstract

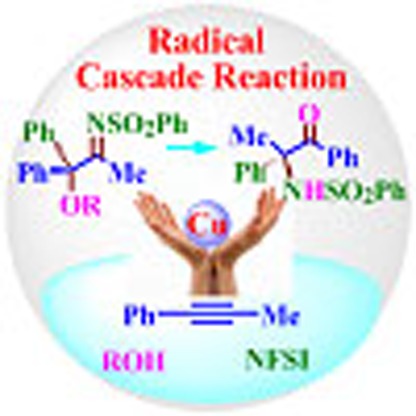

The regioselective construction of C–N bond under mild conditions remains an attractive research field due to the ubiquitous presence of amines in both naturally occurring and synthetic compounds, which manifest high levels of biological activity^1^,^2^. Alkyne functionalization, the addition of functional groups across a triple bond, exemplifies a class of reactions with significant synthetic potential. Accordingly, direct amination reaction of simple alkynes involving general intermolecular C–N bond construction step, such as hydroamiantion^3^,^4^,^5^,^6^, diamination^7^,^8^, aminooxygenation^9^,^10^,^11^,^12^, aminohalogenation^13^,^14^ and aminoacylation^15^ have been successfully developed, during which nucleophilic amination was usually involved with a few strategies employing electrophilic nitrogen sources (Fig. 1a). Cascade reactions initiated by radical addition to alkynes are synthetically very attractive because they allow access to highly complex molecular skeletons in only few synthetic steps under usually mild conditions, enabling them to exhibit high functional group compatiblity^16^. Although intermolecular radical addition to alkynes generating reactive vinyl radicals to perform intramolecular cascade reactions have been well established, their intermolecular multi-component equivalents remain a formidable challenge (*vide infra*). It is thus not surprising in that light that even simple addition reactions of nitrogen-centred radicals to alkynes are very rare^17^,^18^. In fact, compared with the well-established nucleophilic and electrophilic amination reaction, the construction of C–N bonds based on nitrogen-centred radicals have not received sufficient attention. The highly reactive vinyl radical generated by the addition of nitrogen-centred radical to alkynes offers a unique platform for radical-based processes mechanistically distinct from ionic pathways. We anticipate that this novel bond-forming strategy could be harnessed for facile construction of otherwise challenging nitrogen containing molecular architectures with traditional methodologies.

Challenges for the development of general cascade reactions initiated by nitrogen-centred radical addition to alkynes mainly resulted from two reasons: (1) the usually harsh conditions for the generation of nitrogen-centred radicals and their leading propensity for hydrogen abstraction or engaging in other degradation pathway; (2) the lack of a general intramolecular trapping manner to transfer the highly reactive incipient vinyl radical for further intermolecular cascade design. Recently, we developed copper-catalysed aminocyanation, diamination and aminoflurination reaction of alkenes via the efficient generation of nitrogen-centred radical from *N*-fluorobenzenesulfonimide (NFSI) under mild conditions^19^,^20^. As part of our continuing interest in employing NFSI as efficient amination nitrogen source^21^,^22^,^23^,^24^, in this article, a novel aminative multifunctionalization cascade reaction of alkyne with *N*-fluoroarylsulfonimide (as both nitrogen and aryl source) and alcohol (as oxygen source) was developed. Utilizing this simple transformation, α-amino-α-aryl ketones could be efficiently synthesized from both terminal and internal alkynes (Fig. 1b). α-Amino-α-aryl ketones, such as *N*-methylwelwitindolinone C isothiocyanate^25^, ketamine^26^ and prasugrel^27^ belong to an important class of biologically active natural products and pharmaceuticals. They are also useful precursors for the synthesis of heterocycles^28^,^29^,^30^,^31^ and 1,2-amino alcohols^32^,^33^. Recently, starting from readily available substrates, interesting methods for α-amino-α-aryl ketones such as cross-aza benzoin reaction of aldehydes with aryl imines^34^,^35^,^36^,^37^,^38^ and acyloin-type cross-coupling of aryl imines with nitriles^39^ were developed. Although significant progress has been made in the formation of C–N^40^,^41^,^42^,^43^ and C–C(aryl)^44^,^45^,^46^,^47^,^48^ bonds at α-position of the carbonyl group, it is a great challenge to simultaneously form C–N and C–C(aryl) bonds especially for the construction of quaternary carbon centres.

Herein, we report a cascade reaction that offers highly efficient construction of α-amino-α-aryl ketones starting from readily available alkynes, *N*-fluoroarylsulfonimides and alcohols via a highly efficient sequential regioselective nitrogen-centred radical addition to alkyne/aryl migration/desulfonylation/oxygenation/semi-pinacol rearrangement process (Fig. 1b). In addition, the semi-pinacol rearrangement precursors, α-alkyloxyl-α,α-diaryl imines, could also be efficiently obtained under milder conditions.

## Results

### Optimization for the sythesis of α-amino-α-aryl ketones

On the basis of previous reports developed by us^19^,^20^ and others^49^,^50^,^51^, we sought to use NFSI as both nitrogen source and aryl source to investigate aminative multifunctionalization of alkynes. Our investigation commenced with the reaction of phenylacetylene (**1a**, 0.5 mmol) with NFSI (0.75 mmol, 1.5 equiv.) in the presence of Cu(OTf)_2_ (10 mol %) at 90 °C in commercially available CH_3_CN (2 ml) under N_2_ atmosphere, α-amino-α-aryl ketone **2a** was obtained in 30% yield after 8 h. However, when dry CH_3_CN was used, no reaction occurred. Therefore, water (1.5 mmol, 3 equiv.) was added to the reaction system, α-amino-α-aryl ketone **2a** was obtained in 37% yield. In this reaction, C–N, C–C(aryl) and C=O bonds were simultaneously introduced to alkyne **1a**. Delightfully, the readily available CH_3_OH was viable and furnished **2a** in 58% yield (Table 1, entry 1). With pyridine-*N*-oxide or CH_3_COOH as oxygen source, no **2a** was obtained. So the reaction of **1a** with NFSI and CH_3_OH was used as the model to optimize the reaction conditions. As shown in Table 1, other catalysts, such as CuCl, Fe(OTf)_2_, Zn(OTf)_2_ and Sc(OTf)_3_ could catalyse the reaction, but no improved result was obtained (Table 1, entry 2–5). A decrease in the temperature from 90 to 70 °C afforded **2a** in 54% yield (Table 1, entry 6). Further lowering the temperature to 50 °C resulted in sluggish reaction and only a trace amount of **2a** was observed (Table 1, entry 7). Screening of solvents (Table 1, entries 8–10) identified CH_3_CN as the solvent of choice. Finally, a satisfactory yield of 78% was achieved when CF_3_COOH was employed as additive (Table 1, entries 11–14). Considering the number of steps involved in this process, this overall yield indicates of high efficiency of this radical involved cascade. Interestingly, the reaction could also proceed at 130 °C without catalyst to provide **2a** in 32% yield (Table 1, entry 16).

### Scope of terminal alkyne and *N*-fluoroarylsulfonimide substrates

With the optimized conditions at hand (Table 1, entry 14), the scope of aminative multifunctionalization of terminal alkynes was investigated. The tested phenylethetylene derivatives **1** smoothly reacted with NFSI and CH_3_OH to afford the corresponding α-amino-α-aryl ketones **2** or **3** in 48–78% yields (Table 2). For α-amino-α-aryl ketones **2**, phenyl group from NFSI connected to the terminal carbon of alkynes and for α-amino-α-aryl ketones **3**, aryl group from alkynes **1** connected to the terminal carbon of alkynes. For alkynes **1b**–**1f** which bear *ortho*-substitutions, **2b**–**2f** became the major products with the less sterically hindered phenyl group selectively migrated, forming C−C (aryl) bonds. Alkynes **1g**–**1o** with electron donating or withdrawing groups afforded major products **2g**–**2i** and **3j**–**3o** in which the comparatively electron-rich aromatic ring migrated to form C−C (aryl) bonds. In addition, NFR1 (*N*-fluoro-4-methyl-*N*-tosylbenzenesulfonamide) was used instead of NFSI to further explore the scope of this alkyne aminative multifunctionalization. As expected, the reaction of **1** with NFR1 and CH_3_OH proceeded smoothly and provided **4a**–**h** and **5a**–**h** (with **4a**–**h** as major products) in 48–84% yield (Table 3). Similar electronic and steric effects as using NFSI were observed. Trifluoromethyl and cyano groups were compatible and provided the corresponding α-amino-α-aryl ketones **4g** (81%) and **4h** (48%). However, substrates with strong electron-donating groups on the aromatic ring, such as 1-ethynyl-4-methoxybenzene and 1-ethynyl-3-methoxybenzene, were not effective. In addition, reactions between 1-(*tert*-butyl)-4-ethynylbenzene and some other NFSI derivatives were also explored to extend scope and investigate electronic effect of aryl part of NFSI derivatives. For 4-chloro-*N*-((4-chlorophenyl)sulfonyl)-*N*-fluorobenzenesulfonamide (NFR2), **5i** was obtained as a single isomer in yield of 46%. For *N*-fluoroarylsulfonimides 4-*tert*-butyl-*N*-fluoro-*N*-(phenylsulfonyl)benzenesulfonamide (NFR3) and 4-*tert*-butyl-*N*-(4-chlorophenylsulfonyl)-*N*-fluorobenzenesulfonamide (NFR4), the corresponding α-amino-α-aryl ketones were obtained in 72 and 67% yield, respectively. These results showed that the transformation was more efficient for electron-rich aromatic rings than electron-poor aromatic rings in NFSI derivatives. The halogen atom on the aromatic ring was tolerated in this process (**2e**–**i**, **4c**–**f, 5i, 5k**), offering an opportunity for further elaboration.

### Scope of internal alkyne substrates

To examine the generality of this alkyne aminative multifunctionalization, internal alkynes were subsequently examined. In the presence of 5 mol% CuCN, the reaction of pent-1-yn-1-ylbenzene (**6a**, 0.5 mmol), NFSI (1.5 equiv., 0.75 mmol) and *i*-PrOH (1.5 equiv., 0.75 mmol) in dichloromethane (DCM, 2 ml) was carried out at 70 °C under nitrogen atmosphere for 12 h. The expected α-amino-α-aryl ketone **8a** with a quaternary carbon at α-position was afforded in 68% yield (Table 4). As shown in Table 4, an array of α-amino-α-aryl ketones **8** and/or **9** were obtained in yields ranging from 48 to 73%. Similarly, preferential migration of electron-rich aromatic substituent (aryl on the alkyne versus Ph from NFSI) was observed in the product distribution. It should be noted that this aminative multifunctionalization of internal alkynes directly lead to the skeleton of α-tertiary amine derivatives, which are widespread in various natural products and bioactive compounds^52^,^53^,^54^,^55^. Quaternary carbon centres with a nitrogen substituent have been successfully constructed through molecular rearrangement^56^. However, special structures of substrates were necessary. Therefore, the directly aminative multifunctionalization of alkynes could provide a new and facile way for α-tertiary amines. Recently, Murakami and co-workers^57^ reported an interesting Cu- and Rh-catalyzed aminative multifunctionalization of terminal alkynes to form α-amino-α-allyl ketones via α-imino metal carbene intermediate, during which C–C(allyl) bond formed through Claisen-type rearrangement. In their study, for internal alkynes, *N*-sulfonyl-1,2,3-triazoles needed to be pre-prepared.

### Mechanism investigation

Radical scavengers were employed to probe the mechanism of the aminative multifunctionalization of alkynes. Formation of **2a** was completely inhibited when 1 equivalent of 2,6-di-*tert*-butyl-4-methylphenol or 2,2,6,6-tetramethyl-1-piperidinyloxy was added to the reaction. For the reaction with 2,6-di-*tert*-butyl-4-methylphenol as radical scavenger, 26% benzylic C–H amination product was obtained. These results suggested a possible radical mechanism. In combination with our previous finding in amination^19^,^20^,^21^,^22^,^23^,^24^, we proposed a possible mechanism as depicted in Fig. 2. Initially, the *in situ*-generated nitrogen-centred radical **A** added to the triple bond of alkyne (for example **6g**) regioselectively, providing a highly reactive vinyl radical **B**. Subsequently, sequential intramolecular 1,4-aryl migration *via* 5-*ipso* cyclization and desulfonylation would produce amidyl radical **C**^58^,^59^,^60^,^61^. This imidyl radical exists at an equilibrium with its resonance structure α-imino carbon radical **D** which could be stabilized by two aromatic rings and a C=N double bond. Then, the single-electron oxidation of intermediate **D** by NFSI generated a carbocation intermediate **E** and a nitrogen-centred radical **A** to continue the next catalytic cycle. The reaction between intermediate **E** and ROH provided α-alkyloxyl imine **F**. Finally, the protonation and semi-pinacol rearrangement of intermediate **F** furnished aminative multifunctionalization to provide isomer **8g** and **9g**. The ratio of **8g** to **9g** depended on electronic and steric effects of the corresponding aromatic substituents. As depicted in Tables 2, 3, 4, electron-rich and the sterically less-hindered aromatic rings are more prone to migrate, which is in consistency with the requirements of semi-pinacol rearrangement. It is noted that during this transformation, trapping of the incipient vinyl radical by aromatic ring from nitrogen source was a key step leading to intermolecular cascade process, which might provide a new entry to design radical addition initiated multi-component cascade reaction of alkynes.

Since the above-mentioned mechanism invoked a semi-pinacol rearrangement from a relatively stable species α-alkyloxyl-α,α-diaryl imine **F** to the final product, we questioned if this species could be obtained separately with modification of reaction parameters. Recently, semi-pinacol rearrangement of α-hydroxy imines had been successfully applied in natural product as well as catalytic asymmetric syntheses^62^,^63^,^64^. To our delight, the reaction of pent-1-yn-1-ylbenzene (**6a**, 0.5 mmol), NFSI (1.0 mmol, 2.0 equiv) and propan-2-ol (1.5 mmol, 3.0 equiv.) in the presence of Cu(acac)_2_ (5 mol %) at 0 °C in dry CH_3_CN (2 mL) under N_2_ atmosphere was performed for 48 h, α-alkyloxyl-α,α-diaryl imine **7a** was obtained in 71% yield. Besides the reaction temperature, catalyst played an important role in obtaining this product because no desired **7a** was obtained without copper. As shown in Table 5, various alcohols could react with NFSI and alkynes to obtain the corresponding α-alkyloxyl-α,α-diaryl imine **7a**–**7j** in 46–71% yields. It should be noted that diaryl-substituted alkynes are also effective. Starting from 1,2-diphenylethyne (**6k**), the corresponding α-alkyloxyl-α,α-diaryl imine was obtained in 66% under relatively higher temperature (Table 5, entry 11). Interestingly, for substrate **6l**, nitrogen-centred radical highly regioselectively added to the alkyne carbon connected to the aromatic ring with strong electron-withdrawing NO_2_ group. From substrates **6m** and **6n**, regioisomer mixtures (**7m**:**7m′**=1.5:1, **7n**:**7n′**=1:2) were obtained.

**Figure 1:**
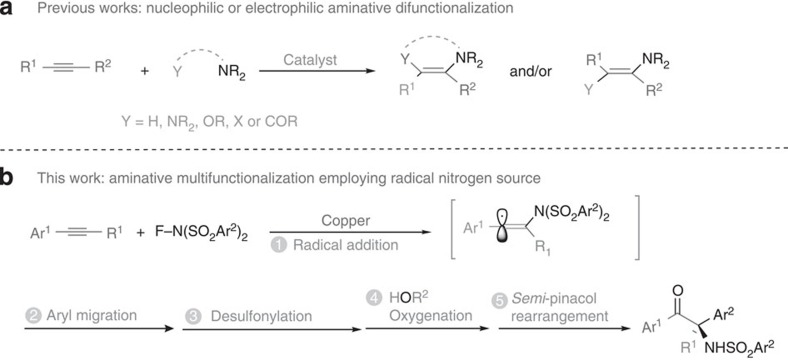
Aminative functionalization of alkynes. (**a**) Nuclephilic or electrophilic aminative difunctionalization. (**b**) Radical cascade aminative multifunctionalization.

**Figure 2:**
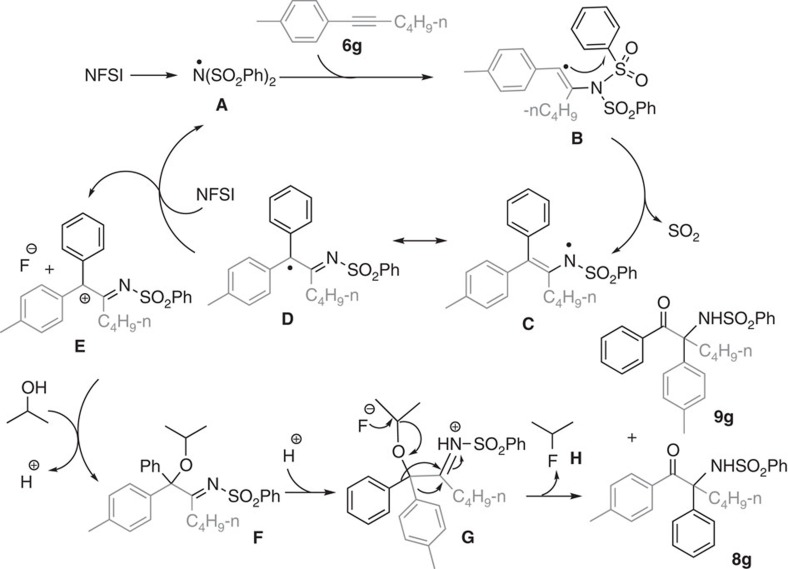
Proposed mechanism. Sequential regioselective nitrogen-centred radical addition to alkyne/aryl migration/desulfonylation/oxygenation/semi-pinacol rearrangement were involved.

Identification of intermediate **F** (Fig. 1) provided strong proof to the proposed mechanism. Therefore, further experiments for more mechanistic information were also carried out. The final α-amino-α-aryl ketone **8a** could be obtained in 87% yield when heating **7a** (0.3 mmol) at 70 °C in the presence of 0.3 mmol trifluoroacetic acid (TFA) in 2 ml DCM for 4 h, lending further support for **7** as the key intermediate in the novel aminative multifunctionalization of alkynes. However, under the same conditions but adding CuCN (5 mol %) instead of TFA, no reaction occured. Instead, when ZnCl_2_ (10 mol%) was added, **8a** was isolated in 60% yield, which showed that ZnCl_2_ additive in Table 4 played an important role for the transformation from intermediate **F** (Fig. 2) to final α-amino-α-aryl ketones. When the semi-pinacol rearrangement of **7a** was performed in the presence of HF acid (1 equiv., 40 wt % in water) instead of TFA, **8a** could be obtained in 68% yield, along with identified side-product 2-fluoropropane (**H**, Fig. 2). Starting from **7l**, the next semi-pinacol rearrangement process was not effective, which elucidated why transformation from diaryl-substituted alkynes to the desired α-amino-α-aryl ketones could not be realized in this study. Starting from **6a**, when *t*-BuOH was employed instead of *i*-PrOH under otherwise same conditions described in Table 5, entry 9, a α-fluoro-α,α-diaryl imine could be obtained in 36% yield, which demonstrated the possible presence of intermediate **E** (Fig. 2).

In conclusion, an unprecedented cascade radical aminative multifunctionalization reaction of various aryl terminal and internal alkynes with *N*-fluoroarylsulfonimides and simple alcohols is developed. This methodology provides a new facile and straightforward way for both α-amino-α-aryl ketones and α-alkyloxyl-α,α-diaryl imines, especially for the construction of quaternary α-amino ketones, which might open a new entry for designing multi-component radical cascade reactions of alkynes. Further studies for the application of this transformation are ongoing in our laboratory.

## Methods

### General methods

For ^1^H, ^19^F and ^13^C NMR spectra of compounds in this manuscript, see Supplementary Figs 1–121. For details of the synthetic procedures, tables including detail experimental, see Supplementary Information.

### Preparation of **2a**

To a solution of the NFSI (0.75 mmol, 236.5 mg) in CH_3_CN (2.0 ml) was added the CH_3_OH (1.5 mmol, 61 μl), TFA (0.5 mmol, 37 μl), 1-Phenylethyne (**1a**, 0.5 mmol, 54 μl) and Cu(OTf)_2_ (0.05 mmol, 18.1 mg) in screw-cap test tube under N_2_ atmosphere. The test tube was then sealed off with a screw-cap and the reaction mixture was stirred at 70 °C for 5.0 h. After the reaction finished, the reaction mixture was cooled to room temperature and quenched by water. The mixture was extracted with CH_2_Cl_2_ (3 × 5.0 ml), the combined organic phases were dried over anhydrous Na_2_SO_4_ and the solvent was evaporated under vacuum. The residue was purified by column chromatography (petroleum ether/ethyl acetate (10:1 v/v)) to give the corresponding product **2a** (136.9 mg, 78%).

### Preparation of **8a**

To a solution of the NFSI (0.75 mmol, 236.5 mg) in CH_2_Cl_2_ (2.0 ml) was added the isopropanol (0.75 mmol, 57 μl), but-1-yn-1-ylbenzene (**6a**, 0.5 mmol, 80 μl), ZnCl_2_ (0.01 mmol, 1.4 mg) and CuCN (0.025 mmol, 2.2 mg) in screw-cap test tube under N_2_ atmosphere. The test tube was then sealed off with a screw-cap and the reaction was stirred at 70 °C for 12.0 h. After the reaction finished, the reaction mixture was cooled to room temperature and quenched by water. The mixture was extracted with CH_2_Cl_2_ (3 × 5.0 ml), the combined organic phases were dried over anhydrous Na_2_SO_4_ and the solvent was evaporated under vacuum. The residue was purified by column chromatography (petroleum ether/ethyl acetate 10:1 (v/v)) to give the corresponding product **8a** (133.7 mg, 68%).

### Preparation of **7a**

To a solution of the NFSI (1.0 mmol, 314.3 mg) in CH_3_CN (2.0 ml) was added the isopropanol (1.5 mmol, 114 μl), but-1-yn-1-ylbenzene (**6a**, 0.5 mmol, 80 μl) and Cu(acac)_2_ (0.025 mmol, 6.5 mg) in screw-cap test tube under N_2_ atmosphere. The test tube was then sealed off with a screw-cap and the reaction was stirred at 0 °C for 48.0 h. After the reaction finished, the reaction mixture was quenched by water. The mixture was extracted with CH_2_Cl_2_ (3 × 5.0 ml), the combined organic phases were dried over anhydrous Na_2_SO_4_ and the solvent was evaporated under vacuum. The residue was purified by column chromatography (petroleum ether/diethyl ether (25:1 v/v)) to give the corresponding product **7a** (154.5 mg, 71%).

### Preparation of **7k**

To a solution of NFSI (1.0 mmol, 315.3 mg) in CH_2_Cl_2_ (2.0 ml) was added methanol (1.5 mmol, 61 μl), 1,2-diphenylethyne (**6k**, 0.5 mmol, 89 mg) and CuCN (0.025 mmol, 2.2 mg) in screw-cap test tube under N_2_ atmosphere. The test tube was then sealed off with a screw-cap and the reaction was stirred for the 48.0 h at 90 °C. After the reaction finished, the reaction mixture was cooled to room temperature and quenched by water. The mixture was extracted with CH_2_Cl_2_ (3 × 5.0 ml), the combined organic phases were dried over anhydrous Na_2_SO_4_ and the solvent was evaporated under vacuum. The residue was purified by column chromatography (petroleum ether/ethyl acetate 20:1 (v/v)) to give the corresponding product **7k** (145.6 mg, 68%).

## Author contributions

G.Z., J.H. and T.X. performed the experiments and analysed the data. Y.L. and Q.Z. designed and directed the project and wrote the manuscript. G.Z. and Y.L. contributed equally to this work. All the authors discussed the results and commented on the manuscript.

## Additional information

**Accession codes**: The X-ray crystallographic coordinates for structures reported in this article have been deposited at the Cambridge Crystallographic Data Centre (CCDC), under deposition number 979497 (**2b**), 979496 (**9h**) and 1031824 (**7h**). These data can be obtained free of charge from The cambridge Crystallographic Data Centre via www.ccdc.cam.ac.uk/data_request/cif.

**How to cite this article:** Zheng, G. *et al*. Radical cascade reaction of alkynes with *N*-fluoroarylsulfonimides and alcohols. *Nat. Commun.* 6:7011 doi: 10.1038/ncomms8011 (2015).

## Supplementary Material

Supplementary Information Supplementary Figures 1-126, Supplementary Tables 1-2, Supplementary Methods and Supplementary References

## Figures and Tables

**Table 1:** Optimization of the reaction conditions.

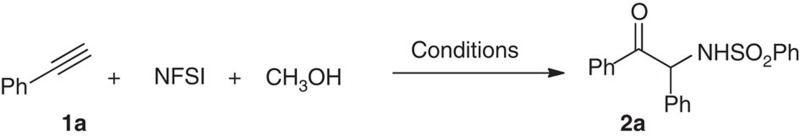						
**Entry**	**Catalyst**	**Additive**	**Solvent**	**Temperature (**^o^**C)**	**Time (h)**	**Yield (%)**
1	Cu(OTf)_2_	None	CH_3_CN	90	5	58
2	CuCl	None	CH_3_CN	90	5	40
3	Fe(OTf)_2_	None	CH_3_CN	90	7	50
4	Zn(OTf)_2_	None	CH_3_CN	90	20	47
5	Sc(OTf)_3_	None	CH_3_CN	90	20	44
6	Cu(OTf)_2_	None	CH_3_CN	70	5	54
7	Cu(OTf)_2_	None	CH_3_CN	50	24	Trace
8	Cu(OTf)_2_	None	DCM	70	24	34
9	Cu(OTf)_2_	None	EtOAc	70	24	NR*
10	Cu(OTf)_2_	None	THF	70	24	—†
11	Cu(OTf)_2_	C_6_H_5_COOH	CH_3_CN	70	4	60
12	Cu(OTf)_2_	CH_3_COOH	CH_3_CN	70	3	61
13	Cu(OTf)_2_	CF_3_SO_3_H	CH_3_CN	70	4	40
14	Cu(OTf)_2_	CF_3_COOH	CH_3_CN	70	5	78
15	None	None	CH_3_CN	70	24	NR*
16	None	None	CH_3_CN	130	10	32

Reaction conditions: **1a** (0.5 mmol), NFSI (1.5 equiv., 0.75 mmol), CH_3_OH (3 equiv., 1.5 mmol), catalysts (10 mol %), additives (1 equiv., 0.5 mmol), anhydrous solvents (2 ml), N_2_ atmosphere. Isolated yield.

^*^NR, no reaction.

^†^HN(SO_2_Ph)_2_ was identified.

**Table 2:** Aminative multifunctionalization of terminal alkynes with NFSI.

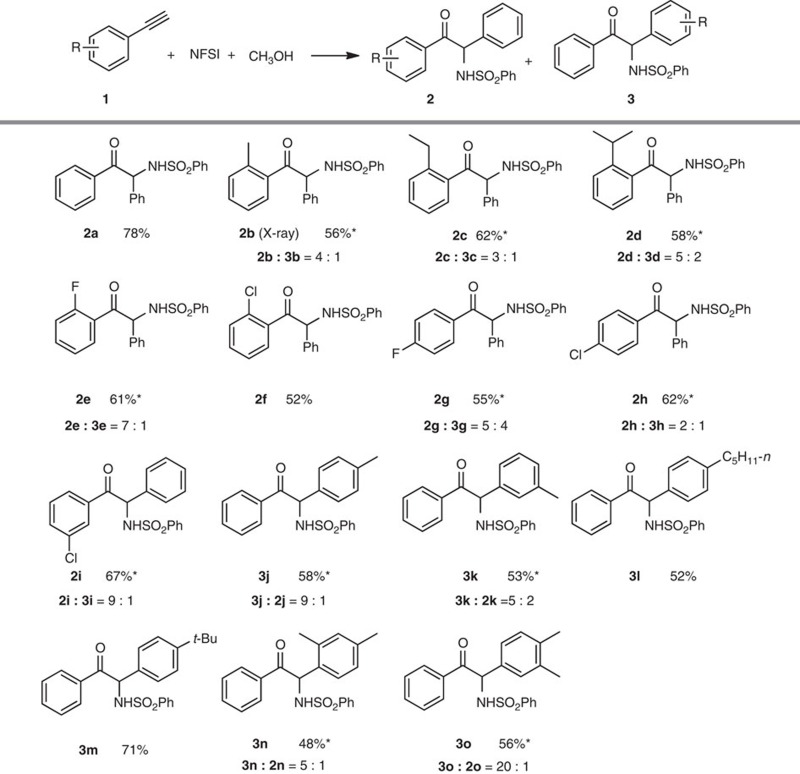

NFSI, *N*-fluorobenzenesulfonimide.

Reaction condition: **1** (0.5 mmol), NFSI (1.5 equiv., 0.75 mmol), CH_3_OH (3 equiv., 1.5 mmol), Cu(OTf)_2_ (10 mol %) and TFA (1.0 equiv., 0.5 mmol) in CH_3_CN (2 ml) at 70 °C under N_2_ atmosphere for 5 h. Isolated yield.

*Mixture of two isomers. The ratio was determined by ^1^H NMR analysis.

**Table 3:** Aminative multifunctionalization of terminal alkynes with NFR1–4.

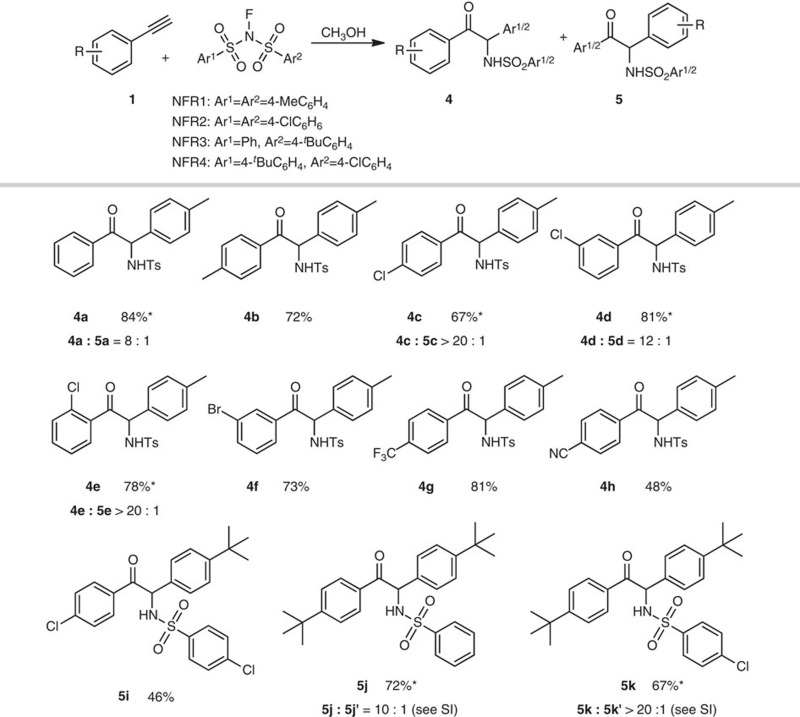

Reactions condition: **1** (0.2 mmol), NFR1–4 (1.5 equiv., 0.3 mmol), CH_3_OH (3 equiv., 0.6 mmol), Cu(OTf)_2_ (10 mol %) and TFA (1.0 equiv., 0.2 mmol) in CH_3_CN (2 ml) at 90 °C under N_2_ atmosphere for 8 h. Isolated yields.

*Mixture of two isomers. The ratio was determined by ^1^H NMR analysis.

**Table 4:** Aminative multifunctionalization of internal alkynes with NFSI.

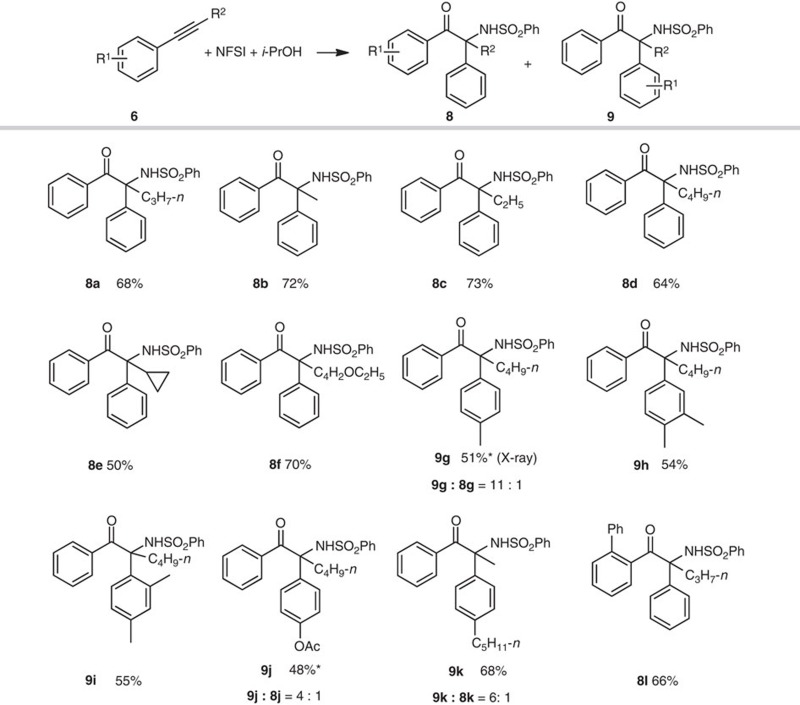

NFSI, *N*-fluorobenzenesulfonimide.

Reactions condition: **6** (0.5 mmol), NFSI (1.5 equiv., 0.75 mmol), *i*-PrOH (1.5 equiv., 0.75 mmol), CuCN (5 mol %) and ZnCl_2_ (2 mol %) in CH_2_Cl_2_ (2 ml) at 70 °C under N_2_ atmosphere for 12 h. Isolated yields.

*Mixture of two isomers. The ratio was determined by ^1^H NMR analysis.

**Table 5:** Syntheses of α-alkyloxyl-α,α-diaryl imine.

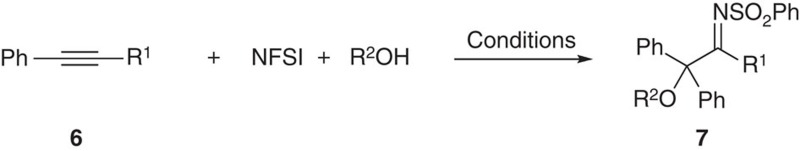					
**Entry**	**R**^**1**^	**R**^**2**^**OH**	**Temperature (**^**o**^**C)**	**Product**	**Yield (%)**
1	*n*-C_3_H_7_	*i*-PrOH	0	**7a**	71
2	Me	*i*-PrOH	10	**7b**	68
3	*n*-C_4_H_9_	*i*-PrOH	0	**7c**	63
4	*n*-C_3_H_7_	CH_3_OH	10	**7d**	52
5	*n*-C_3_H_7_	EtOH	10	**7e**	62
6	*n*-C_3_H_7_	Butan-1-ol	10	**7f**	58
7	*n*-C_3_H_7_	Butan-2-ol	0	**7g**	65
8	*n*-C_3_H_7_	Cyclohexanol	10	**7h**	70
9	*n*-C_3_H_7_	Prop-2-yn-1-ol	25	**7i**	54
10	*n*-C_3_H_7_	(*E*)-but-2-en-1-ol	25	**7j**	46
11*	Ph	CH_3_OH	90	**7k**	66
12*	4-NO_2_C_6_H_4_	CH_3_OH	90	**7l**	45
13*	4-acetyl C_6_H_4_	CH_3_OH	90	**7m:7m′=1.5:1**	41†
14*	4-^*t*^BuC_6_H_4_	CH_3_OH	90	**7n:7n′=1:2**	54†

Reactions condition: **6** (0.5 mmol), NFSI (2 equiv., 1.0 mmol), R^2^OH (3 equiv., 1.5 mmol), Cu(acac)_2_ (5 mol %) in CH_3_CN (2 ml) under N_2_ atmosphere for 48 h. Isolated yields.

^*^Reactions condition: **6** (0.5 mmol), NFSI (2 equiv., 1.0 mmol), CH_3_OH (3 equiv., 1.5 mmol), CuCN (5 mol %) in CH_2_Cl_2_ (2 ml) at 90 °C under N_2_ atmosphere for 48 h. Isolated yields.

^†^Mixture of two isomers. The ratio was determined by ^1^H NMR analysis.
