# Supplementary material for: Radical cascade reaction of alkynes with N-fluoroarylsulfonimides and alcohols
Source: Nat Commun. 2015 Apr 22;6:7011. doi: 10.1038/ncomms8011 (PMC4421815; doi:10.1038/ncomms8011)
Supplement: Supplementary Information — Supplementary Figures 1-126, Supplementary Tables 1-2, Supplementary Methods and Supplementary References [file ncomms8011-s1.pdf]

Supplementary Figures

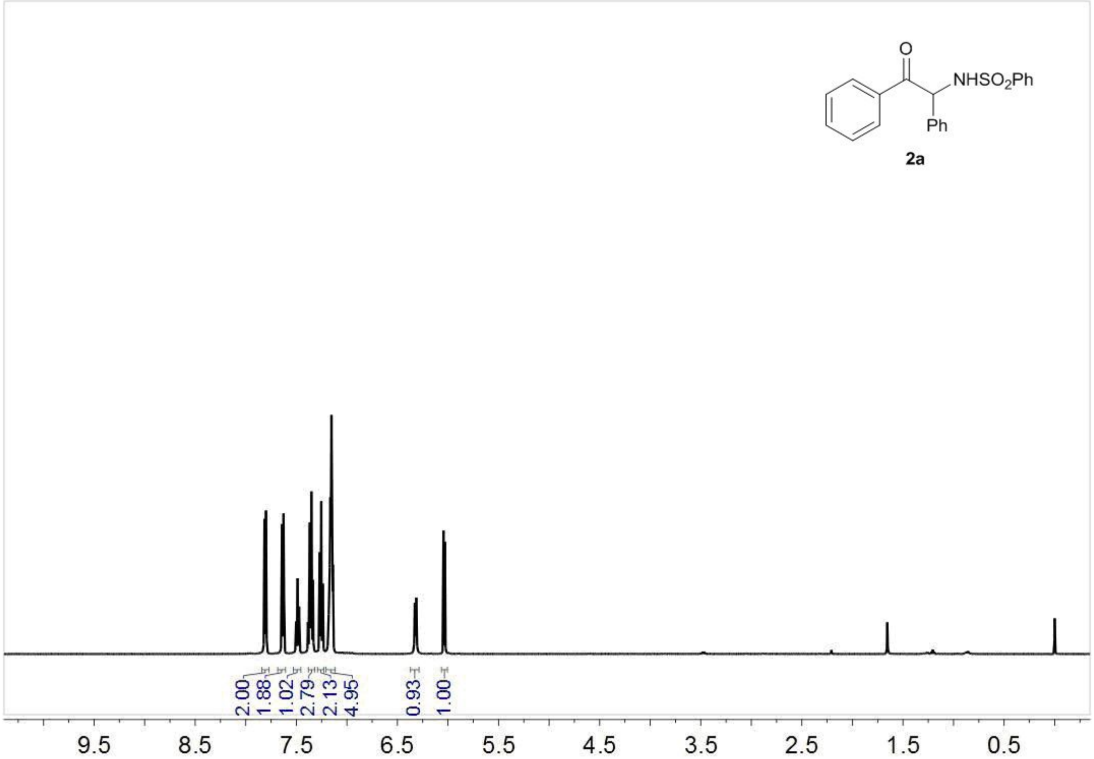

Supplementary Figure 1. ^{1}H NMR (500 MHz, CDCl$_3$) spectrum for 2a.

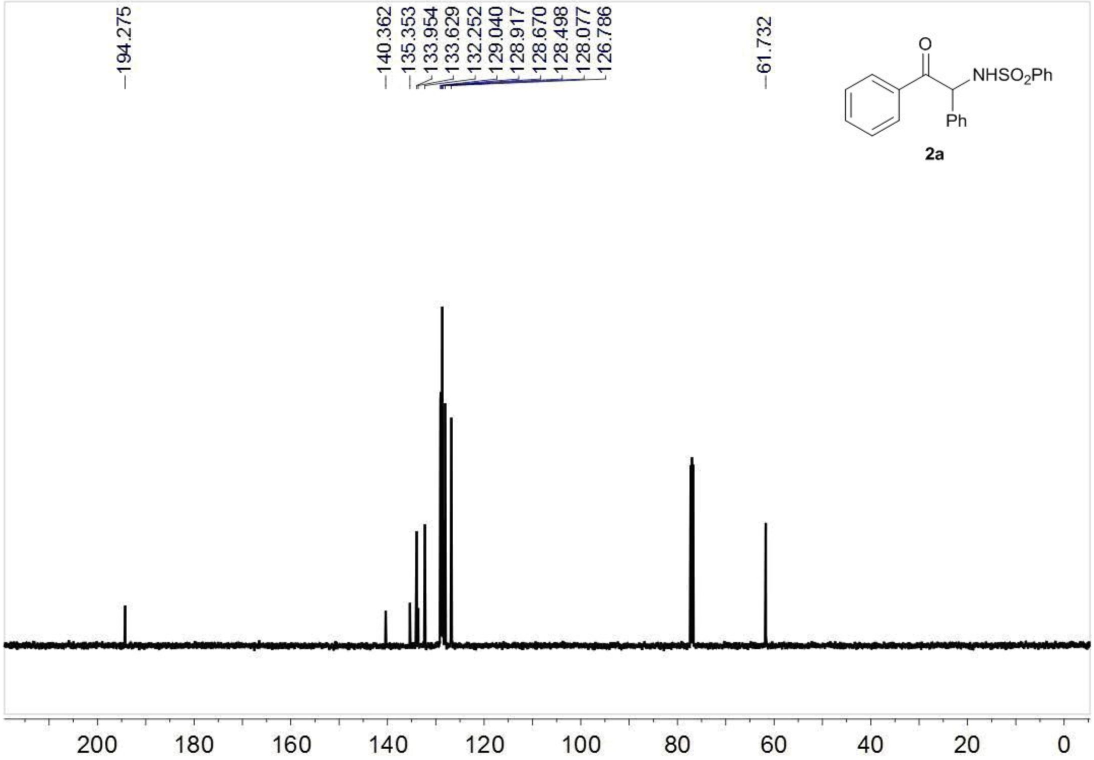

Supplementary Figure 2. ^{13}C NMR (125 MHz, CDCl$_3$) spectrum for 2a.

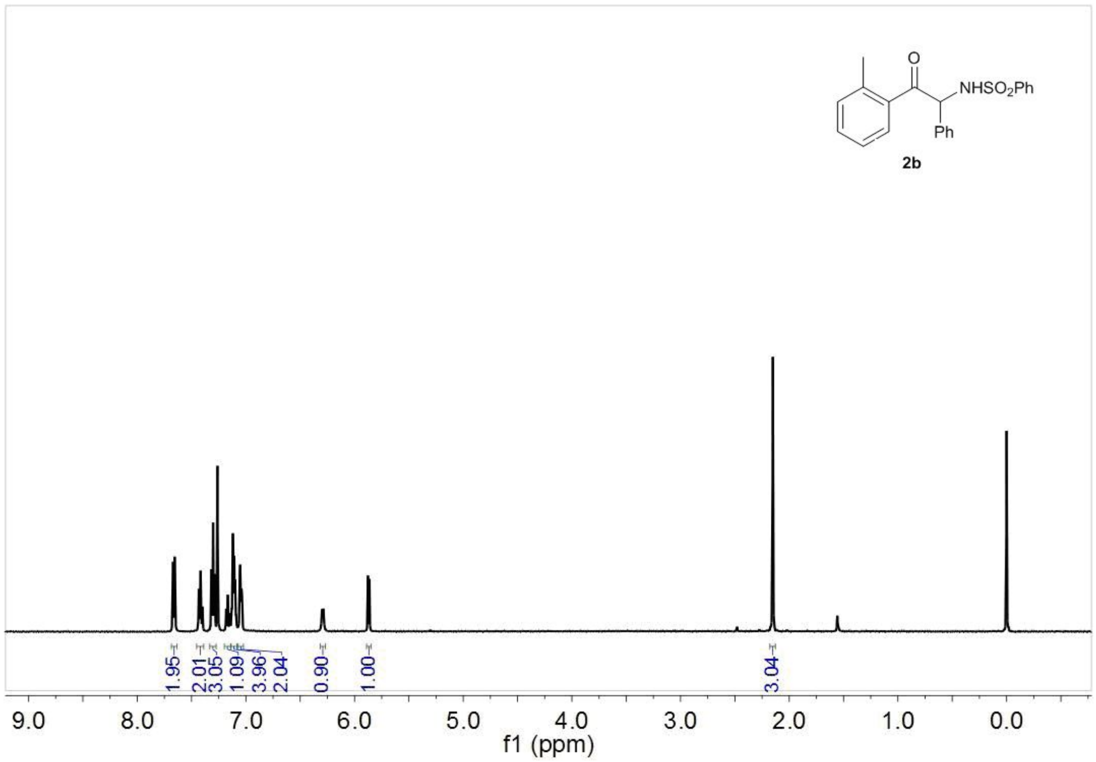

Supplementary Figure 3. ^{1}H NMR (500 MHz, $CDCl_3$) spectrum for 2b.

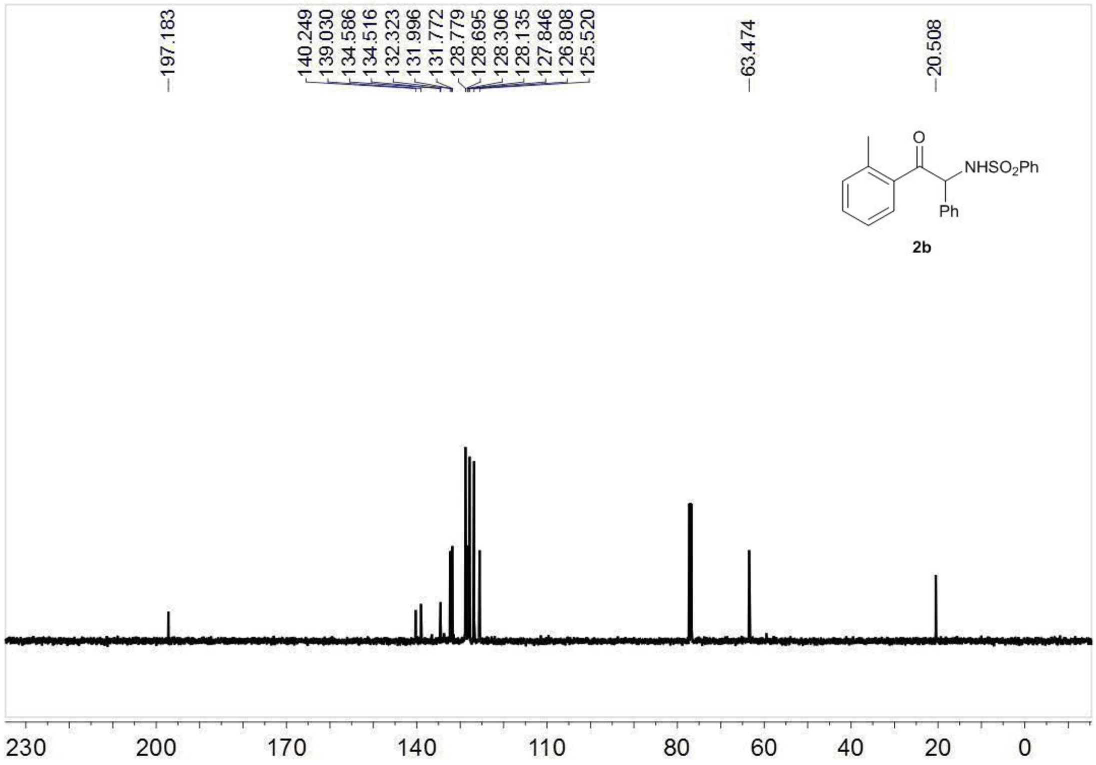

Supplementary Figure 4. ^{13}C NMR (125 MHz, $CDCl_3$) spectrum for 2b.

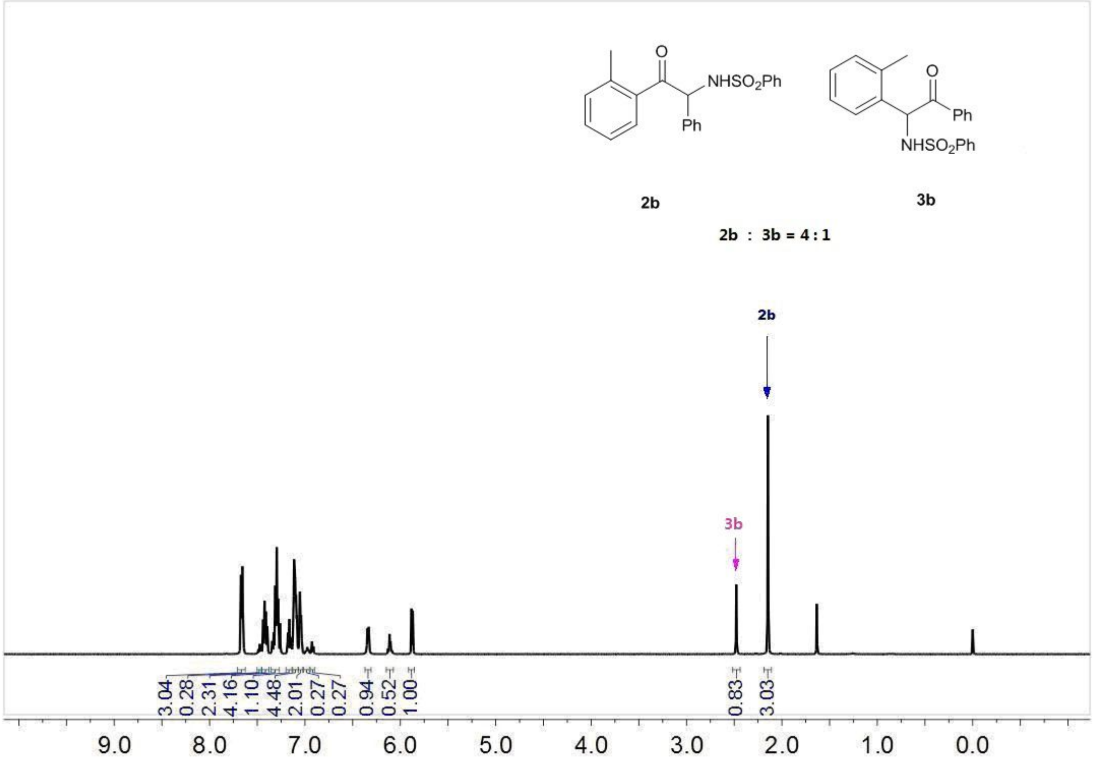

Supplementary Figure 5. ^{1}H NMR (500 MHz, $CDCl_3$) spectra for 2b and 3b.

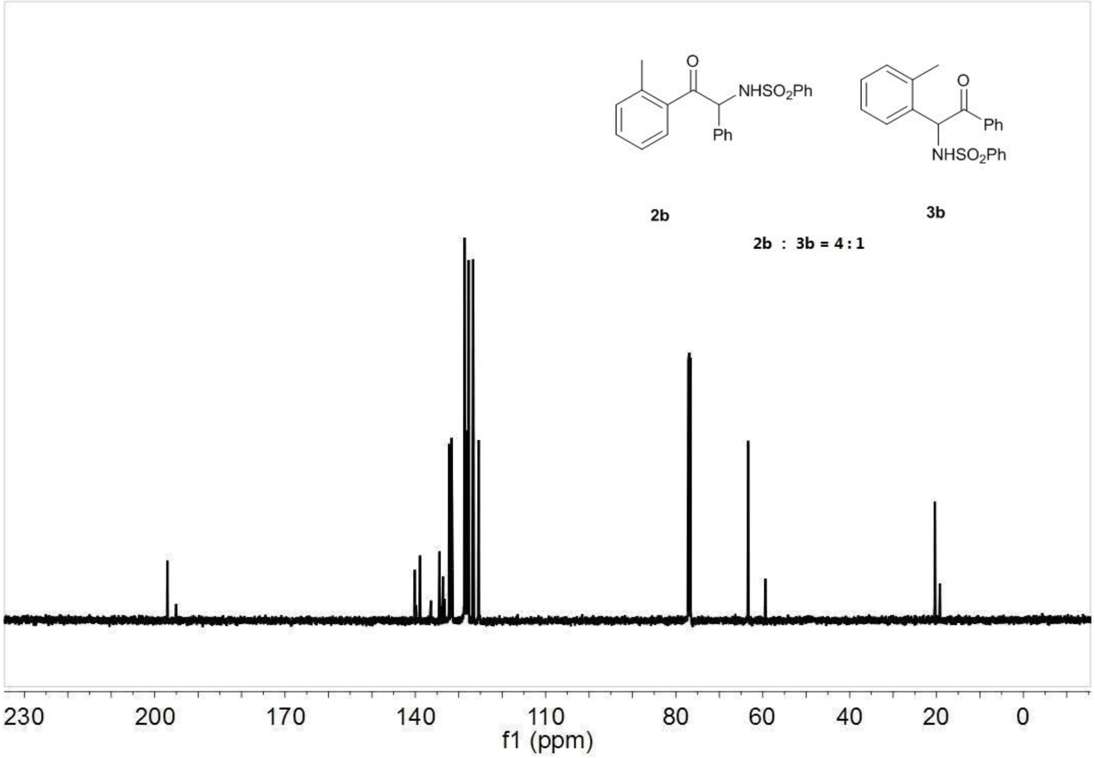

Supplementary Figure 6. ^{13}C NMR (125 MHz, $CDCl_3$) spectra for 2b and 3b.

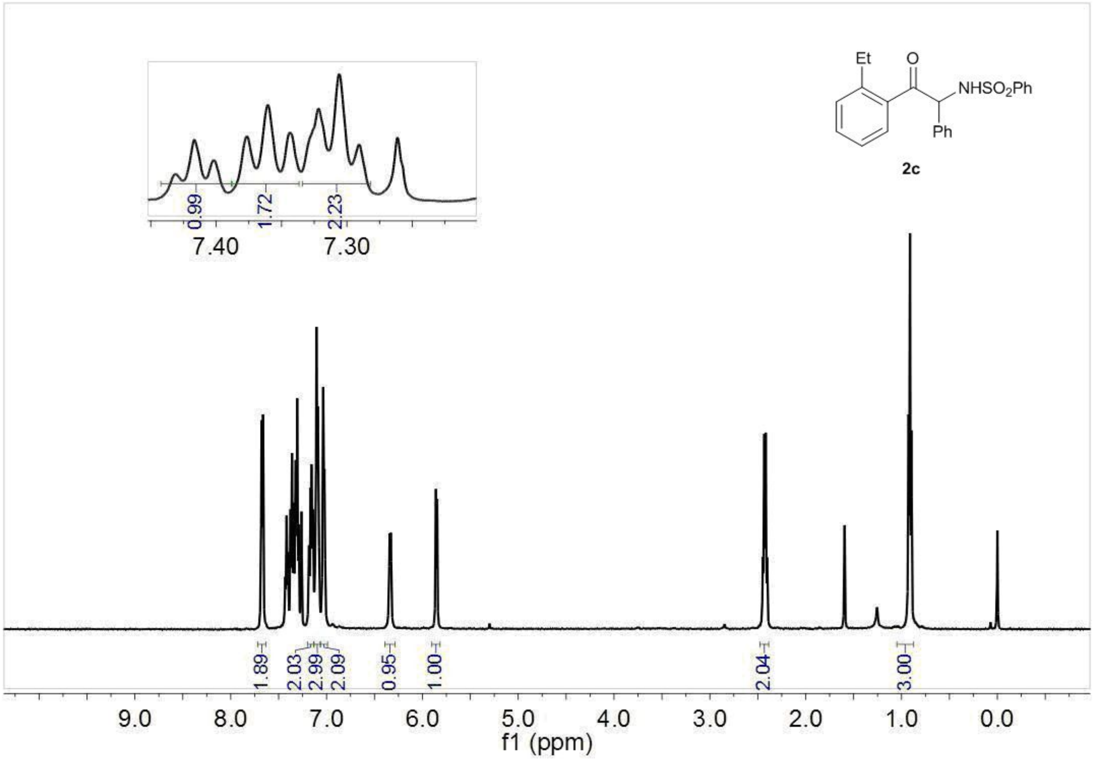

Supplementary Figure 7. ^{1}H NMR (500 MHz, $CDCl_3$) spectrum for 2c.

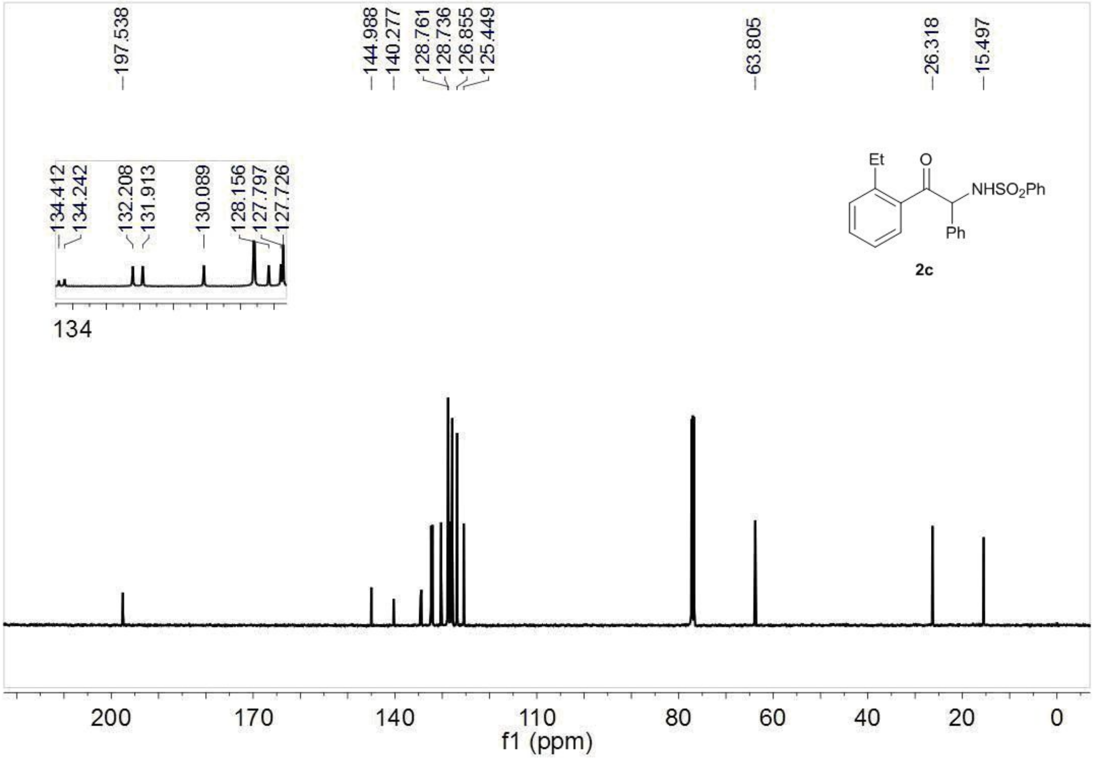

Supplementary Figure 8. ^{13}C NMR (125 MHz, $CDCl_3$) spectrum for 2c.

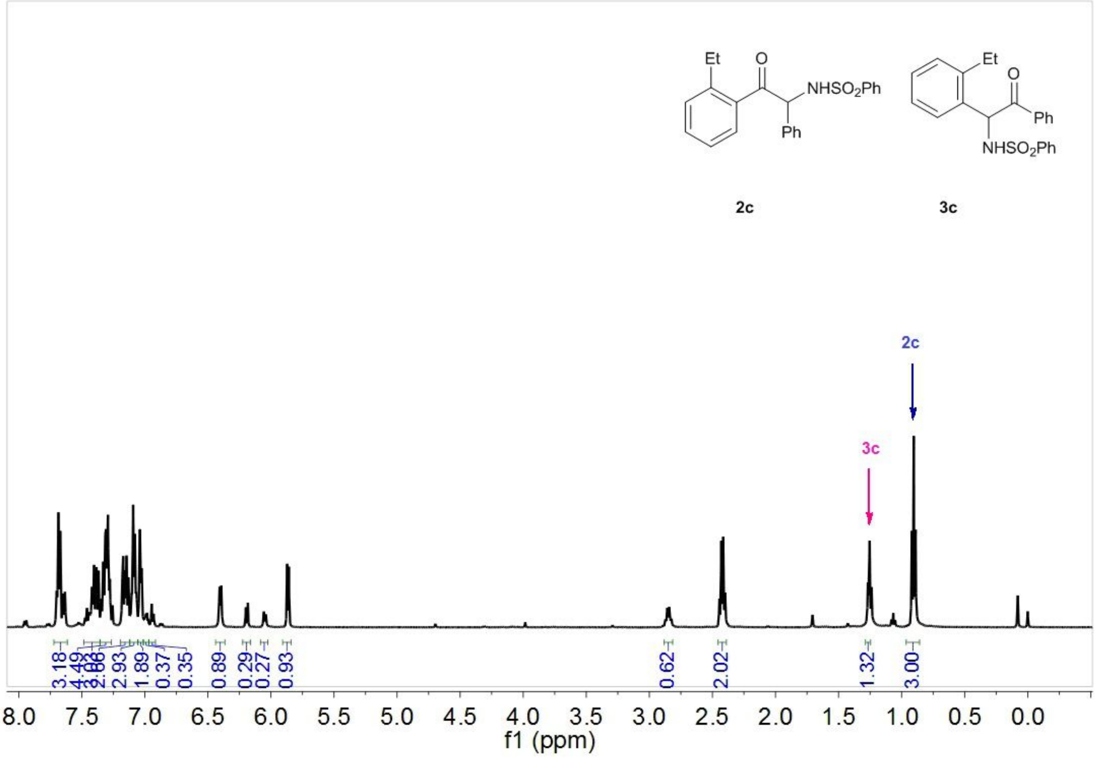

Supplementary Figure 9. ^{1}H NMR (500 MHz, $CDCl_3$) spectra for 2c and 3c.

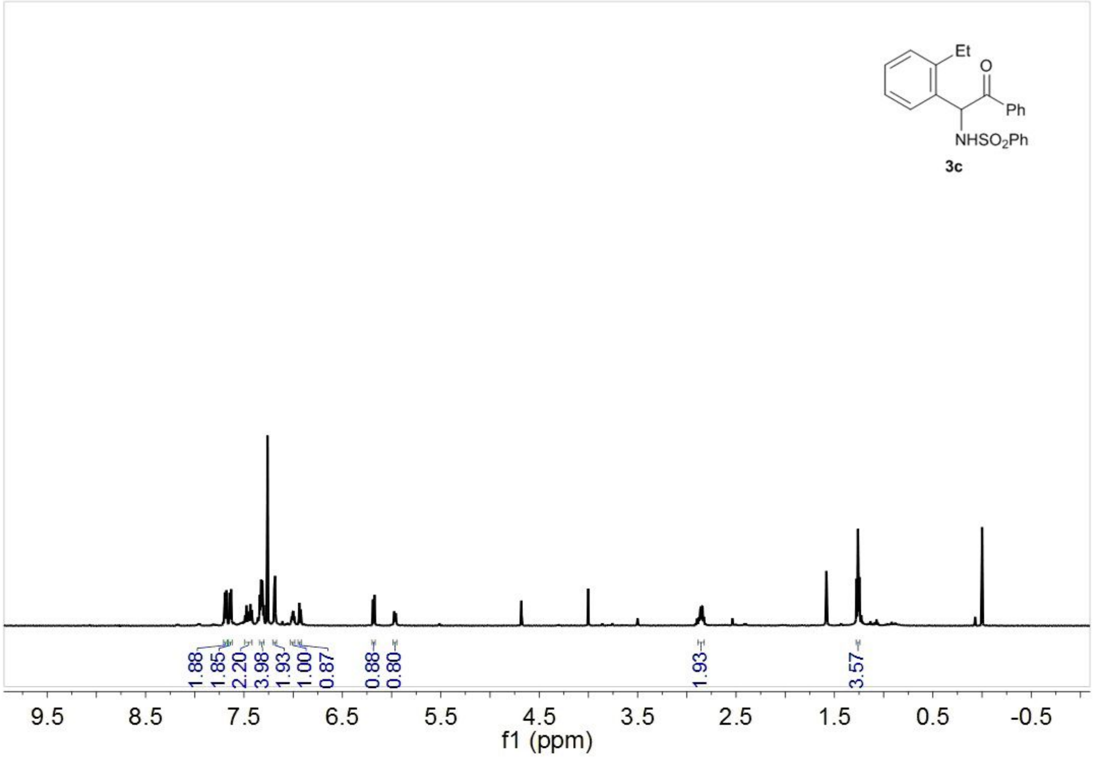

Supplementary Figure 10. ^{1}H NMR (500 MHz, $CDCl_3$) spectrum for 3c.

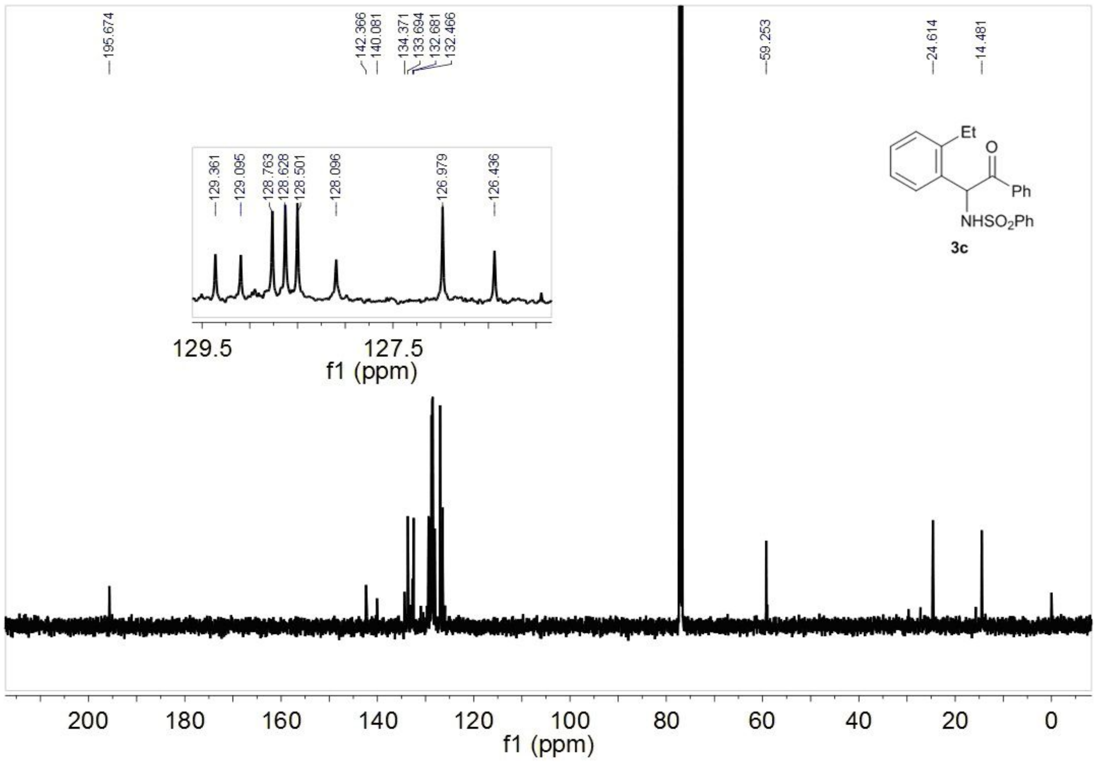

Supplementary Figure 11. ^{13}C NMR (125 MHz, $CDCl_3$) spectrum for 3c.

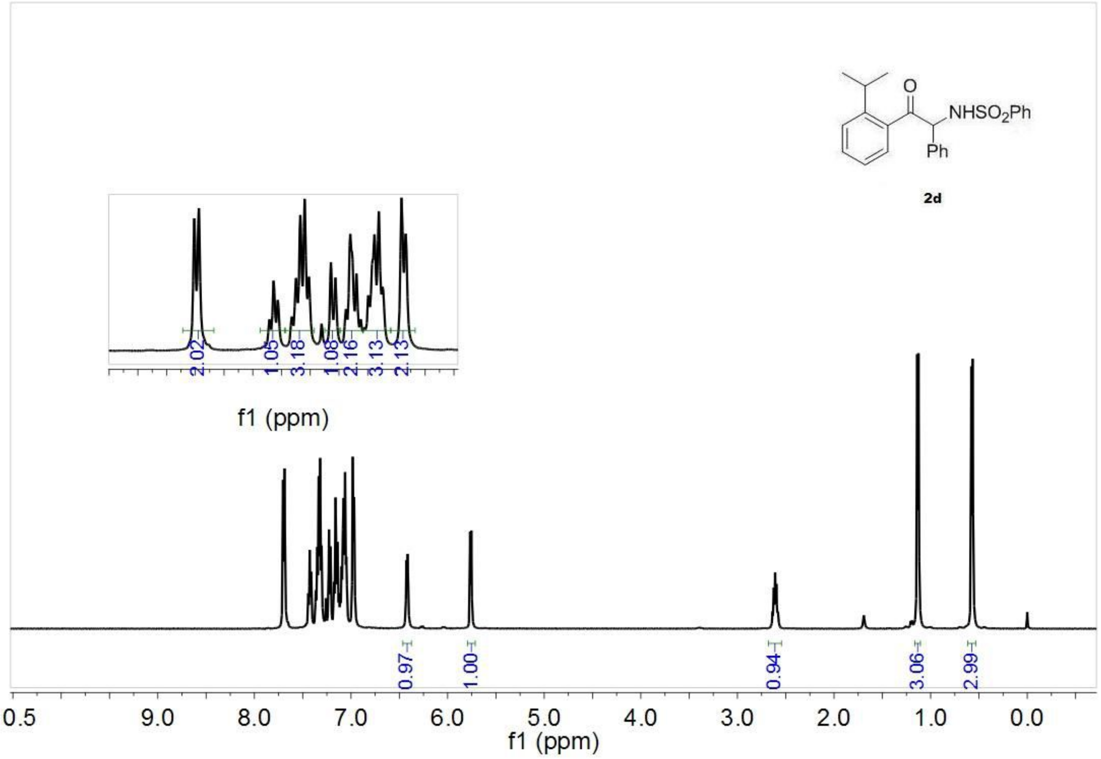

Supplementary Figure 12. ^{1}H NMR (500 MHz, $CDCl_3$) spectrum for 2d.

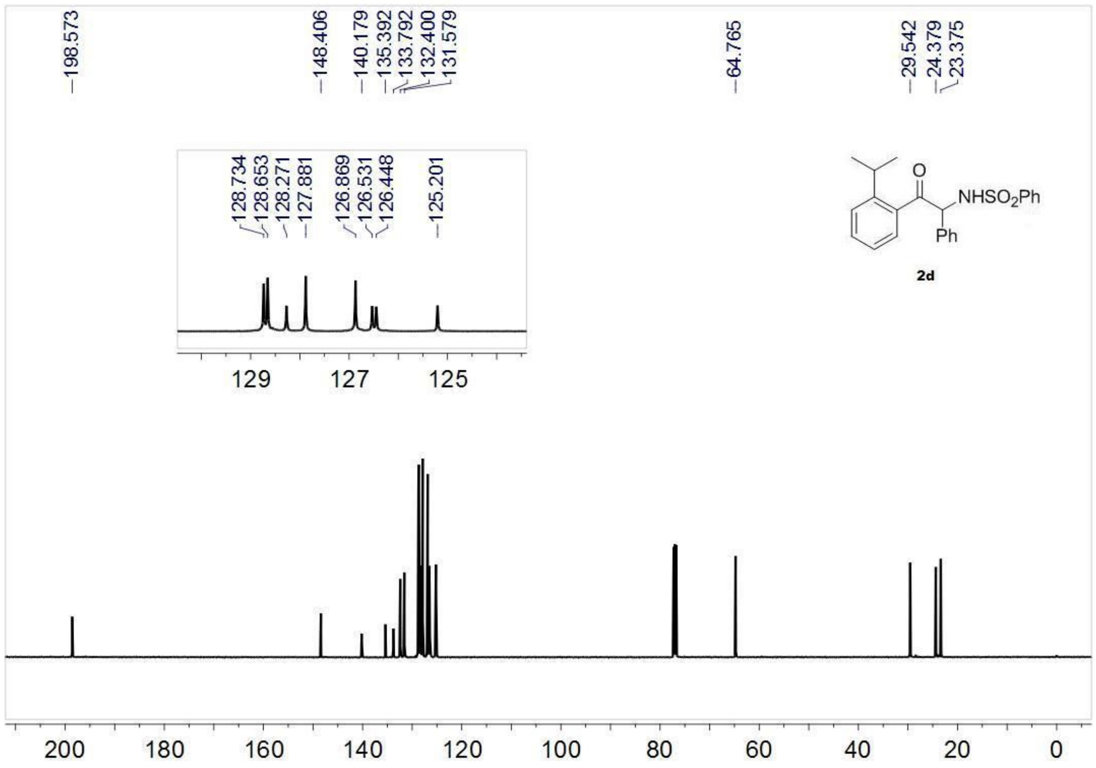

Supplementary Figure 13. ^{13}C NMR (125 MHz, $CDCl_3$) spectrum for 2d.

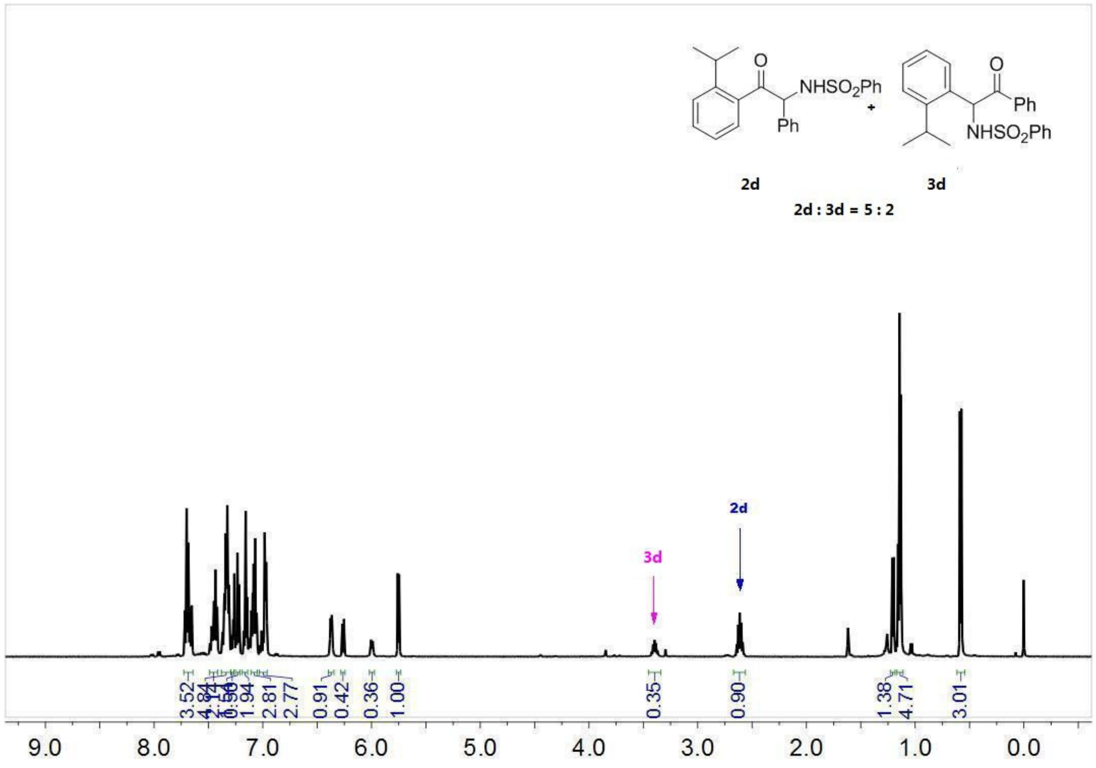

Supplementary Figure 14. ^{1}H NMR (500 MHz, $CDCl_3$) spectra for 2d and 3d.

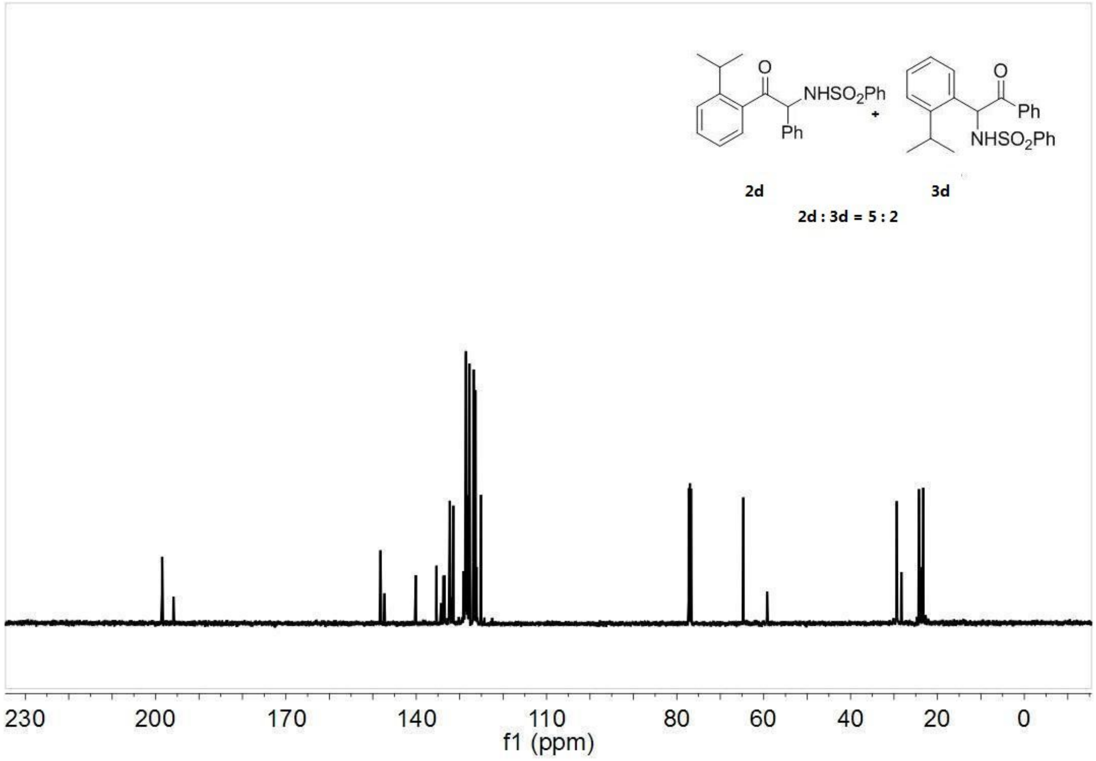

Supplementary Figure 15. ^{13}C NMR (125 MHz, $CDCl_3$) spectra for 2d and 3d.

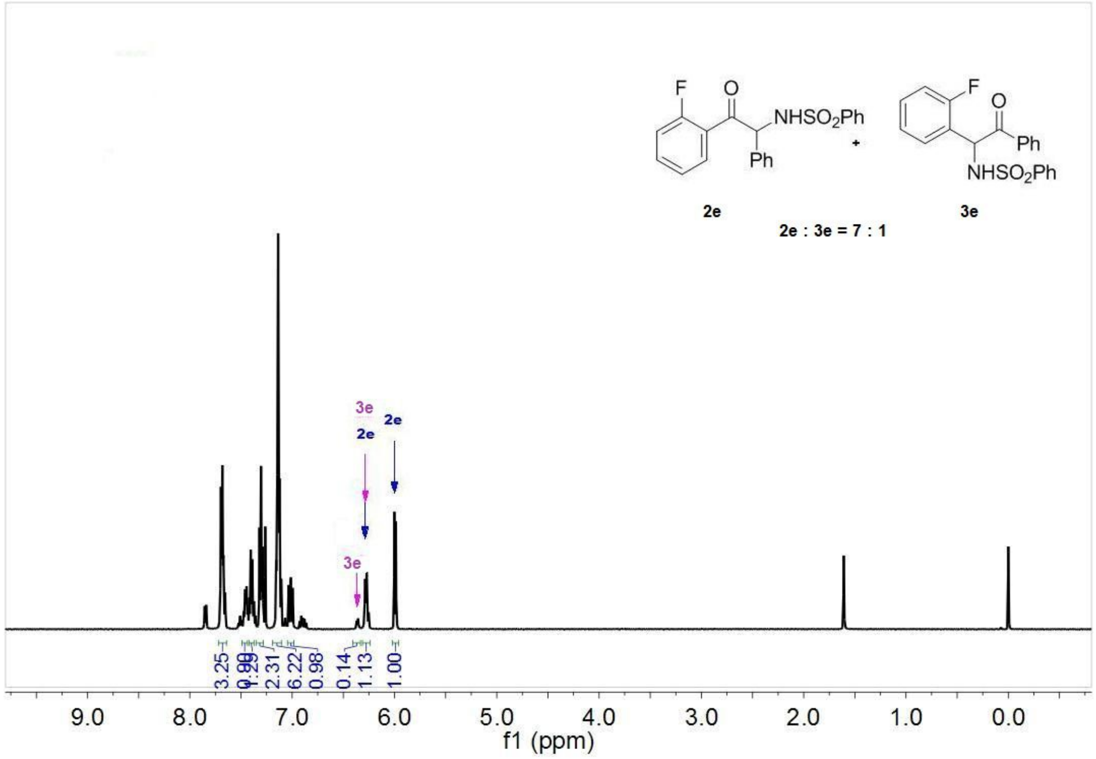

Supplementary Figure 16. ^{1}H NMR (500 MHz, $CDCl_3$) spectra for 2e and 3e.

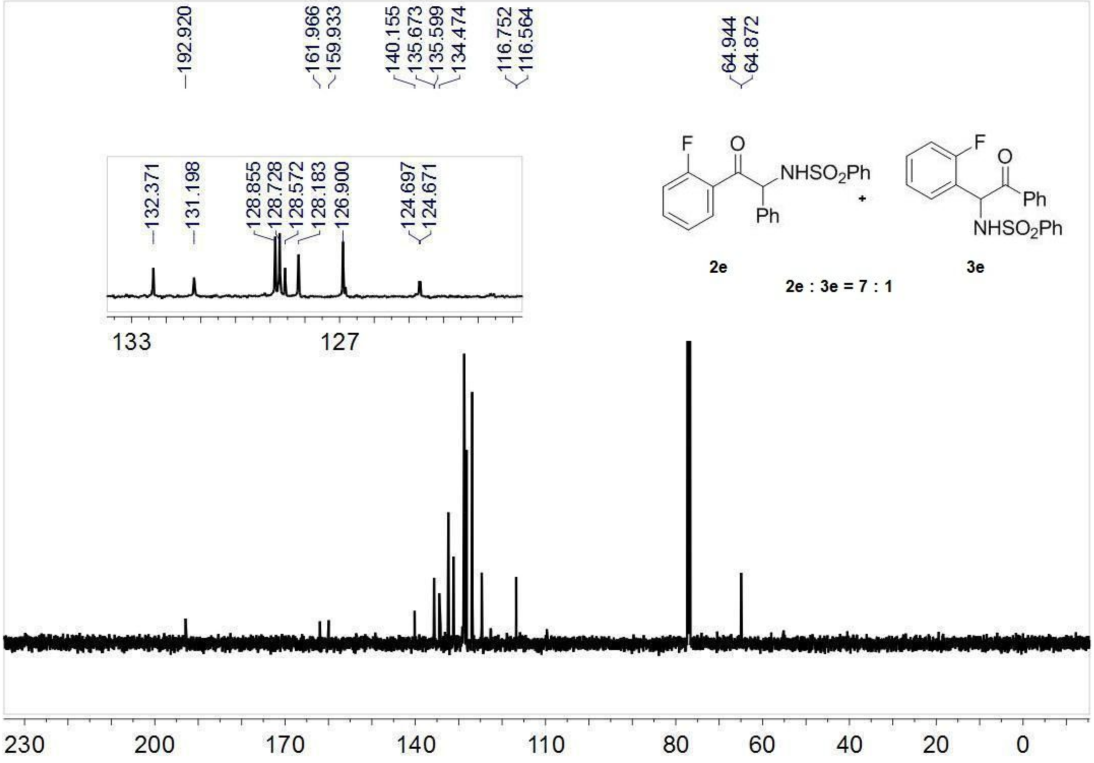

Supplementary Figure 17. ^{13}**C NMR (125 MHz, CDCl**$_3$**) spectra for 2e and 3e.**

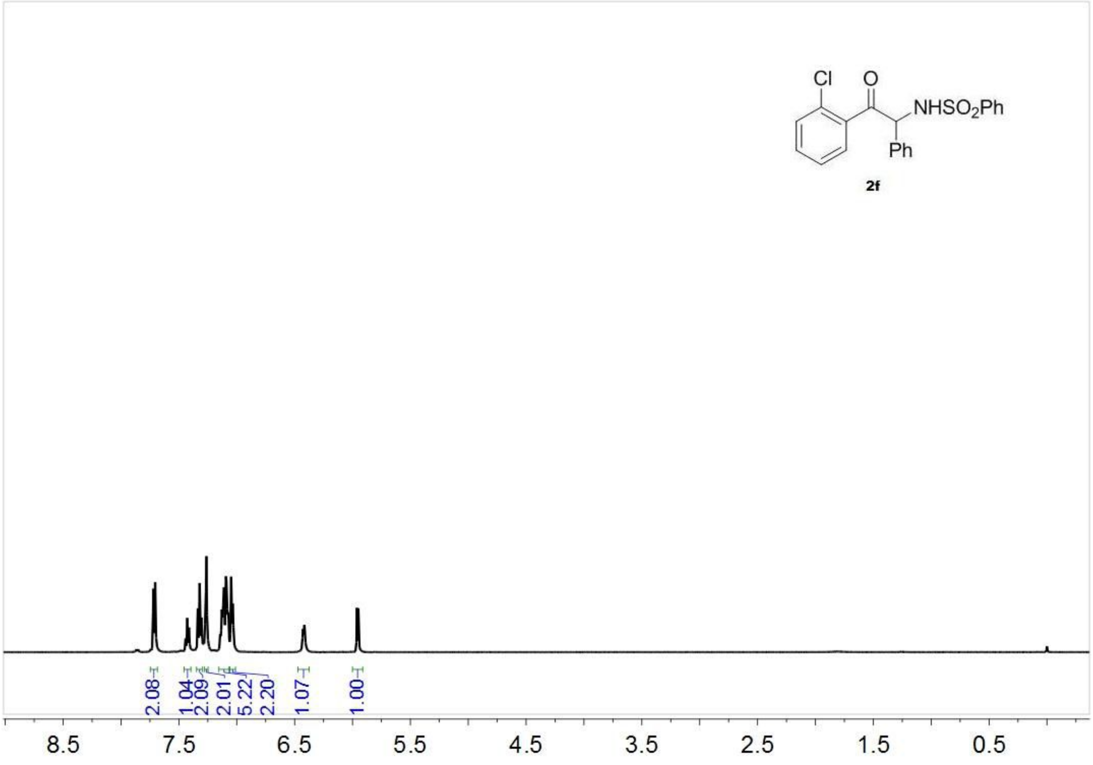

Supplementary Figure 18. ^{1}**H NMR (500 MHz, CDCl**$_3$**) spectrum for2f.**

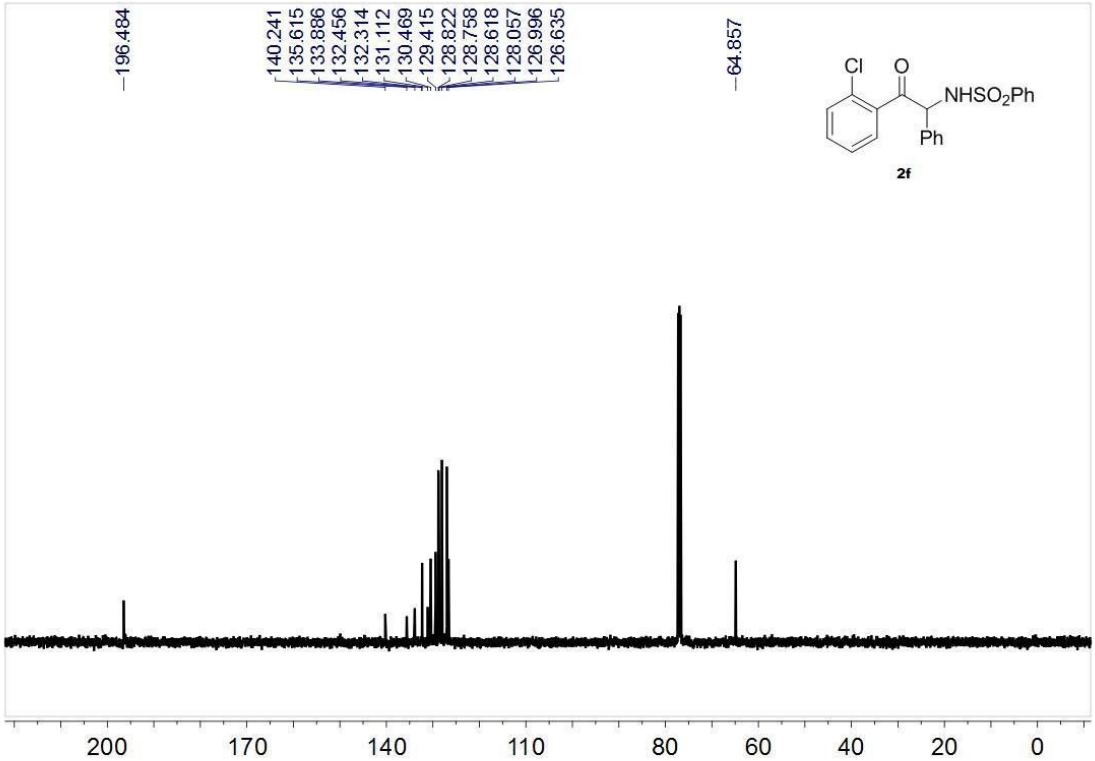

Supplementary Figure 19. ^{13}C NMR (125 MHz, $CDCl_3$) spectrum for 2f.

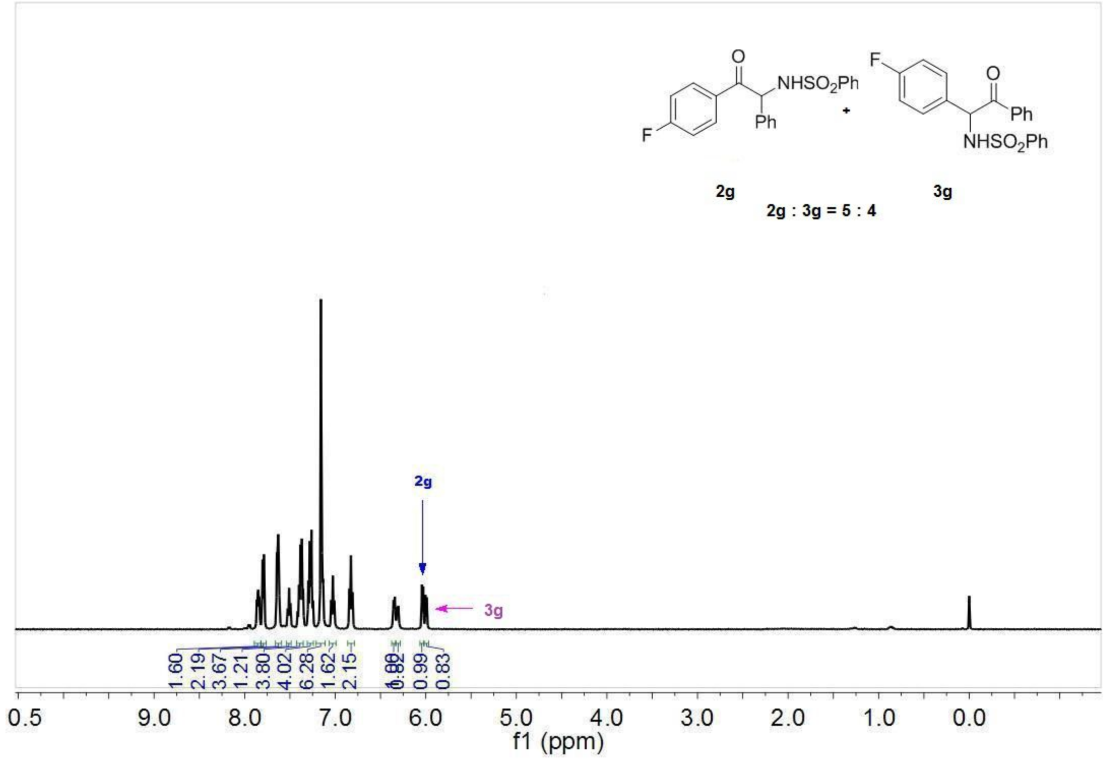

Supplementary Figure 20. ^{1}H NMR (500 MHz, $CDCl_3$) spectra for2g and 3g.

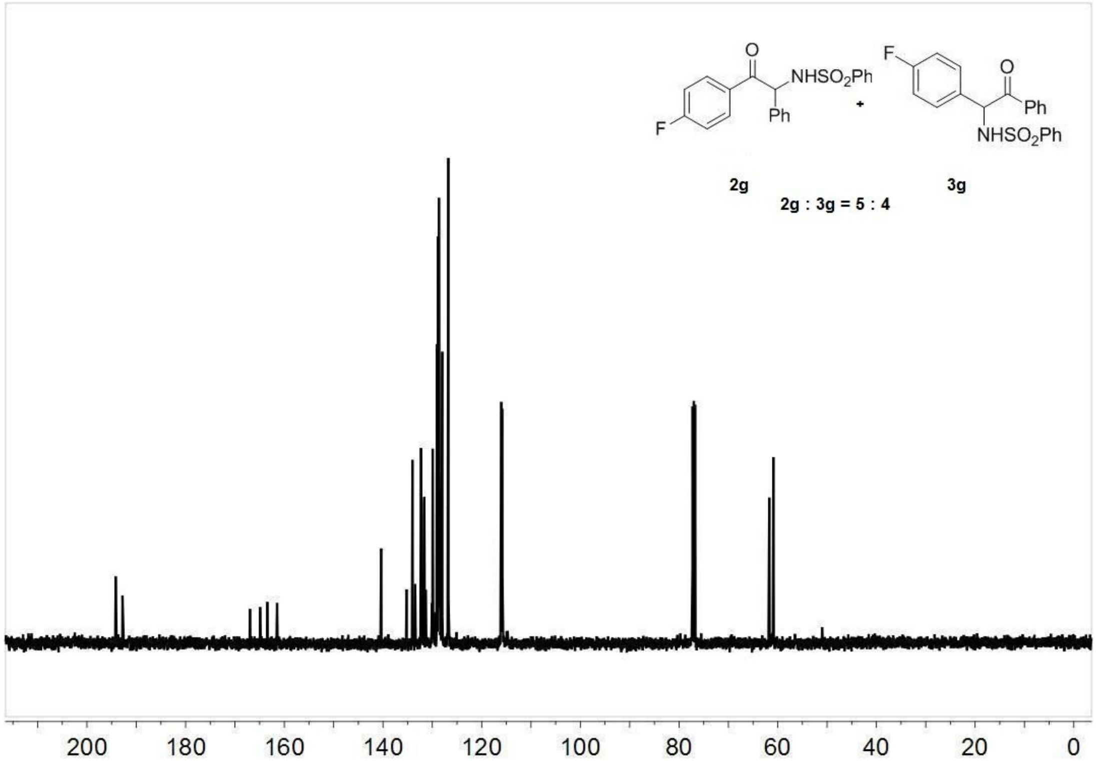

Supplementary Figure 21. ^{13}C NMR (125MHz, CDCl$_3$) spectra for 2g and 3g.

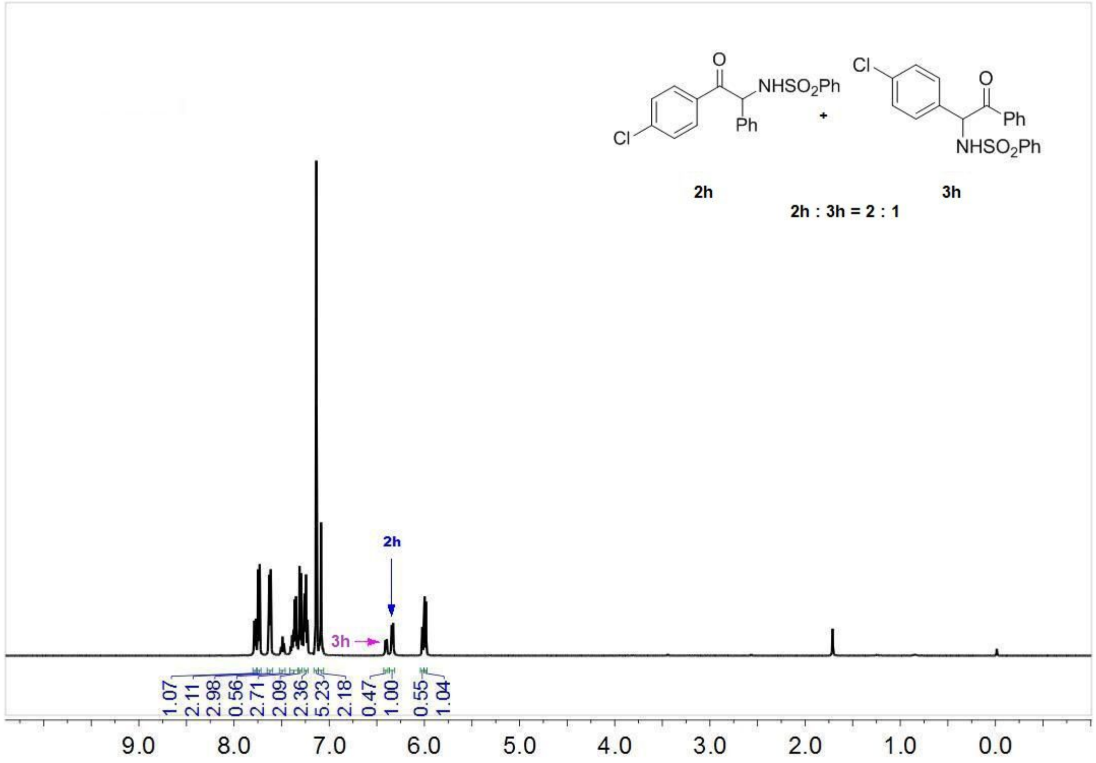

Supplementary Figure 22. ^{1}H NMR (500 MHz, CDCl$_3$) spectra for 2h and 3h.

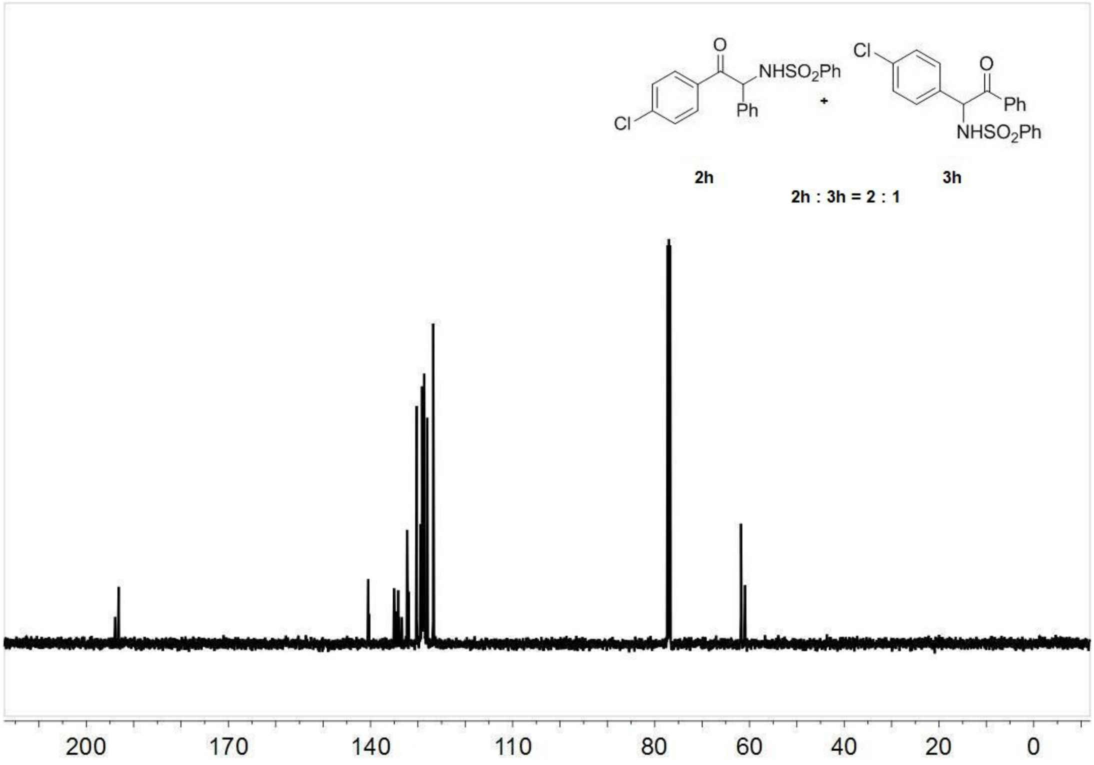

Supplementary Figure 23. ^{13}C NMR (125 MHz, $CDCl_3$) spectra for 2h and 3h.

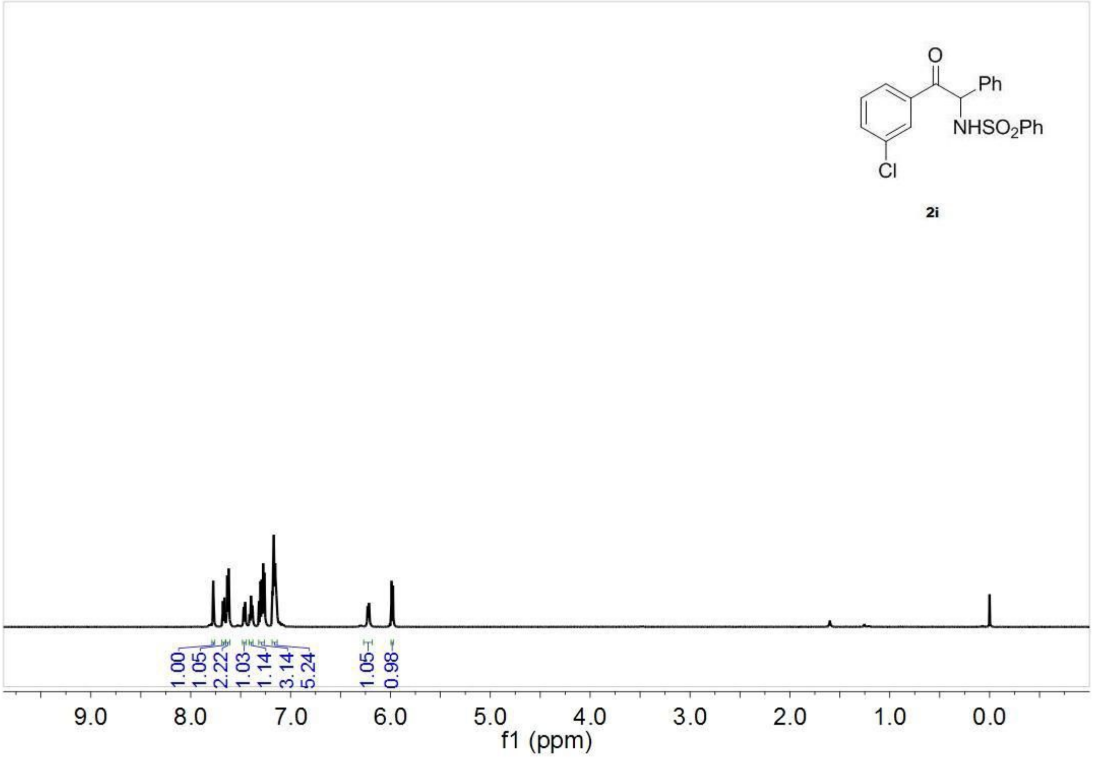

Supplementary Figure 24. ^{1}H NMR (500 MHz, $CDCl_3$) spectrum for 2i.

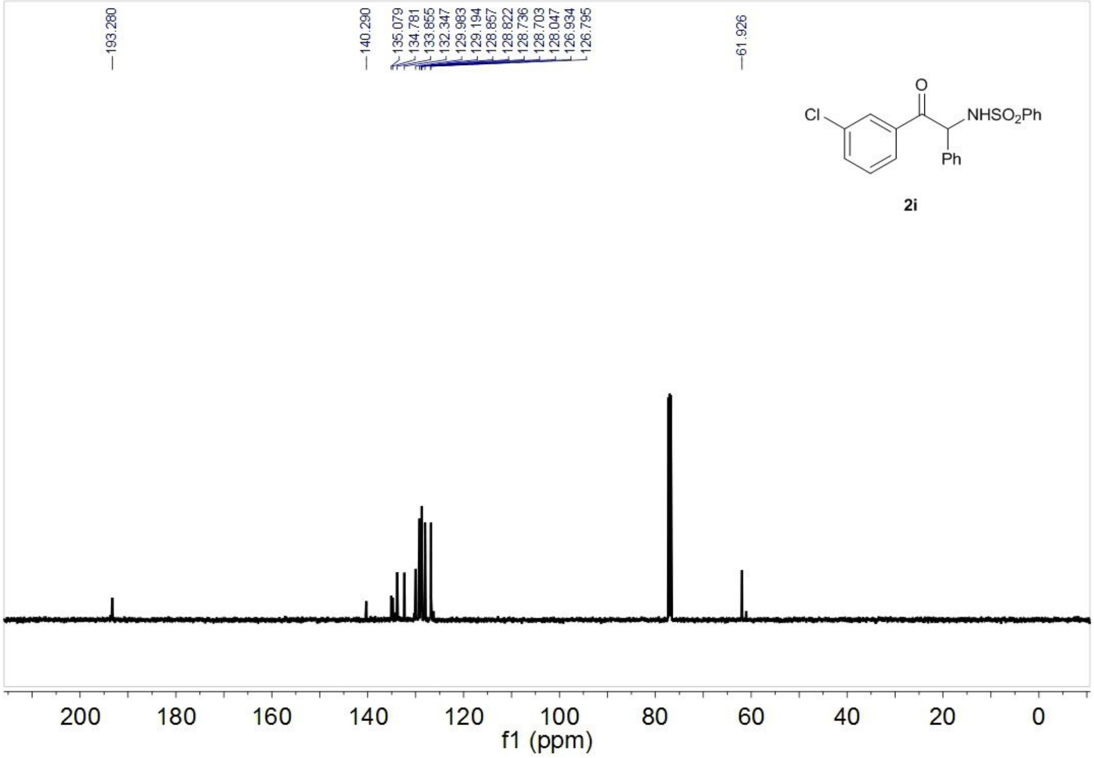

Supplementary Figure 25. ^{13}C NMR (125 MHz, $CDCl_3$) spectrum for 2i.

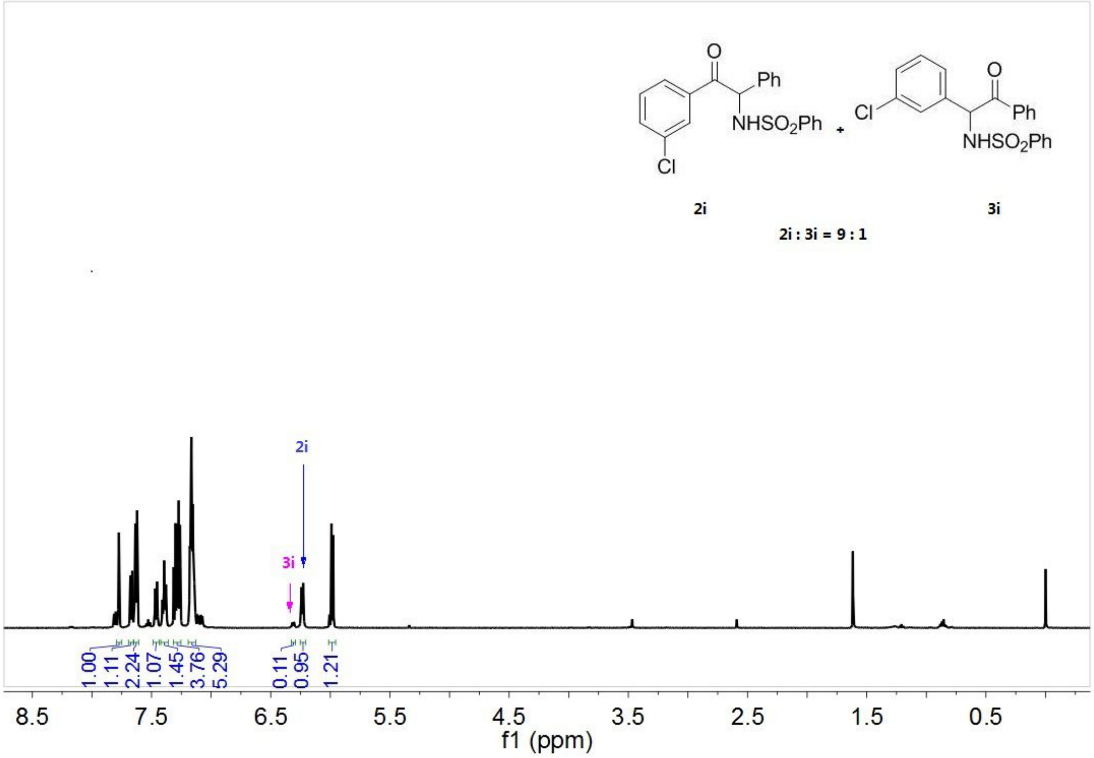

Supplementary Figure 26. ^{1}H NMR (500 MHz, $CDCl_3$) spectra for 2i and 3i.

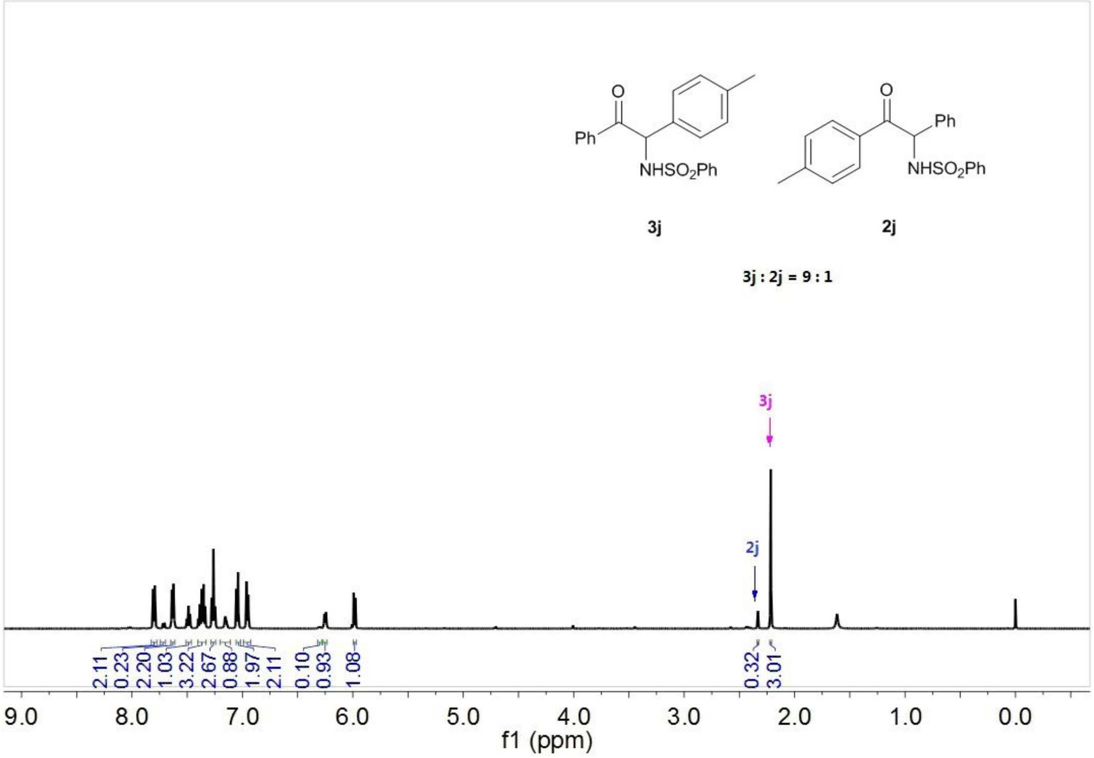

Supplementary Figure 27. ^{1}H NMR (500 MHz, $CDCl_3$) spectra for 3j and 2j.

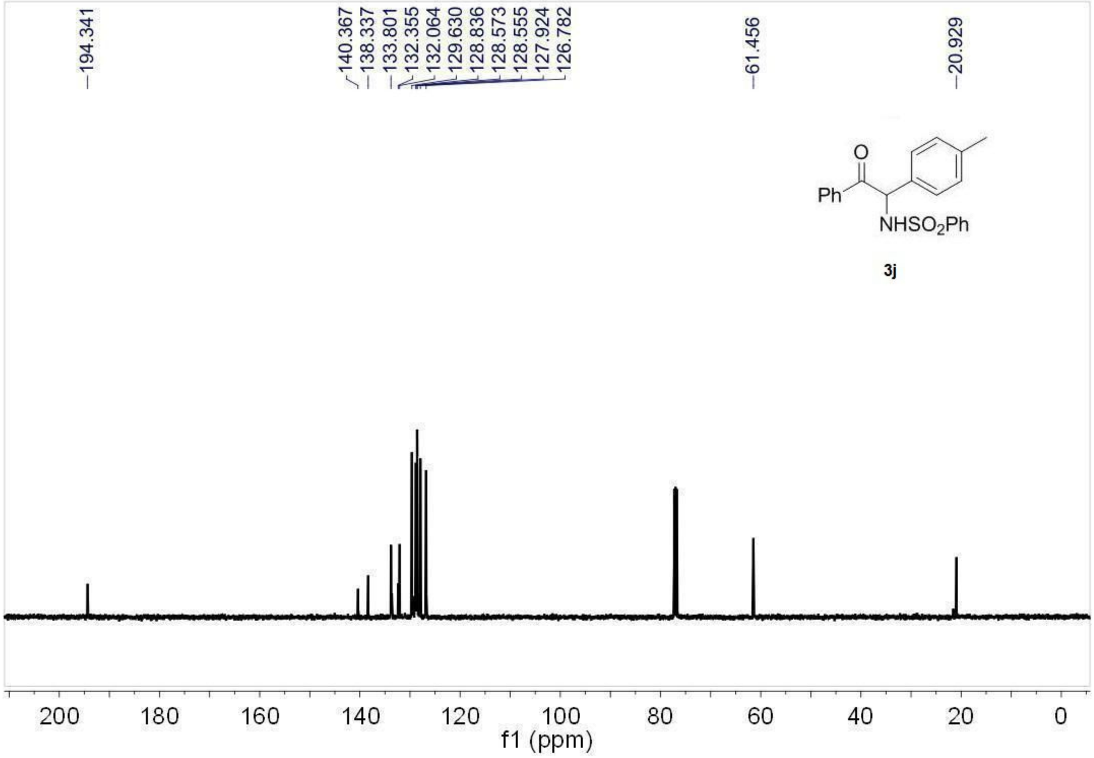

Supplementary Figure 28. ^{13}C NMR (125 MHz, $CDCl_3$) spectra for 3j and 2j.

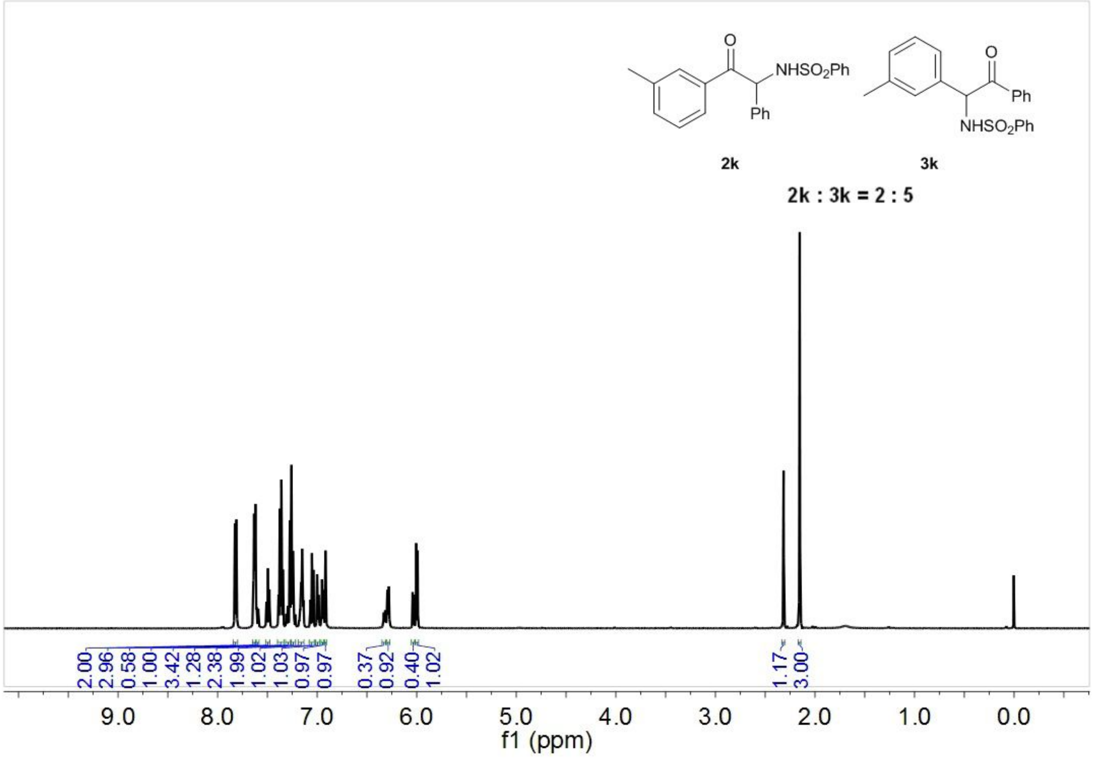

Supplementary Figure 29. ^{1}H NMR (500 MHz, $CDCl_3$) spectra for 2k and 3k.

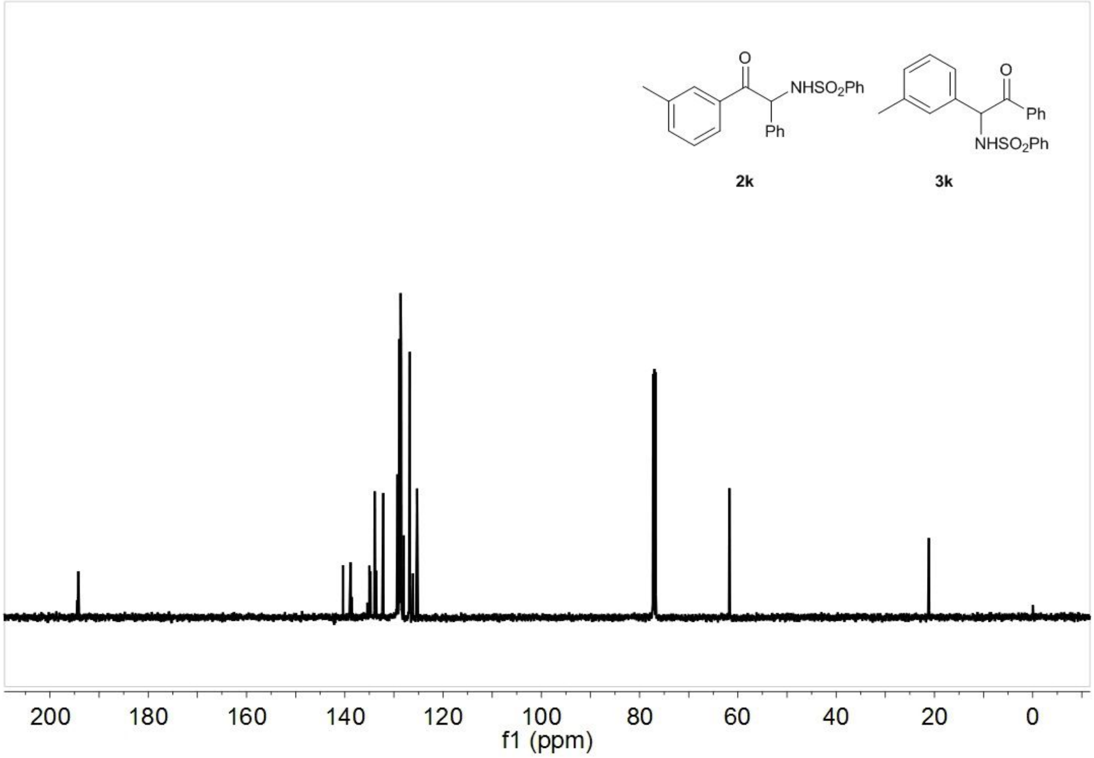

Supplementary Figure 30. ^{13}C NMR (125 MHz, $CDCl_3$) spectra for 2k and 3k.

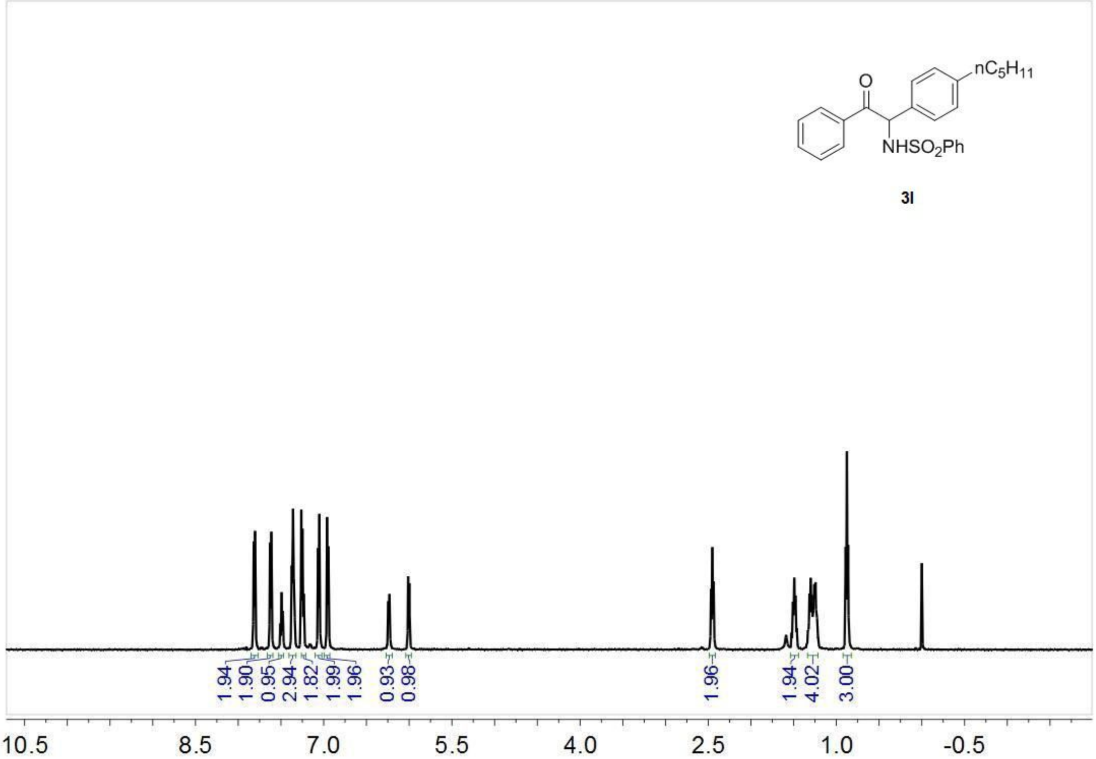

Supplementary Figure 31. ^{1}H NMR (500 MHz, CDCl$_3$) spectrum for 3l.

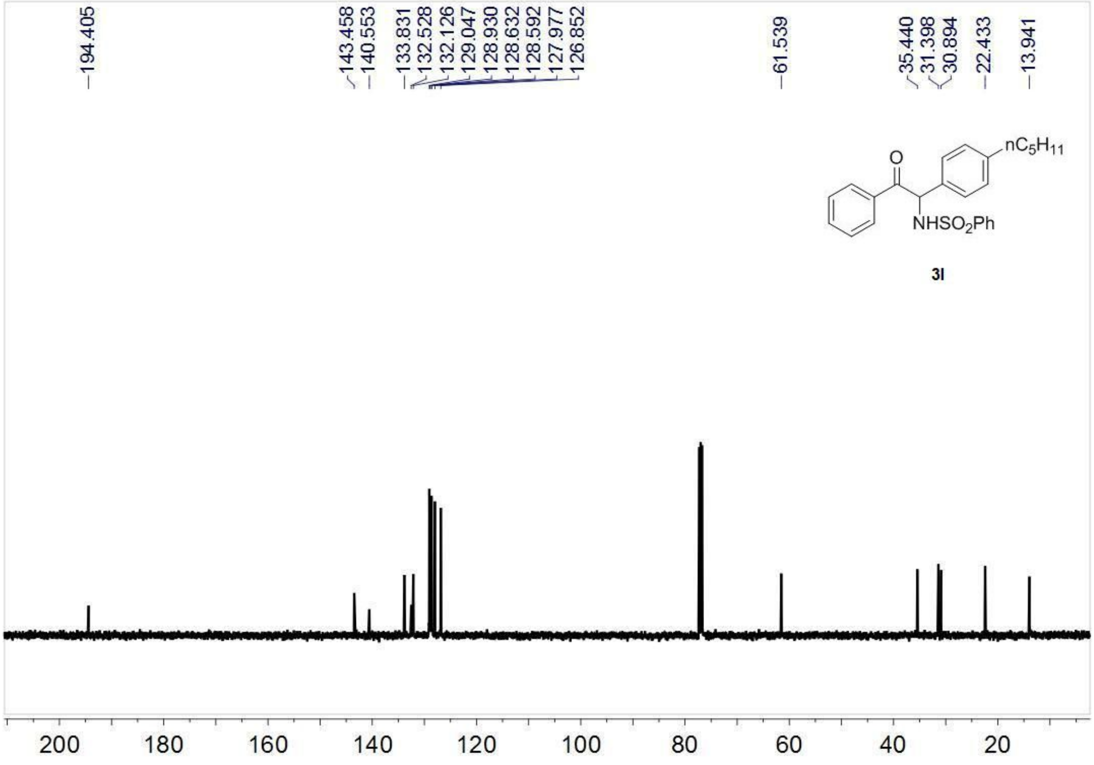

Supplementary Figure 32. ^{13}C NMR (125 MHz, CDCl$_3$) spectrum for 3l.

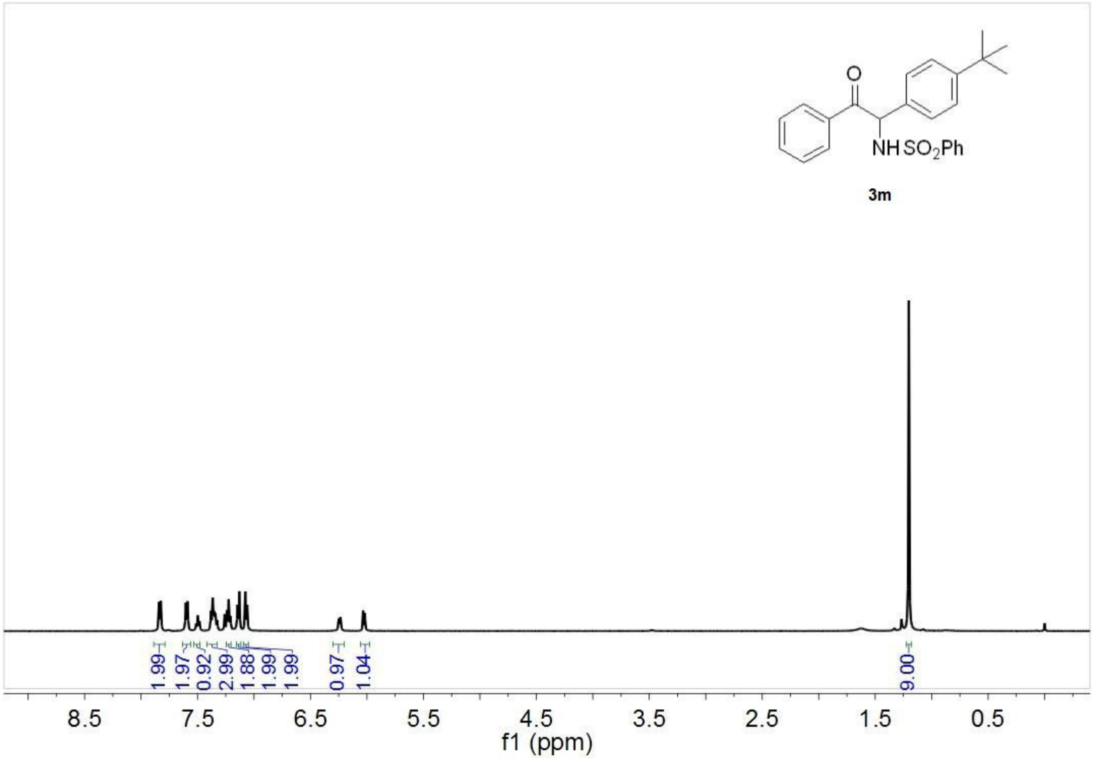

Supplementary Figure 33. ^{1}H NMR (500 MHz, CDCl₃) spectrum for 3m.

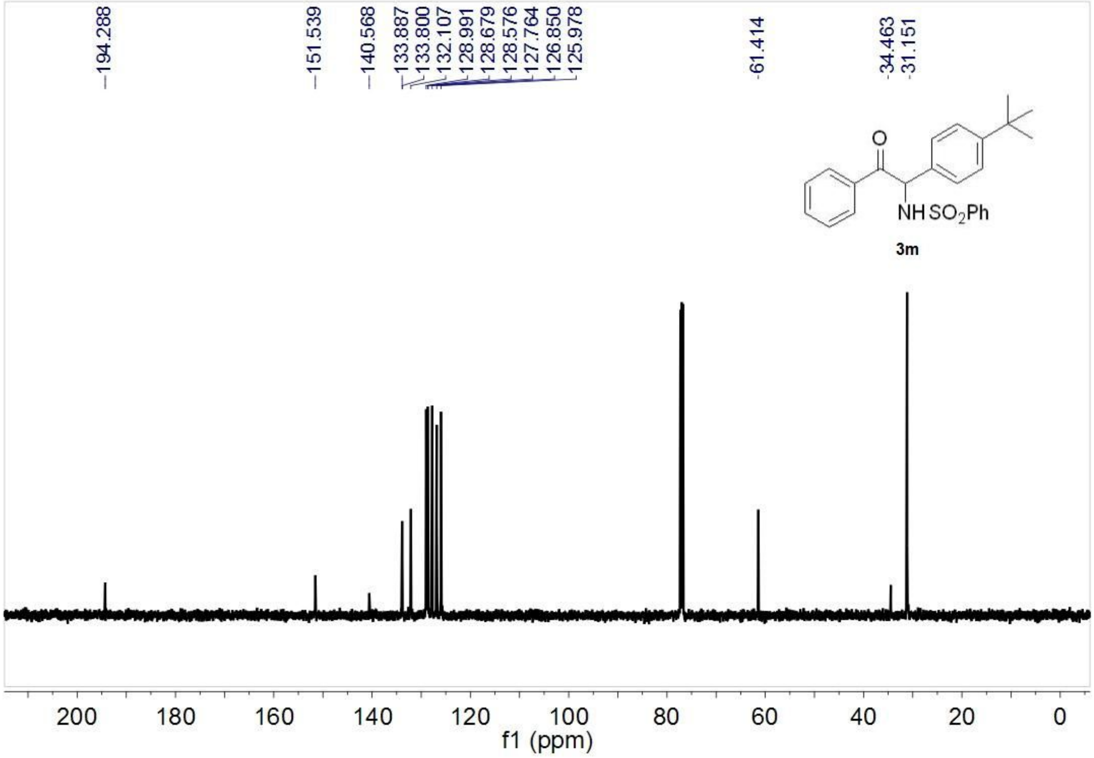

Supplementary Figure 34. ^{13}C NMR (125 MHz, CDCl₃) spectrum for 3m.

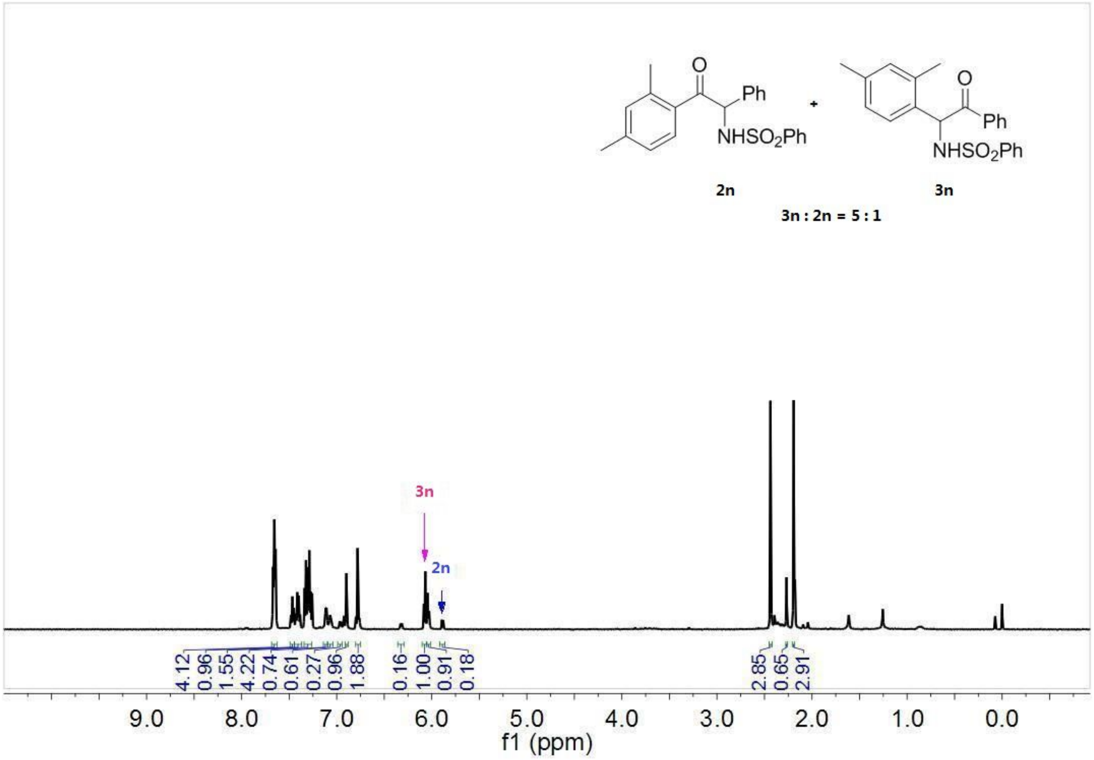

Supplementary Figure 35. ^{1}H NMR (500 MHz, $CDCl_3$) spectra for 2n and 3n.

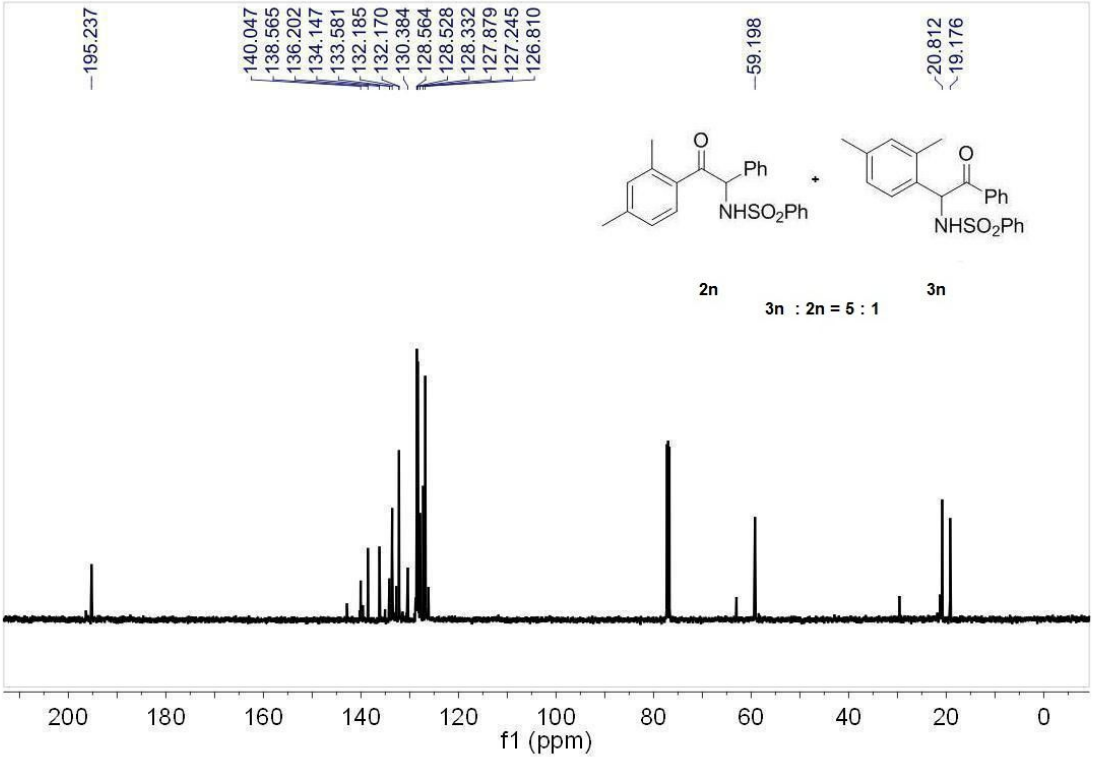

Supplementary Figure 36. ^{13}C NMR (125 MHz, $CDCl_3$) spectra for 2n and 3n.

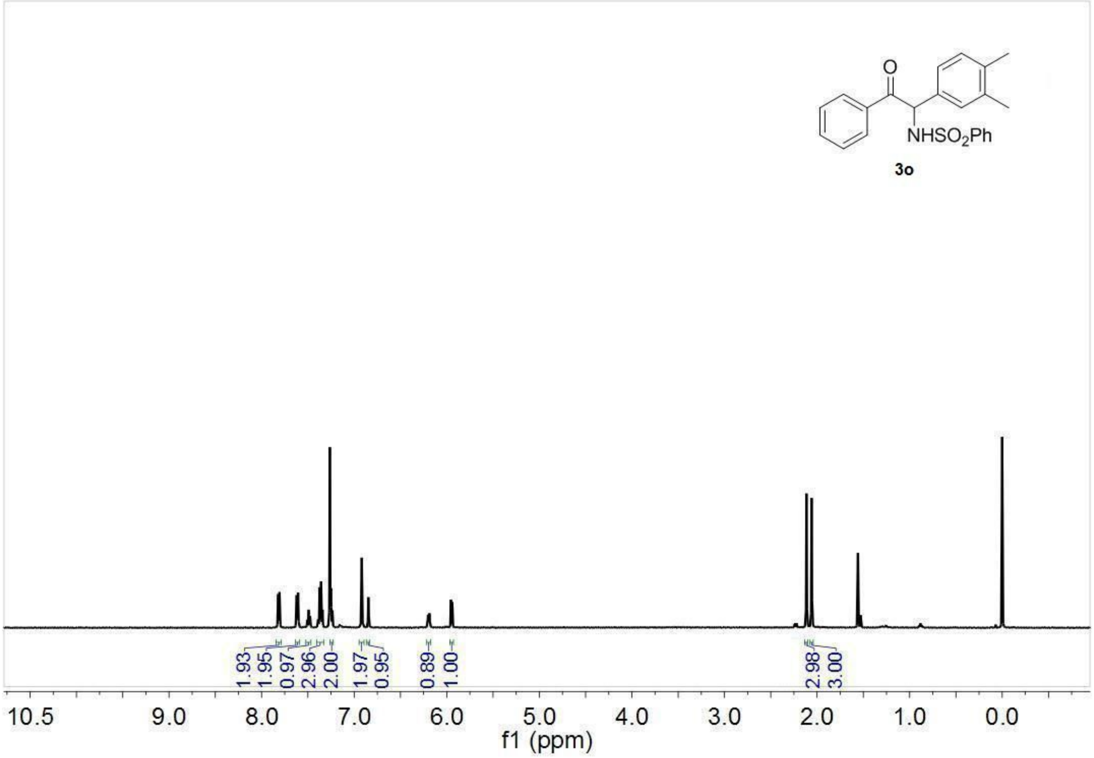

Supplementary Figure 37. ^{1}H NMR (500 MHz, $CDCl_3$) spectrum for 3o.

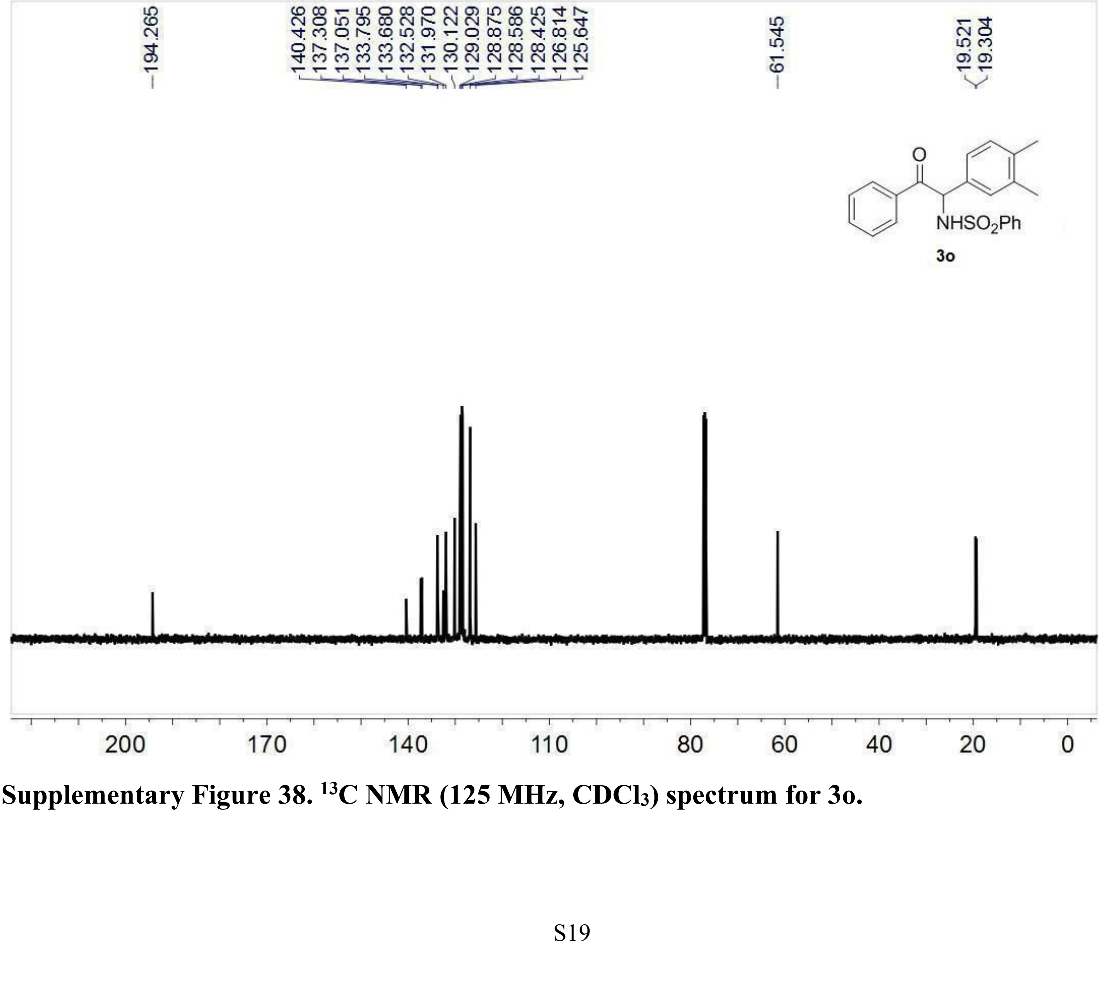

Supplementary Figure 38. ^{13}C NMR (125 MHz, $CDCl_3$) spectrum for 3o.

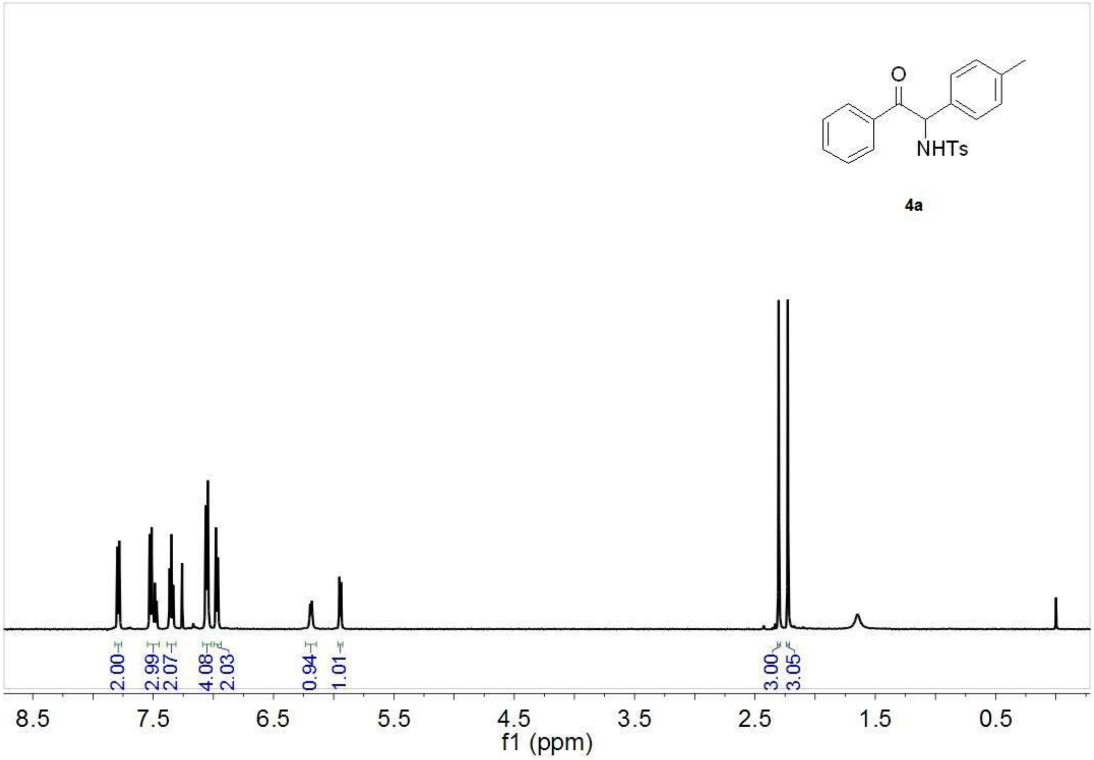

Supplementary Figure 39. ^{1}**H NMR (500 MHz, CDCl**$_3$**) spectrum for 4a.**

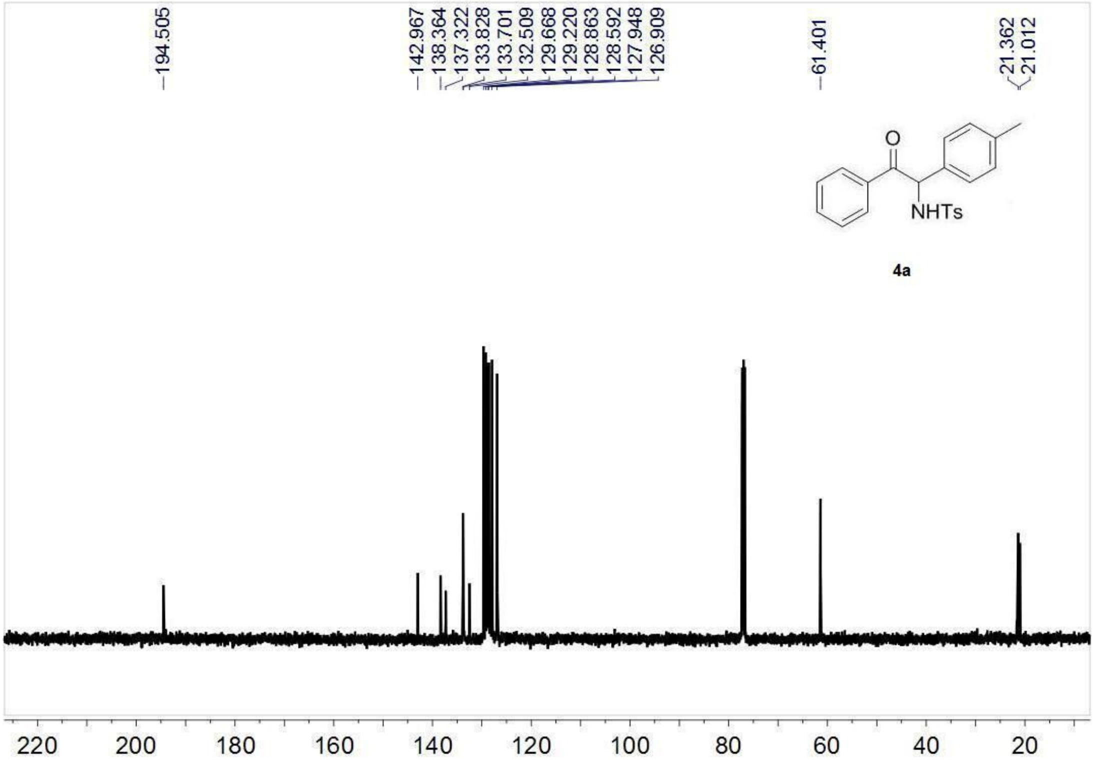

Supplementary Figure 40. ^{13}**C NMR (125 MHz, CDCl**$_3$**) spectrum for 4a.**

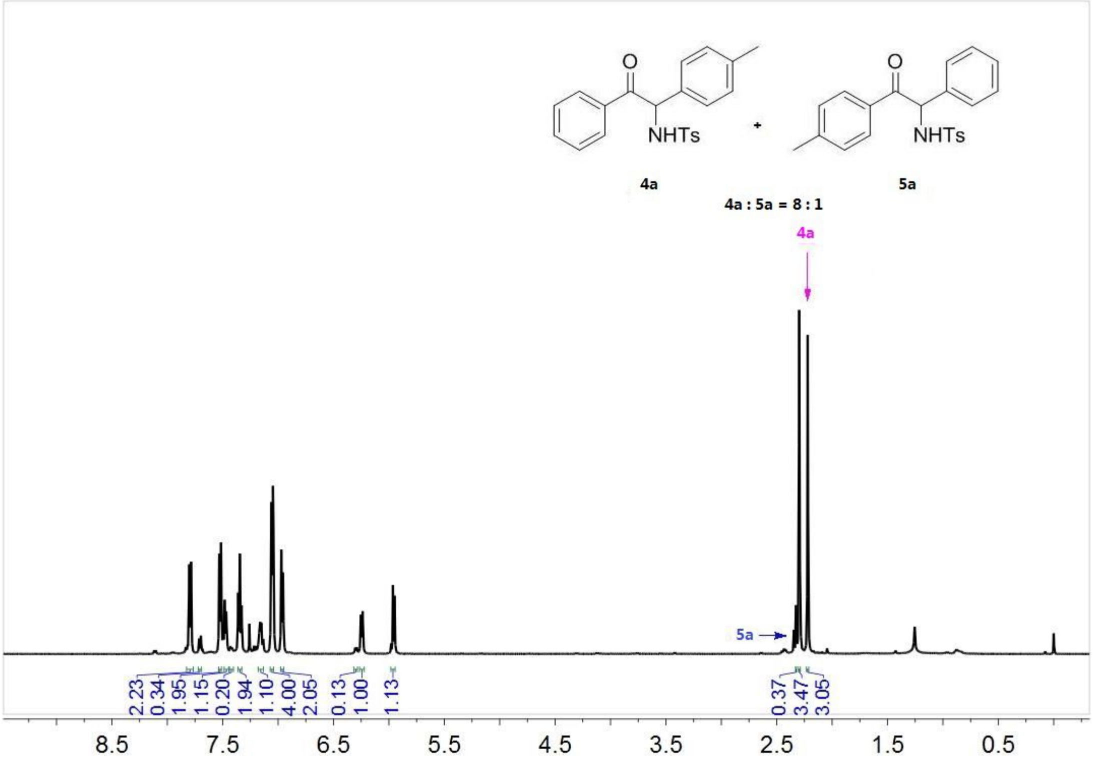

Supplementary Figure 41. ^{1}H NMR (500 MHz, CDCl$_3$) spectra for 4a and 5a.

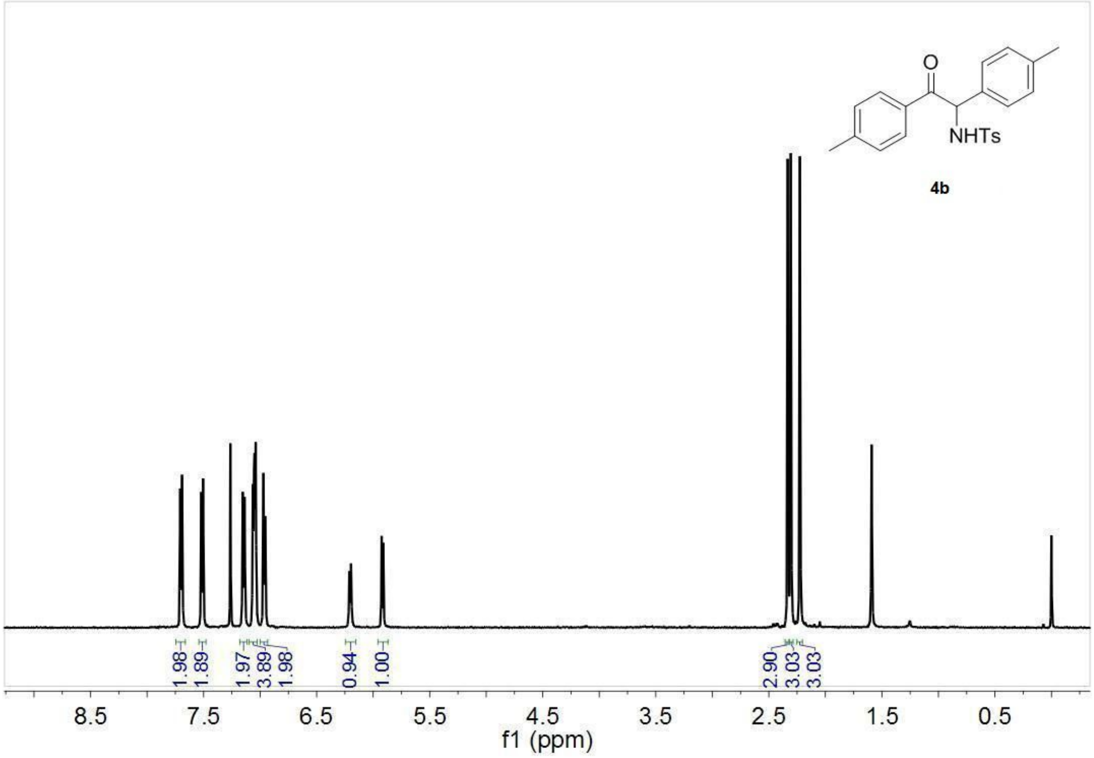

Supplementary Figure 42. ^{1}H NMR (500 MHz, CDCl$_3$) spectrum for 4b.

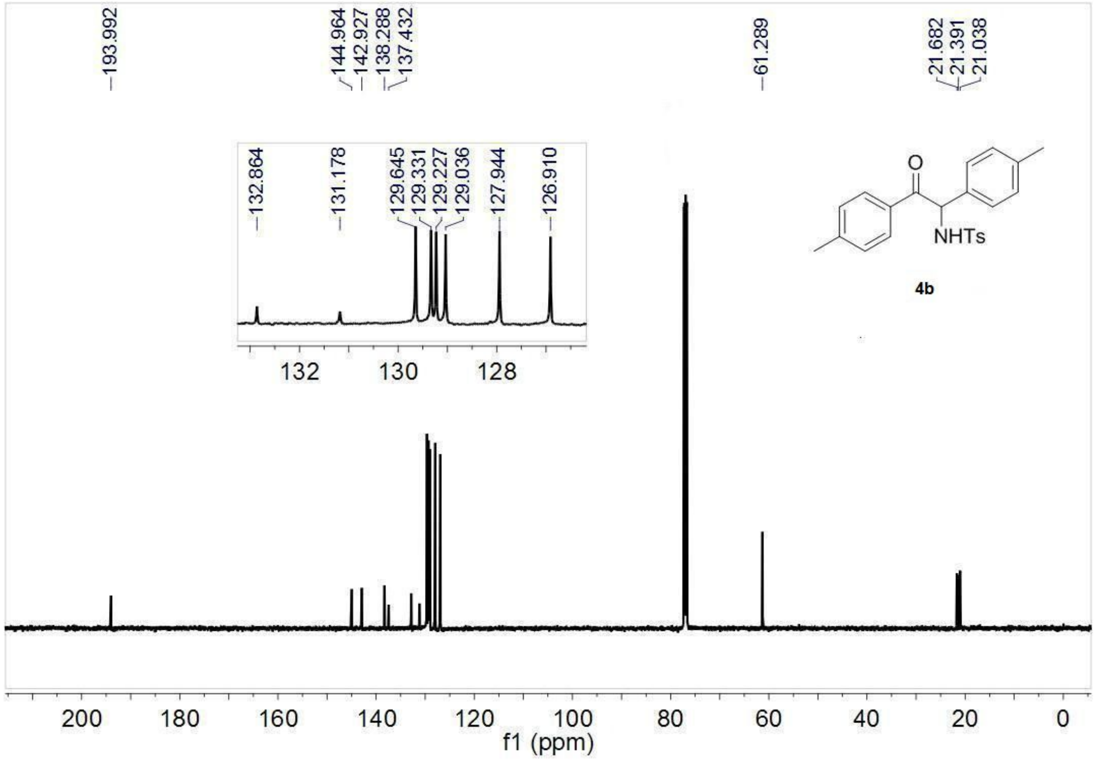

Supplementary Figure 43. ^{13}C NMR (125 MHz, $CDCl_3$) spectrum for 4b.

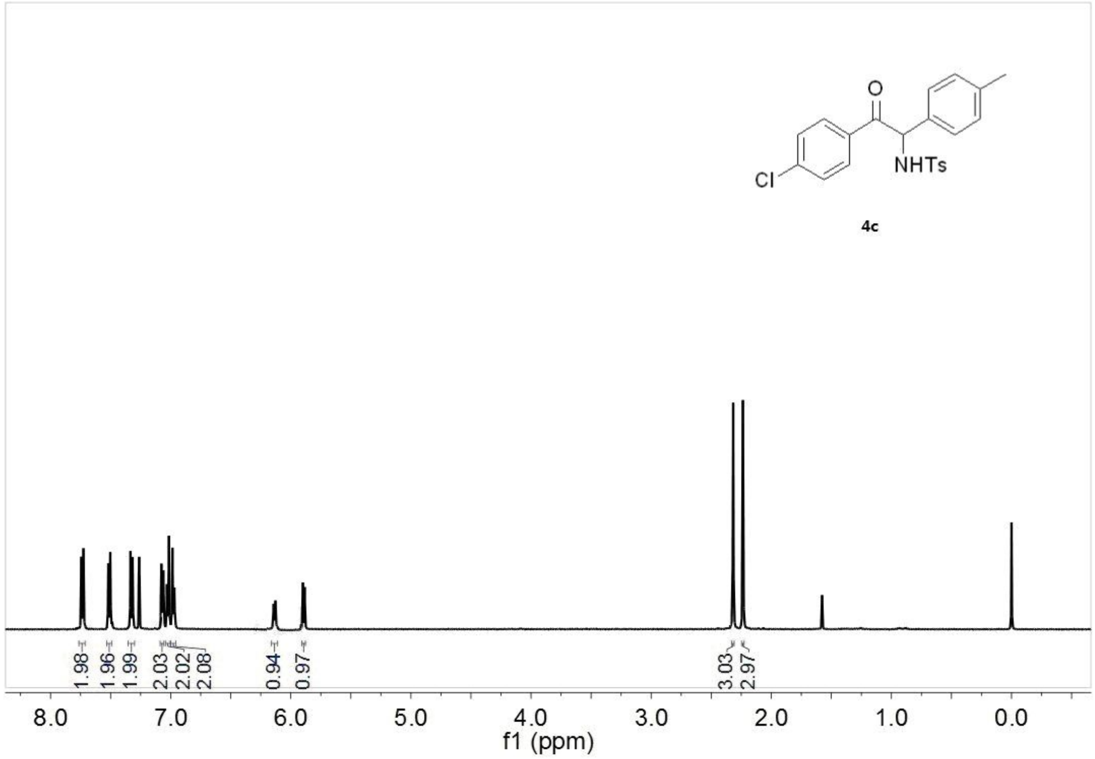

Supplementary Figure 44. ^{1}H NMR (500 MHz, $CDCl_3$) spectrum for 4c.

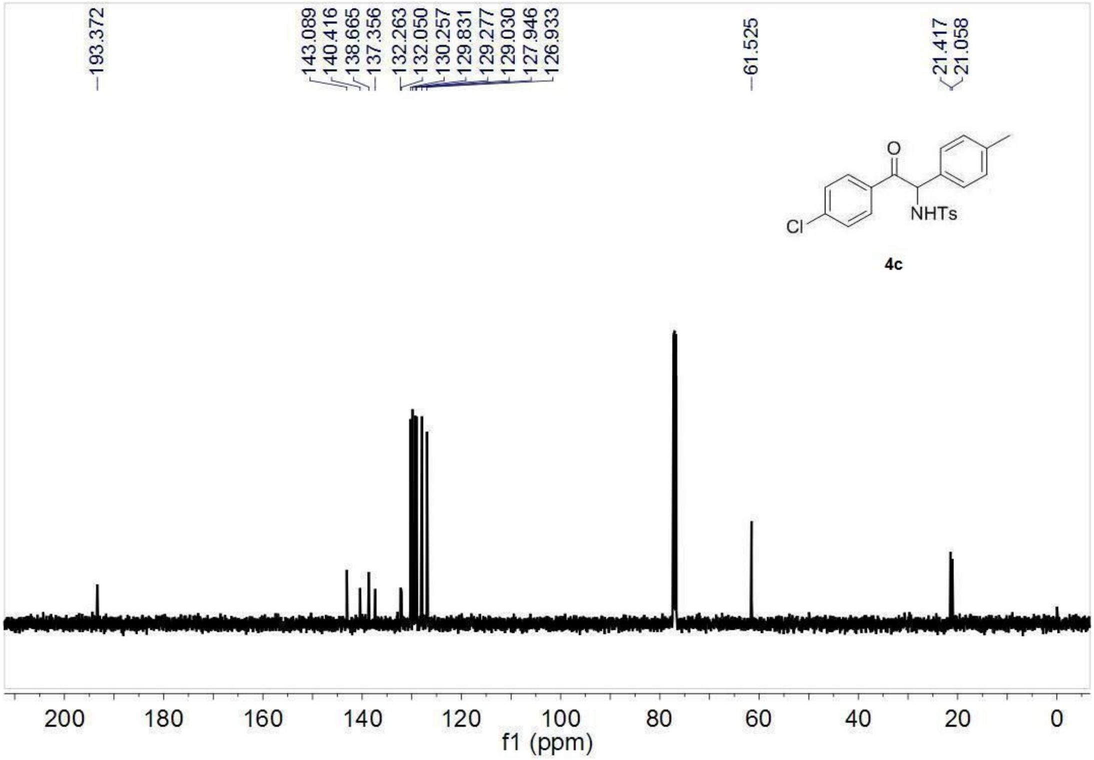

Supplementary Figure 45. ^{13}C NMR (125 MHz, $CDCl_3$) spectrum for 4c.

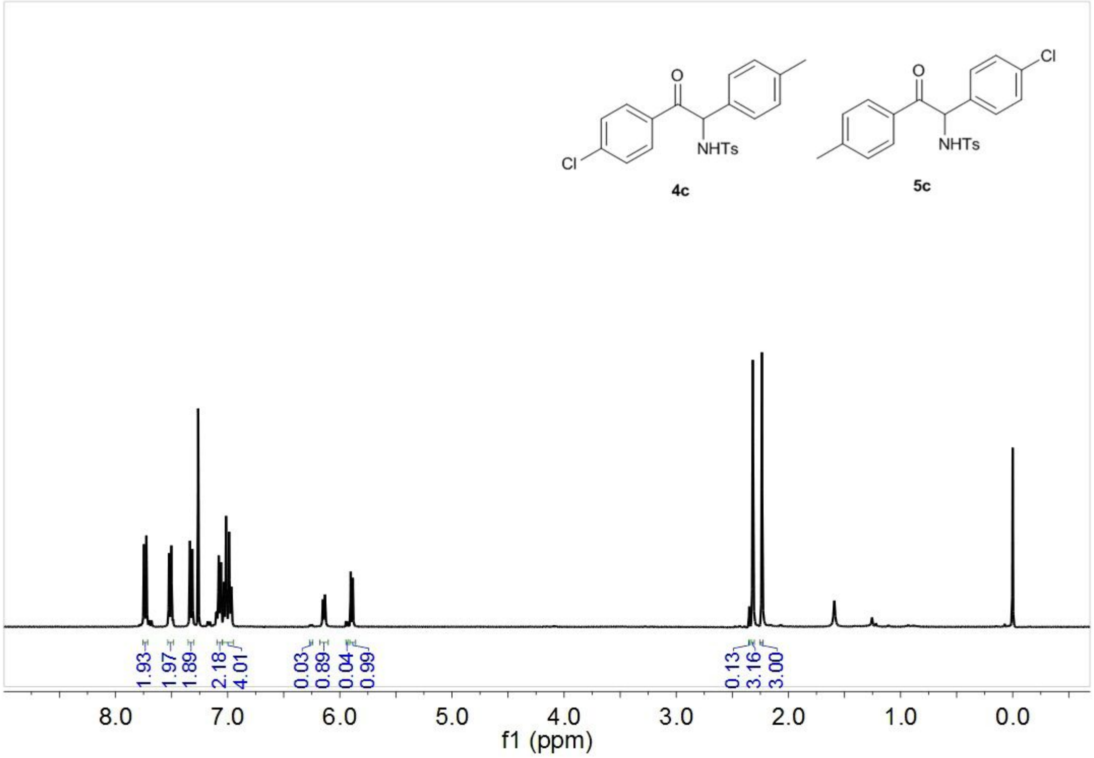

Supplementary Figure 46. ^{1}H NMR (400 MHz, $CDCl_3$) spectra for 4c and 5c.

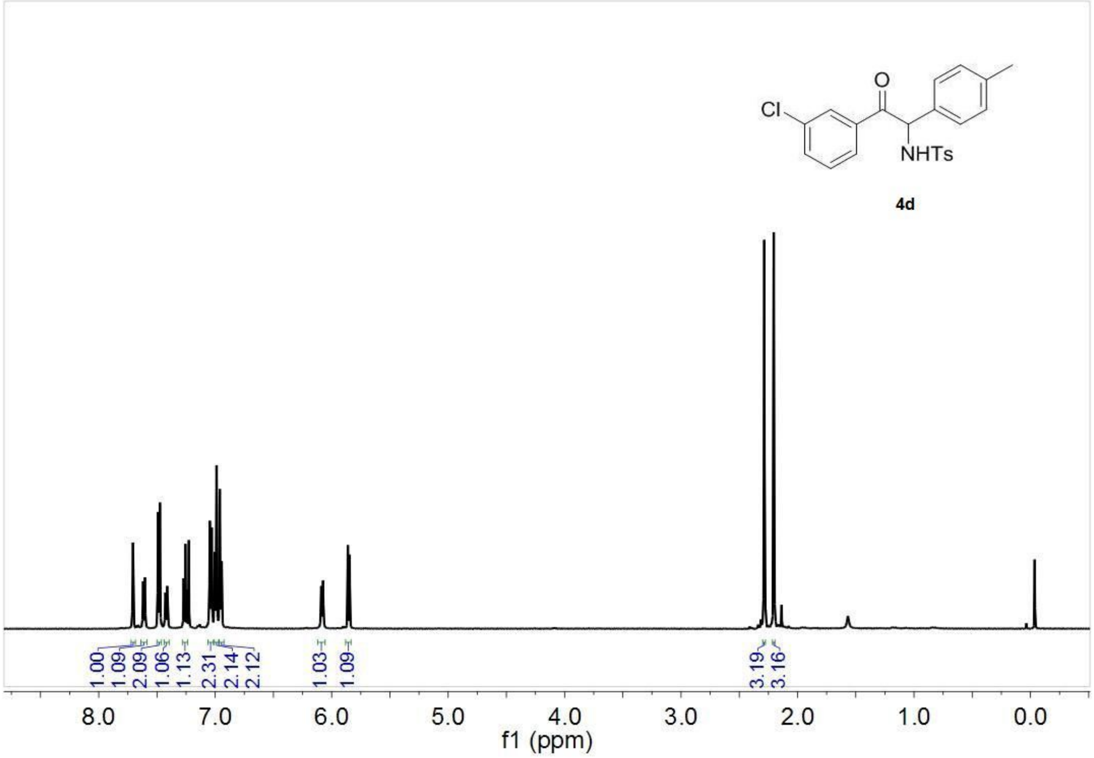

Supplementary Figure 47. ^{1}H NMR (500 MHz, $CDCl_3$) spectrum for 4d.

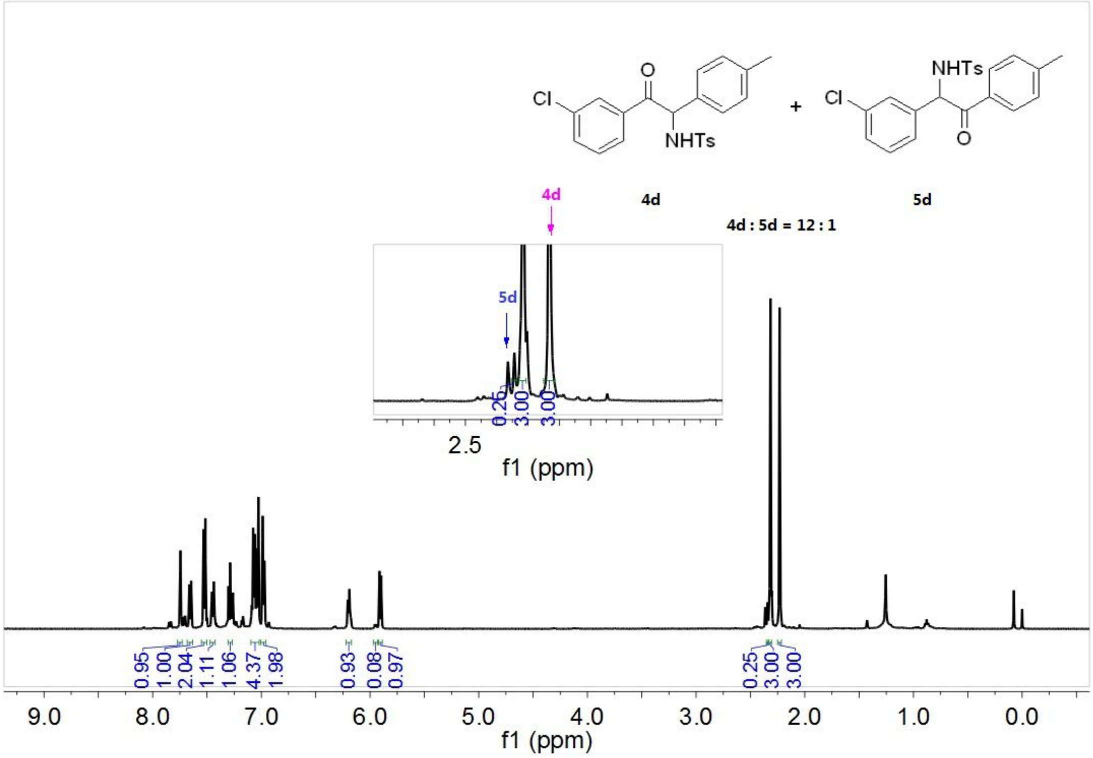

Supplementary Figure 48. ^{1}H NMR (500 MHz, $CDCl_3$) spectra for 4d and 5d.

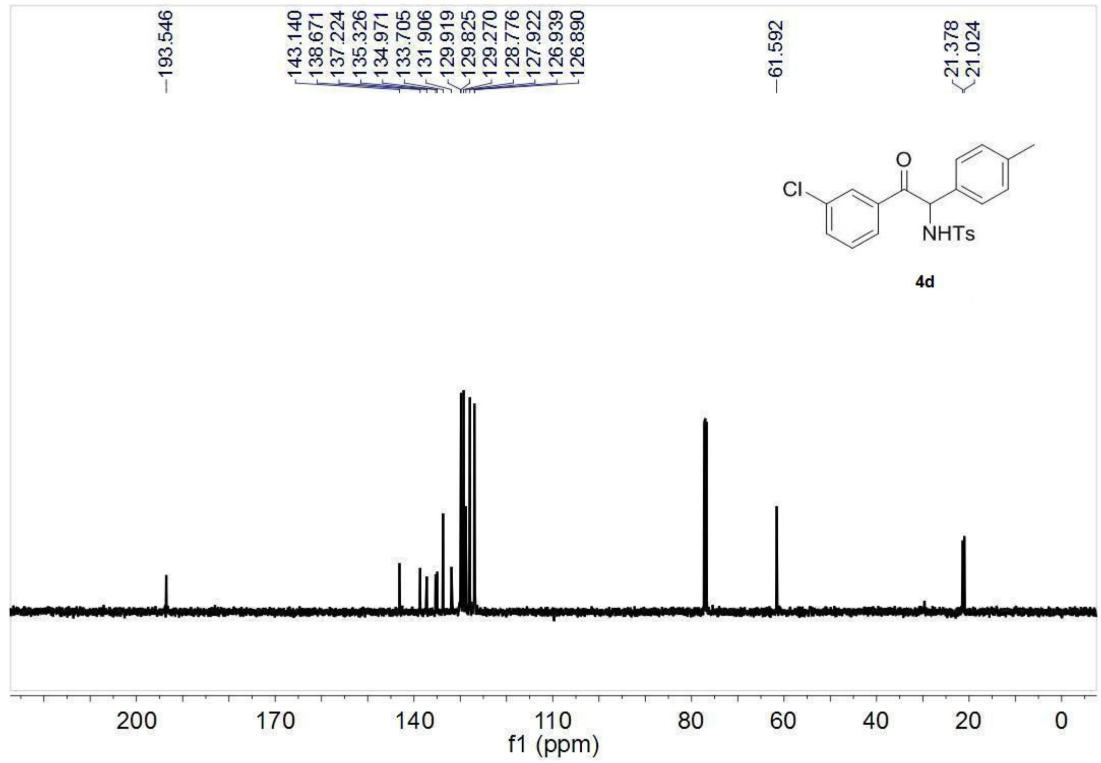

Supplementary Figure 49. ^{13}**C NMR (125 MHz, CDCl$_3$) spectrum for 4d.**

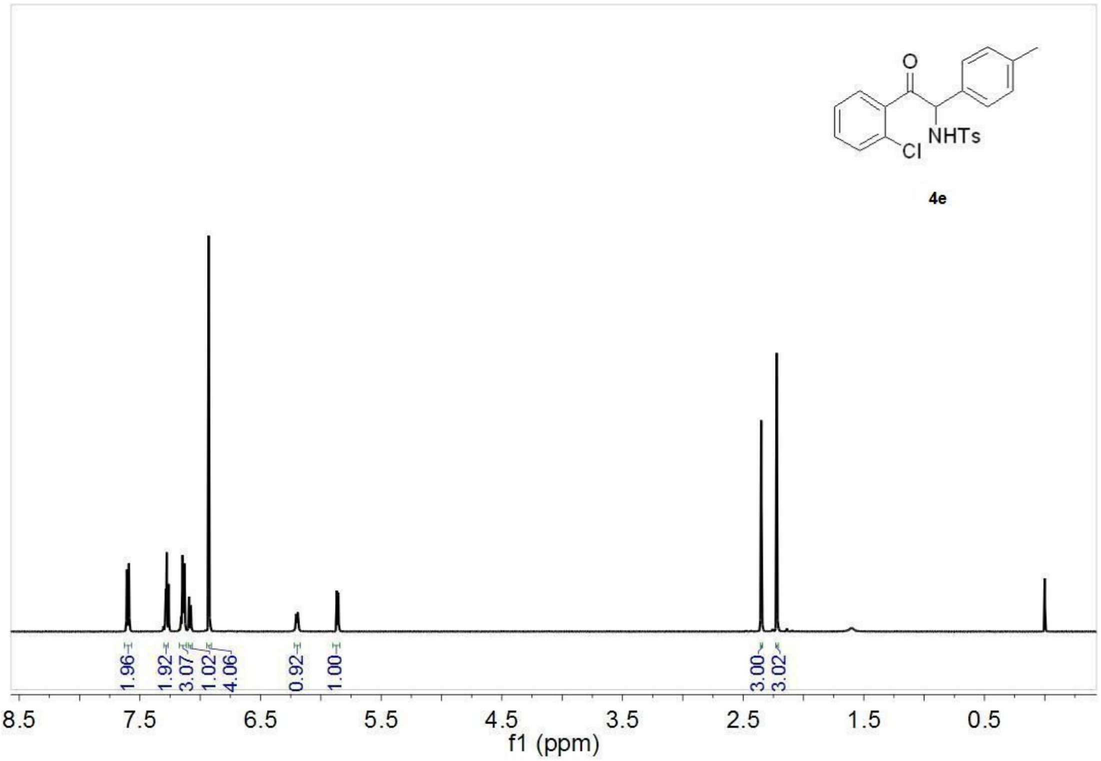

Supplementary Figure 50. ^{1}**H NMR (500 MHz, CDCl$_3$) spectrum for 4e.**

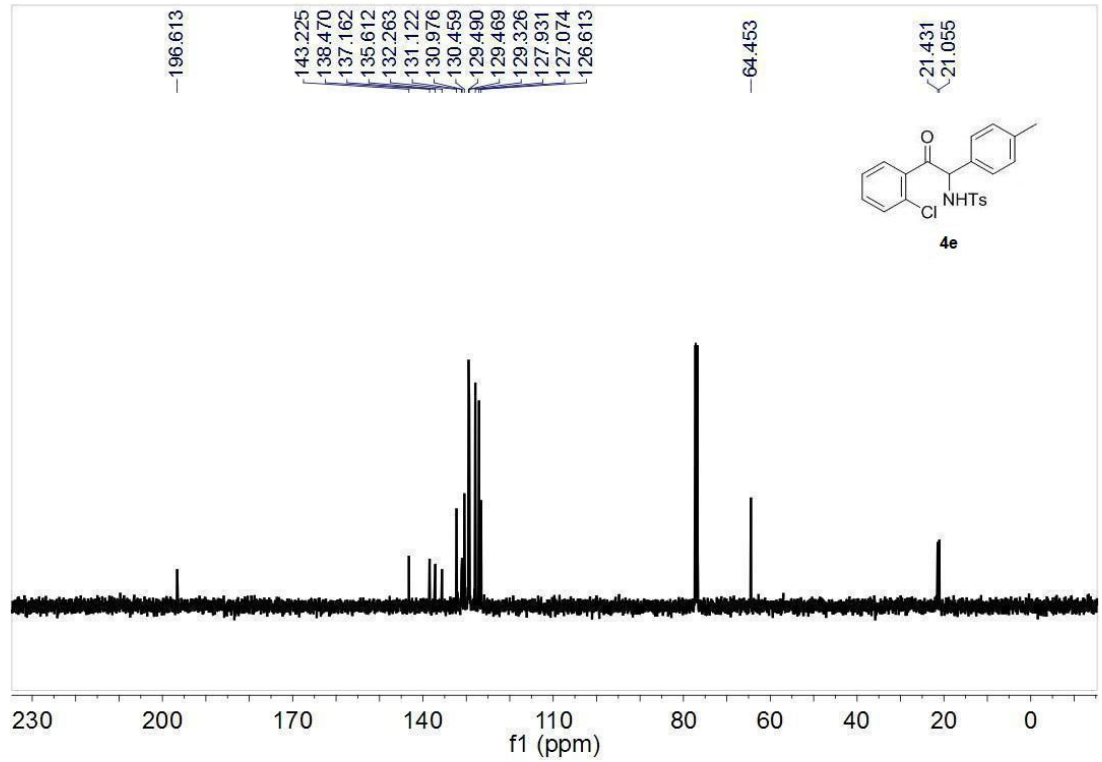

Supplementary Figure 51. ^{13}**C NMR (125 MHz, CDCl**$_3$**) spectrum for 4e.**

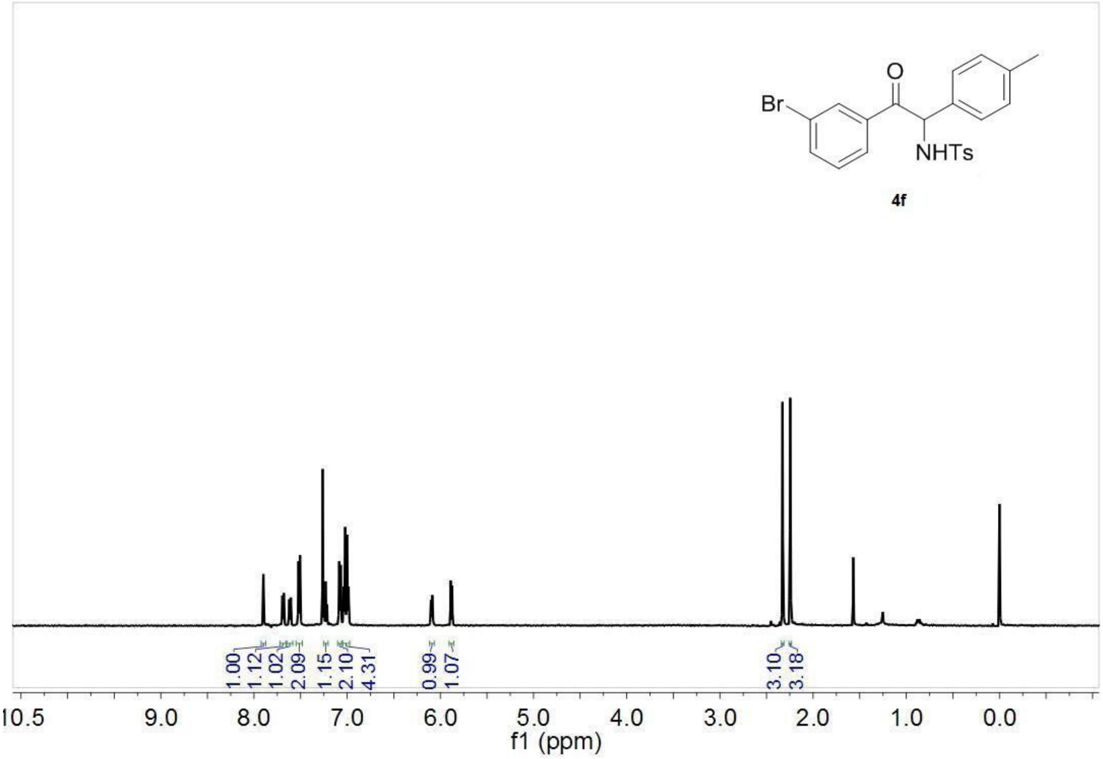

Supplementary Figure 52. ^{1}**H NMR (500 MHz, CDCl**$_3$**) spectrum for 4f.**

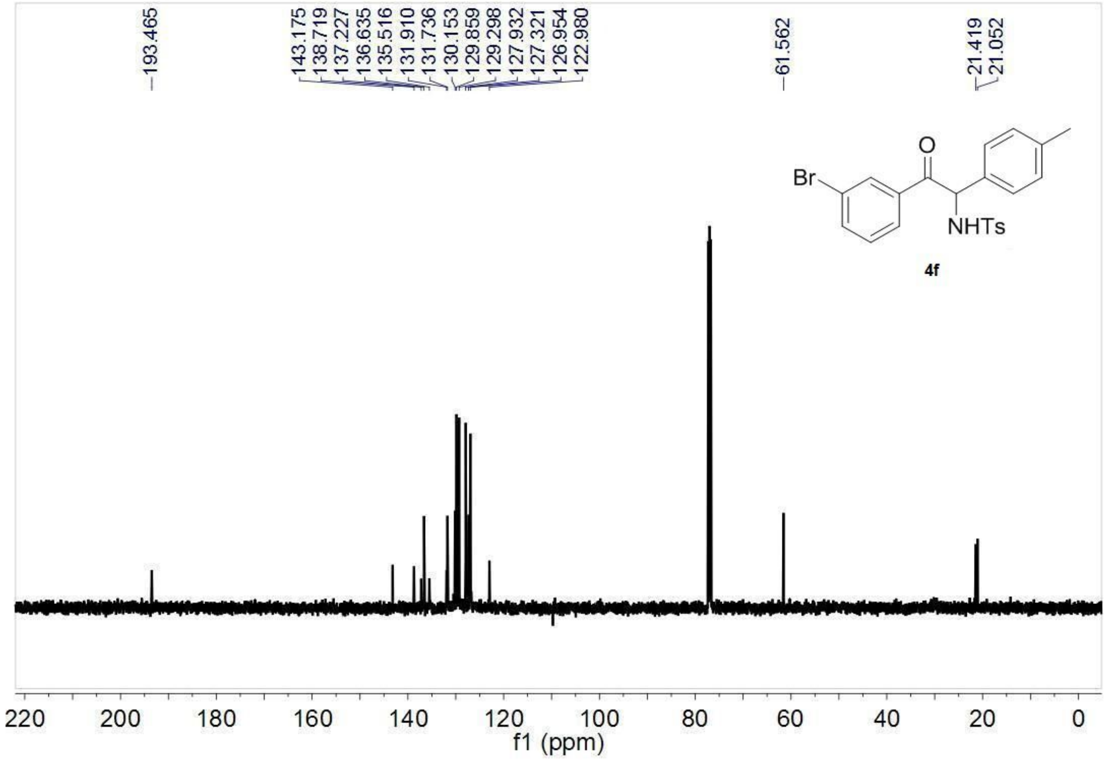

Supplementary Figure 53. **^{13}C NMR (125 MHz, $CDCl_3$) spectrum for 4f.**

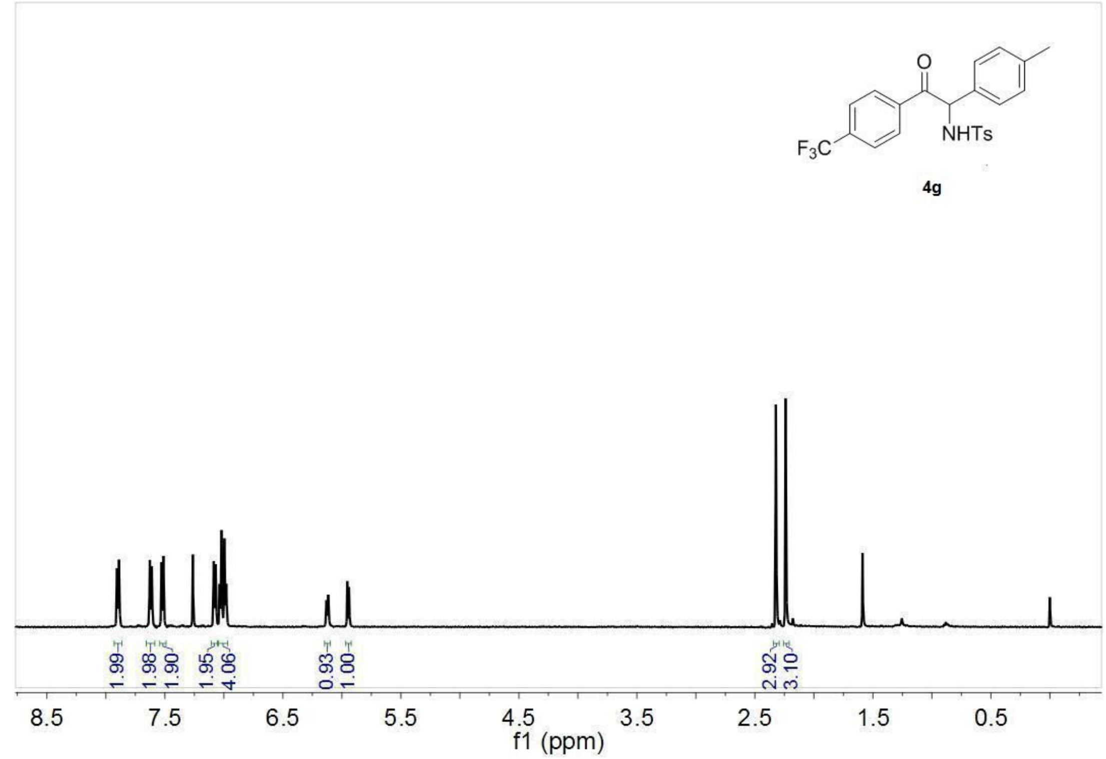

Supplementary Figure 54. **^{1}H NMR (500 MHz, $CDCl_3$) spectrum for 4g.**

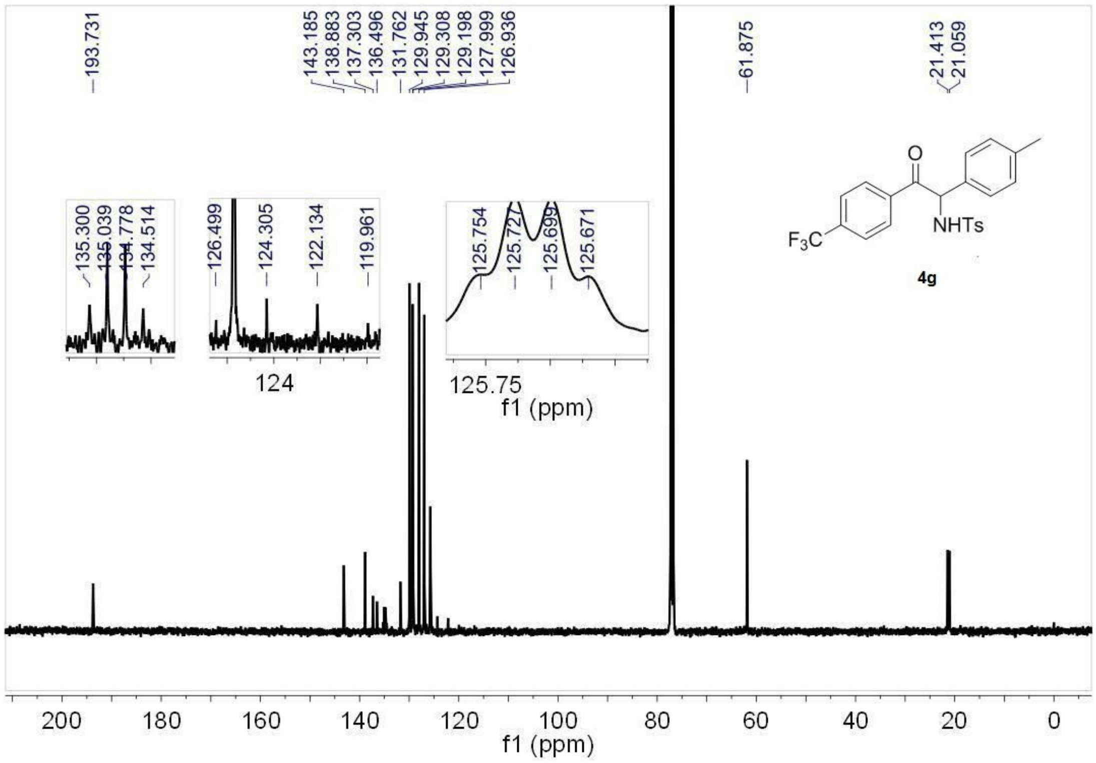

Supplementary Figure 55. ^{13}C NMR (125 MHz, $CDCl_3$) spectrum for 4g.

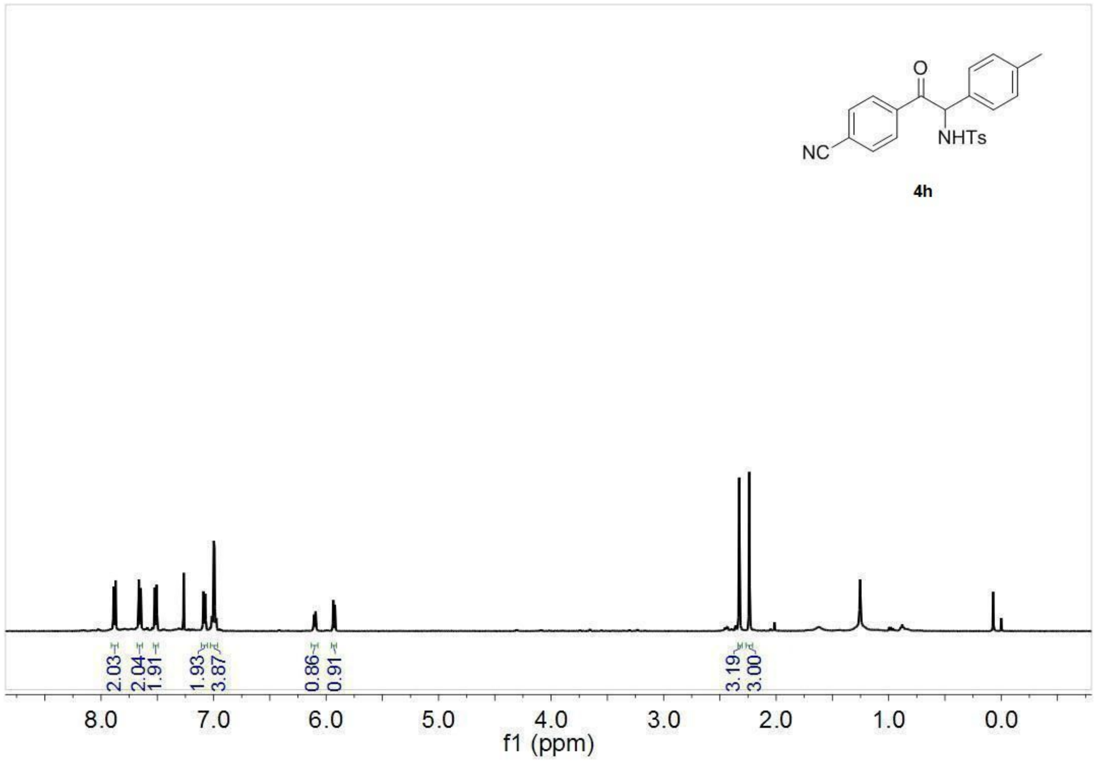

Supplementary Figure 56. ^{1}H NMR (500 MHz, $CDCl_3$) spectrum for 4h.

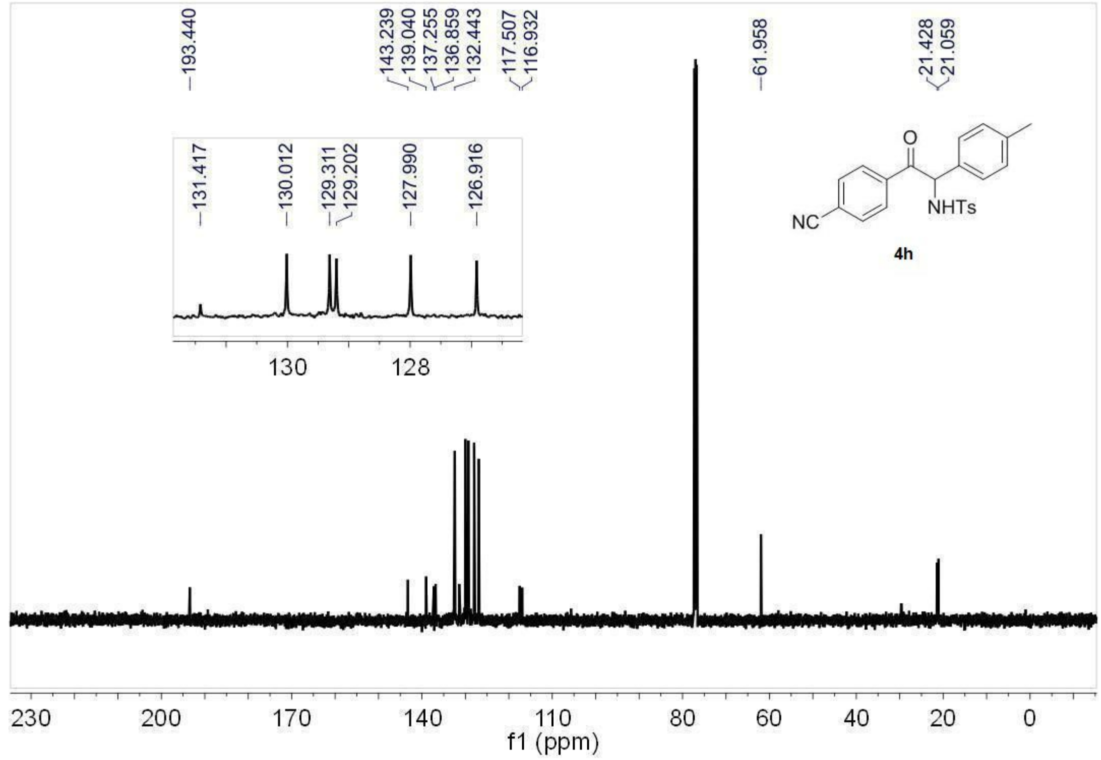

Supplementary Figure 57. ^{13}C NMR (125 MHz, $CDCl_3$) spectrum for 4h.

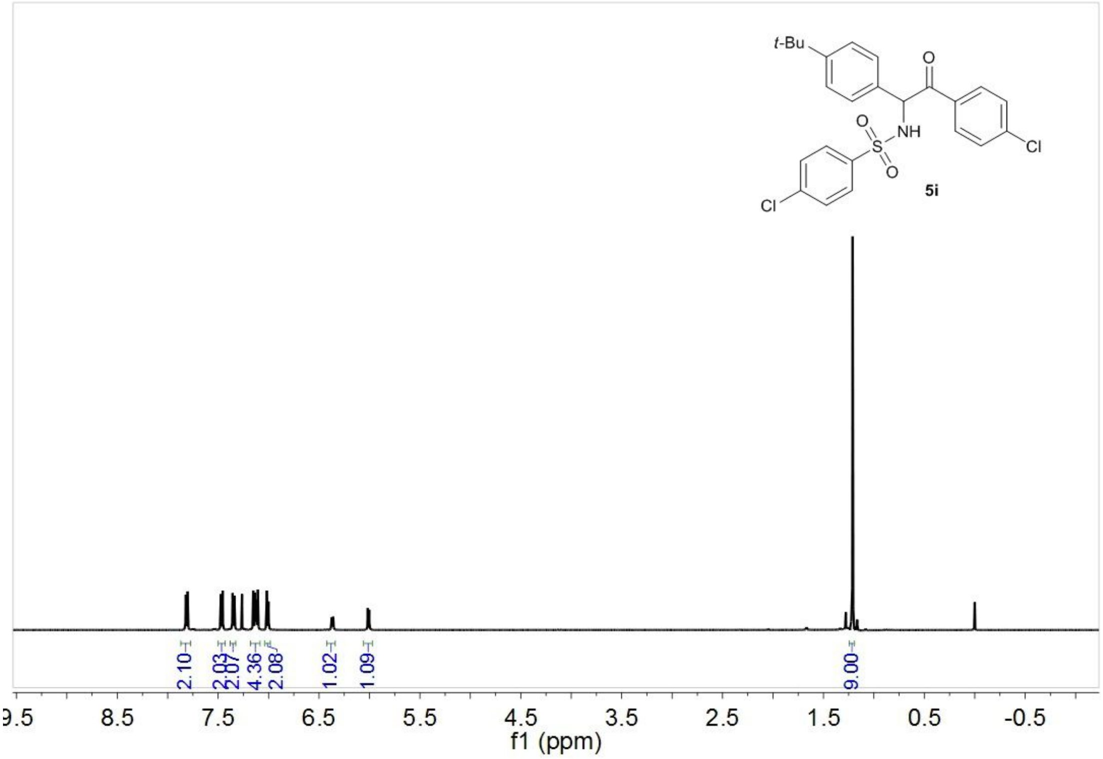

Supplementary Figure 58. ^{1}H NMR (500 MHz, $CDCl_3$) spectrum for 5i.

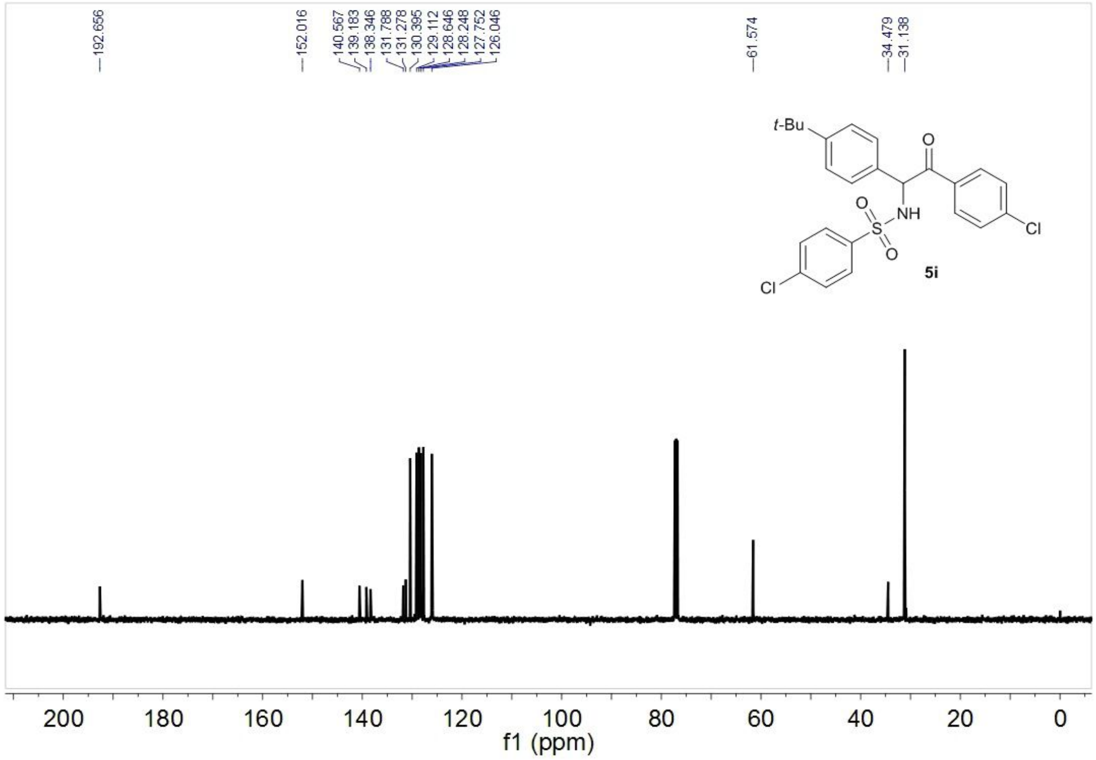

Supplementary Figure 59. ^{13}C NMR (125 MHz, $CDCl_3$) spectrum for 5i.

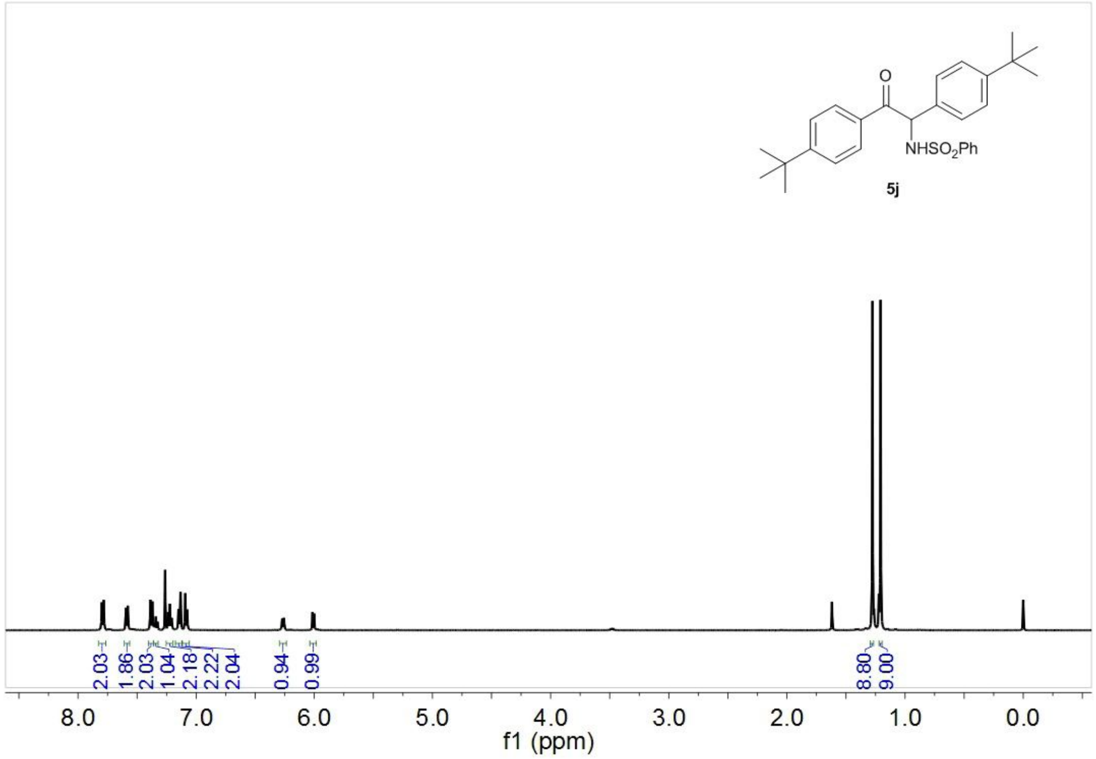

Supplementary Figure 60. ^{1}H NMR (500 MHz, $CDCl_3$) spectrum for 5j.

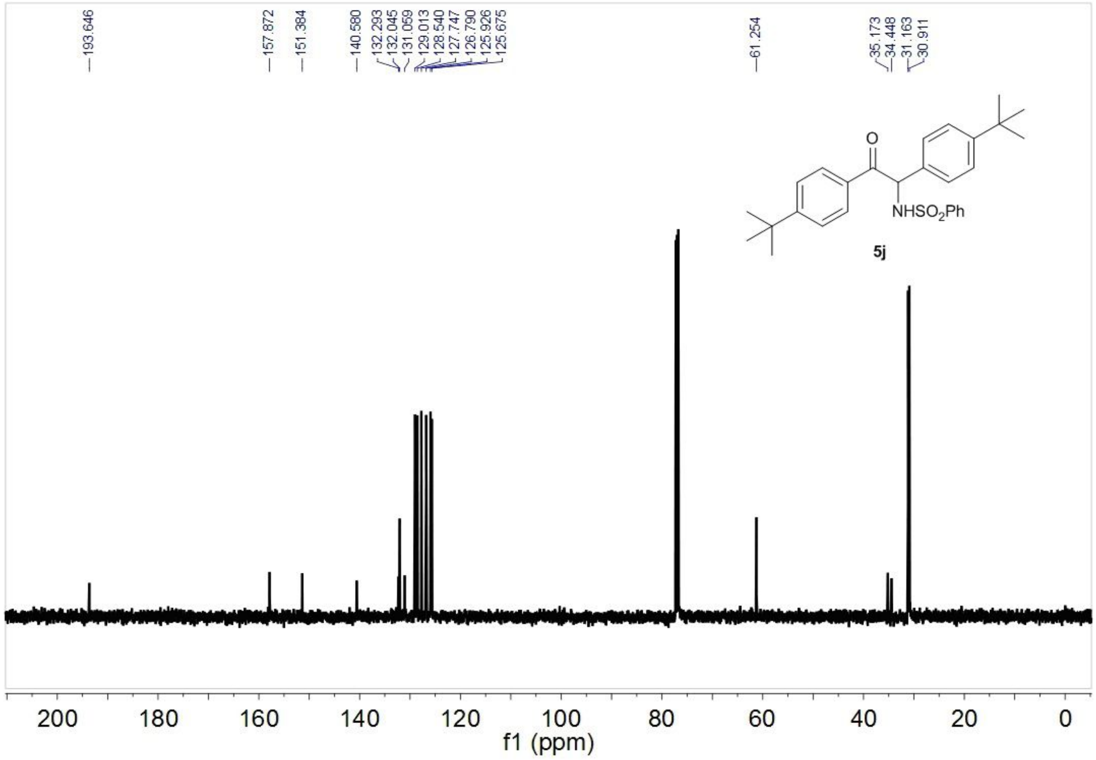

Supplementary Figure 61. ^{13}C NMR (125 MHz, $CDCl_3$) spectrum for 5j.

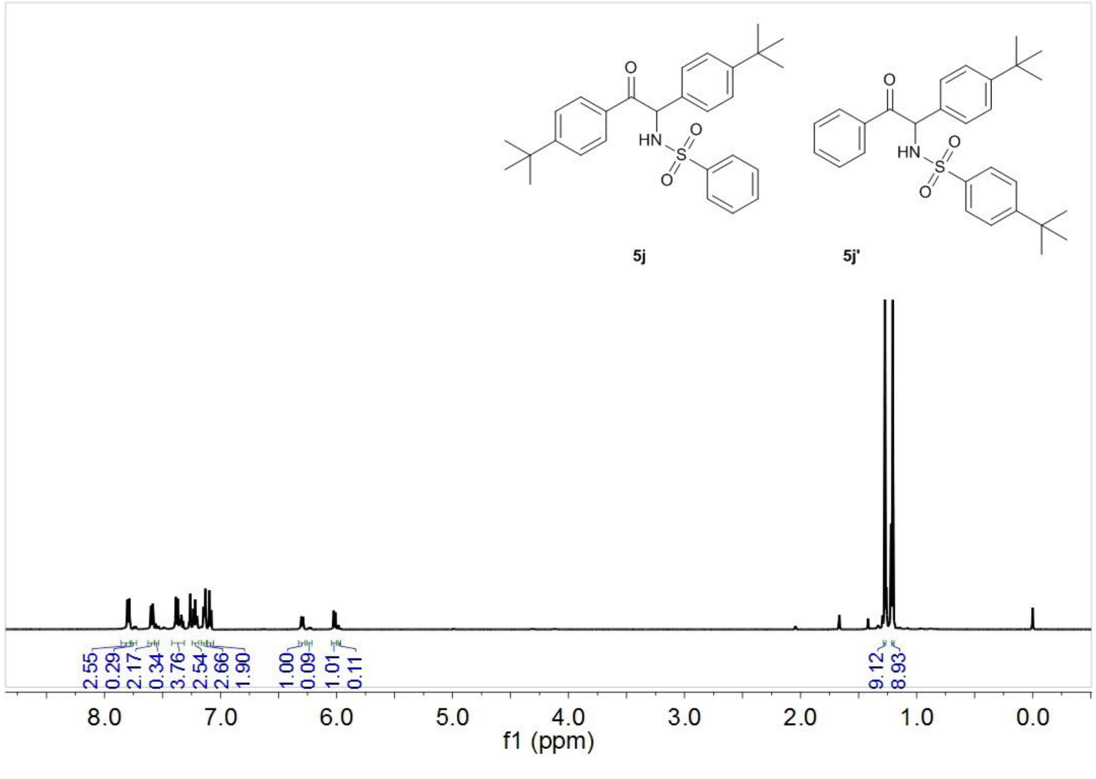

Supplementary Figure 62. ^{1}H NMR (500 MHz, $CDCl_3$) spectra for 5j and 5j'.

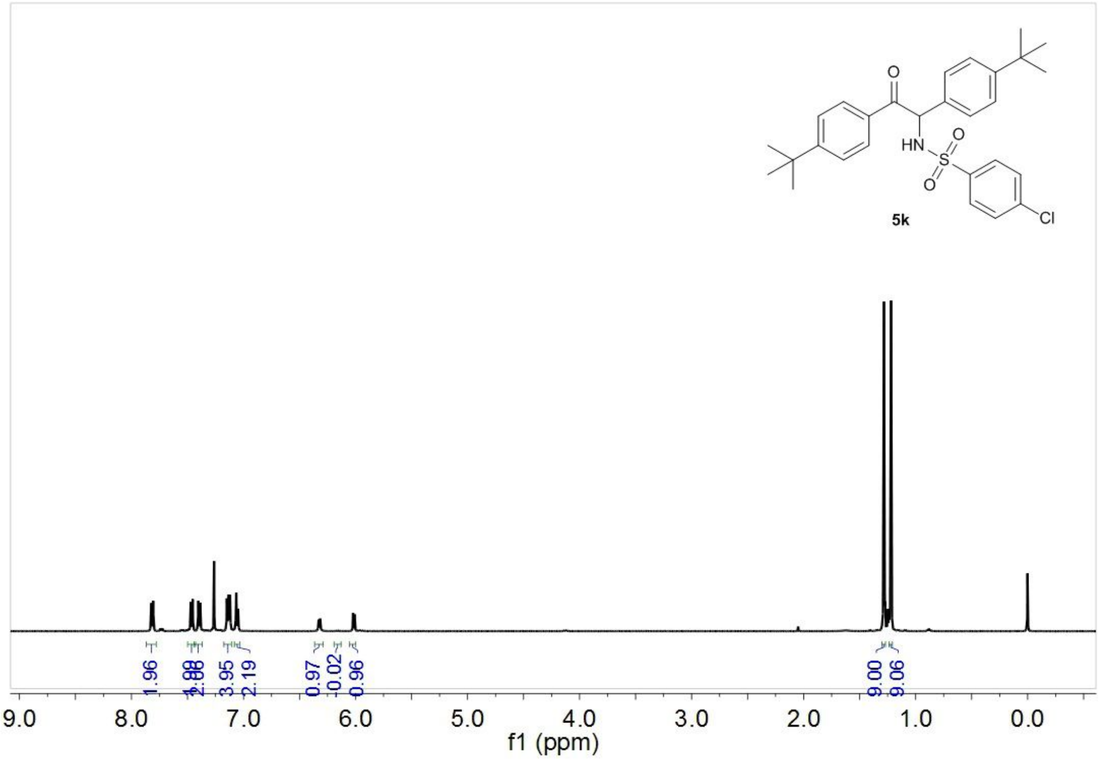

Supplementary Figure 63. ^{1}H NMR (500 MHz, $CDCl_3$) spectra for 5k and 5k'.

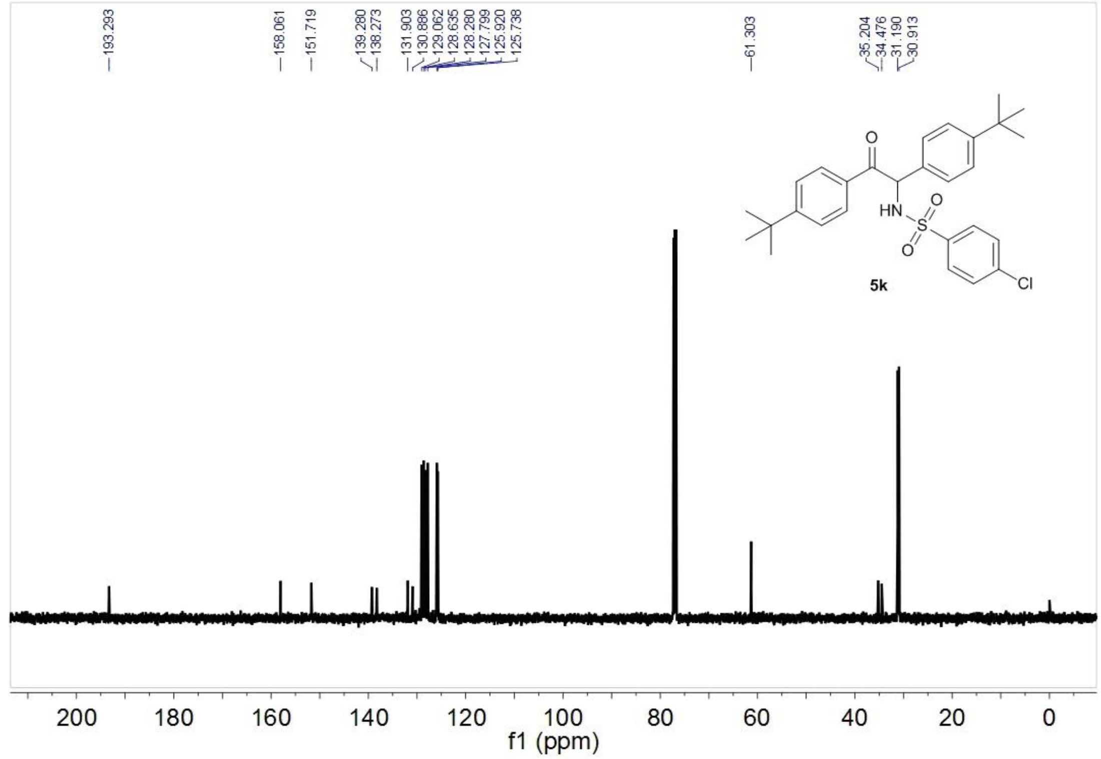

Supplementary Figure 64. ^{13}C NMR (125 MHz, $CDCl_3$) spectrum for 5k.

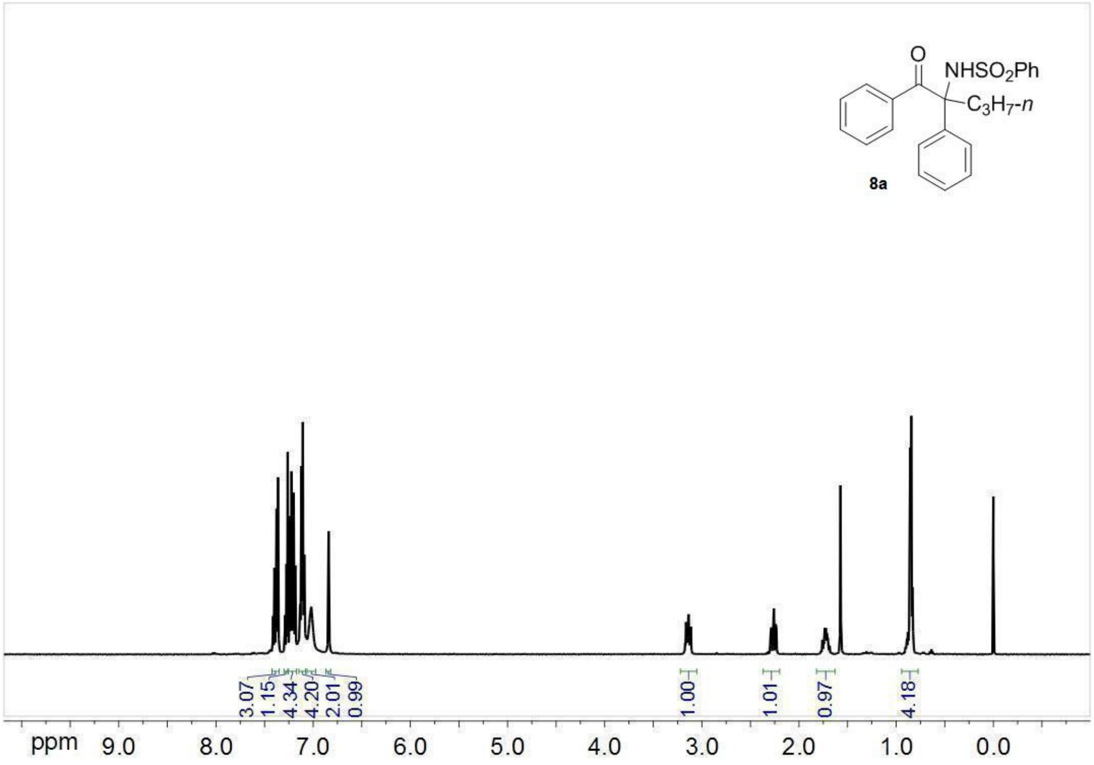

Supplementary Figure 65. ^{1}H NMR (500 MHz, $CDCl_3$) spectrum for 8a.

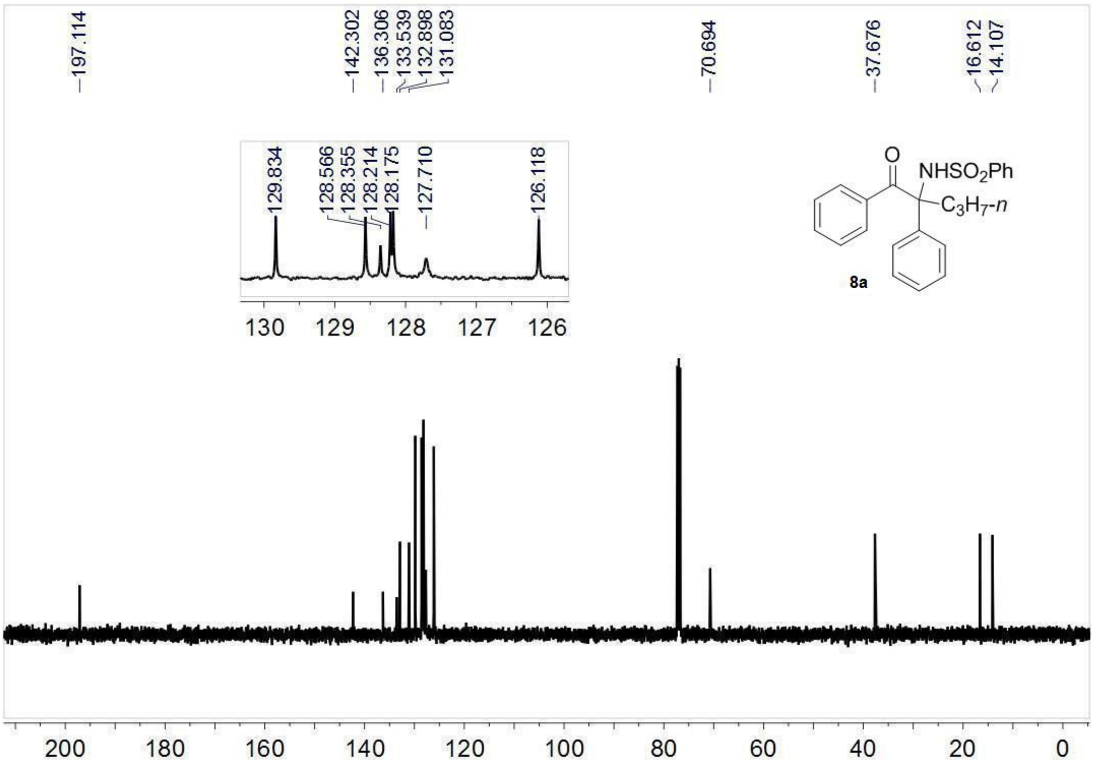

Supplementary Figure 66. ^{13}C NMR (125 MHz, $CDCl_3$) spectrum for 8a.

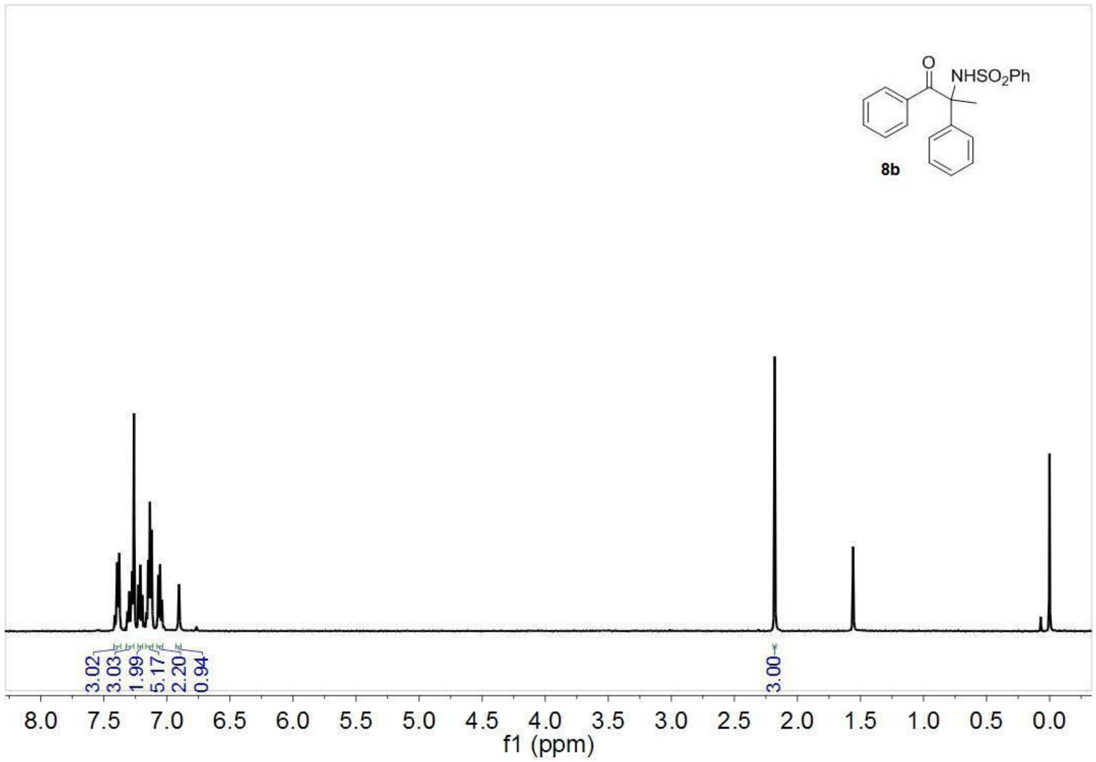

Supplementary Figure 67. ^{1}H NMR (500 MHz, $CDCl_3$) spectrum for 8b.

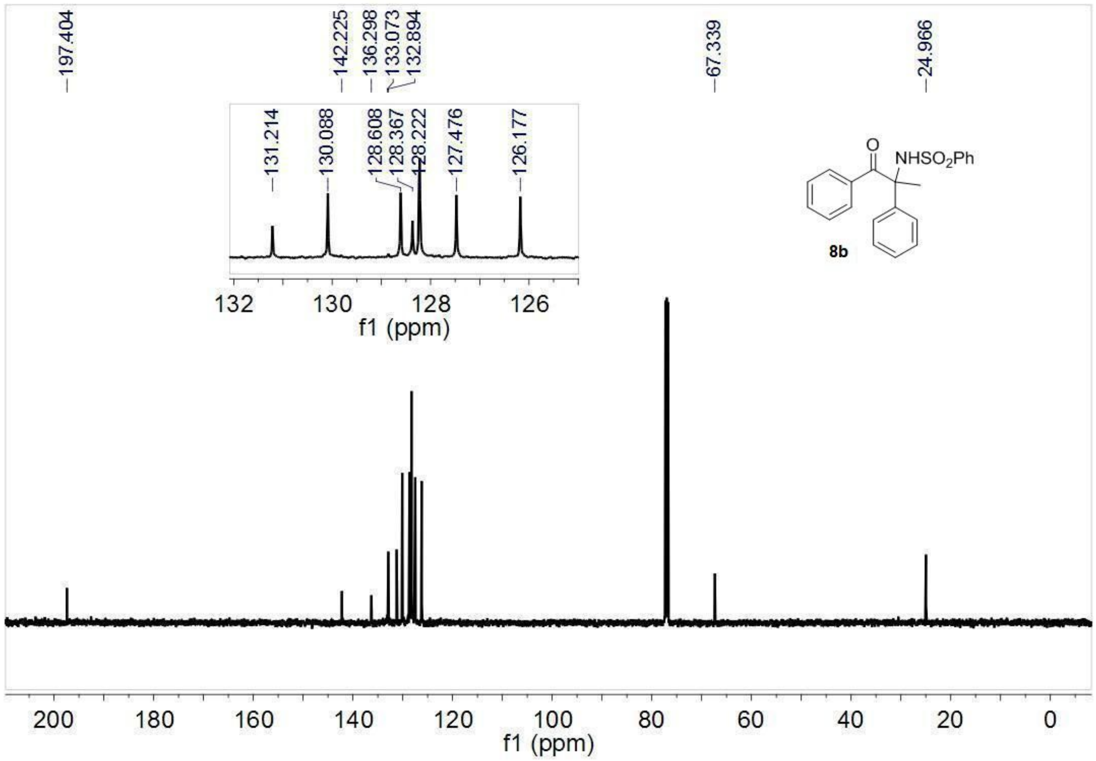

Supplementary Figure 68. ^{13}C NMR (125 MHz, $CDCl_3$) spectrum for 8b.

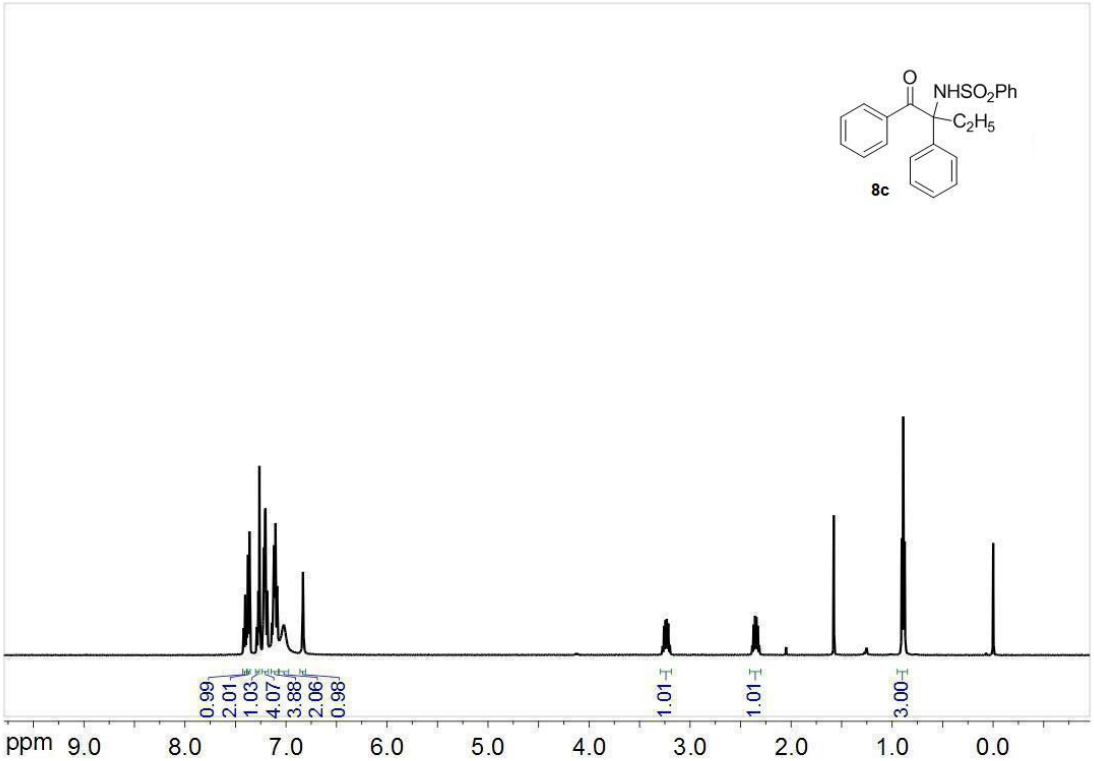

Supplementary Figure 69. ^{1}H NMR (500 MHz, $CDCl_3$) spectrum for 8c.

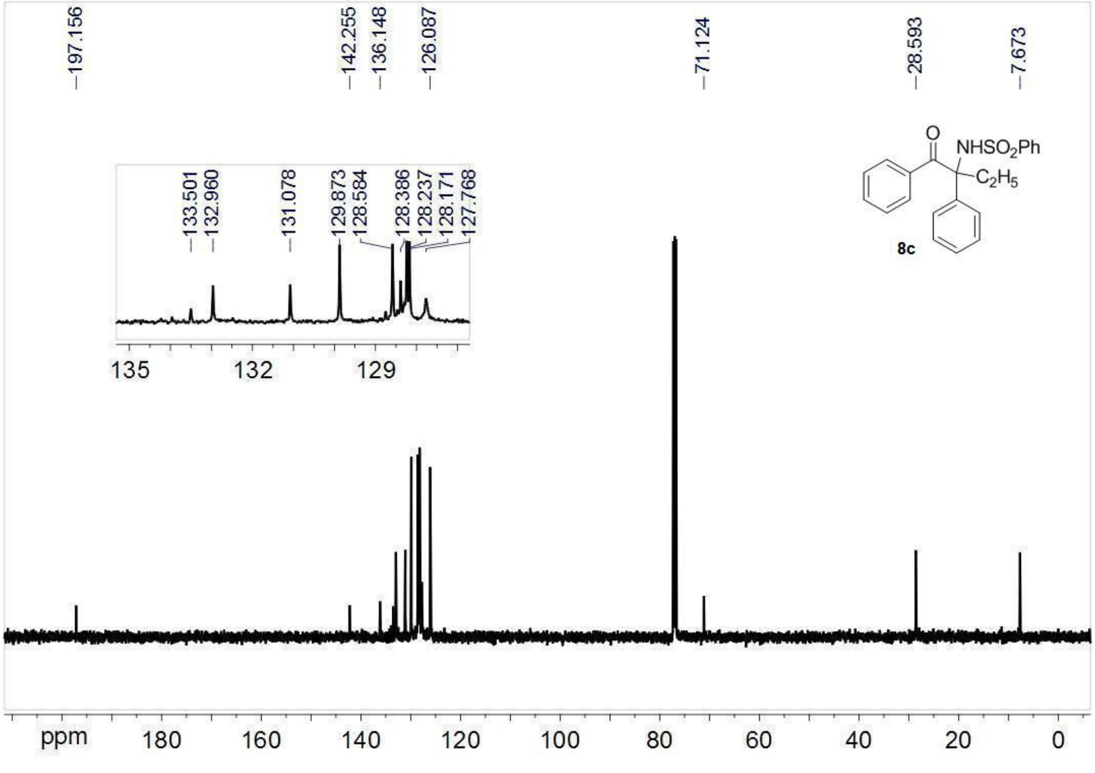

Supplementary Figure 70. ^{13}C NMR (125 MHz, $CDCl_3$) spectrum for 8c.

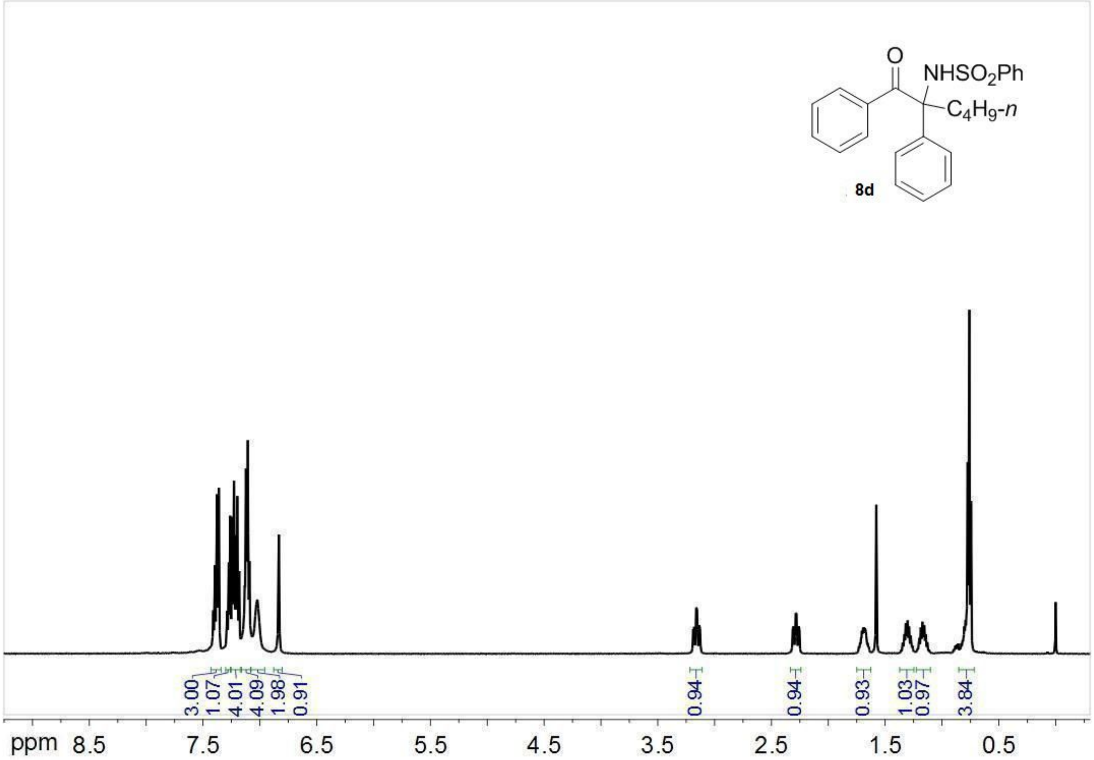

Supplementary Figure 71. ^{1}H NMR (500 MHz, $CDCl_3$) spectrum for 8d.

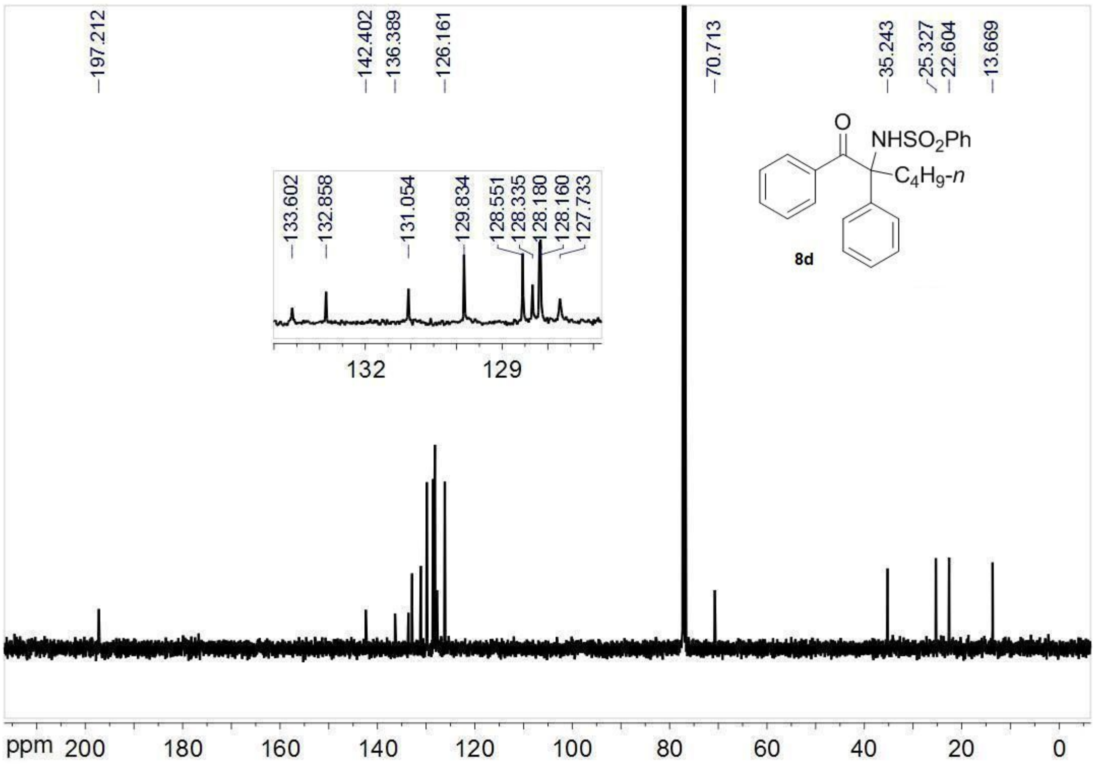

Supplementary Figure 72. ^{13}C NMR (125 MHz, $CDCl_3$) spectrum for 8d.

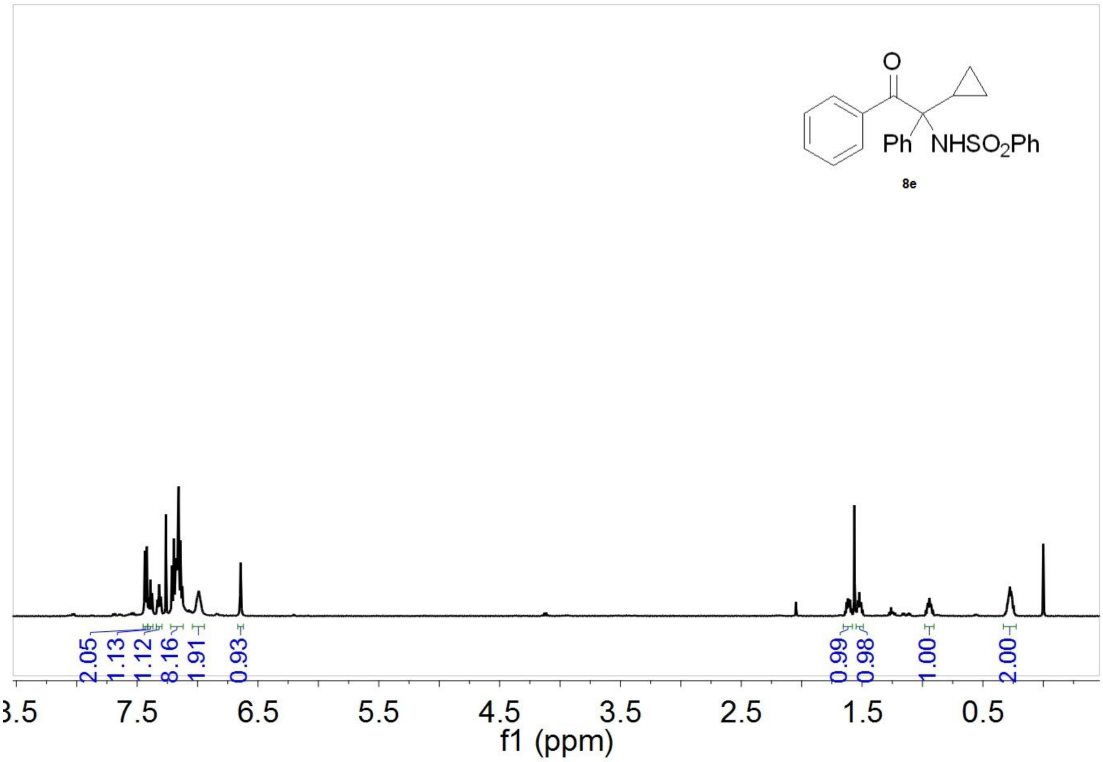

Supplementary Figure 73. ^{1}H NMR (500 MHz, $CDCl_3$) spectrum for 8e.

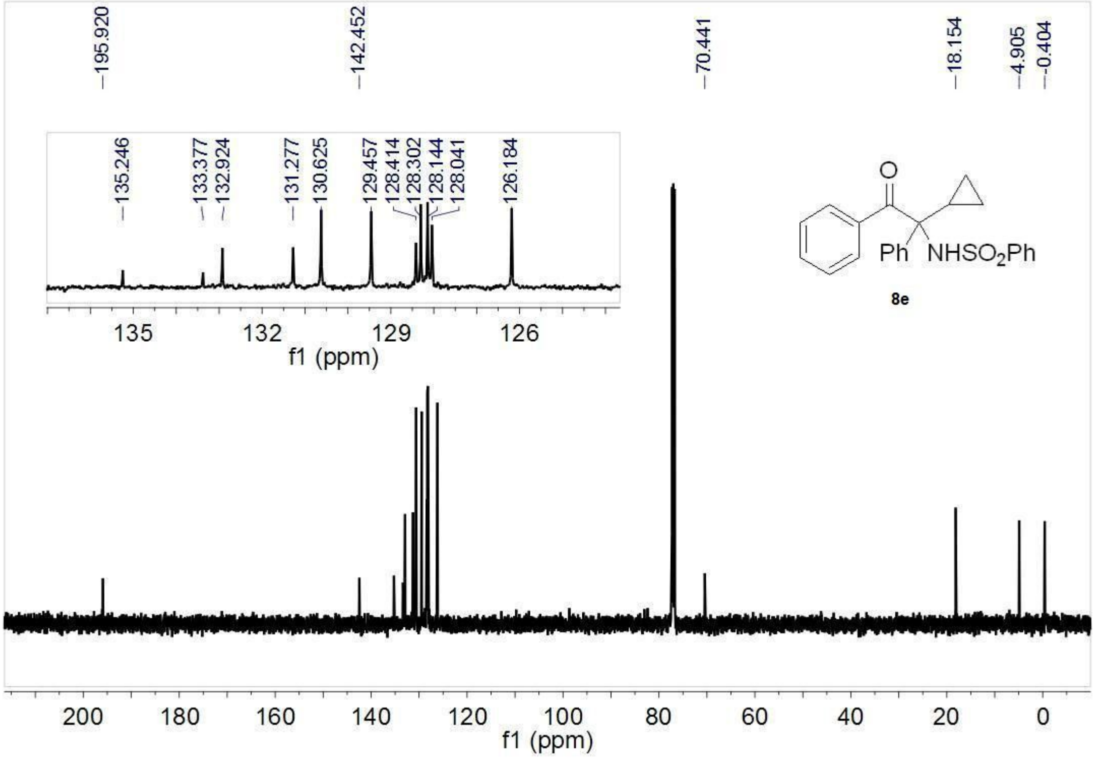

Supplementary Figure 74. ^{13}C NMR (125 MHz, $CDCl_3$) spectrum for 8e.

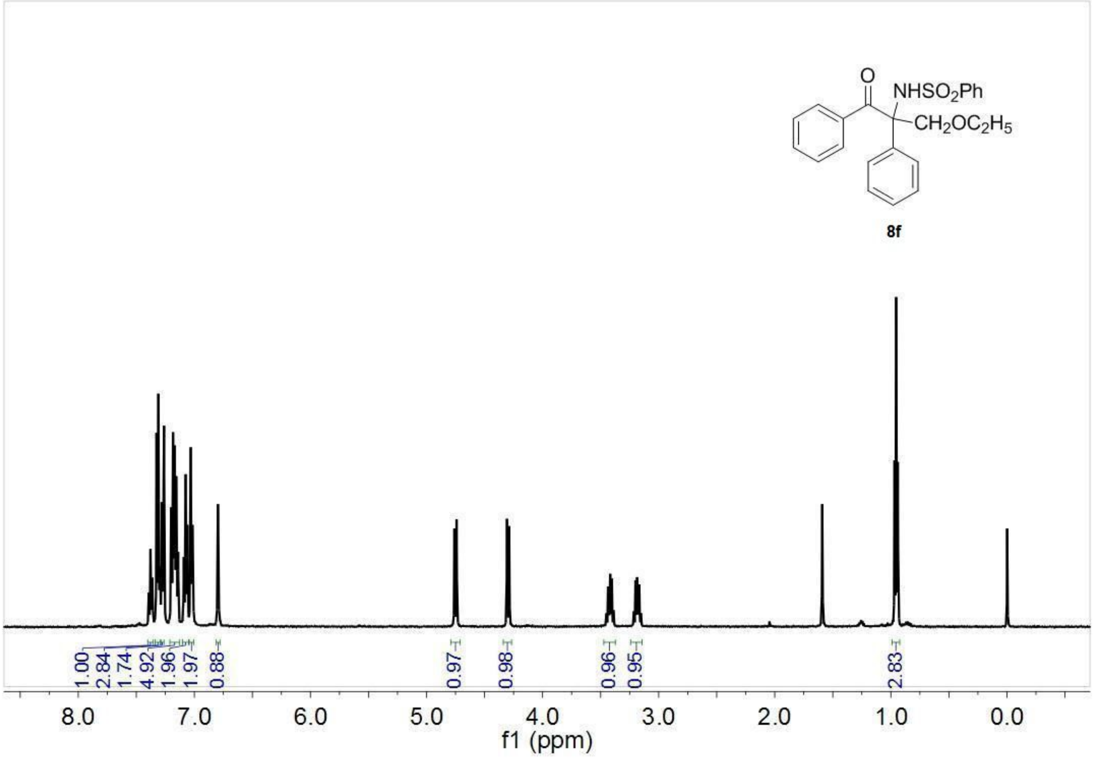

Supplementary Figure 75. ^{1}H NMR (500 MHz, $CDCl_3$) spectrum for 8f.

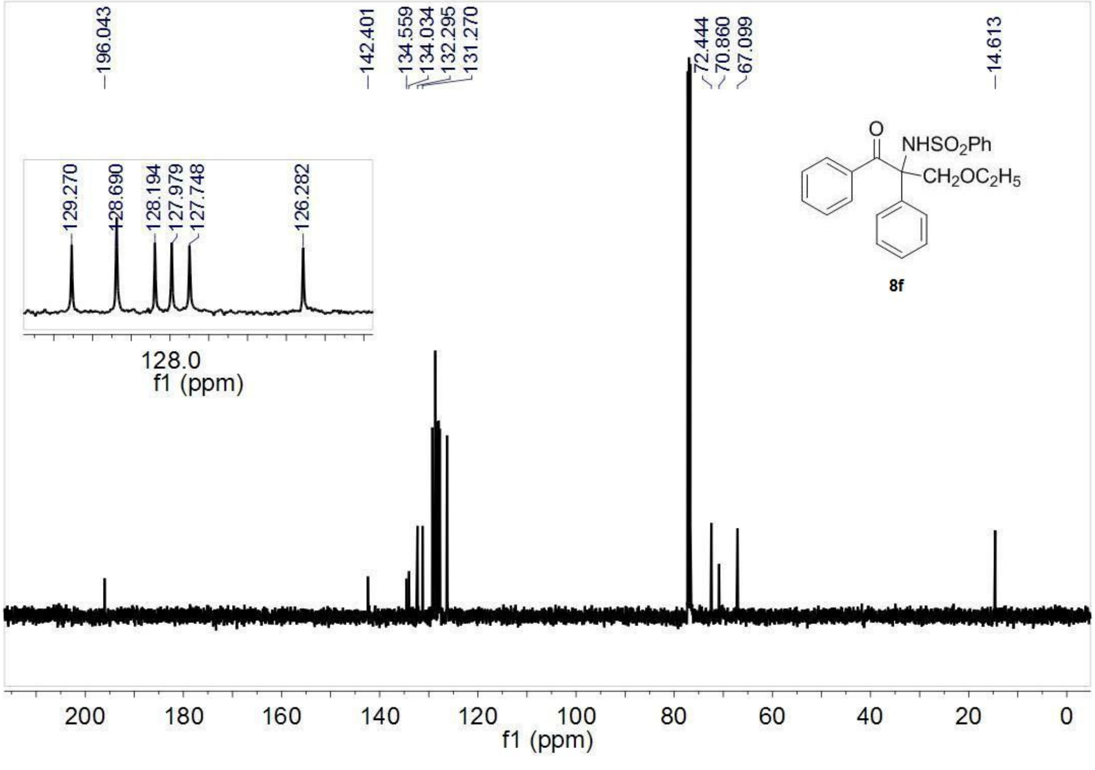

Supplementary Figure 76. ^{13}C NMR (125 MHz, $CDCl_3$) spectrum for 8f.

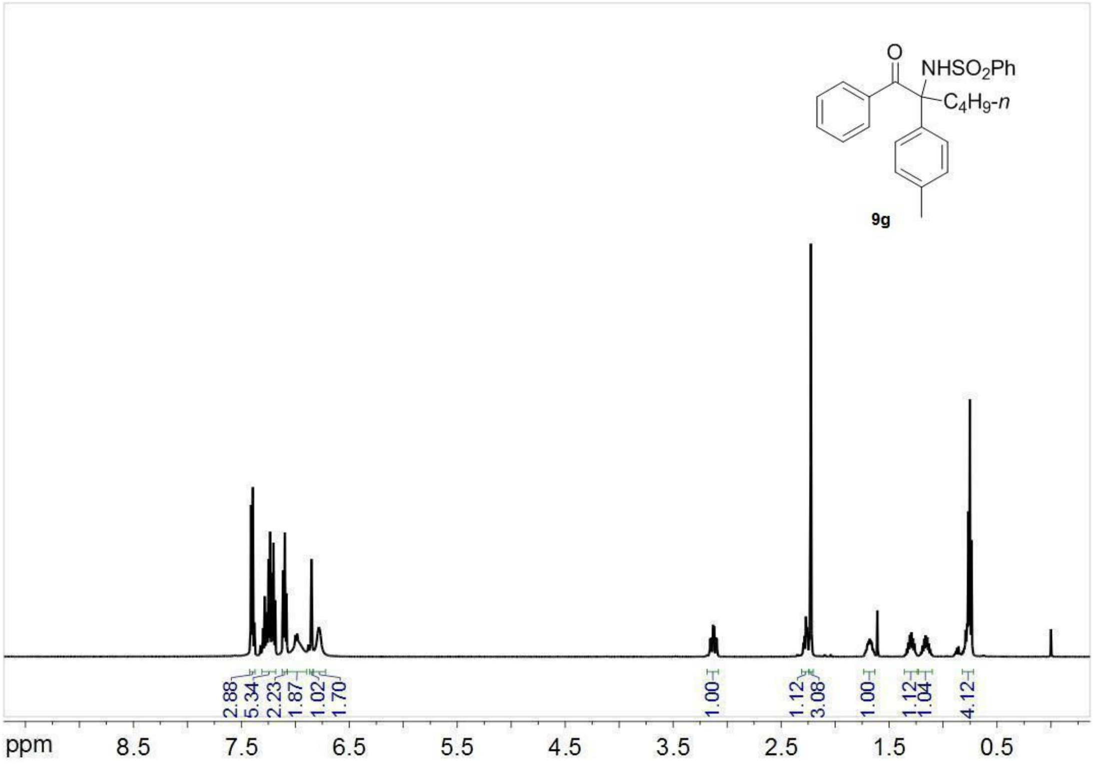

Supplementary Figure 77. ^{1}H NMR (500 MHz, $CDCl_3$) spectrum for 9g.

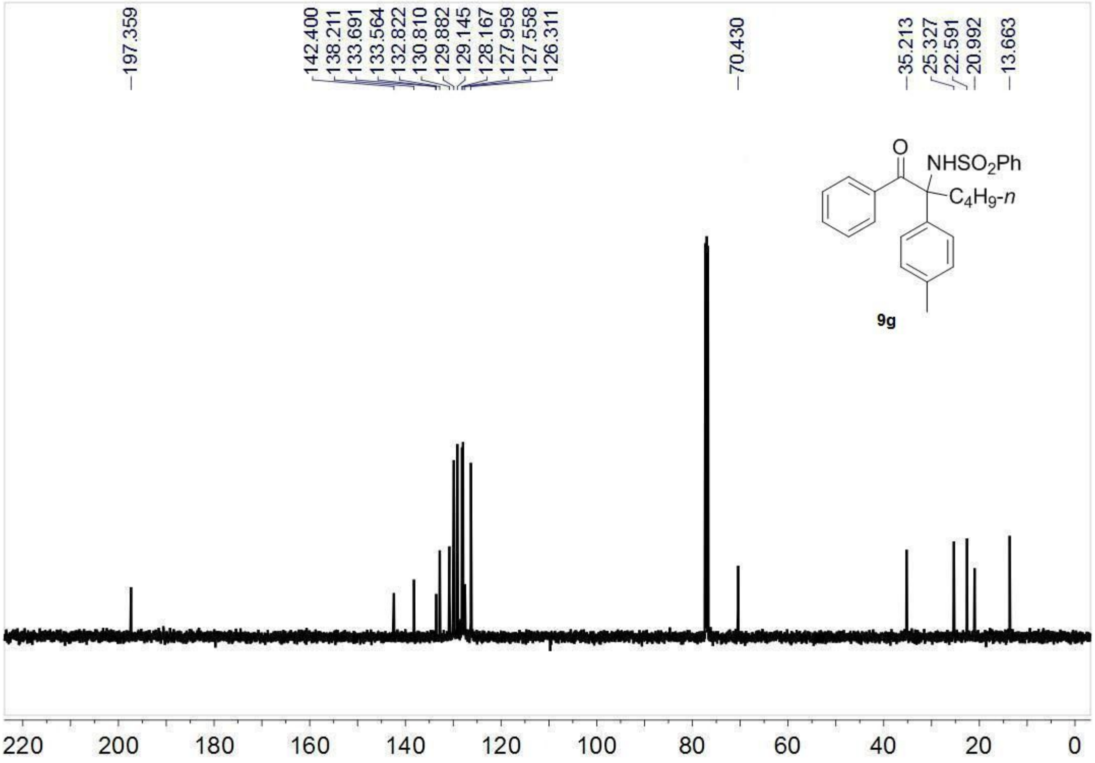

Supplementary Figure 78. ^{13}C NMR (125 MHz, $CDCl_3$) spectrum for 9g.

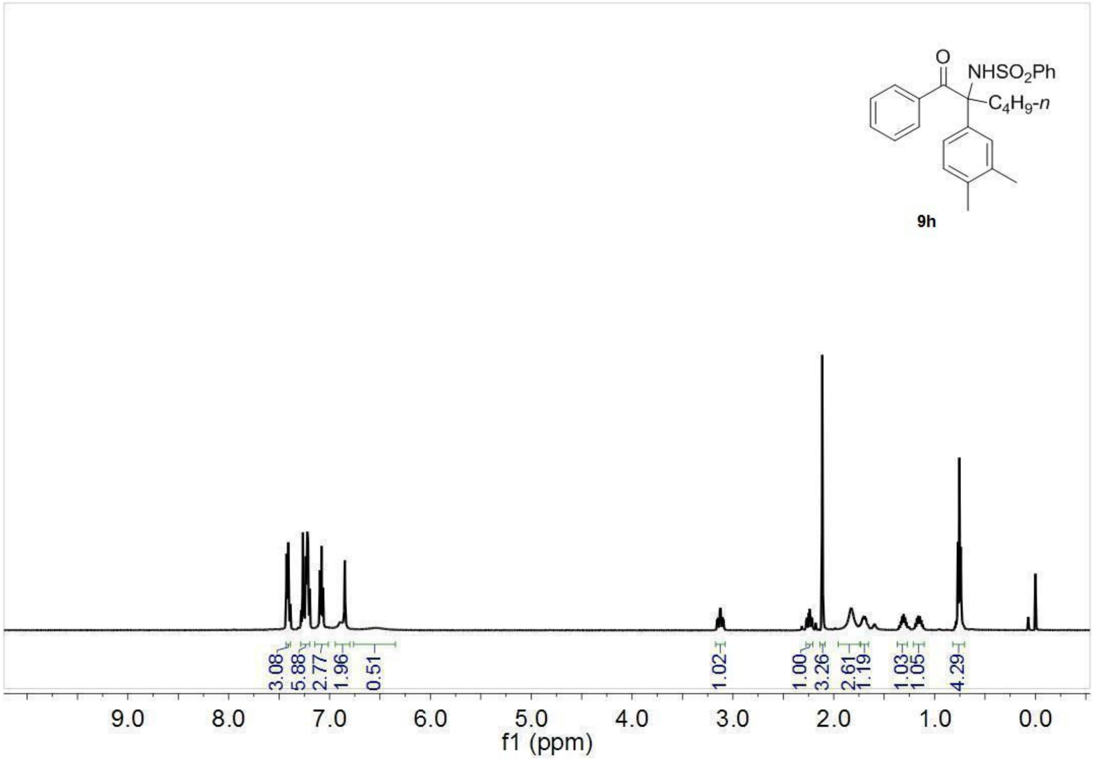

Supplementary Figure 79. ^{1}H NMR (500 MHz, CDCl$_3$) spectrum for 9h.

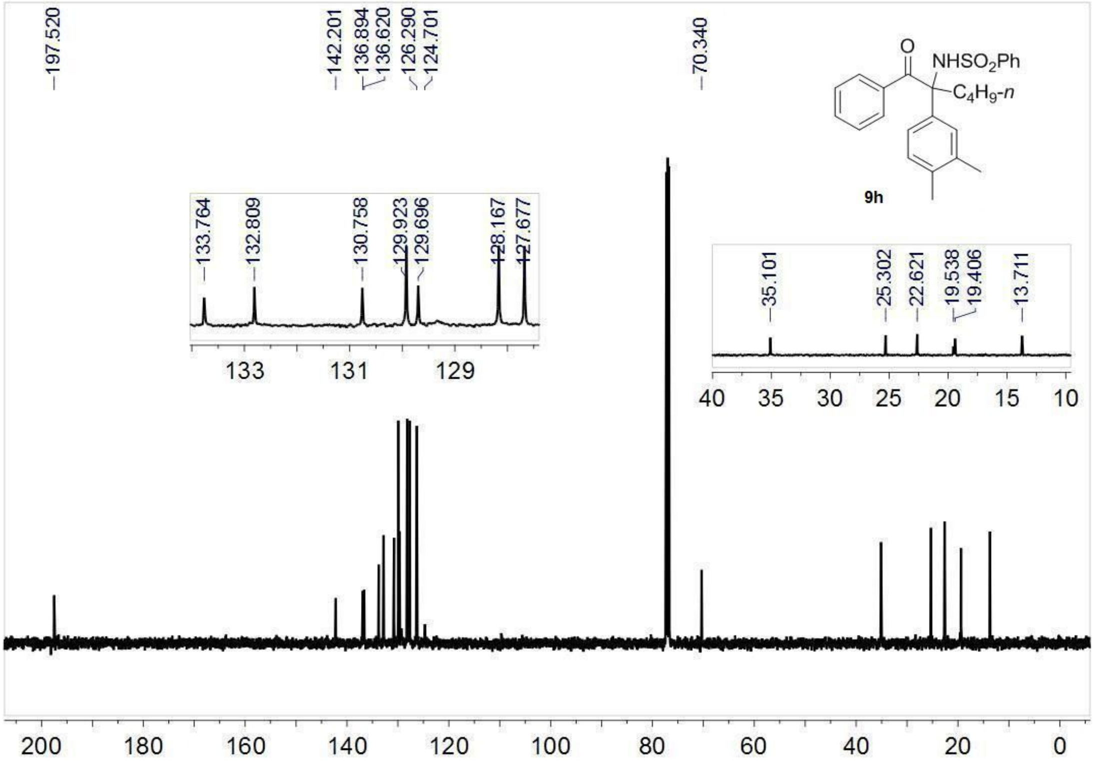

Supplementary Figure 80. ^{13}C NMR (125 MHz, CDCl$_3$) spectrum for 9h.

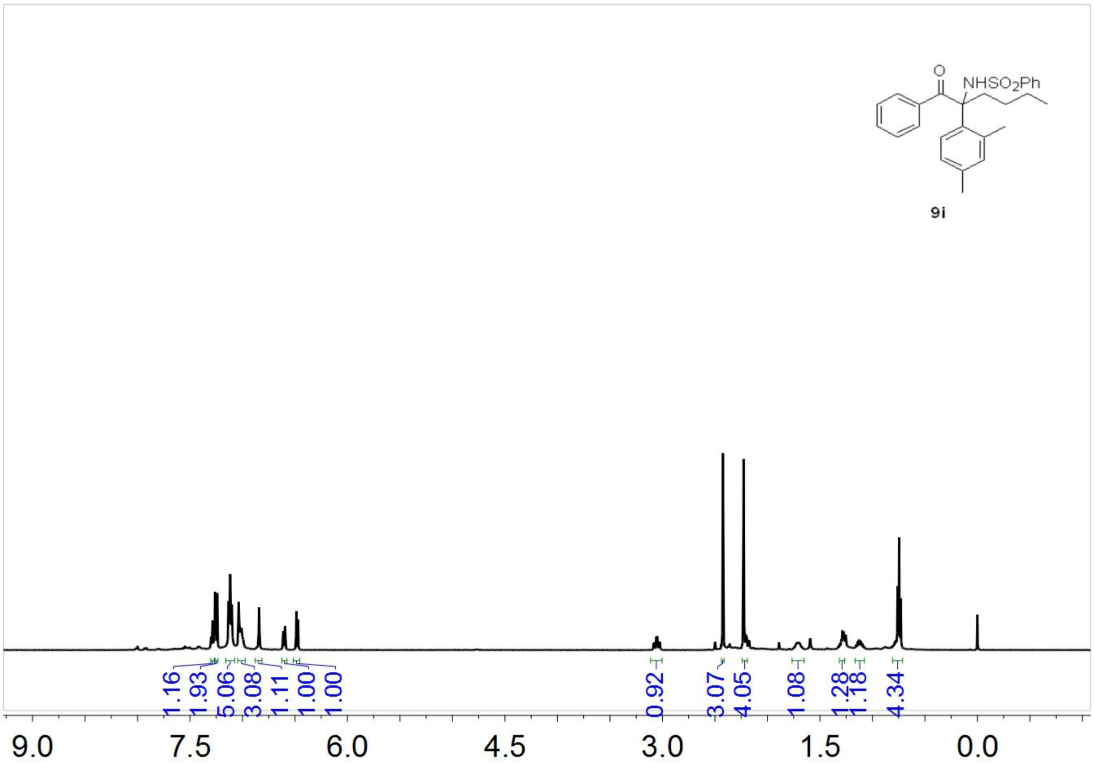

Supplementary Figure 81. ^{1}H NMR (500 MHz, $CDCl_3$) spectrum for 9i.

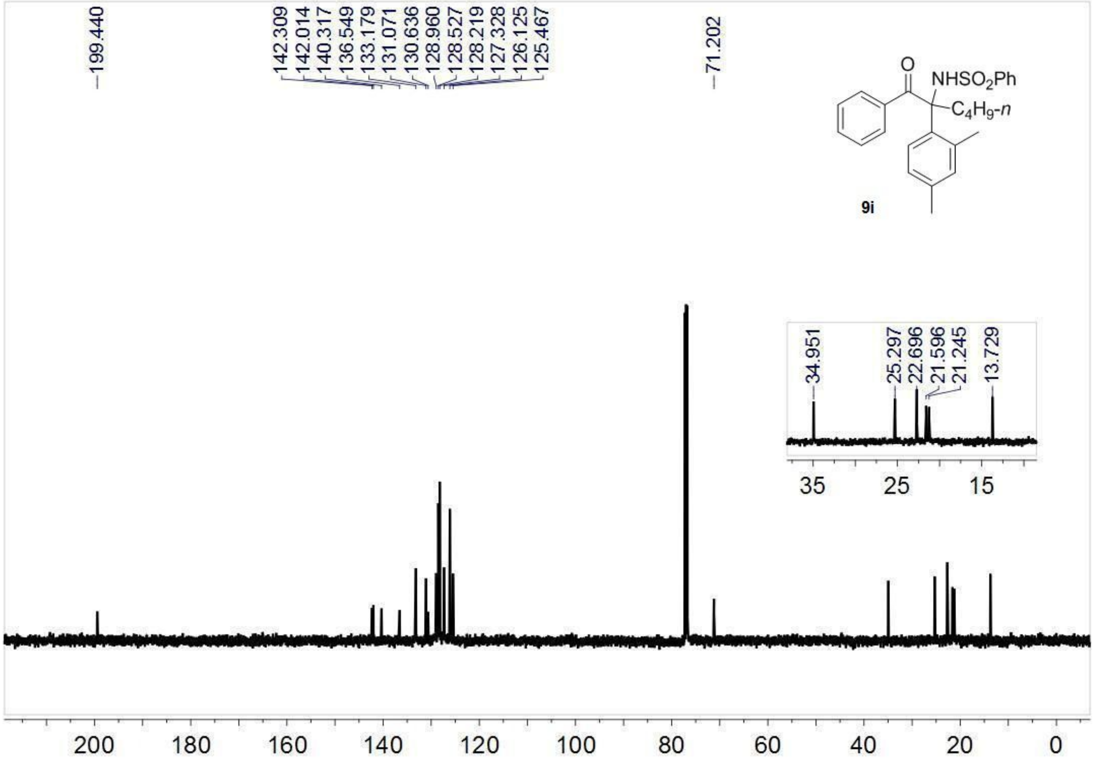

Supplementary Figure 82. ^{13}C NMR (125 MHz, $CDCl_3$) spectrum for 9i.

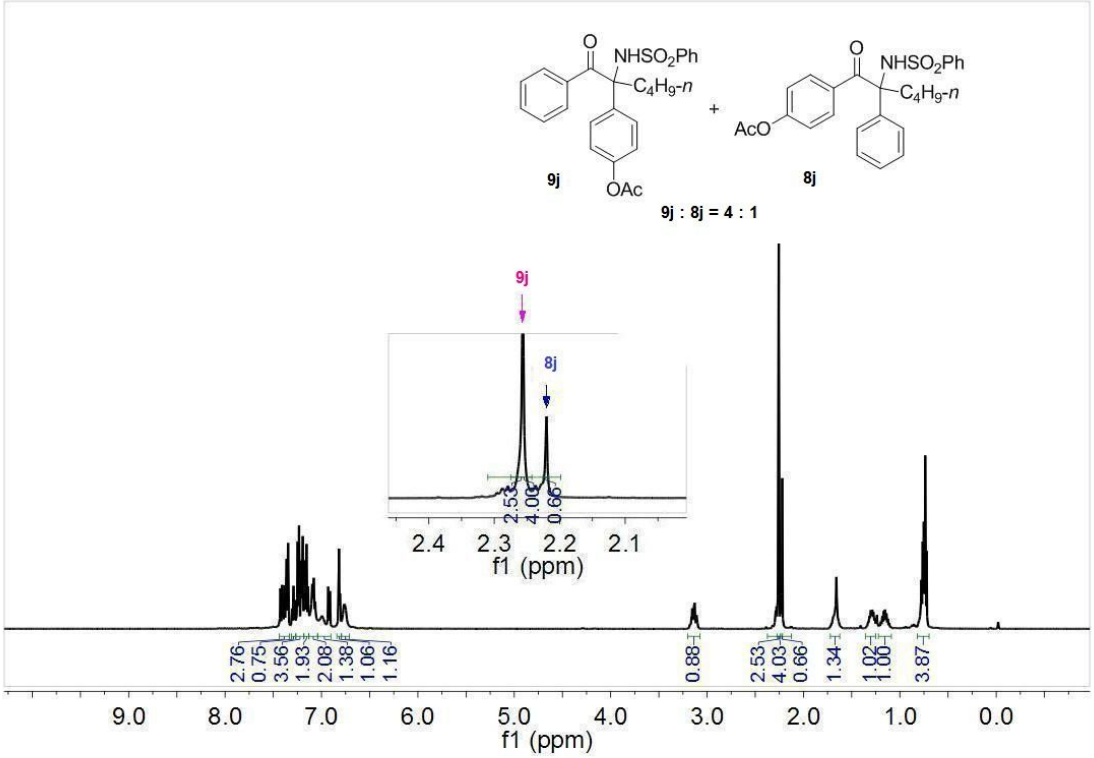

Supplementary Figure 83. ^{1}H NMR (500 MHz, $CDCl_3$) spectra for 8j and 9j.

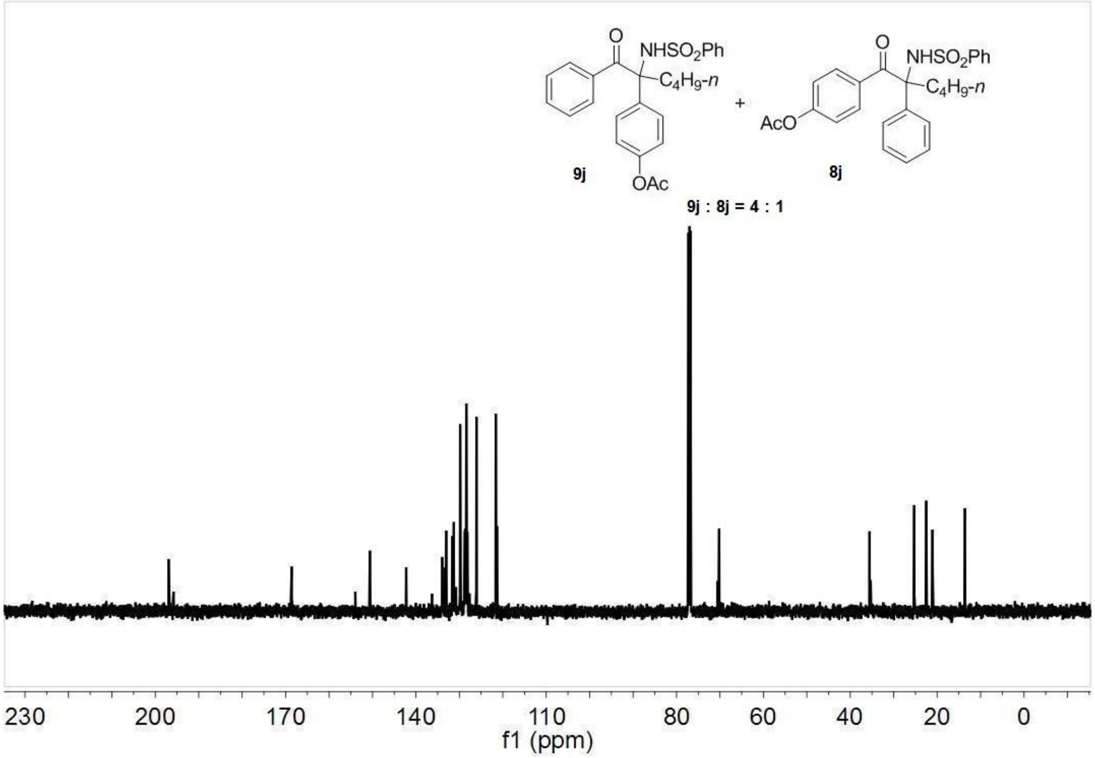

Supplementary Figure 84. ^{13}C NMR (125 MHz, $CDCl_3$) spectra for 8j and 9j.

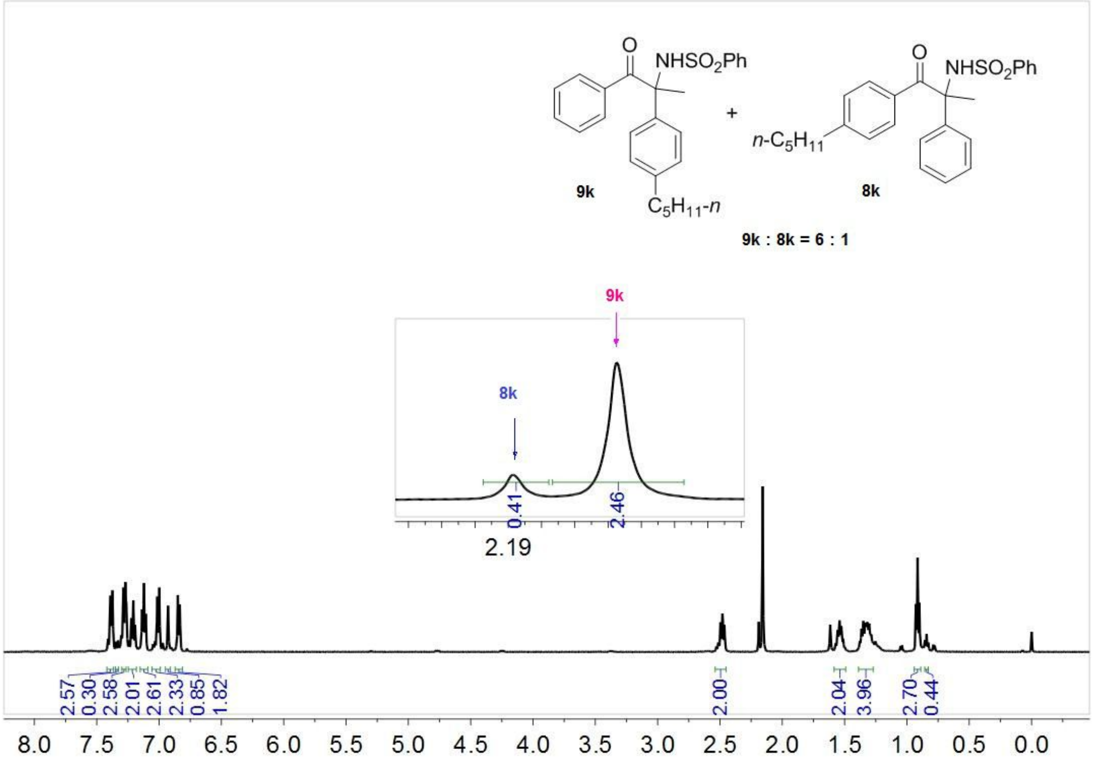

Supplementary Figure 85. ^{1}H NMR (500 MHz, $CDCl_3$) spectra for 8k and 9k.

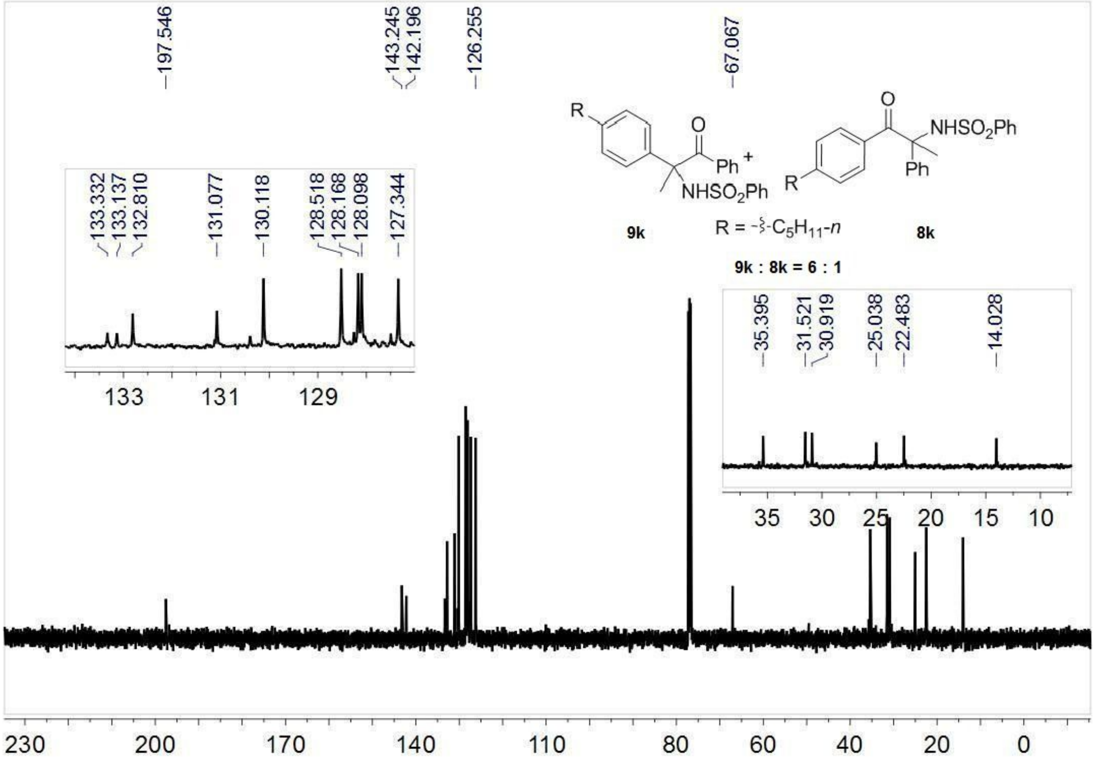

Supplementary Figure 86. ^{13}C NMR (125 MHz, $CDCl_3$) spectra for 8k and 9k.

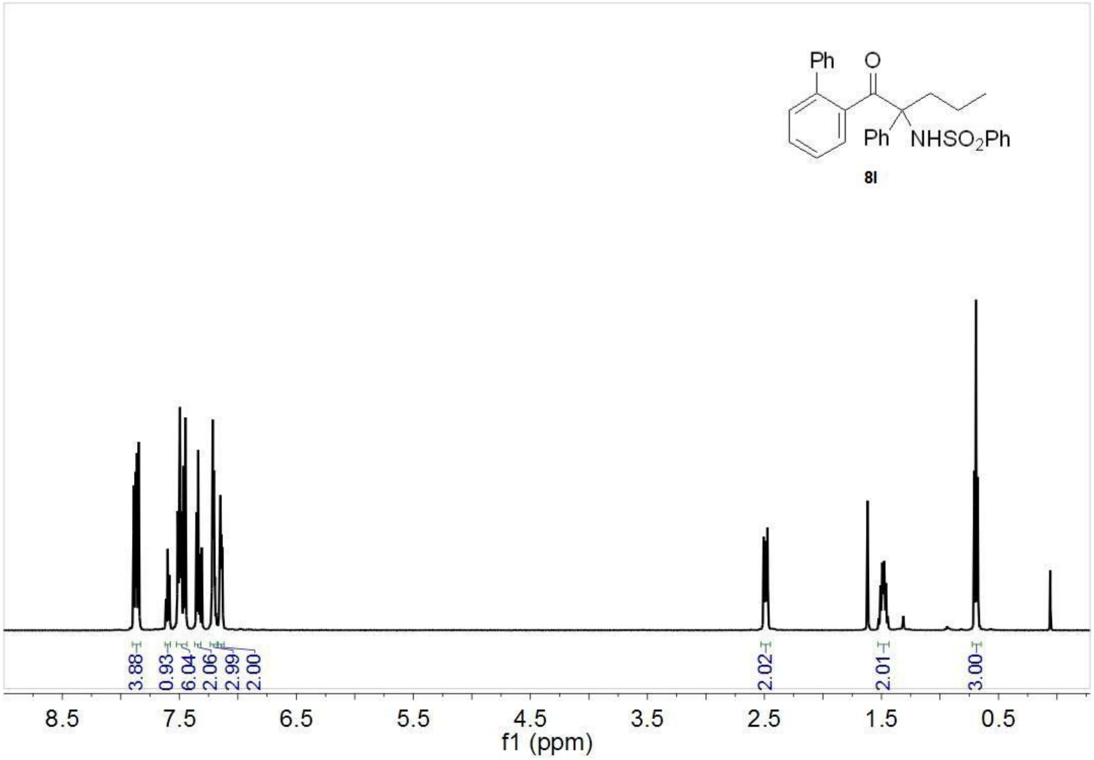

Supplementary Figure 87. ^{1}H NMR (400 MHz, $CDCl_3$) spectrum for 8l.

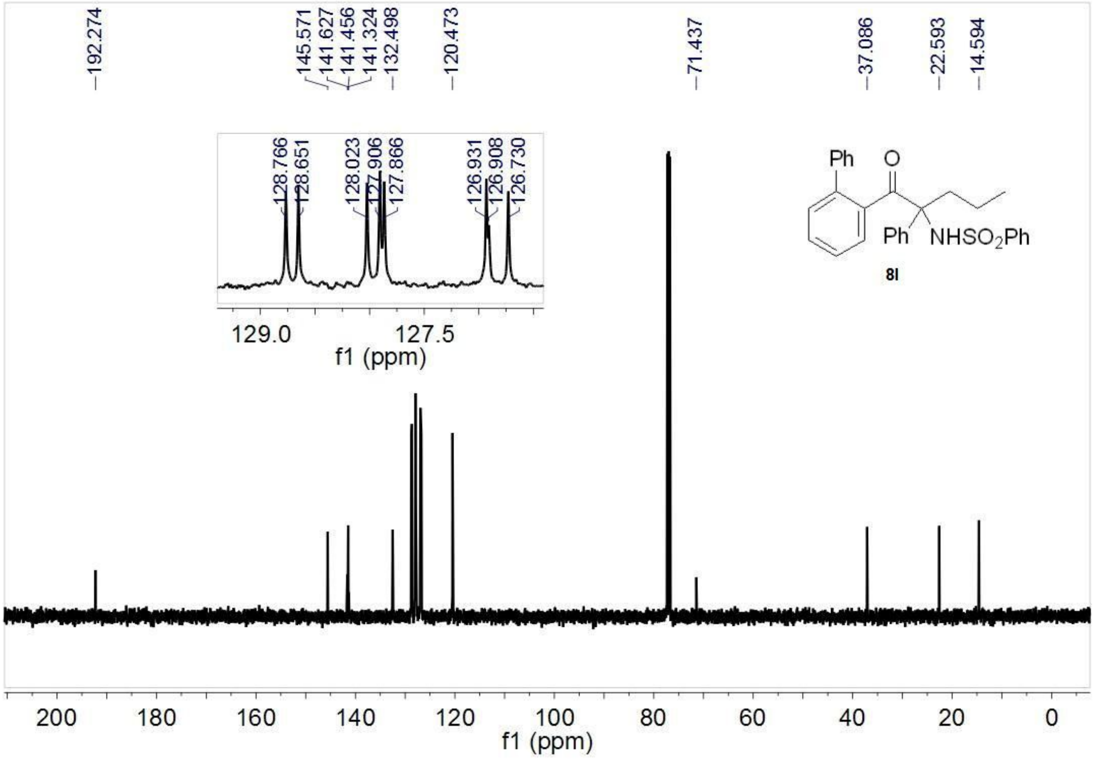

Supplementary Figure 88. ^{13}C NMR (100 MHz, $CDCl_3$) spectrum for 8l.

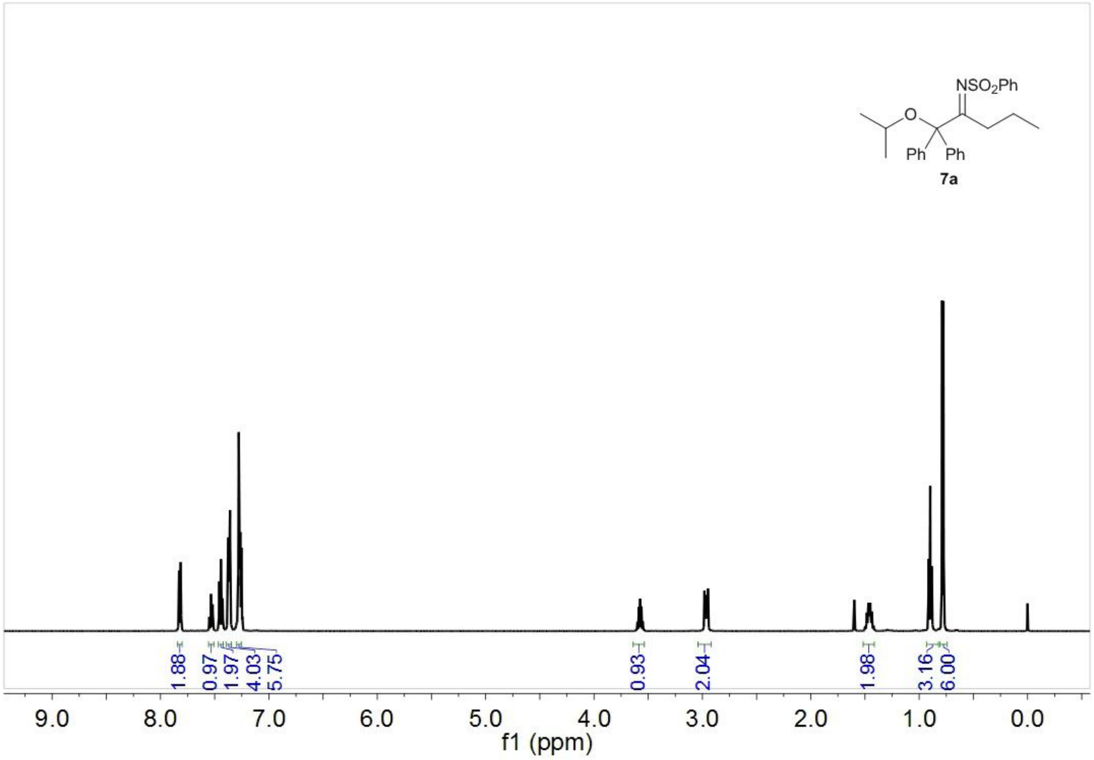

Supplementary Figure 89. ^{1}H NMR (500 MHz, $CDCl_3$) spectrum for 7a.

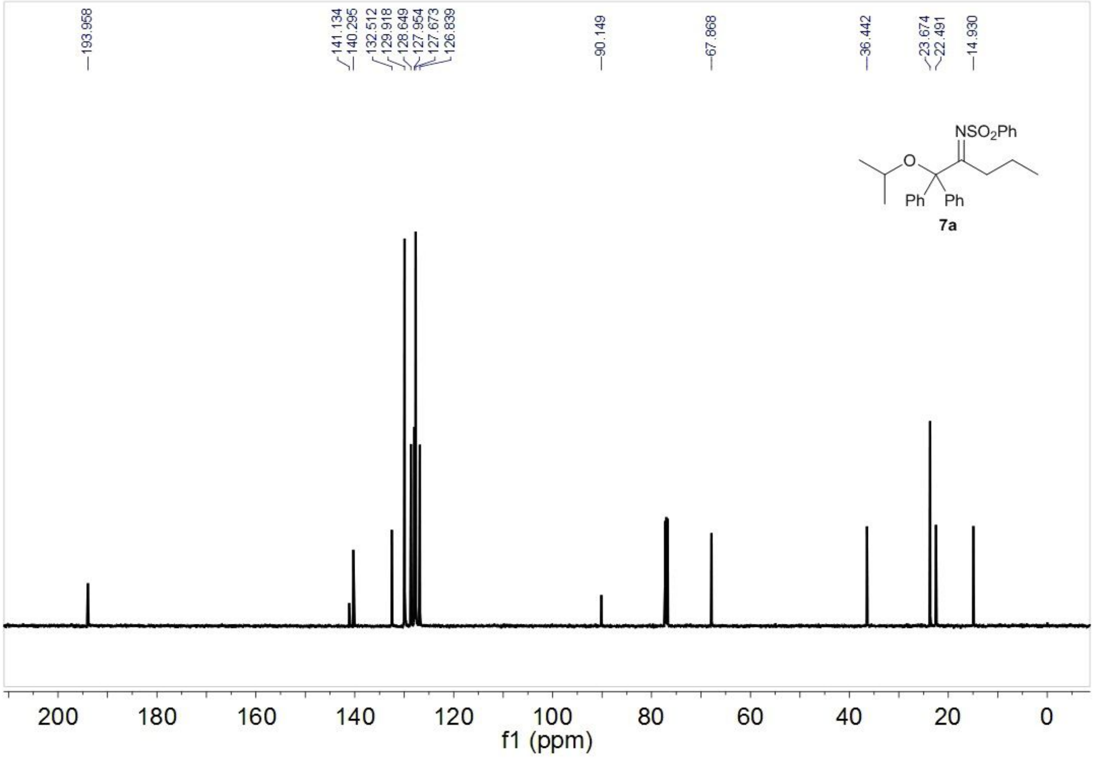

Supplementary Figure 90. ^{13}C NMR (125 MHz, $CDCl_3$) spectrum for 7a.

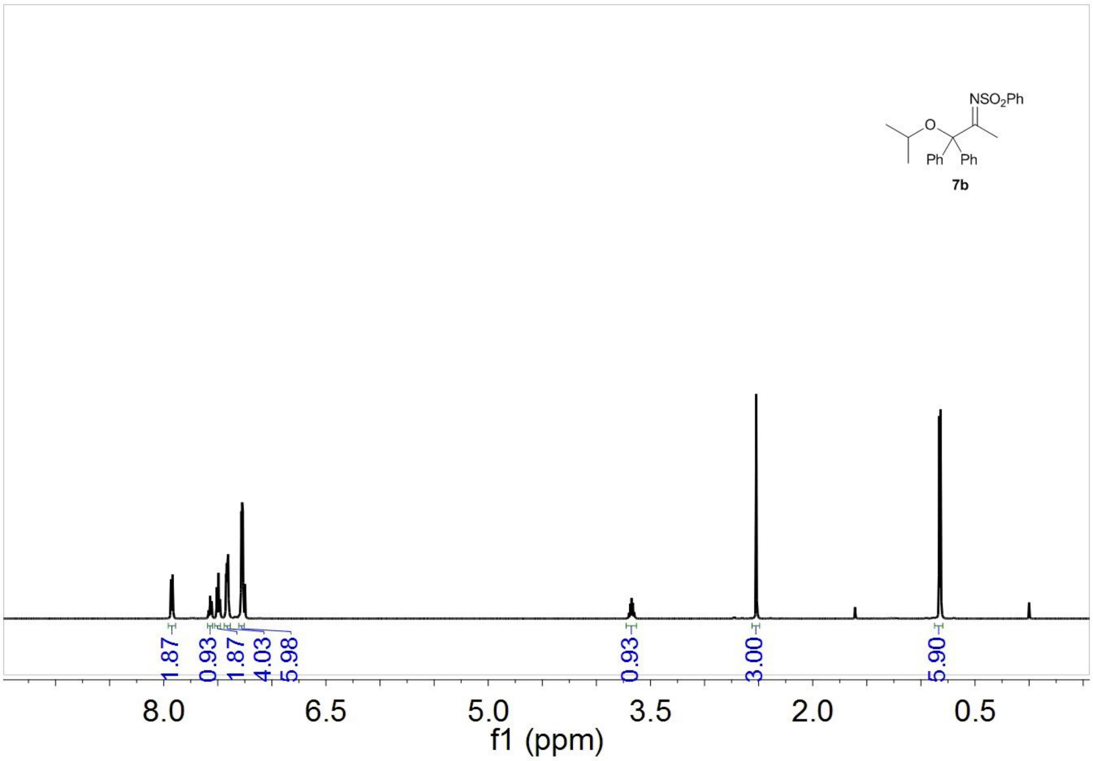

Supplementary Figure 91. ^{1}H NMR (500 MHz, $CDCl_3$) spectrum for 7b.

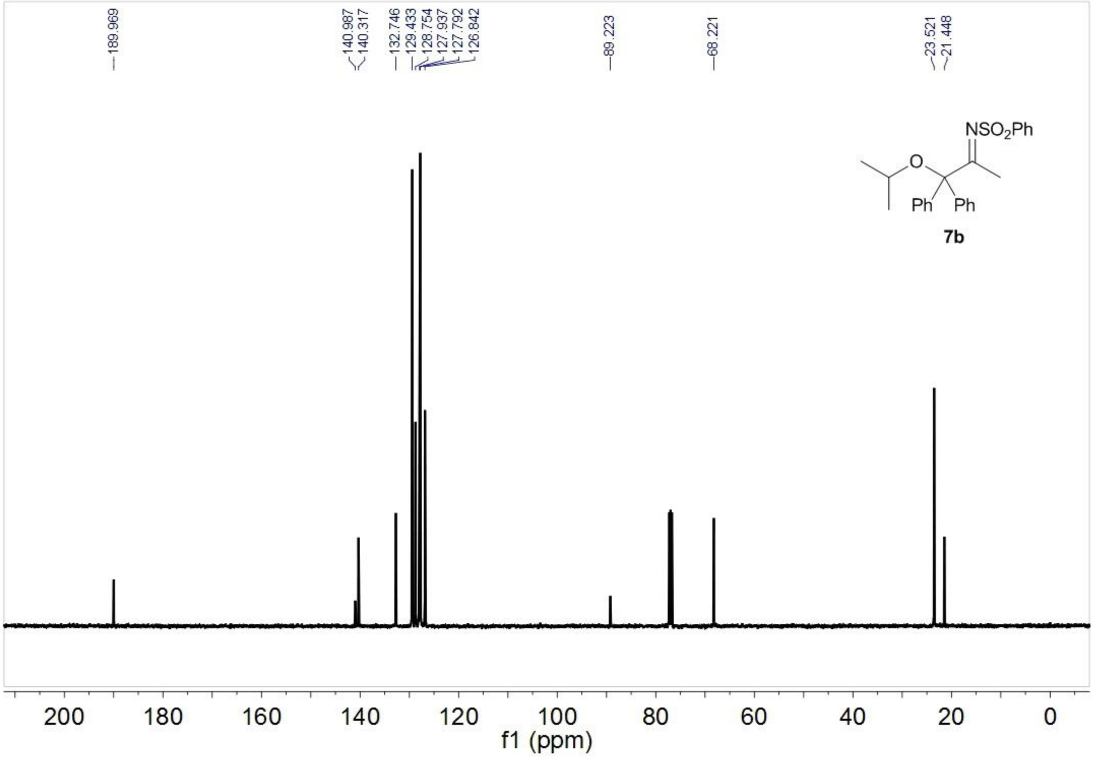

Supplementary Figure 92. ^{13}C NMR (125 MHz, $CDCl_3$) spectrum for 7b.

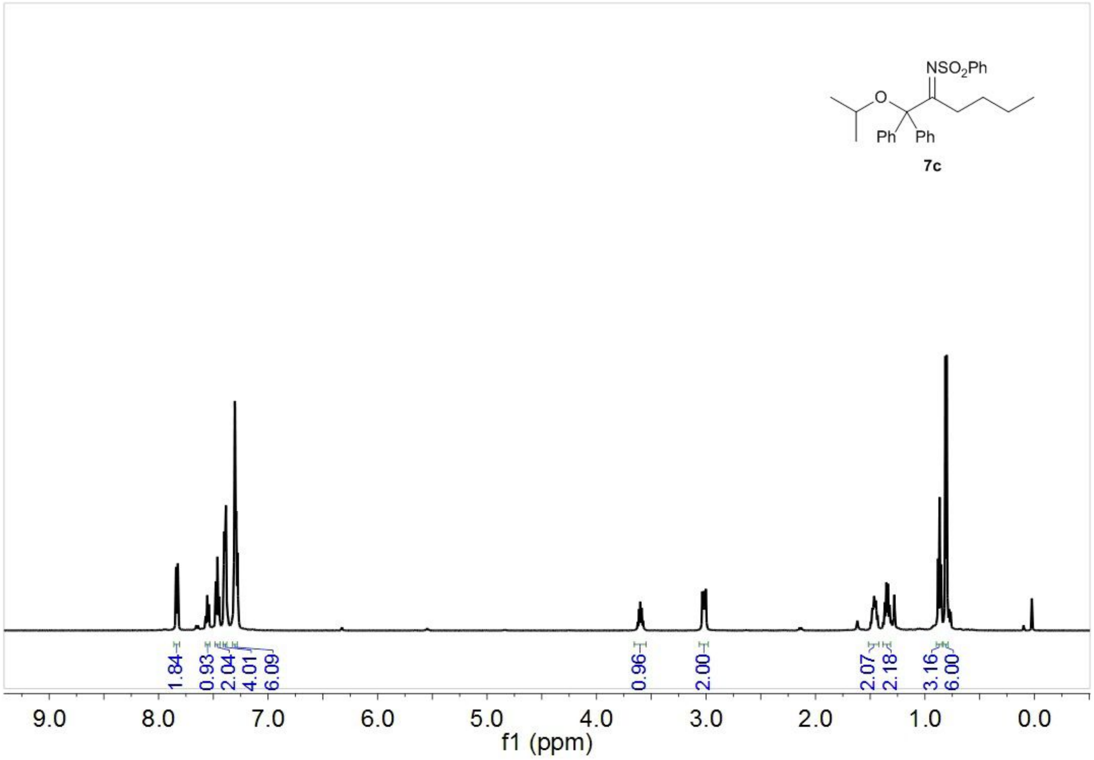

Supplementary Figure 93. ^{1}H NMR (500 MHz, $CDCl_3$) spectrum for 7c.

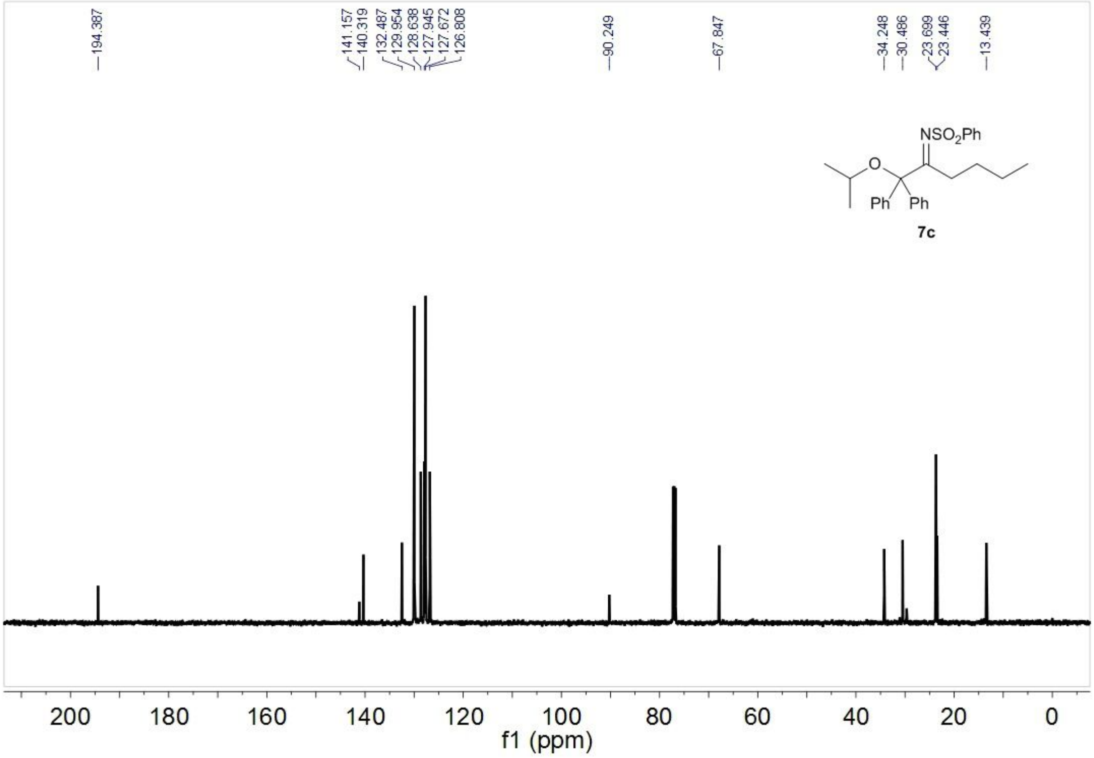

Supplementary Figure 94. ^{13}C NMR (125 MHz, $CDCl_3$) spectrum for 7c.

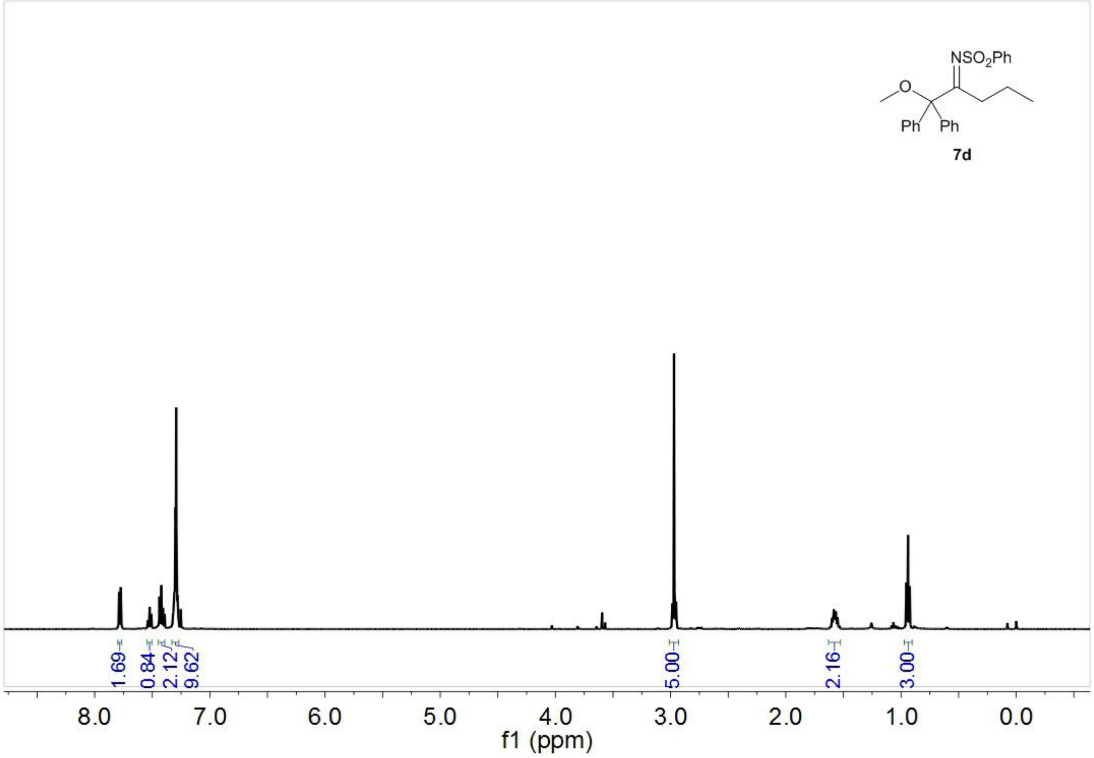

Supplementary Figure 95. ^{1}H NMR (500 MHz, $CDCl_3$) spectrum for 7d.

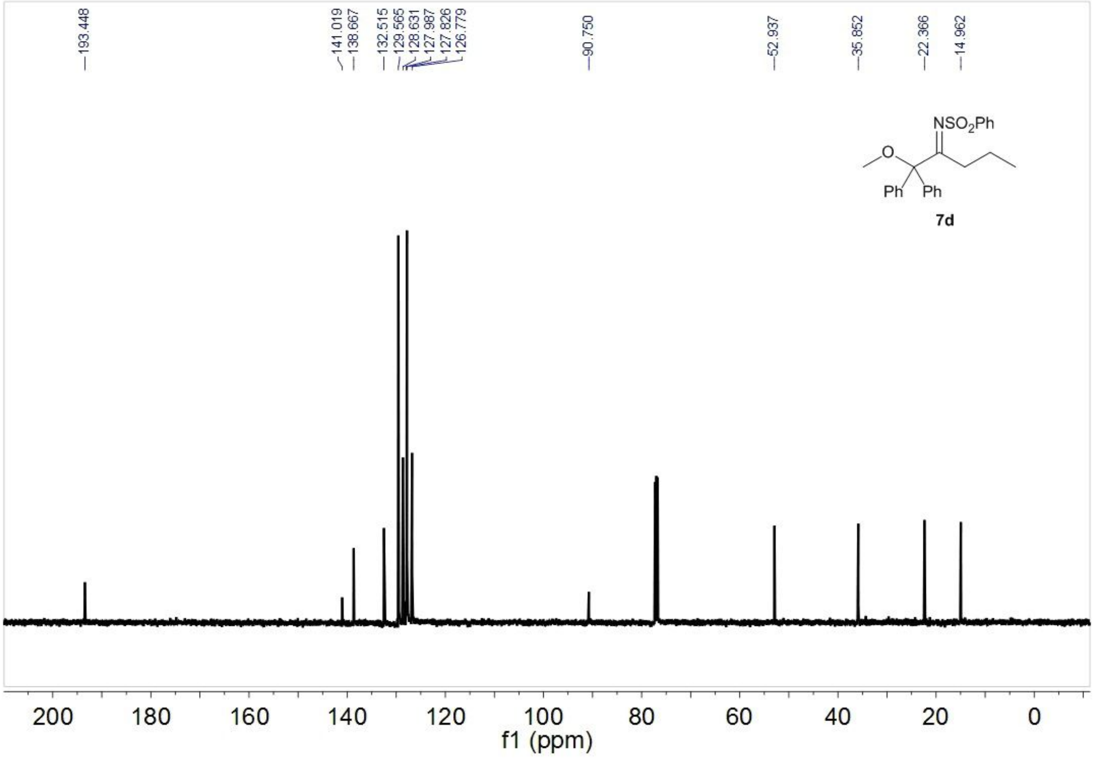

Supplementary Figure 96. ^{13}C NMR (125 MHz, $CDCl_3$) spectrum for 7d.

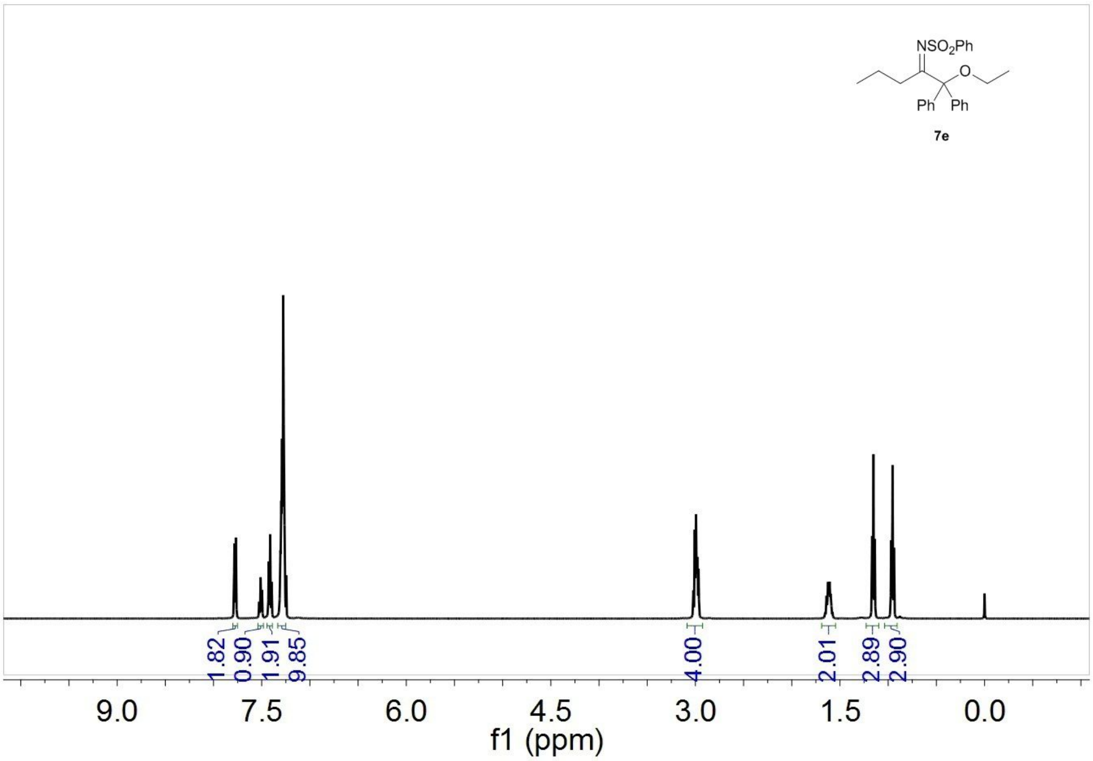

Supplementary Figure 97. ^{1}H NMR (500 MHz, $CDCl_3$) spectrum for 7e.

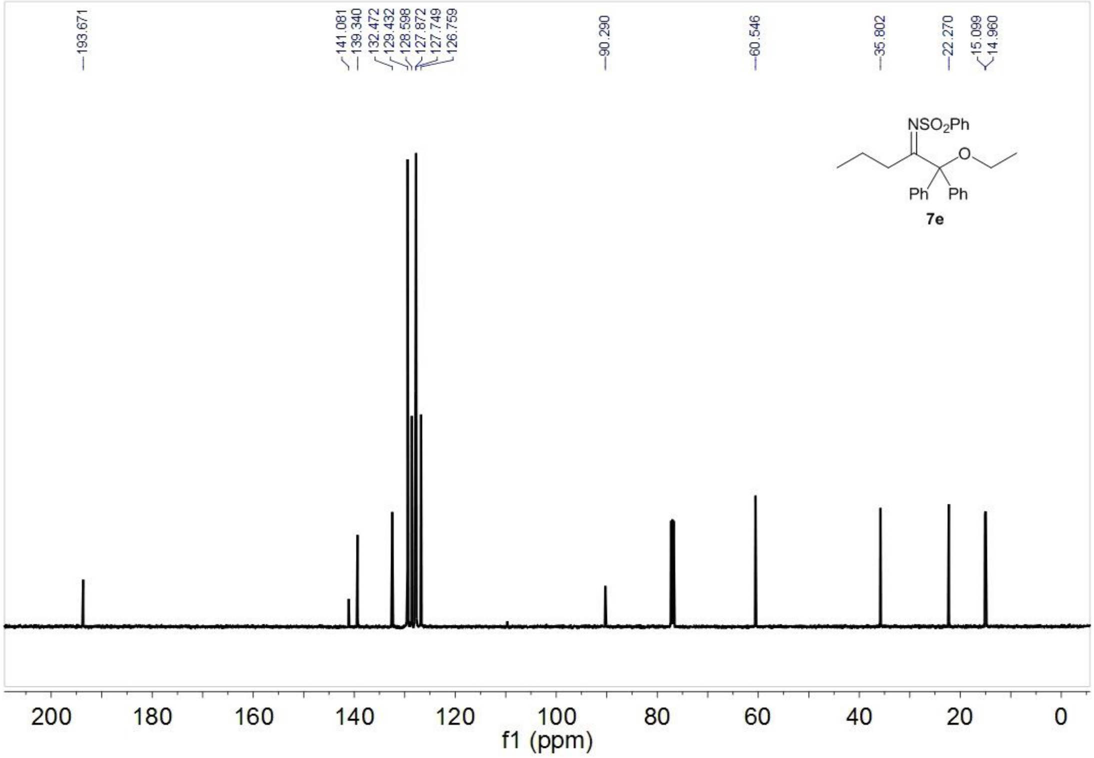

Supplementary Figure 98. ^{13}C NMR (125 MHz, $CDCl_3$) spectrum for 7e.

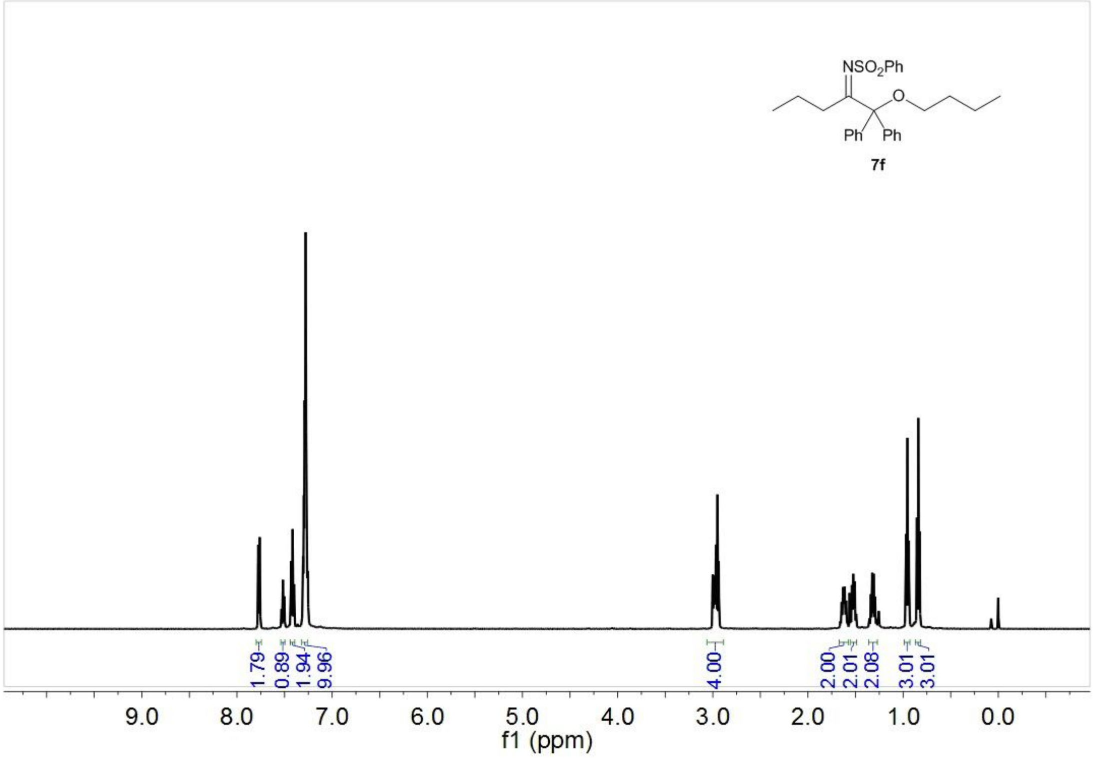

Supplementary Figure 99. **^{1}H NMR (500 MHz, $CDCl_3$) spectrum for 7f.**

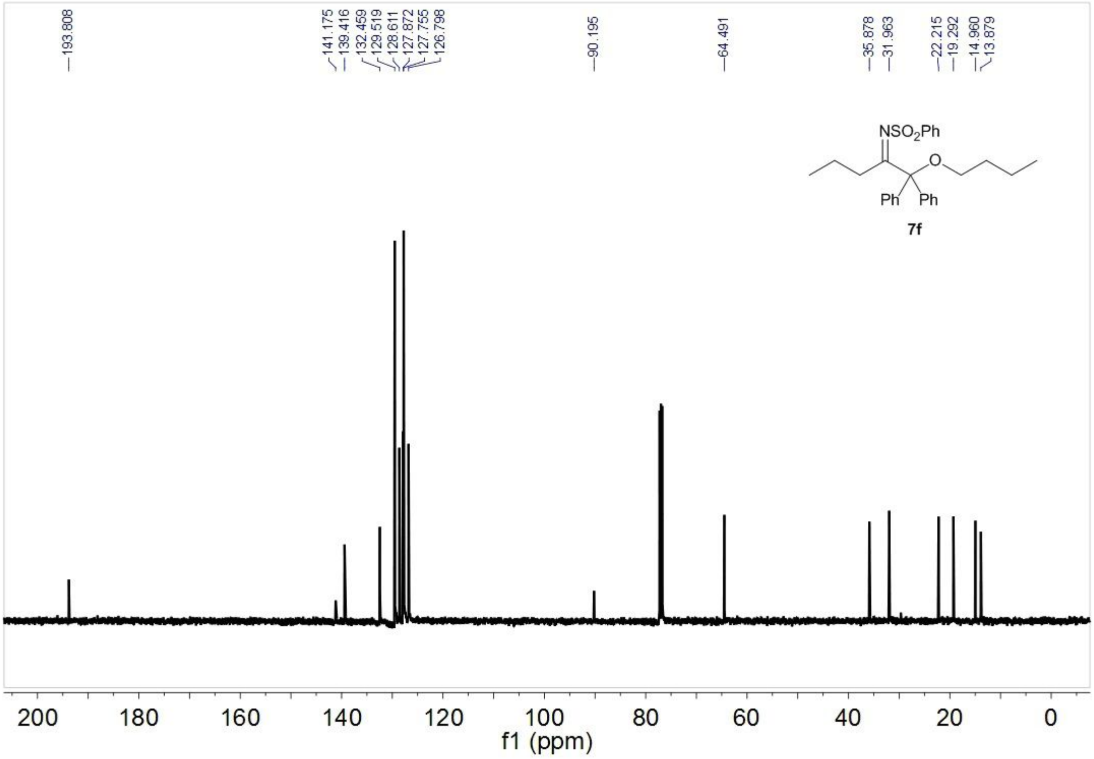

Supplementary Figure 100. **^{13}C NMR (125 MHz, $CDCl_3$) spectrum for 7f.**

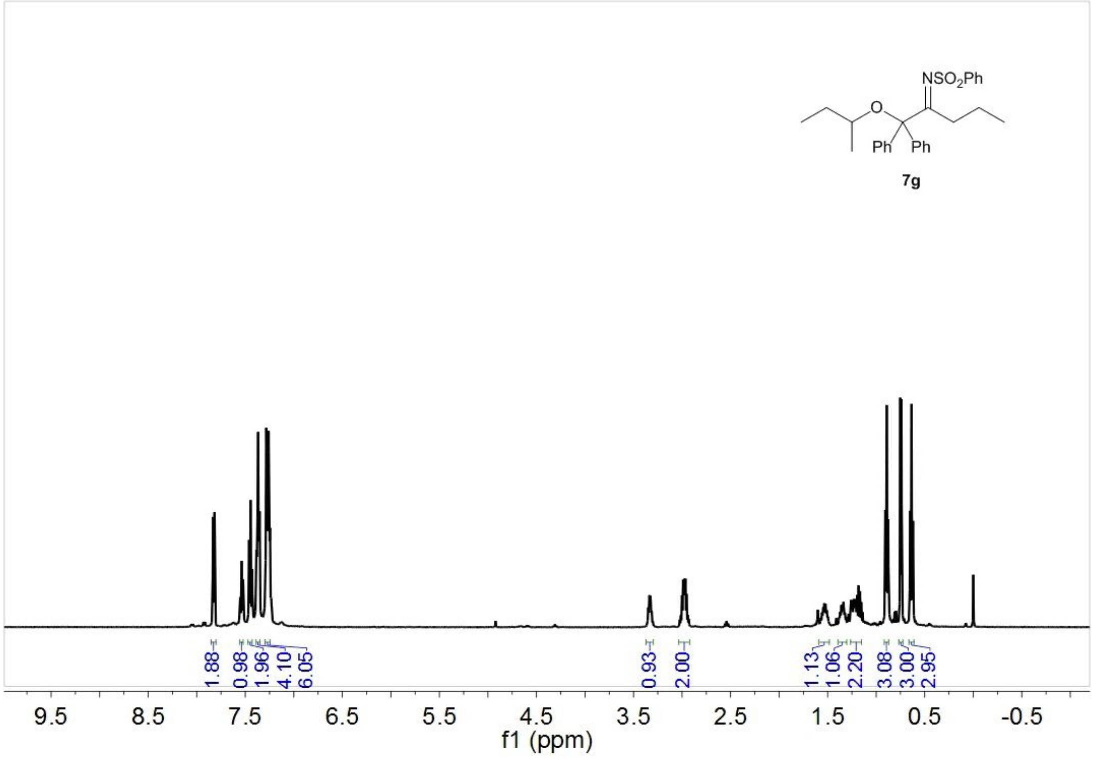

Supplementary Figure 101. ^{1}**H NMR (500 MHz, CDCl$_3$) spectrum for 7g.**

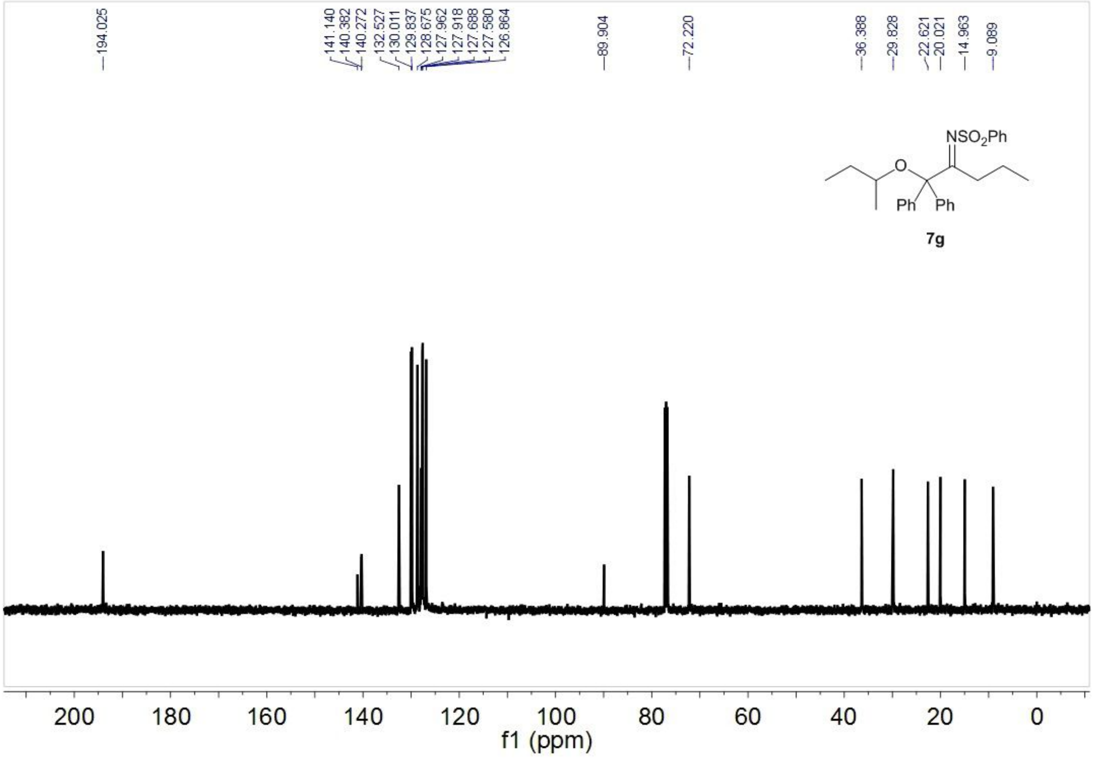

Supplementary Figure 102. ^{13}**C NMR (125 MHz, CDCl$_3$) spectrum for 7g.**

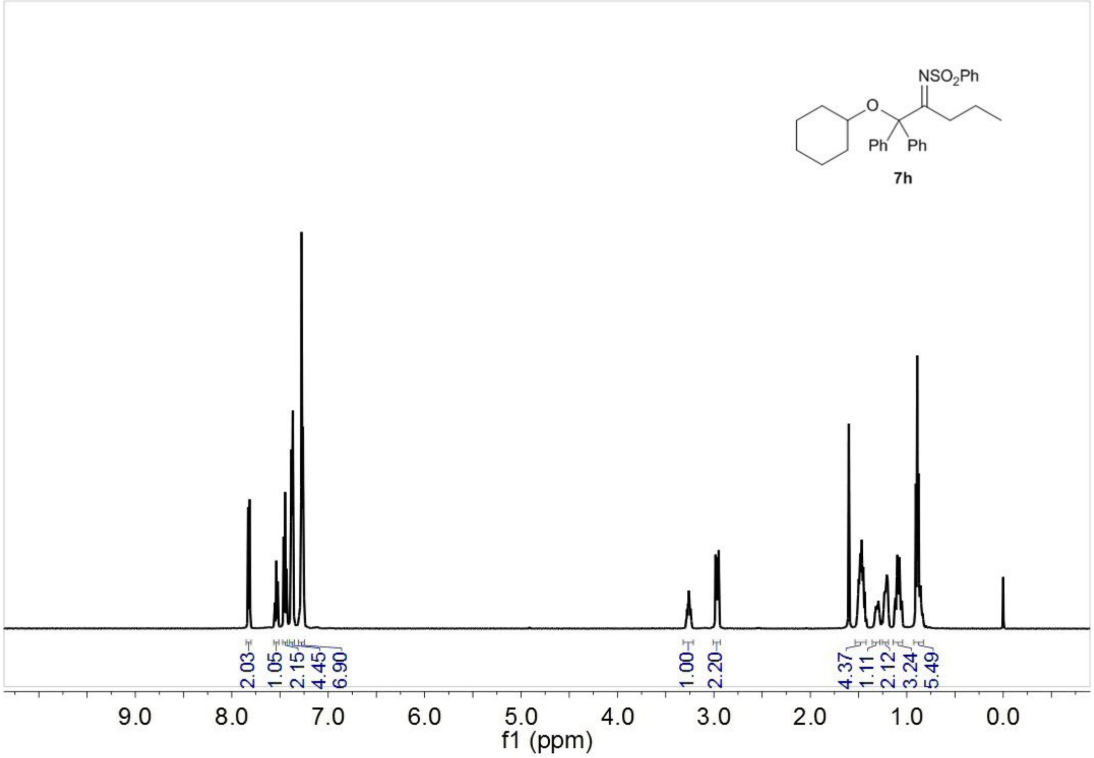

Supplementary Figure 103. **^{1}H NMR (500 MHz, CDCl$_3$) spectrum for 7h.**

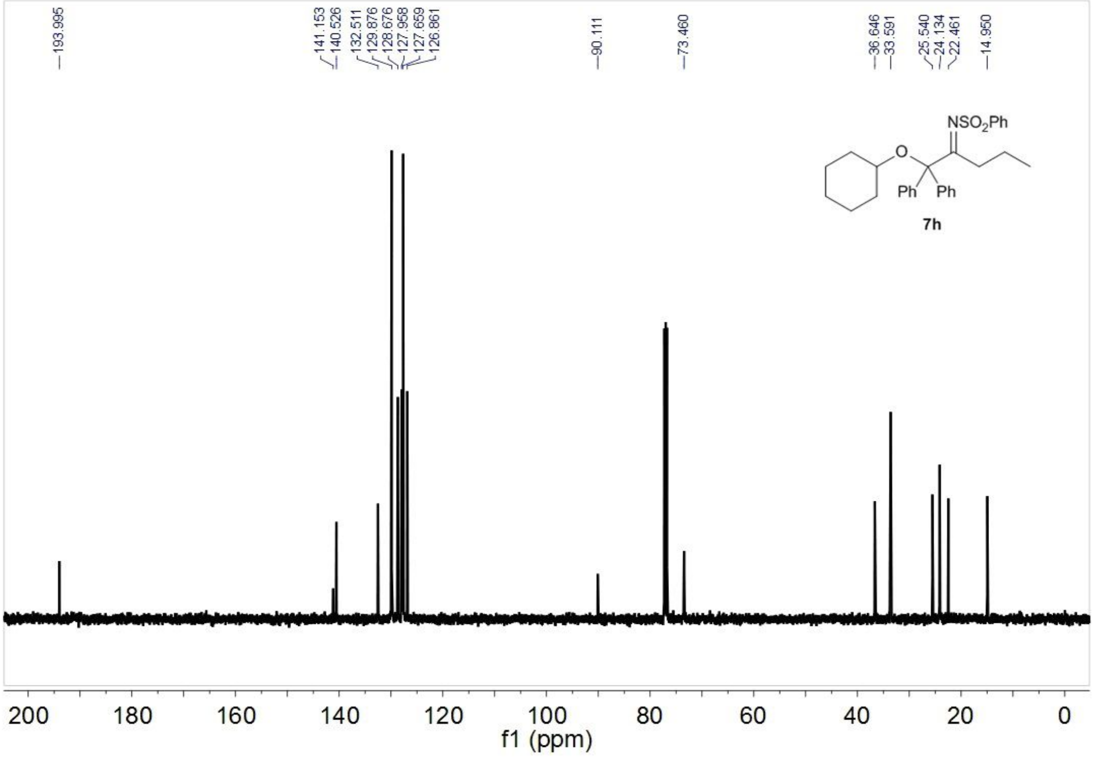

Supplementary Figure 104. **^{13}C NMR (125 MHz, CDCl$_3$) spectrum for 7h.**

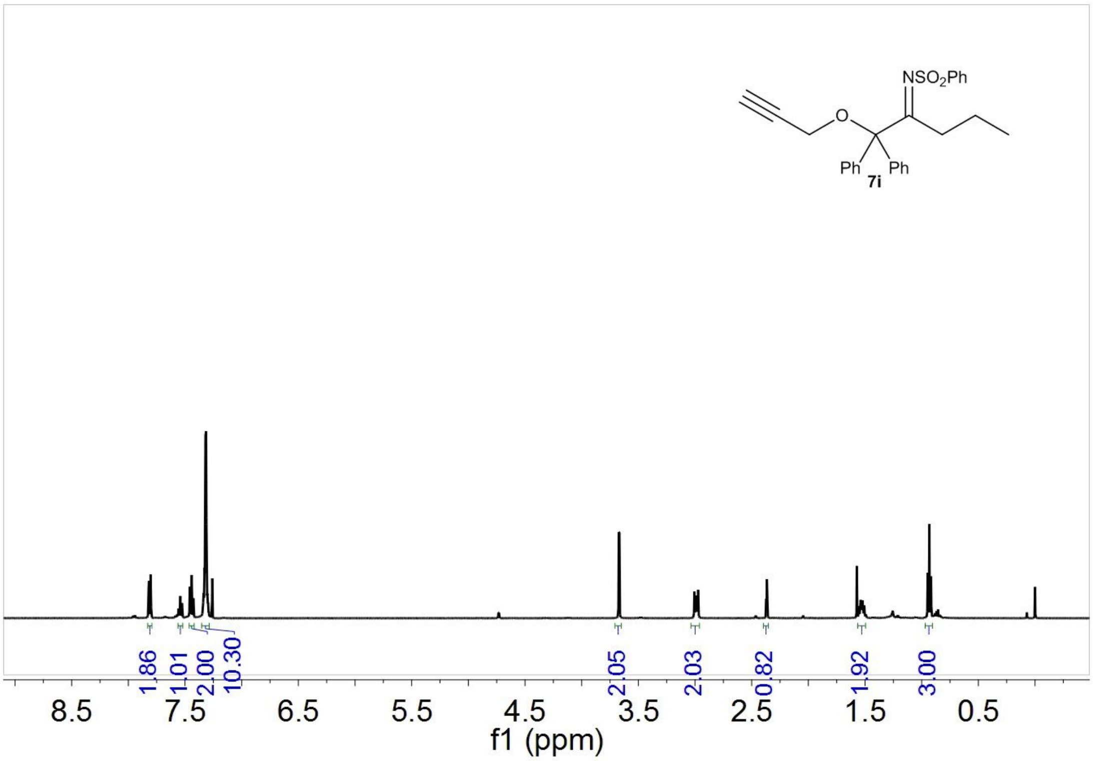

Supplementary Figure 105. ^{1}H NMR (500 MHz, $CDCl_3$) spectrum for 7i.

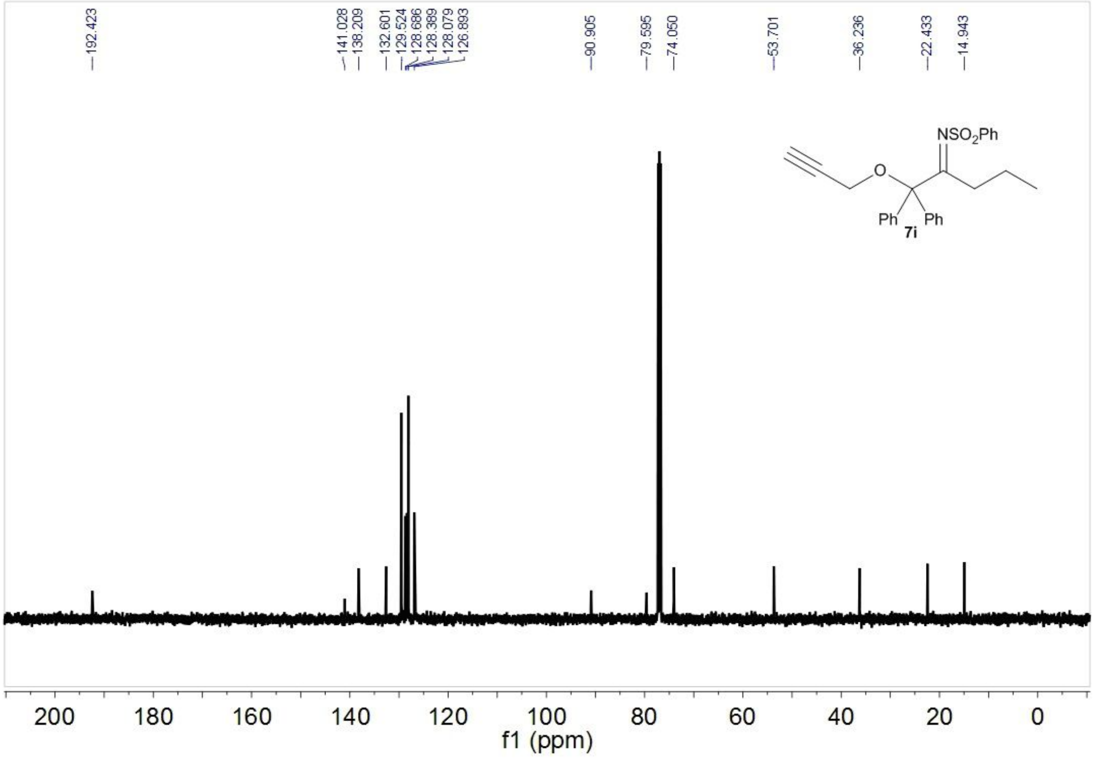

Supplementary Figure 106. ^{13}C NMR (125 MHz, $CDCl_3$) spectrum for 7i.

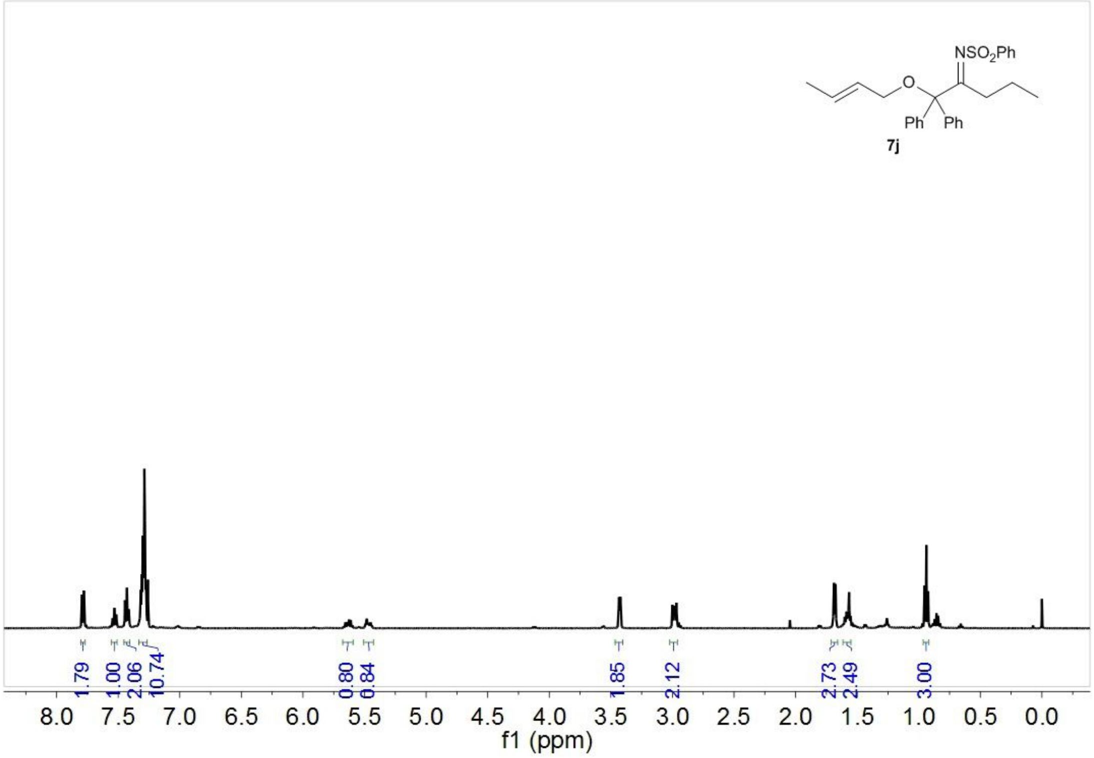

Supplementary Figure 107. **^{1}H NMR (500 MHz, $CDCl_3$) spectrum for 7j.**

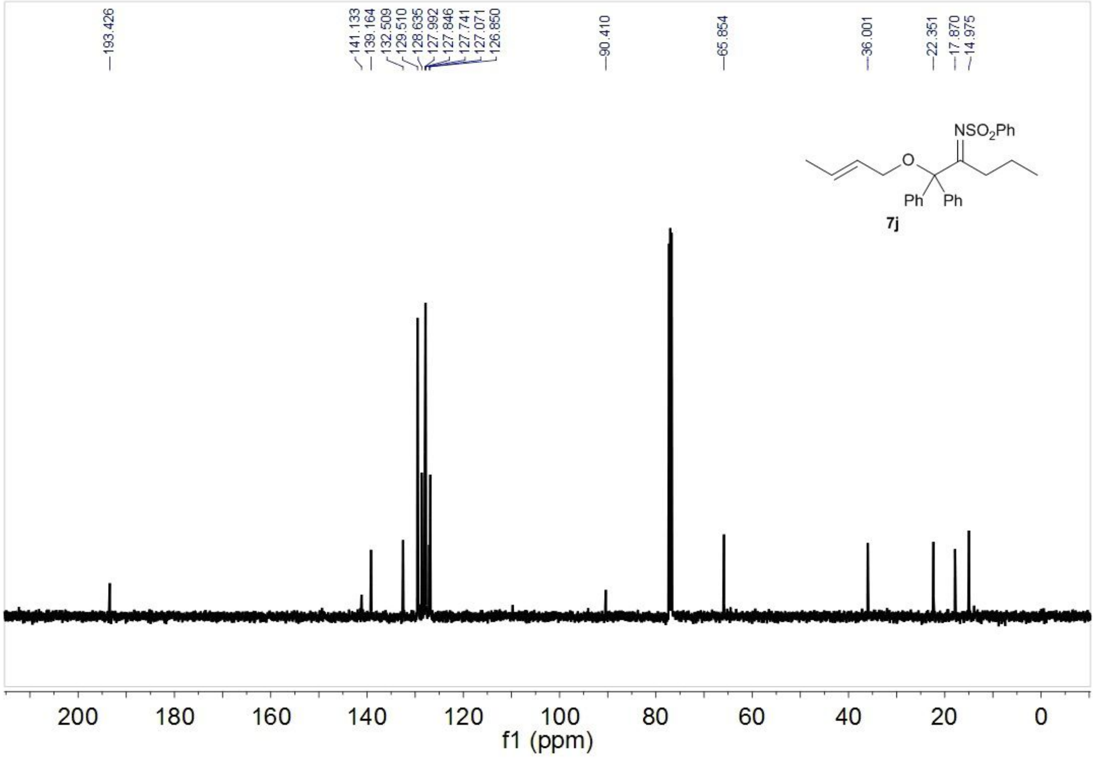

Supplementary Figure 108. **^{13}C NMR (125 MHz, $CDCl_3$) spectrum for 7j.**

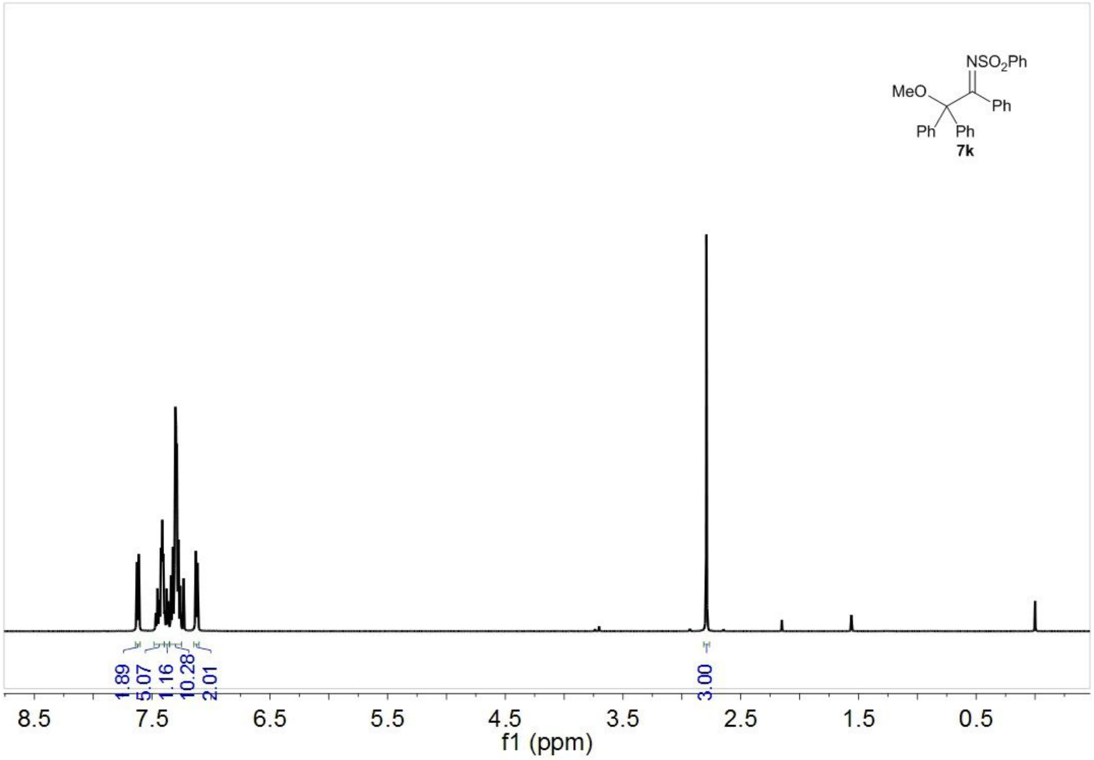

Supplementary Figure 109. ^{1}**H NMR (500 MHz, CDCl**$_3$**) spectrum for 7k.**

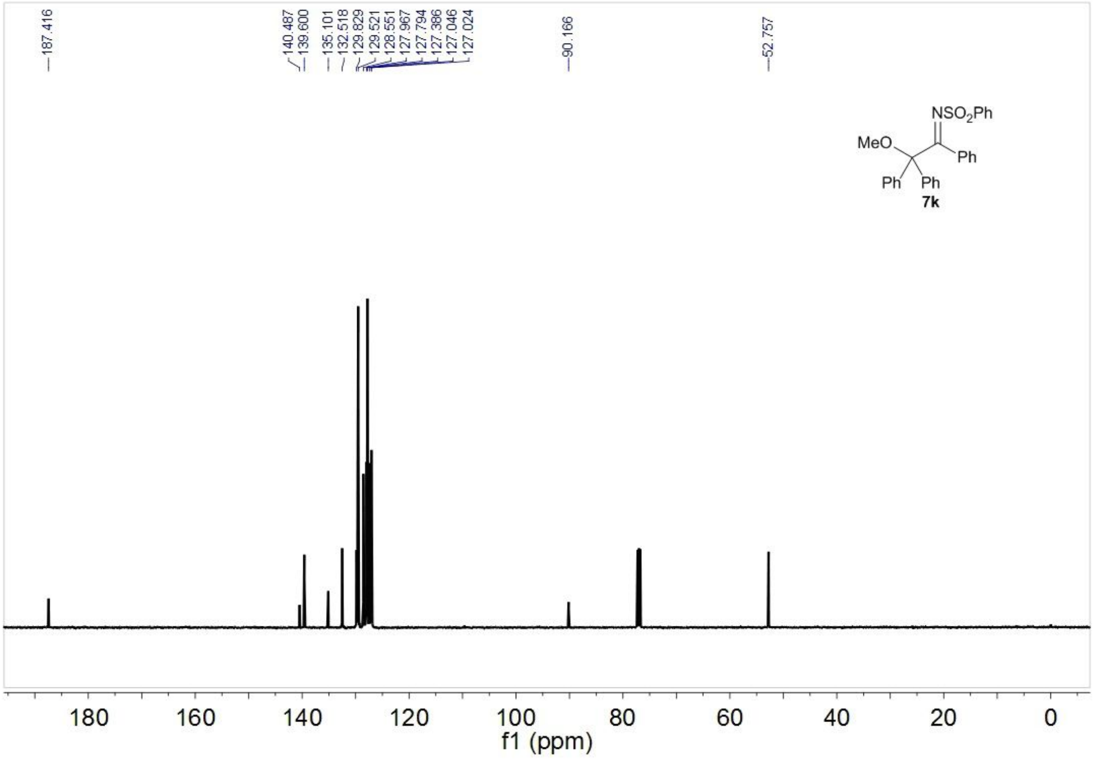

Supplementary Figure 110. ^{13}**C NMR (125 MHz, CDCl**$_3$**) spectrum for 7k.**

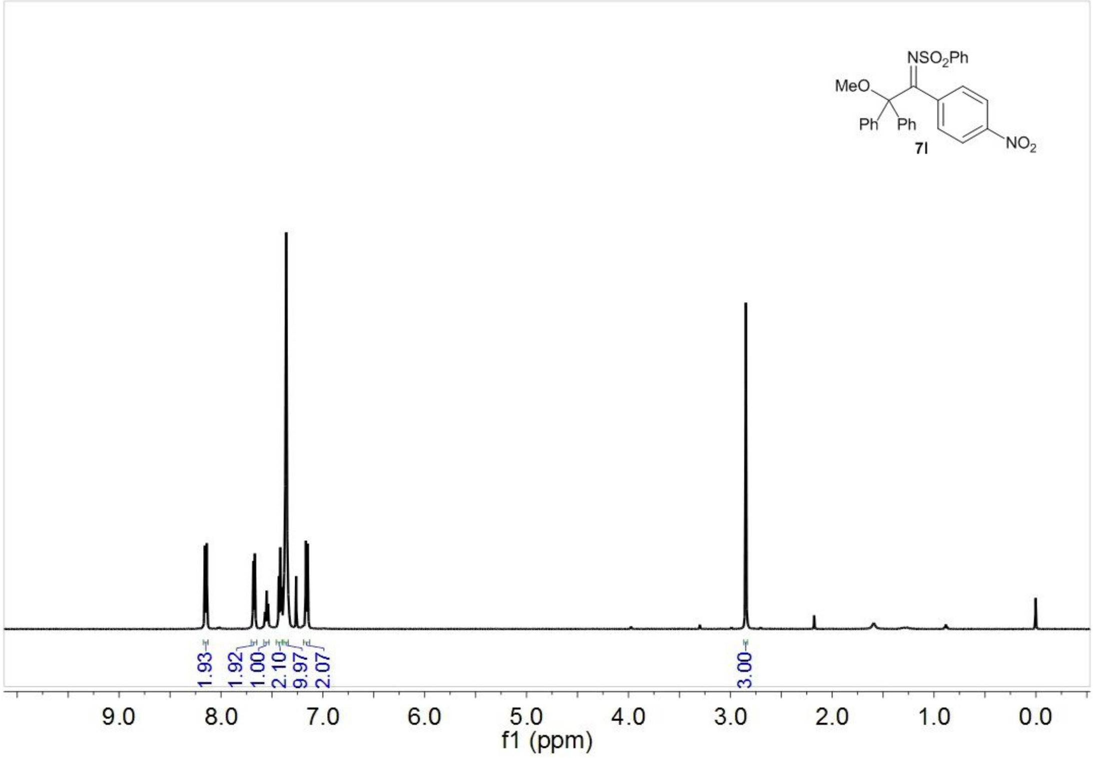

Supplementary Figure 111. ^{1}**H NMR (500 MHz,** $CDCl_3$**) spectrum for 7l.**

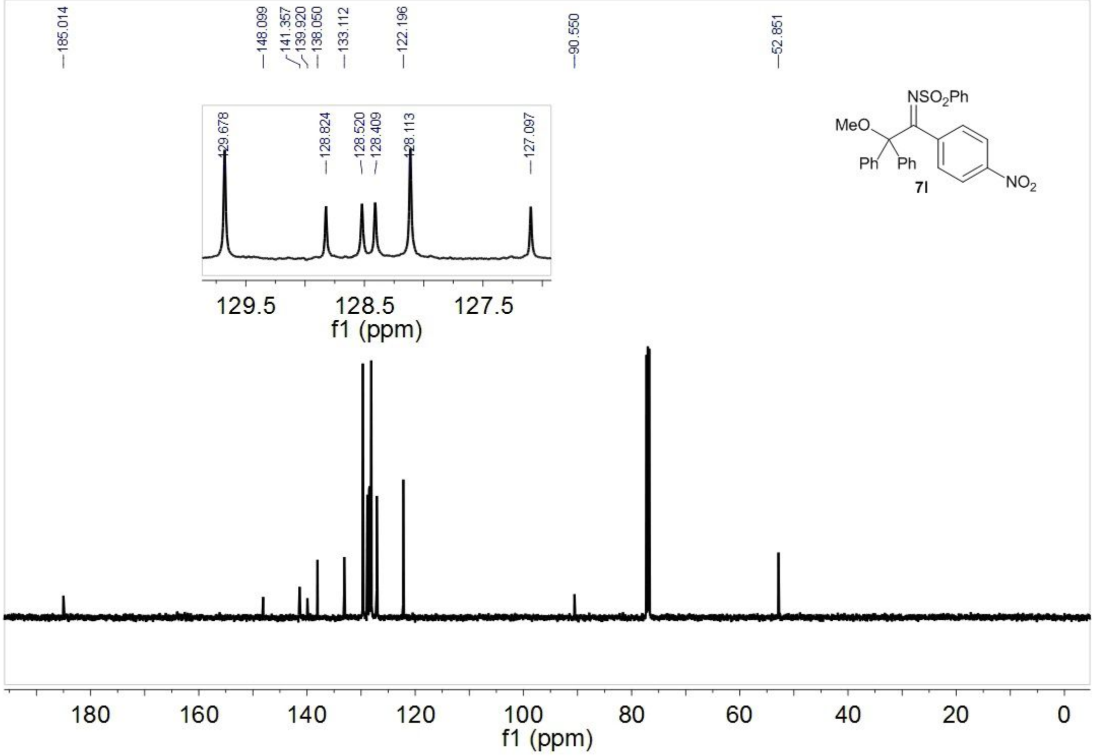

Supplementary Figure 112. ^{13}**C NMR (125 MHz,** $CDCl_3$**) spectrum for 7l.**

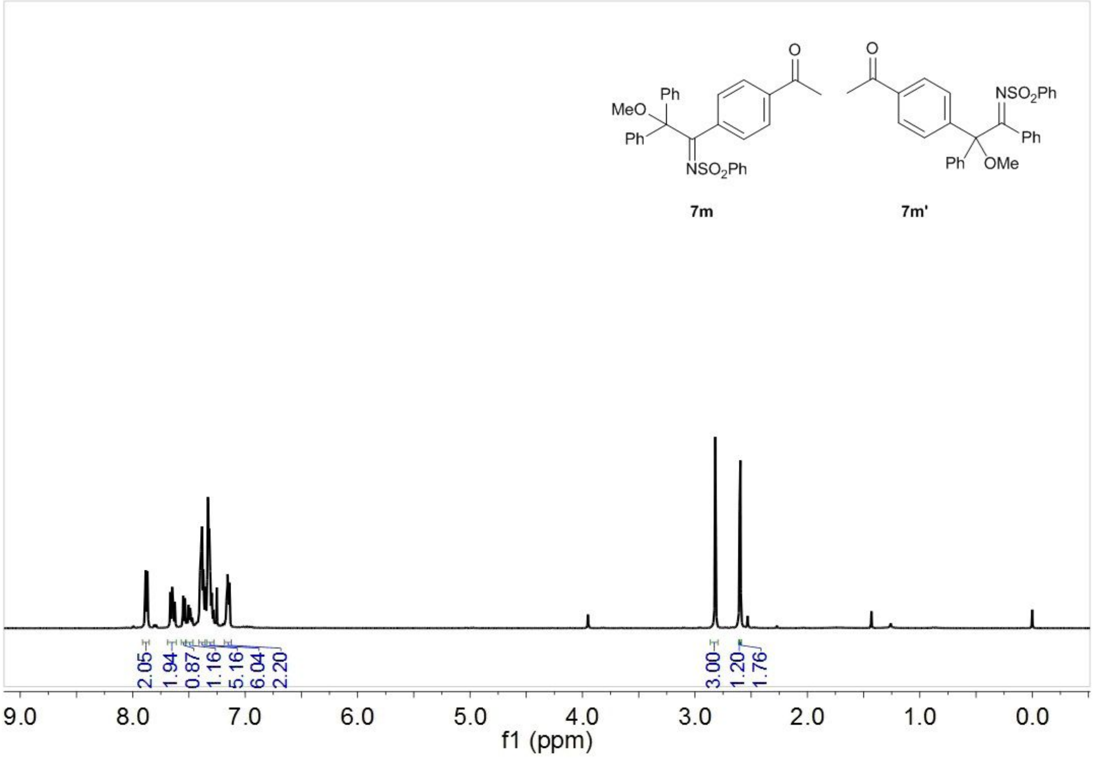

Supplementary Figure 113. ^{1}H NMR (500 MHz, $CDCl_3$) spectra for 7l and 7l'.

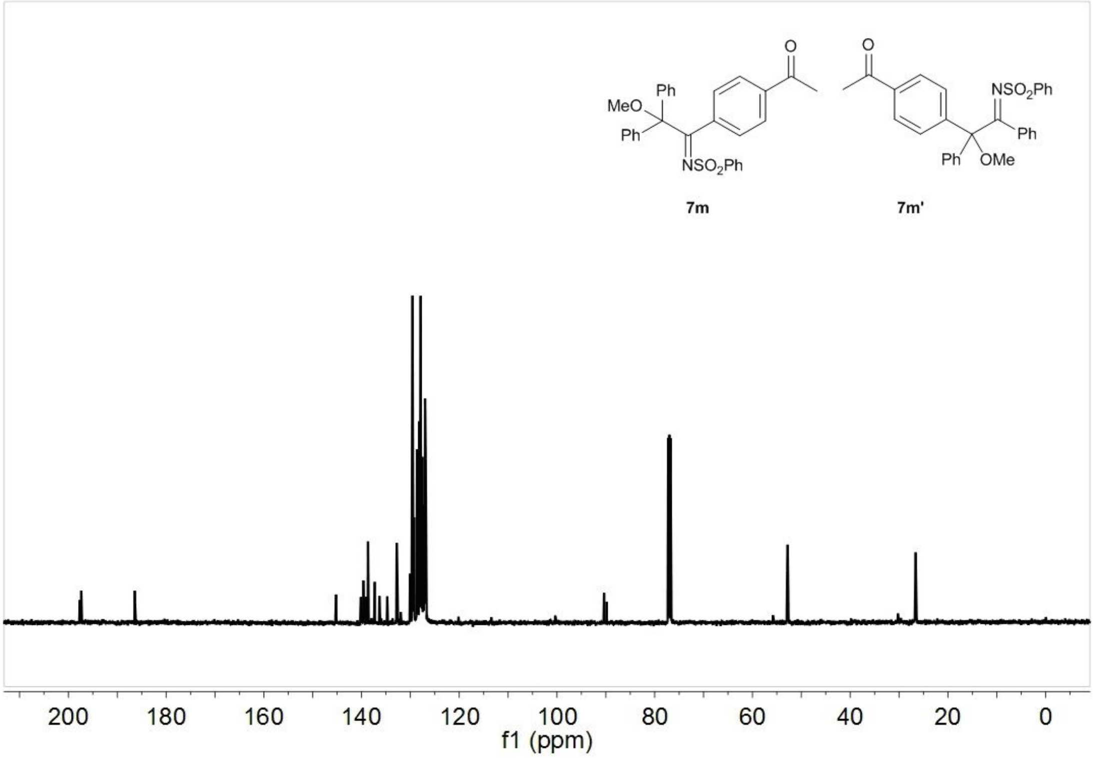

Supplementary Figure 114. ^{13}C NMR (500 MHz, $CDCl_3$) spectra for 7l and 7l'.

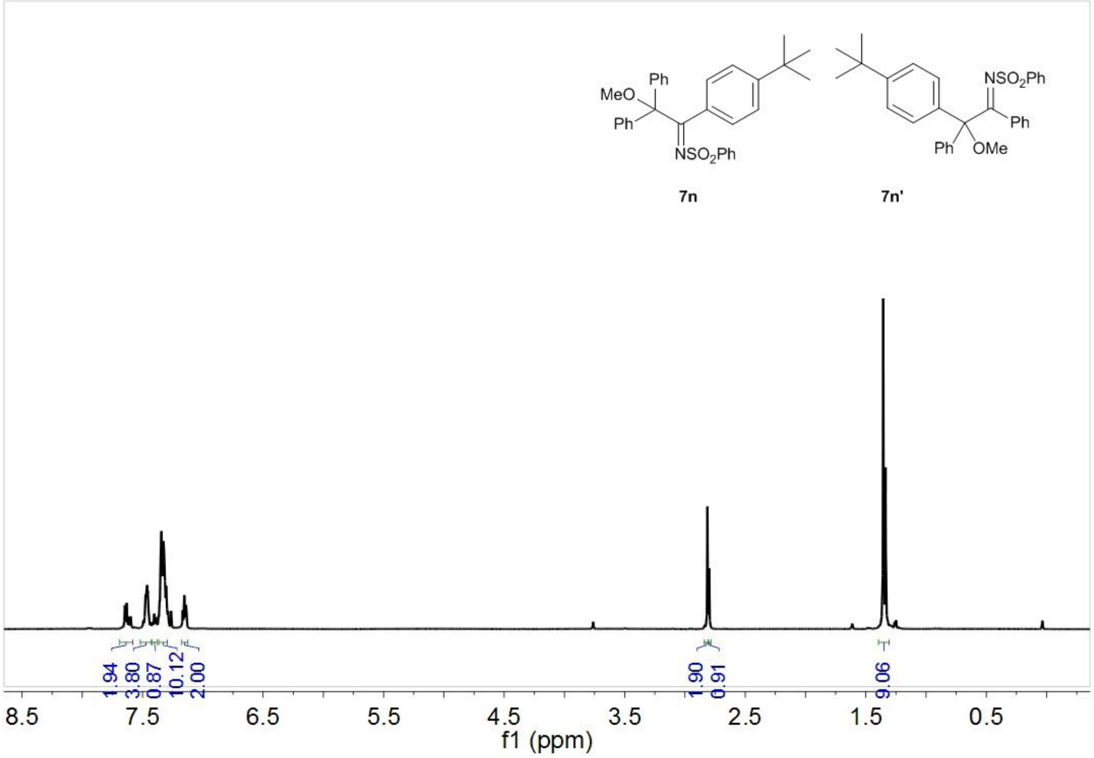

Supplementary Figure 115. ^{1}**H NMR (500 MHz, CDCl$_3$) spectra for 7m and 7m'.**

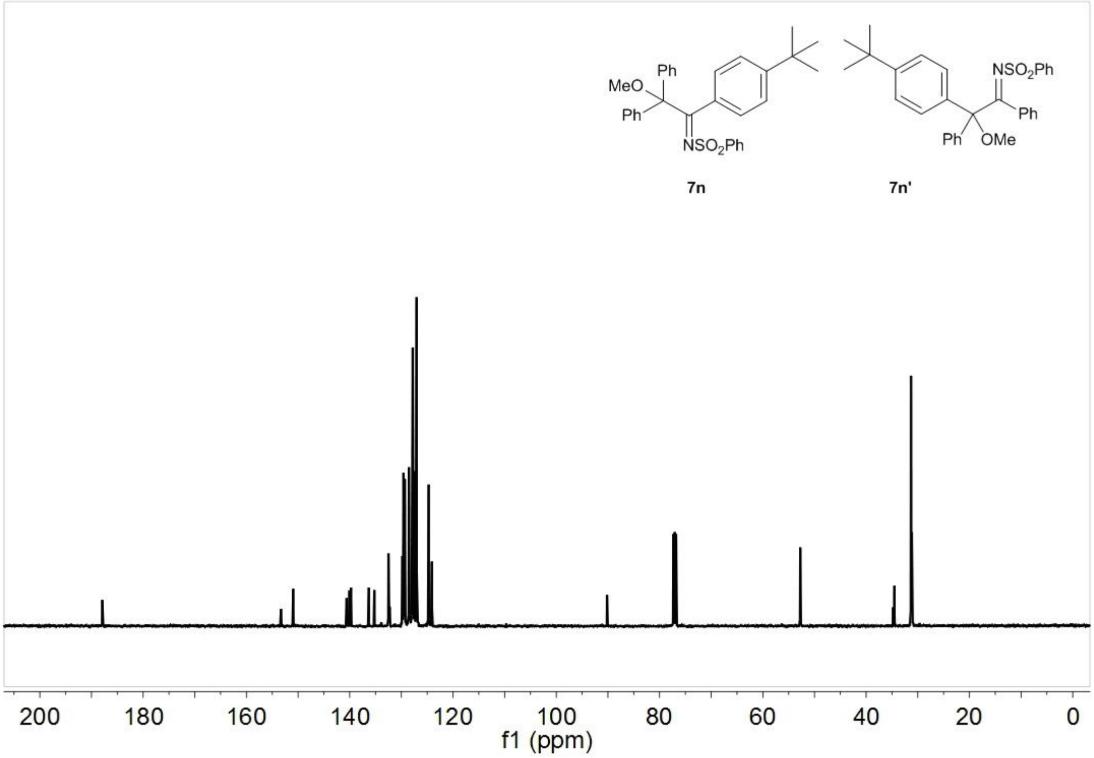

Supplementary Figure 116. ^{13}**C NMR (500 MHz, CDCl$_3$) spectra for 7m and 7m'.**

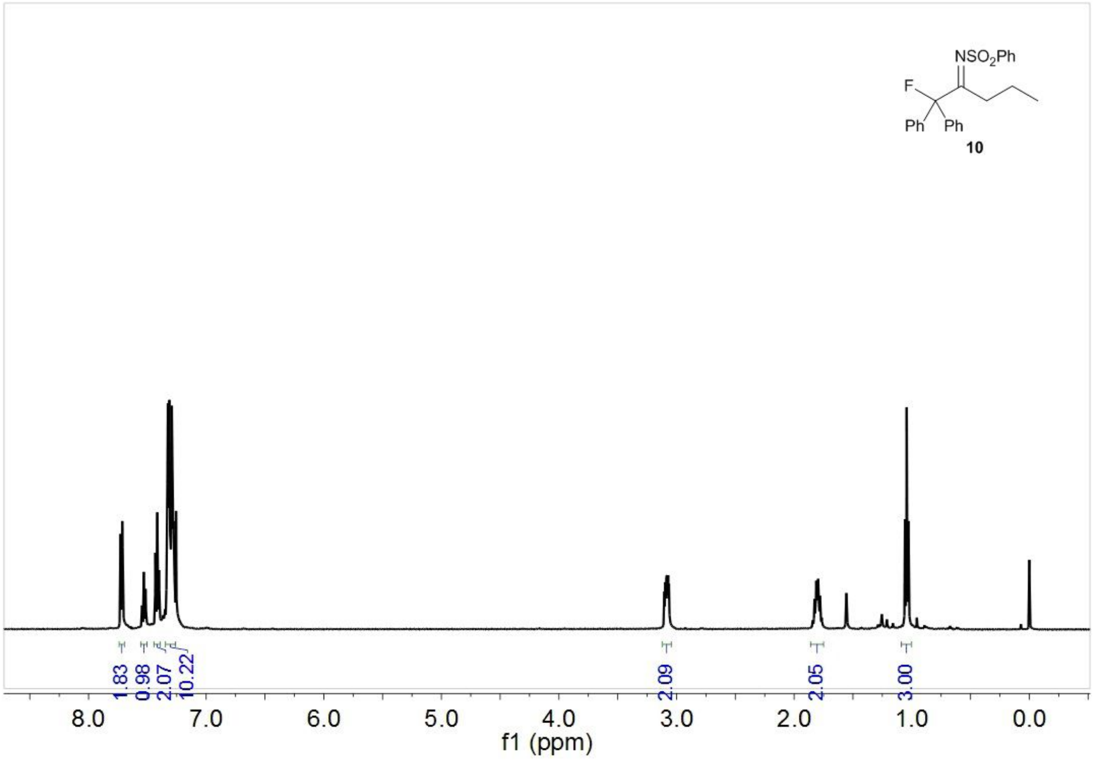

Supplementary Figure 117. ^{1}H NMR (500 MHz, $CDCl_3$) spectrum for 10.

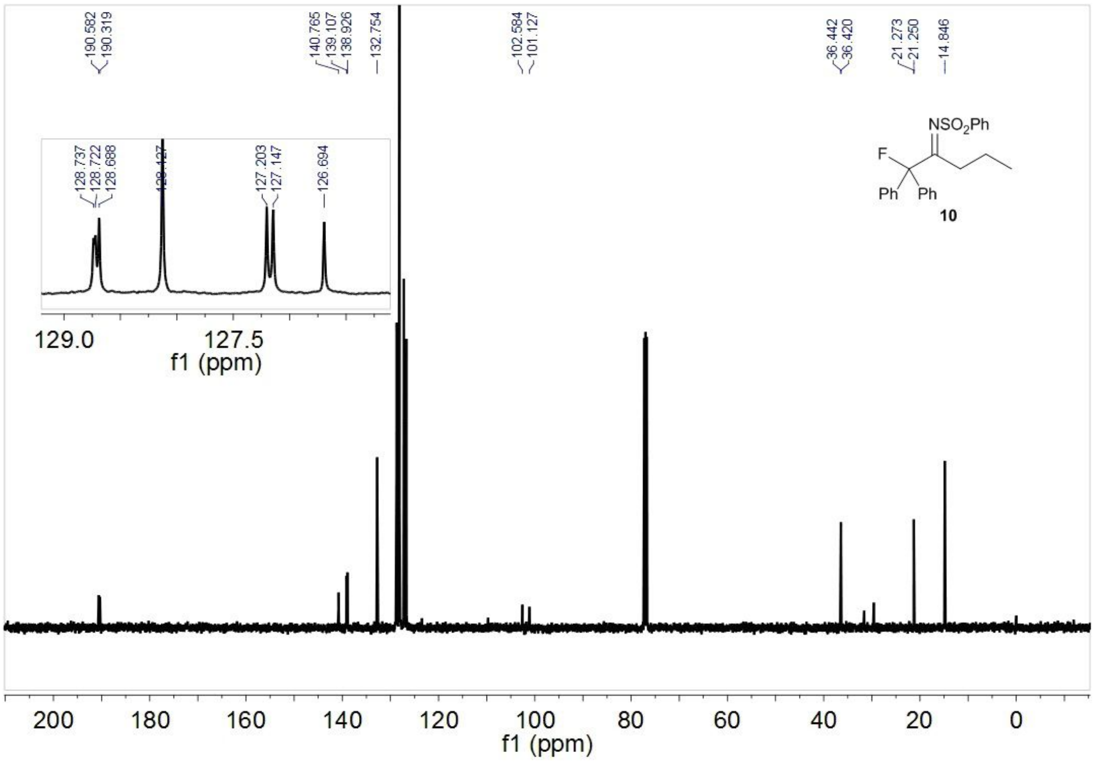

Supplementary Figure 118. ^{13}C NMR (125 MHz, $CDCl_3$) spectrum for 10.

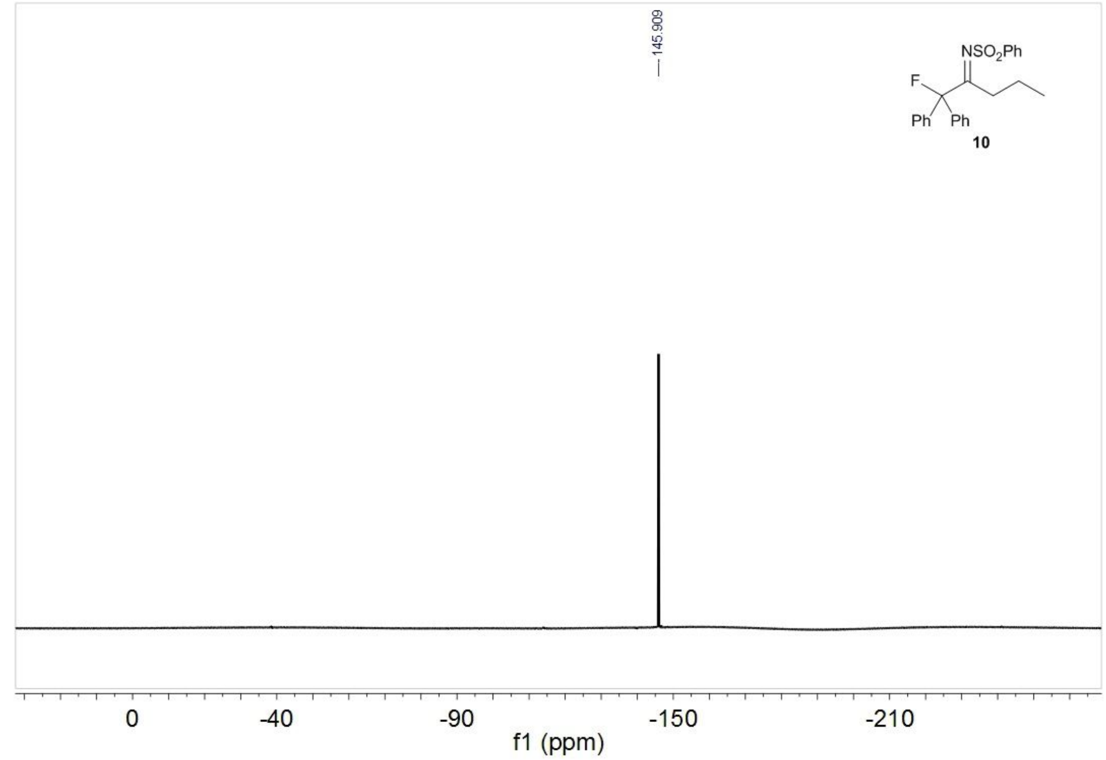

Supplementary Figure 119. ^{19}F NMR (500 MHz, CDCl$_3$) spectrum for 10.

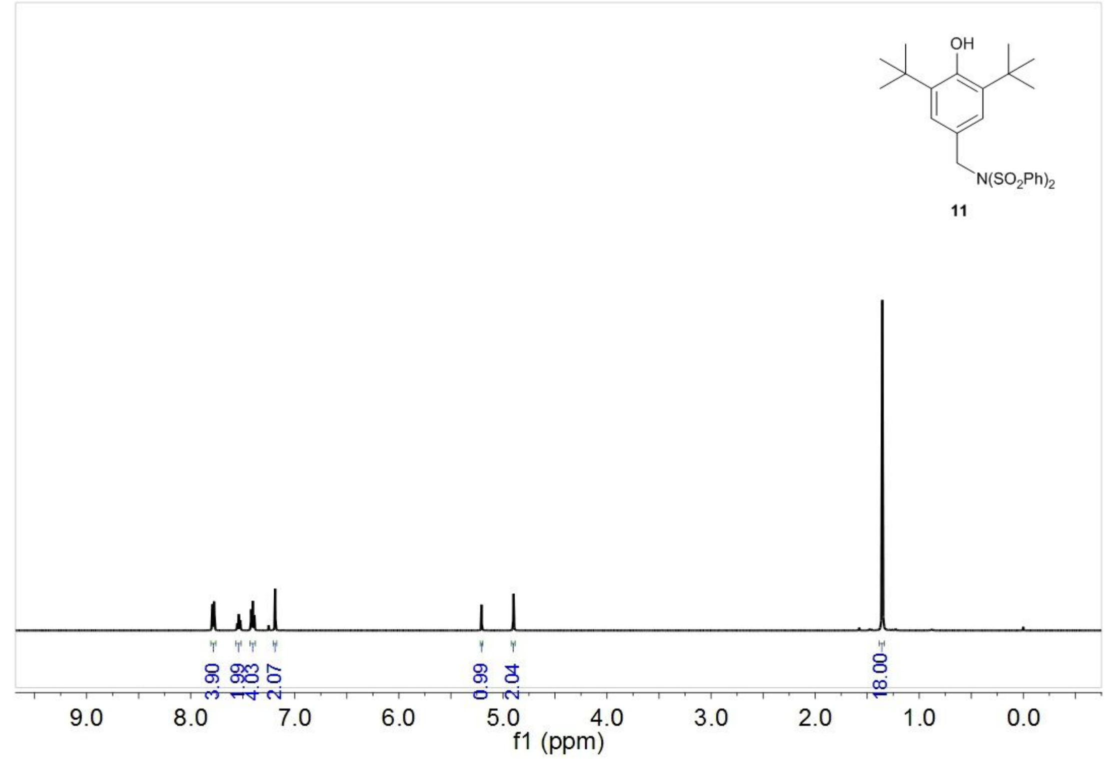

Supplementary Figure 120. ^{1}H NMR (500 MHz, CDCl$_3$) spectrum for 11.

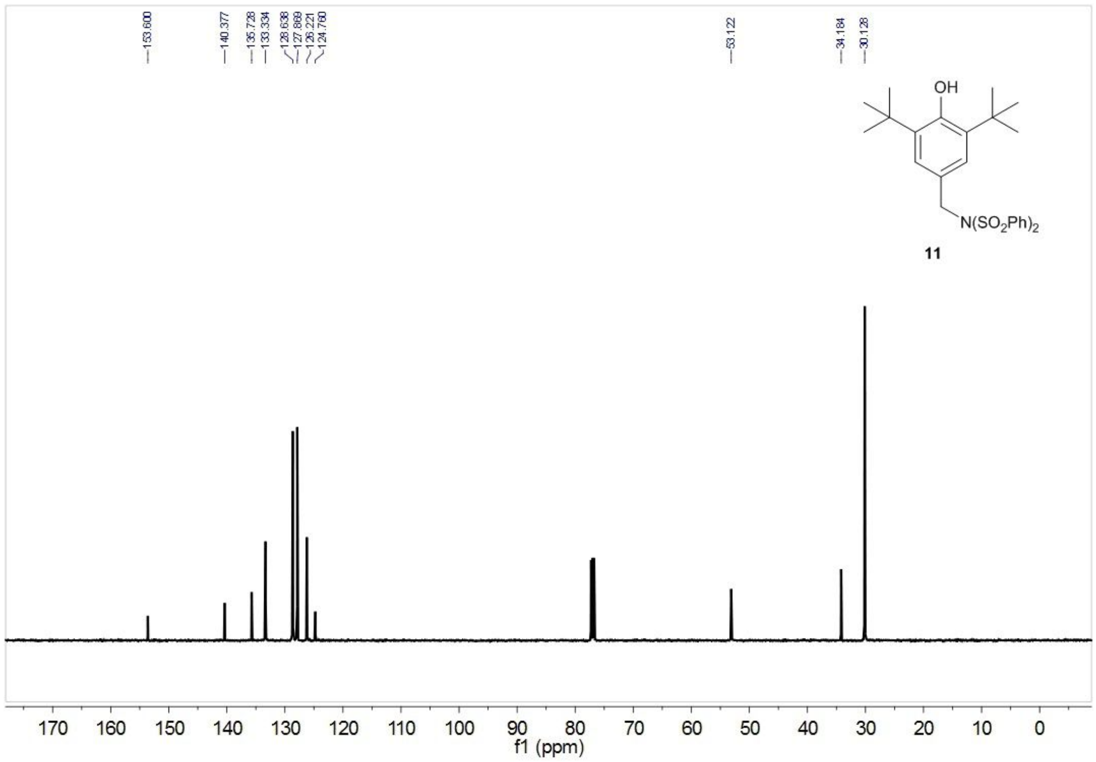

Supplementary Figure 121. ^{13}C NMR (125 MHz, CDCl$_3$) spectrum for 11.

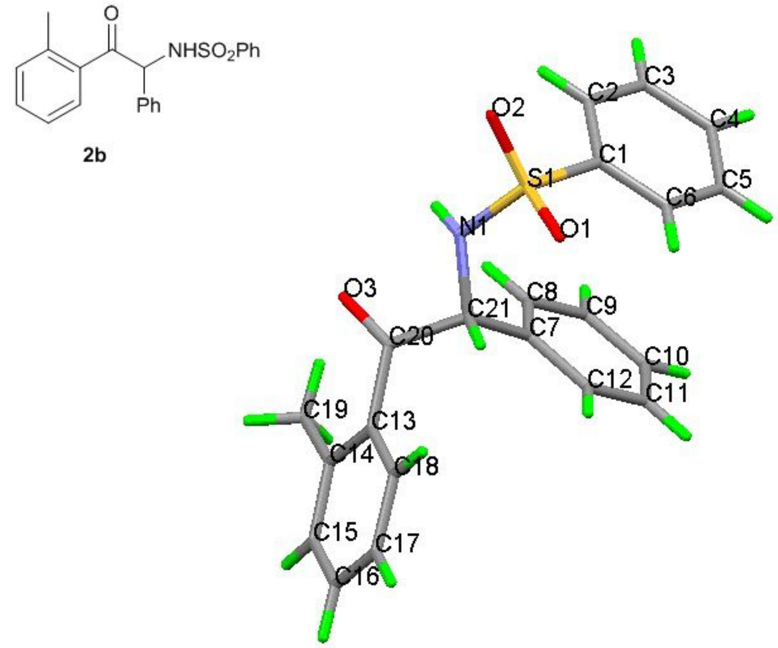

Supplementary Figure 122. X-Ray Crystallography of 2b

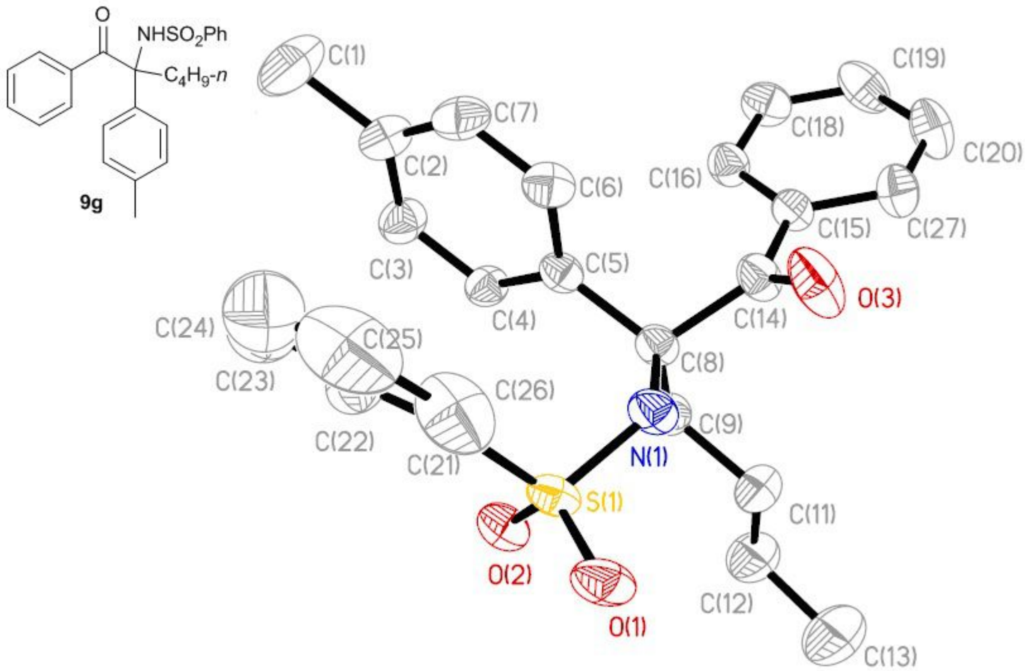

Supplementary Figure 123. X-Ray Crystallography of 9g

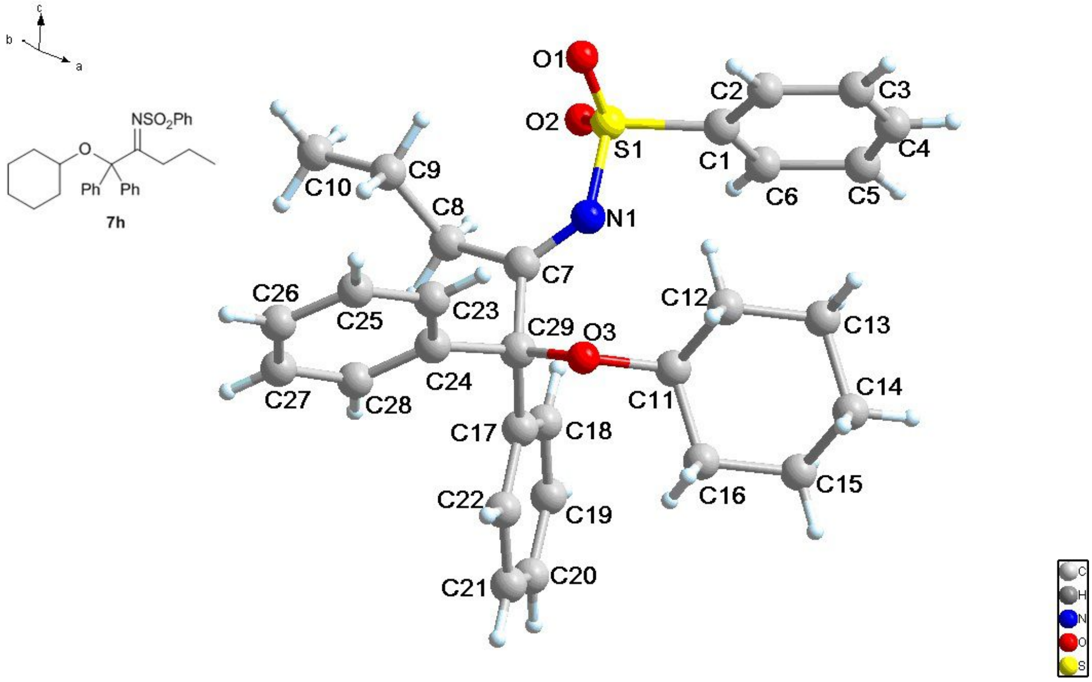

Supplementary Figure 124. X-Ray Crystallography of 7h.

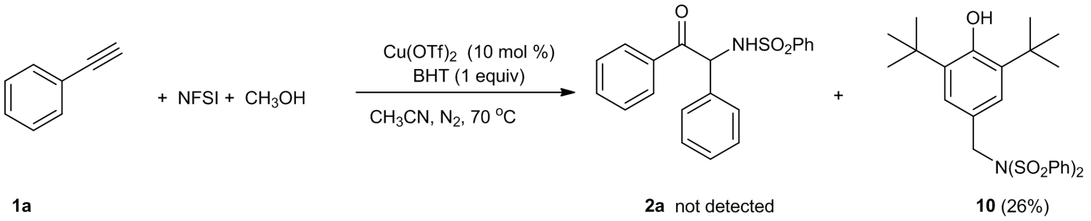

Supplementary Figure 125. BHT (2,6-di-*tert*-butyl-4-methylphenol) as radical scavenger.

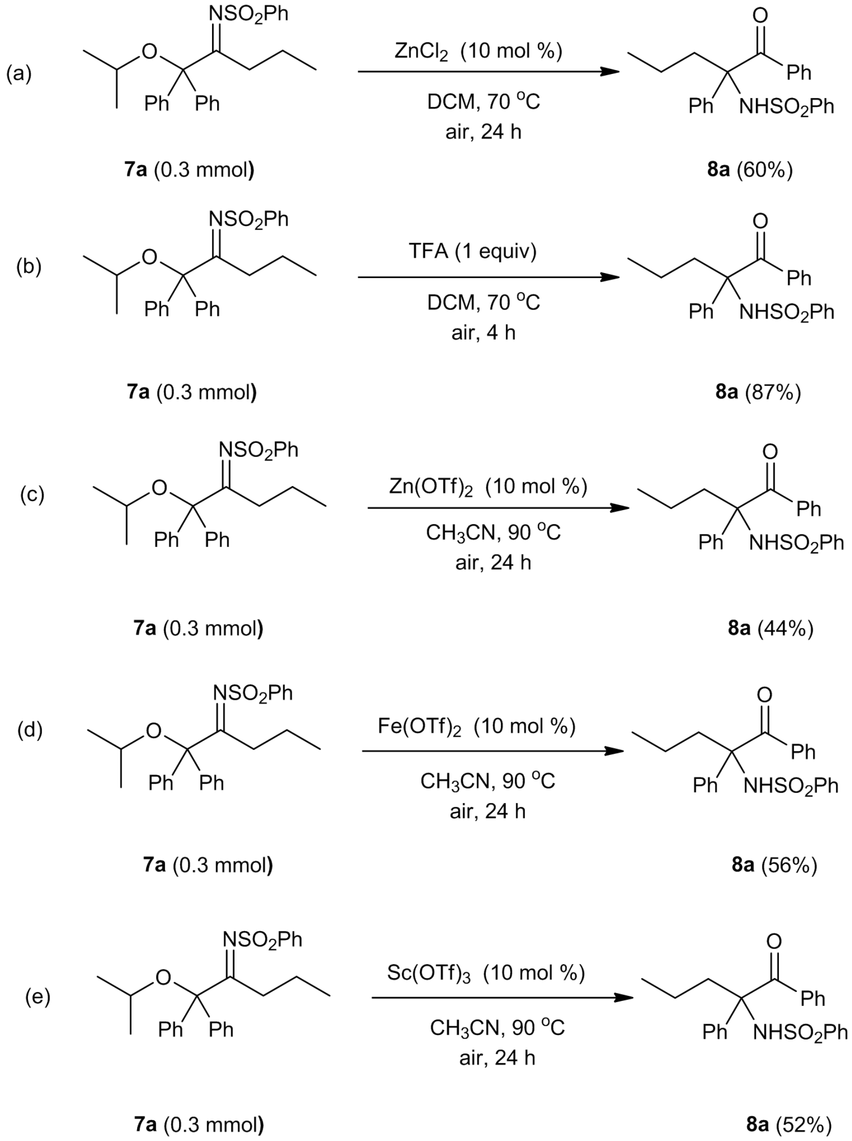

Supplementary Figure 126. Semi-pinacol rearrangement of **7a.**

Supplementary Tables

Supplementary Table 1. Optimization of aminative multifunctionalization of internal alkynes[a,b]

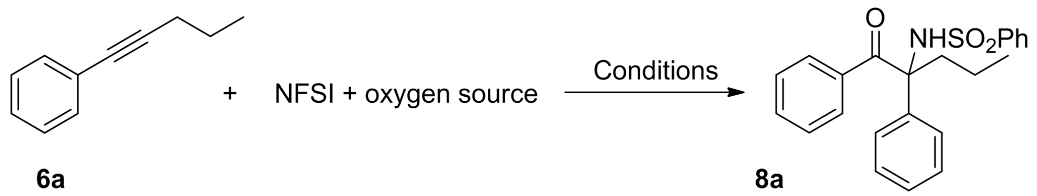

6a **8a**

entry	oxygen source	catalyst	additive (equiv)	solvent	yield (%)
1	CH_3OH	CuCN	None	DCM	38
2	EtOH	CuCN	None	DCM	40
3	*tert*-butanol	CuCN	None	DCM	28
4	isopropanol	CuCN	None	DCM	57
5	Pyridine-*N*-oxide	CuCN	None	DCM	0
6	CH_3COOH	CuCN	None	DCM	0
7	isopropanol	Cu_2Se	None	DCM	32
8	isopropanol	$Cu(OTf)_2$	None	DCM	38
9	isopropanol	$Cu(acac)_2$	None	DCM	41
10	isopropanol	AgOTf	None	DCM	33
11	isopropanol	$Fe(OTf)_2$	None	DCM	Trace
12	isopropanol	CuCN	None	DCE	46
13	isopropanol	CuCN	None	EtOAc	18
14	isopropanol	CuCN	None	CH_3CN	17
15	isopropanol	CuCN	None	THF	0
16	isopropanol	CuCN	None	acetone	Trace
17	isopropanol	CuCN	None	DMF	0
18	isopropanol	CuCN	BF_3•Et_2O (1)	DCM	32
19	isopropanol	CuCN	Na_2CO_3 (1)	DCM	NR[c]
20	isopropanol	CuCN	CH_3COOH (1)	DCM	35
21	isopropanol	CuCN	$Zn(OTf)_2$ (1)	DCM	22
22	isopropanol	CuCN	$ZnCl_2$ (0.1)	DCM	64
**23**	**isopropanol**	**CuCN**	**$ZnCl_2$ (0.02)**	**DCM**	**68**
23[d]	isopropanol	CuCN	$ZnCl_2$ (0.02)	DCM	60

[a]Reaction conditions: **6a** (0.5 mmol), NFSI (1.5 equiv, 0.75 mmol), oxygen source (1.5 equiv, 0.75 mmol), catalysts (5 mol %), additives, anhydrous solvents (2 mL), nitrogenous atmosphere, 12 h. [b]Yield of the isolated product. [c]NR = no reaction. [d]10 mol % of catalyst was used.

Supplementary Table 2. Optimization of α-alkyloxyl-α,α-diaryl imine Syntheses[a,b]

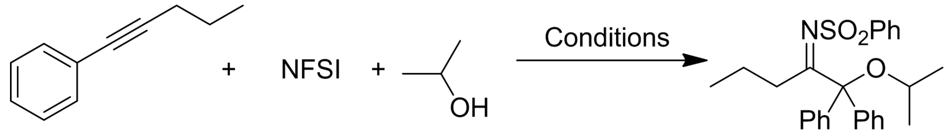

6a **7a**

entry	catalyst	additive (equiv)	solvent	T (°C)	yield (%)
1	CuTc	none	CH_3CN	25	32
2	CuCN	none	CH_3CN	25	19
3	Cu_2Se	none	CH_3CN	25	31
4	CuCl	none	CH_3CN	25	28
5	$[Cu(CH_3CN)_4]PF_6$	none	CH_3CN	25	35
6	$[Cu(CH_3CN)_4]BF_4$	none	CH_3CN	25	38
7	$CuCl_2$	none	CH_3CN	25	44
8	$Cu(OAc)_2$	none	CH_3CN	25	51
9	$Cu(OTf)_2$	none	CH_3CN	25	48
10	$Cu(acac)_2$	none	CH_3CN	25	55
11	$Cu(acac)_2$	none	DCM	25	NR[c]
12	$Cu(acac)_2$	none	NMP	25	0
13	$Cu(acac)_2$	none	THF	25	0
14	$Cu(acac)_2$	none	DMF	25	0
15	$Cu(acac)_2$	$ZnCl_2$ (0.1)	CH_3CN	25	47
16	$Cu(acac)_2$	$Zn(OTf)_2$ (0.1)	CH_3CN	25	50
17	$Cu(acac)_2$	$Sc(OTf)_3$ (0.1)	CH_3CN	25	32
18	$Cu(acac)_2$	TFA (0.1)	CH_3CN	25	51
19	$Cu(acac)_2$	TFA (2.0)	CH_3CN	25	34
20	$Cu(acac)_2$	Pyridine (2.0)	CH_3CN	25	27
21	$Cu(acac)_2$	BHT (2.0)	CH_3CN	25	Trace
22	$Cu(acac)_2$	TEMPO (2.0)	CH_3CN	25	Trace
24	$Cu(acac)_2$	none	CH_3CN	40	28
**24[d]**	**$Cu(acac)_2$**	**none**	**CH_3CN**	**0**	**71**
25[e]	$Cu(acac)_2$	none	CH_3CN	25	NR[c]
26	none	none	CH_3CN	25	NR[c]

[a]Reaction conditions: **6a** (0.5 mmol), NFSI (2 equiv, 1.0 mmol), oxygen source (3 equiv, 1.5 mmol), catalysts (5 mol %), additives, anhydrous solvents (2 mL), nitrogenous atmosphere, 24 h. [b]Yield of the isolated product. [c]NR = no reaction. [d]reaction performed for 48 h. [e] under air.

Supplementary Methods

General. All commercially available compounds were purchased from Aldrich or J&K Chemical Limited. All commercially supplied chemicals were used without further purification. ^{1}H NMR spectra were recorded at 25 ºC on a Varian 500 MHz, ^{13}C NMR spectra were recorded at 25 ºC on a Varian 125 MHz, and TMS as internal standard. The chemical shifts (δ) are given in parts per million relative to internal standard TMS (0 ppm for ^{1}H) and $CDCl_3$ (77.0 ppm for ^{13}C). High resolution mass spectra were recorded on Bruck microtof. Flash column chromatography was performed on silica gel 60 (particle size 300-400 mesh ASTM, purchased from Taizhou, China).

General procedure for preparation of alkynes.

Method A: Terminal alkynes **1c**, **1d**, **1n** were prepared according to the procedure reported by Krause[1].

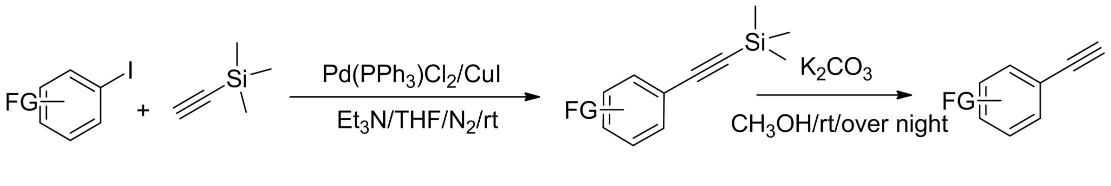

A **B** **C** **1**
(1c, 1d, 1n)

To a stirred mixture of 10 mmol of **A**, 76 mg (0.4 mmol) of CuI, and 140 mg (0.2 mmol) of $Pd(PPh_3)_2Cl_2$ in 15 mL of THF was added 2.5 g (15.0 mmol) of triethylamine. A solution of 1.2 g (12 mmol) of trimethylsilylacetylene **B** in 2 mL of THF was then added over 1 h. The solvent was evaporated, and the residue was treated with pentane. Filtration through Celite and evaporation of the solvent obtain analytically pure **C**. Then **C** was dissolved in 20 mL CH_3OH in 50 mL round flask, and 2.76 g (20 mmol) K_2CO_3 was added. The reaction mixture was stirred vigorously over night. The solvent was evaporated, and the residue was treated with pentane. Filtration through Celite and evaporation of the solvent, analytically pure **1** was obtained.

Method B: Internal alkynes **6e**, **6g**, **6h**, **6i**, **6j**, **6l, 6m, 6n** were prepared according to the procedure reported by Krause.[1]

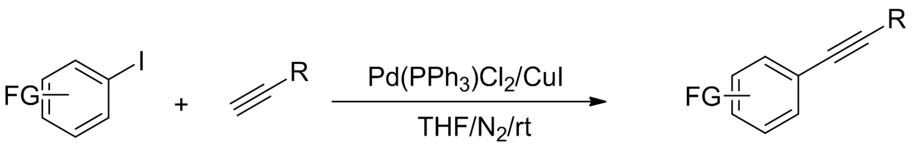

A **D** **6** (**6e, 6g, 6h, 6i, 6j, 6l, 6m, 6n**)

A mixture of 5.0 mmol of **A**, 175 mg (0.25 mmol) of $Pd(PPh_3)_2Cl_2$, 33 mg (0.125 mmol) of PPh_3, 7.5 mmol of **D**, and 1.01 g (7.5 mmol) of triethylamine in 20 mL of THF was stirred for 20 min at room temperature, and 12 mg (0.06 mmol) of CuI was then added. After being stirred for 16 h, the solvent was evaporated, and the residue was treated with pentane. Filtration through Celite and evaporation of the solvent, analytically pure **6** was obtained.

Method C: 6k was prepared according to the classic method.

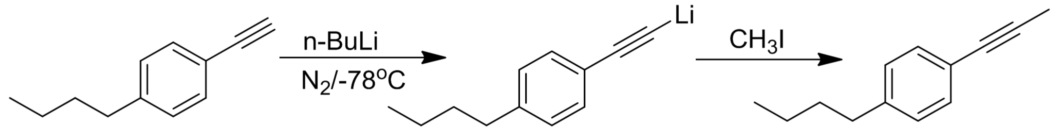

1 **6k**

Under N_2 atmosphere, compound **1** (10 mmol) was added into three-neck-bottle (50 mL) and mixed with THF (20 mL). The reaction system was cooled to -78 °C before *n*-BuLi (5 mL, 2.5 M, 12.5 mmol) was injected. After stirring for additional 2 hours at -78 °C, CH_3I (15 mmol) was added dropwise into the reaction system. The reaction system was stirred overnight before quenched by water (20 mL). Using DCM to extract, the organic layer was dried by anhydrous $MgSO_4$. After removing the solvent on vacuum, the residue was subjected to flash column chromatograph by using PE as eluent to give compound **6k.**

General procedure for preparation of NFSI derivatives. NFSI derivatives were prepared according to the procedure reported by He.[2]

General procedure for aminative multifunctionalization of terminal alkynes with NFSI. (take 2a as an example).

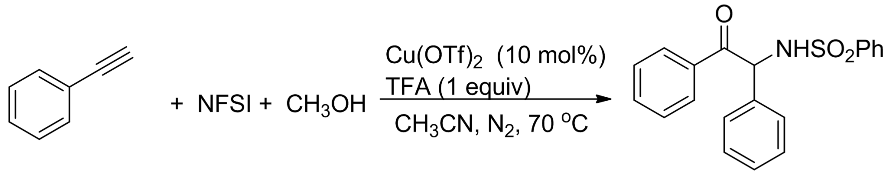

1a **2a**

To a solution of the NFSI (0.75 mmol, 236.5 mg) in CH_3CN (2.0 mL) was added the CH_3OH (1.5 mmol, 61 μL), TFA(0.5 mmol, 37 μL), 1-Phenylethyne (**1a**, 0.5 mmol, 54 μL) and $Cu(OTf)_2$ (0.05 mmol, 18.1 mg) in screw-cap test tube under N_2 atmosphere. The test tube was then sealed off with a screw-cap and the reaction mixture was stirred at 70 ℃ for 5.0 h. After the reaction finished, the reaction mixture was cooled to room temperature and quenched by water. The mixture was extracted with CH_2Cl_2 (3 × 5.0 mL), the combined organic phases were dried over anhydrous Na_2SO_4 and the solvent was evaporated under vacuum. The residue was purified by column chromatography (petroleum ether/ethyl acetate 10:1 (v/v)) to give the corresponding product **2a** (136.9 mg, 78%) .

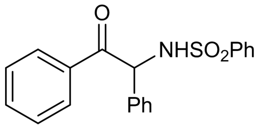

2a

***N*-(2-oxo-1,2-diphenylethyl)benzenesulfonamide 2a**

For **2a,** white solid, mp. 166–171 °C. NMR Spectroscopy: **^{1}H NMR** (500 MHz, $CDCl_3$): δ = 6.06 (d, *J* = 7.0 Hz, 1H), 6.32 (d, *J* = 7.5 Hz, 1H), 7.14–7.18 (m, 5H), 7.25 (t, *J* = 7.5 Hz, 2H), 7.34–7.39 (m, 3H), 7.49 (t, *J* = 7.5 Hz, 1H), 7.63 (d, *J* = 8.5 Hz, 2H), 7.80 (d, *J* = 8.5 Hz, 2H); **^{13}C**

NMR (125 MHz; $CDCl_3$): δ = 61.7, 126.8, 128.1, 128.5, 128.7, 128.9, 129.0, 132.3, 133.6, 134.0, 135.4, 140.4, 194.3. IR (in KBr): 3324, 3052, 2924, 1681, 1593, 1494, 1448, 1344, 1158, 752, 720, 706 cm^{-1}. Mass Spectrometry: HRMS (ESI-TOF) (m/z): Calcd for $C_{20}H_{17}NNaO_3S$ ($[M + Na]^+$), 374.0821, found, 374.0829.

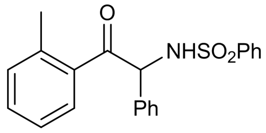

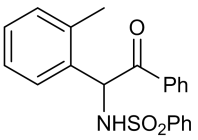

2b **3b**

***N*-(2-oxo-1-phenyl-2-(*o*-tolyl)ethyl)benzenesulfonamide 2b**

***N*-(2-oxo-2-phenyl-1-(*o*-tolyl)ethyl)benzenesulfonamide 3b**

The title compounds were prepared according to the synthesis of **2a** to afford **2b** and **3b** (56%). The products could not readily be separated by silica gel chromatography. The ratio of the isomor was determined by ^{1}H NMR Spectroscopy. The ^{1}H NMR spectrum of the product showed a 4:1 mixture of **2b** and a compound tentatively assigned as **3b** based on the methyl peak at δ 2.15 for **2b** and at δ 2.48 for **3b**. **2b** was further purified by recrystallization from petroleum ether/diethyl ether 3:1 (v/v).

For **2b**, white solid, mp. 168–172 °C. NMR Spectroscopy: **^{1}H NMR** (500 MHz; $CDCl_3$): δ = 2.15 (s, 3H), 5.87 (d, J = 7.0 Hz, 1H), 6.33 (d, J = 6.0 Hz, 1H), 7.04–7.05 (m , 2H), 7.09–7.12 (m, 4H), 7.17 (t, J= 7.5 Hz, 1H), 7.30 (t, J= 8.0 Hz, 3H), 7.39 (d, J = 7.5 Hz, 2H), 7.43 (d, J = 7.5 Hz, 2H), 7.65–7.68 (m , 2H); **^{13}C NMR** (125 MHz; $CDCl_3$): δ = 20.5, 63.5, 125.5, 126.8, 127.8, 128.1, 128.3, 128.7, 128.8, 131.8, 132.0, 132.3, 134.5, 134.6, 139.0, 140.2, 197.2. IR (in KBr): 3261, 3063, 3025, 2962, 1688, 1598, 1485, 1449, 1392, 1164, 752, 700 cm^{-1}. Mass Spectrometry: HRMS (ESI-TOF) (m/z): Calcd for $C_{21}H_{19}NNaO_3S$ ($[M + Na]^+$), 388.0978, found 388.0972.

For **2b** and **3b**, NMR Spectroscopy: **^{1}H NMR** (500 MHz; $CDCl_3$): δ = 2.15 (s, 3H), 2. 48 (s, 0.8H), 5.87 (d, J = 7.0 Hz, 1H), 7.09–7.12 (m, 0.5H), 6.33 (d, J = 6.0 Hz, 1H), 6.92 (d, J = 7.5 Hz, 0.3H), 6.97–6.99 (m, 0.3H), 7.04–7.05 (m, 2H), 7.09–7.12 (m, 4.5H), 7.17 (t, J= 7.5 Hz, 1H), 7.28–7.34 (m, 4.2H), 7.40–7.44 (m, 2.3H), 7.47 (t, J = 7.5 Hz, 0.3H), 7.65–7.68 (m , 3H); **^{13}C NMR** (125 MHz; $CDCl_3$): δ = 19.3, 20.5, 59.5, 63.5, 125.5, 126.6, 126.8, 127.8, 128.0, 128.1, 128.3, 128.4, 128.6, 128.7, 128.8, 131.7, 131.8, 132.0, 132.3, 132.4, 133.3, 133.7, 134.1, 134.5, 134.6, 136.5, 139.0, 140.0, 140.3, 195.2, 197.2.

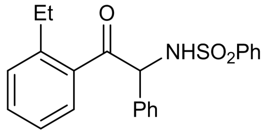

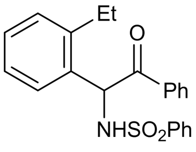

2c **3c**

***N*-(2-(2-ethylphenyl)-2-oxo-1-phenylethyl)benzenesulfonamide 2c**

***N*-(1-(2-ethylphenyl)-2-oxo-2-phenylethyl)benzenesulfonamide 3c**

The title compounds were prepared according to the synthesis of **2a** to afford **2c** and **3c** (56%). The ratio of the isomer was determined by ^{1}H NMR Spectroscopy. The ^{1}H NMR spectrum of the crude product showed a 3:1 mixture of **2c** and a compound tentatively assigned as **3c** based on the

methyl peak at δ 0.91 for **2c** and at δ 1.25 for **3c**. **2c** and **3c** was further purified by silica gel chromatography (petroleum ether/ diethyl ether 12:1 (v/v)).

For **2c**, white solid, mp. 165–167 °C. NMR Spectroscopy: **^{1}H NMR** (500 MHz; $CDCl_3$): δ = 0.91 (t, J = 7.5 Hz, 3H), 2.42 (q, J = 7.5 Hz, 2H), 5.85 (d, J = 6.5 Hz, 1H), 6.34 (d, J = 7.0 Hz, 1H), 7.02–7.04 (m, 2H), 7.09–7.11 (m, 3H), 7.14–7.18 (m, 2H), 7.31 (t, J = 8.0 Hz, 2H), 7.36 (t, J = 8.0 Hz, 2H), 7.42 (t, J = 7.5 Hz, 1H), 7.67 (d, J = 7.5 Hz, 2H); **^{13}C NMR** (125 MHz; $CDCl_3$): δ = 15.5, 26.3, 63.8, 125.4, 126.9, 127.7, 127.8, 128.2, 128.7, 128.8, 130.1, 131.9, 132.2, 134.2, 134.4, 140.3, 145.0, 197.5. IR (in KBr): 3282, 3064, 2963, 2928, 1689, 1598, 1485, 1448, 1330, 1164, 745, 689 cm^{-1}. Mass Spectrometry: HRMS (ESI-TOF) (m/z): Calcd for $C_{22}H_{22}NO_3S$ ([M + H]$^+$), 380.1315, found 380.1324.

For 3c, yellow liquid. NMR Spectroscopy: **^{1}H NMR** (500 MHz; $CDCl_3$): δ = 1.26 (t, J = 7.5 Hz, 3H), 2.83–2.89 (m, 2H), 5.96 (d, J = 6.5 Hz, 1H), 6.18 (d, J = 7.0 Hz, 1H), 6.93 (d, J = 7.5 Hz, 1H), 6.99–7.01 (m, 1H), 7.18–7.19 (m, 2H), 7.30–7.34 (m, 4H), 7.42–7.50 (m, 2H), 7.64 (d, J = 7.5 Hz, 2H), 7.68–7.70 (m, 2H); **^{13}C NMR** (125 MHz; $CDCl_3$): δ = 14.8, 24.6, 59.3, 126.4, 127.0, 128.1, 128.5, 128.6, 128.8, 129.1, 129.4, 132.5, 132.7, 133.7, 140.1, 142.4, 195.7. IR (in KBr): 3283, 3064, 2968, 2932, 2876, 1689, 1596, 1488, 1448, 1340, 1162, 753, 721, 689 cm^{-1}. Mass Spectrometry: HRMS (ESI-TOF) (m/z): Calcd for $C_{22}H_{22}NO_3S$ ([M + H]$^+$), 380.1315, found 380.1313.

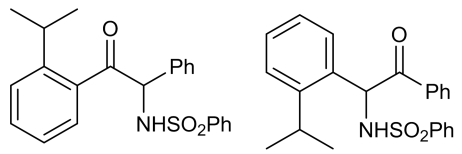

2d **3d**

***N*-(2-(2-isopropylphenyl)-2-oxo-1-phenylethyl)benzenesulfonamide 2d**

***N*-(1-(2-isopropylphenyl)-2-oxo-2-phenylethyl)benzenesulfonamide 3d**

The title compounds were prepared according to the synthesis of **2a** to afford **2d** and **3d** (58%). The products could not readily be separated by silica gel chromatography. The ratio of the isomer was determined by ^{1}H NMR Spectroscopy. The ^{1}H NMR spectrum of the product showed a 5:2 mixture of **2d** and a compound tentatively assigned as **3d** based on the methine peak at δ 2.58–2.64 for **2d** and at δ 3.37–3.42 for **3d**. **2d** was further purified by recrystallization from petroleum ether/diethyl ether 3:1 (v/v).

For **2d**, white solid, mp. 162–164 °C. NMR Spectroscopy: **^{1}H NMR** (500 MHz; $CDCl_3$): δ = 0.57 (d, J = 7.0 Hz, 3H), 1.13 (d, J = 6.5 Hz, 3H), 2.58–2.64 (m, 1H), 5.76 (d, J = 6.0 Hz, 1H), 6.42 (d, J = 6.0 Hz, 1H), 6.97 (d, J = 7.0 Hz, 2H), 7.05–7.10 (m, 3H), 7.12–7.18 (m, 2H), 7.22 (d, J = 8.0 Hz, 1H), 7.30–7.36 (m, 3H), 7.43 (t, J = 7.0 Hz, 1H), 7.70 (J = 8.0 Hz, 2H); **^{13}C NMR** (125 MHz; $CDCl_3$): δ = 23.4, 24.4, 29.5, 64.8, 125.2, 126.4, 126.5, 126.9, 127.9, 128.3, 128.6, 128.7, 131.6, 132.4, 133.8, 135.4, 140.2, 148.4, 198.6. IR (in KBr): 3298, 3064, 3027, 3003, 2963, 2926, 2867, 1691, 1341, 1163, 752, 701 cm^{-1}. Mass Spectrometry: HRMS (ESI-TOF) (m/z): Calcd for $C_{23}H_{24}NO_3S$ ([M + H]$^+$), 394.1471, found 394.1467.

For **2d** and **3d**, NMR Spectroscopy: **^{1}H NMR** (500 MHz; $CDCl_3$): δ = 0.57 (d, J = 7.0 Hz, 3H), 1.13–1.16 (m, 4.3H), 1.20 (d, J = 7.0 Hz, 3H), 2.58–2.64 (m, 1H), 3.37–3.42 (m, 0.4H), 5.76 (d, J = 6.0 Hz, 1H), 6.00 (d, J = 6.0 Hz, 0.4H), 6.26 (d, J = 6.0 Hz, 0.4H), 6.42 (d, J = 6.0 Hz, 1H), 6.97

(d, J = 7.0 Hz, 2.5H), 7.01 (t, J = 7.0 Hz, 0.4H), 7.05–7.10 (m, 3H), 7.14–7.17 (m, 2H), 7.22–7.25 (m, 1.5H), 7.27–7.29 (m, 0.5H), 7.30–7.36 (m, 5H), 7.42–7.45 (m, 2H), 7.65–7.72 (m, 3.5H); **^{13}C NMR** (125 MHz; $CDCl_3$): δ = 23.3, 23.7, 24.1, 24.3, 28.3, 29.4, 59.2, 64.7, 125.1, 126.1, 126.4, 126.7, 126.8, 126.9, 127.8, 128.1, 128.2, 128.4, 128.5, 128.6, 128.7, 128.8, 129.1, 131.5, 131.8, 132.3, 132.4, 133.5, 133.7, 134.3, 135.3, 140.1, 147.3, 148.2, 195.9, 198.5.

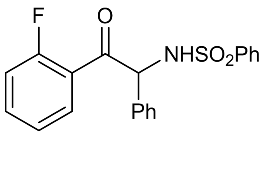

2e

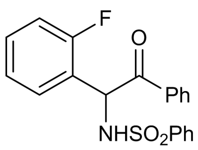

3e

***N*-(2-(2-fluorophenyl)-2-oxo-1-phenylethyl)benzenesulfonamide 2e**

***N*-(1-(2-fluorophenyl)-2-oxo-2-phenylethyl)benzenesulfonamide 3e**

The title compounds were prepared according to the synthesis of **2a** to afford **2e** and **3e** (61%). The products could not readily be separated by silica gel chromatography, so they were characterized as a mixture. The ratio of the isomer was determined by ^{1}H NMR Spectroscopy. The ^{1}H NMR spectrum of the product showed a 7:1 mixture of **2e** and a compound tentatively assigned as **3e** based on the imine peak at δ 5.99 for **2e** and at δ 6.36 for **3e**.

For **2e** and **3e**, NMR Spectroscopy: **^{1}H NMR** (500 MHz; $CDCl_3$): δ = 5.99 (d, J = 7.5 Hz, 1.00H), 6.28 (d, J = 7.5 Hz, 1.13H), 6.36 (d, J = 7.0 Hz, 0.14H), 6.86–6.93 (m, 0.27H), 7.00–7.03 (m, 1H), 7.07 (t, J = 7.0 Hz, 0.41H), 7.10–7.16 (m, 6.22H), 7.28–7.32 (m, 2.31H), 7.37–7.42 (m, 1.37H), 7.43–7.47 (m, 1.00H), 7.50 (t, J = 7.5 Hz, 0.15H), 7.65–7.70 (m, 3.25H), 7.85 (d, J = 7.5 Hz, 0.32H); **^{13}C NMR** (125MHz; $CDCl_3$): δ = 64.9 (d, J = 9 Hz), 116.7 (d, J = 24 Hz), 124.7 (d, J = 3 Hz), 126.9, 128.2, 128.6, 128.7, 128.9, 131.2 (d, J = 2 Hz), 132.4, 134.5, 135.7 (d, J = 9 Hz), 140.2, 160.1 (d, J = 254 Hz), 192.9 (d, J = 4 Hz). Mass Spectrometry: HRMS (ESI-TOF) (m/z): Calcd for $C_{20}H_{17}FNO_3S$, ([M + H]$^+$), 370.0908, found 370.0914.

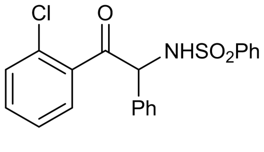

2f

***N*-(2-(2-chlorophenyl)-2-oxo-1-phenylethyl)benzenesulfonamide 2f**

The title compound was prepared according to the synthesis of **2a** to afford **2f** (52%).

For **2f**, white solid, mp. 136–139 °C. NMR Spectroscopy: **^{1}H NMR** (500 MHz; $CDCl_3$): δ = 5.96 (d, J = 6.5Hz, 1H), 6.42 (d, J = 6.5 Hz, 1H), 7.04 (d, J = 7.0 Hz, 2H), 7.07–7.14 (m, 5H), 7.26 (s, 2H), 7.32 (d, J = 8.0 Hz, 2H), 7.43 (t, J = 7.5 Hz, 1H), 7.71 (t, J = 7.5 Hz, 2H); **^{13}C NMR** (125 MHz; $CDCl_3$): δ = 64.9, 126.6, 127.0, 128.1, 128.6, 128.7, 128.8, 129.4, 130.5, 131.1, 132.3, 132.5, 133.9, 135.6, 140.2, 196.5. IR (in KBr): 3284, 3070, 3027, 2953, 1703, 1386, 1163, 1090, 769, 754, 738, 697 cm^{-1}. Mass Spectrometry: HRMS (ESI-TOF) (m/z): Calcd for $C_{20}H_{17}ClNO_3S$, ([M + H]$^+$), 386.0612, found. 386.0620.

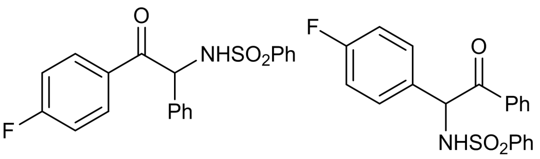

2g **3g**

***N*-(2-(4-fluorophenyl)-2-oxo-1-phenylethyl)benzenesulfonamide 2g**
***N*-(1-(4-fluorophenyl)-2-oxo-2-phenylethyl)benzenesulfonamide 3g**

The title compounds were prepared according to the synthesis of **2a** to afford **2g** and **3g** (55%). The products could not readily be separated by silica gel chromatography, so they were characterized as a mixture. The ratio of the isomer was determined by ^{1}H NMR Spectroscopy. The ^{1}H NMR spectrum of the product showed a 5:4 mixture of compounds tentatively assigned as **2g** and **3g** based on the methine peak at δ 6.30 for **2g** and at δ 6.00 for **3g**.

For **2g** and **3g**, NMR Spectroscopy: ^{1}H NMR (500 MHz; $CDCl_3$): δ = 6.00 (d, J = 7.0 Hz, 0.8H), 6.04 (d, J = 6.5 Hz, 1H), 6.31 (d, J = 7.5 Hz, 0.8H), 6.35 (d, J = 7.0 Hz, 1H), 6.83 (t, J = 9.5 Hz, 2H), 7.03 (t, J = 8.5 Hz, 1.6H), 7.13–7.16 (m, 6.3H), 7.26–7.30 (m, 4H), 7.35–7.41 (m, 3.8H), 7.51 (t, J = 7.0 Hz, 1.2H), 7.64–7.64 (m, 3.7H), 7.79 (d, J = 7.5 Hz ,2H), 7.84–7.87 (m, 1.6H); ^{13}C NMR (125 MHz; $CDCl_3$): δ = 60.9, 61.7, 115.9 (d, J = 22 Hz), 116.0 (d, J = 22 Hz), 126.8, 128.0, 128.6, 128.7 (d, J = 5 Hz), 128.9, 129.1, 129.4, 129.9 (d, J = 8 Hz), 130.1, 131.3 (d, J = 3 Hz), 131.7 (d, J = 10 Hz), 132.2, 132.3, 133.5, 134.0, 135.2, 140.4, 162.5 (d, J = 253 Hz), 165.9 (d, J = 255 Hz), 192.8, 194.2. Mass Spectrometry: HRMS (ESI-TOF) (m/z): Calcd for $C_{20}H_{16}FNNaO_3S$, ($[M + Na]^+$), 392.0727, found 392.0725.

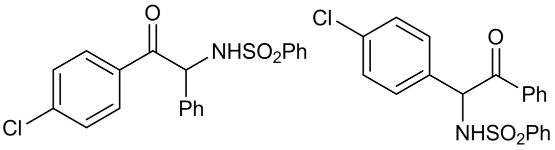

2h **3h**

***N*-(2-(4-chlorophenyl)-2-oxo-1-phenylethyl)benzenesulfonamide 2h**
***N*-(1-(4-chlorophenyl)-2-oxo-2-phenylethyl)benzenesulfonamide 3h**

The title compounds were prepared according to the synthesis of **2a** to afford **2h** and **3h** (62%). The products could not readily be separated by silica gel chromatography, so they were characterized as a mixture. The ratio of the isomer was determined by ^{1}H NMR Spectroscopy. The ^{1}H NMR spectrum of the product showed a 2:1 mixture of compounds tentatively assigned as **2h** and **3h** based on the imine peak at δ 6.33 for **2h** and at δ 6.40 for **3h**.

For **2h** and **3h**, NMR Spectroscopy: **^{1}H NMR** (500 MHz; $CDCl_3$): δ = 5.98 (d, J = 7.0 Hz, 1H), 6.03 (d, J= 7.0 Hz, 0.5H), 6.27 (d, J= 7.0 Hz, 1H), 6.42 (d, J = 7.0 Hz, 0.5H), 7.08–7.12 (m, 2H), 7.15–7.17 (m, 5H), 7.22–7.26 (m, 2.4H), 7.30 (d, J = 8.5 Hz, 2H), 7.33–7.41 (m, 2.7H), 7.50 (t, J = 7.5 Hz, 0.5H), 7.62 (d, J= 7.5 Hz, 3H), 7.75 (d, J = 8.5 Hz, 2H). 7.79 (d, J= 7.0 Hz, 1H); **^{13}C NMR** (125 MHz; $CDCl_3$): δ = 60.9, 61.8, 126.8, 128.0, 128.6, 128.7, 128.8, 128.9, 129.0, 129.1, 129.2, 129.4, 130.3, 131.9, 132.2 132.3, 133.4, 134.0, 134.2, 134.5, 135.0, 140.2, 140.3, 140.6, 193.1, 193.3. Mass Spectrometry: HRMS (ESI-TOF) (m/z): Calcd for $C_{20}H_{17}ClNO_3S$, ($[M + H]^+$), 386.0612, found 386.0620.

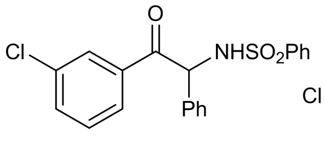

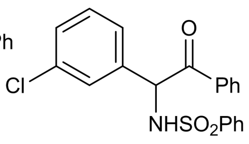

2i **3i**

***N*-(2-(3-chlorophenyl)-2-oxo-1-phenylethyl)benzenesulfonamide 2i**

***N*-(1-(3-chlorophenyl)-2-oxo-2-phenylethyl)benzenesulfonamide 3i**

The title compounds were prepared according to the synthesis of **2a** to afford **2i** and **3i** (67%). The ratio of the isomer was determined by ^{1}H NMR Spectroscopy. The ^{1}H NMR spectrum of the product showed a 9:1 mixture of **2i** and a compound tentatively assigned as **3i** based on the imine peak at δ 6.23 for **2i** and at δ 6.31 for **3i**. **2i** was futher purified by recrystallization from petroleum ether/diethyl ether 3:1 (v/v).

For **2i**, white solid, mp. 183–185 °C. NMR Spectroscopy: **^{1}H NMR** (500 MHz; $CDCl_3$): δ = 5.98 (d, J = 7.0 Hz, 1H), 6.24 (d, J = 7.0 Hz, 1H), 7.13–7.18 (m, 5H), 7.26–7.32 (m, 3H), 7.39 (t, J = 7.5 Hz, 1H), 7.45–7.47 (m, 1H), 7.62–7.64 (m, 2H) 7.67 (d, J = 7.5 Hz, 1H), 7.77 (t, J = 7.0 Hz, 1H); **^{13}C NMR** (125 MHz; $CDCl_3$): δ = 61.9, 126.8, 126.9, 128.0, 128.7, 128.8, 128.9, 129.2, 130.0, 132.3, 133.9, 134.8, 135.1, 135.2, 140.3, 193.3. IR (in KBr): 3256, 3078, 2950, 1692, 1492, 1333, 1168, 1091, 878, 755, 702 cm^{-1}. Mass Spectrometry: HRMS (ESI-TOF) (m/z): Calcd for $C_{20}H_{17}ClNO_3S$, ([M + H]$^{+}$), 386.0612, found 386.0615.

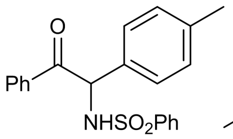

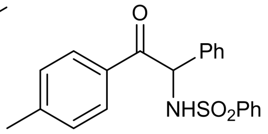

3j **2j**

***N*-(2-oxo-2-phenyl-1-(*p*-tolyl)ethyl)benzenesulfonamide 3j**

***N*-(2-oxo-1-phenyl-2-(*p*-tolyl)ethyl)benzenesulfonamide 2j**

The title compounds were prepared according to the synthesis of **2a** to afford **2j** and **3j** (58%). The ratio of the isomer was determined by ^{1}H NMR Spectroscopy. The ^{1}H NMR spectrum of the crude product showed a 9:1 mixture of **3j** and compound tentatively assigned as **2j** based on the methyl peak at δ 2.33 for **2j** and at δ 2.22 for **3j**.

For **3j**, white solid, mp. 149–152 °C. NMR Spectroscopy: **^{1}H NMR** (500 MHz; $CDCl_3$): δ = 2.22 (s, 3H), 5.98 (d, J = 7.5 Hz, 1H), 6.25 (d, J = 7.5 Hz, 1H), 6.95 (d, J = 7.5 Hz, 2H), 7.05 (d, J = 8.0 Hz, 2H), 7.26 (t, J = 7.5 Hz, 2H), 7.34–7.40 (m, 3H), 7.47 (t, J = 7.5 Hz, 1H), 7.63 (d, J = 7.5 Hz, 2H), 7.80 (d, J = 7.5 Hz, 2H); **^{13}C NMR** (125 MHz; $CDCl_3$): δ = 20.9, 61.5, 126.8, 127.9, 128.5, 128.6, 128.8, 129.6, 132.1, 132.4, 133.8, 138.3, 140.4, 194.3. IR (in KBr): 3267, 3059, 2918, 1676, 1345, 1166, 843, 753, 690 cm^{-1}. Mass Spectrometry: HRMS (ESI-TOF) (m/z): Calcd for $C_{21}H_{19}NNaO_3S$, ([M + Na]$^{+}$), 388.0978, found 388.0972.

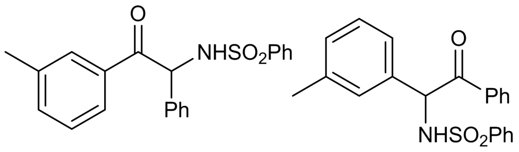

2k **3k**

***N*-(2-oxo-1-phenyl-2-(*m*-tolyl)ethyl)benzenesulfonamide 2k**

***N*-(2-oxo-2-phenyl-1-(*m*-tolyl)ethyl)benzenesulfonamide 3k**

The title compounds were prepared according to the synthesis of **2a** to afford **2k** and **3k** (53%). The products could not readily be separated by silica gel chromatography, so they were characterized as a mixture. The ratio of the isomer was determined by ^{1}H NMR Spectroscopy. The ^{1}H NMR spectrum of the product showed a 5:2 mixture of compounds tentatively assigned as **3k** and **2k** based on the methyl peak at δ 2.31 for **2k** and at δ 2.16 for **3k**.

For **2k** and **3k**, NMR Spectroscopy: **^{1}H NMR** (500 MHz; $CDCl_3$): δ = 2.17 (s, 3H), δ = 2.31 (s, 1.2H), 5.99 (d, J = 7.5 Hz, 1H), 6.02 (d, J = 7.5 Hz, 0.4H), 6.24 (d, J = 7.5Hz, 1H), 6.28 (d, J = 7.5Hz, 0.4H), 6.92 (s, 1H), 6.94 (d, J = 7.5 Hz, 1H), 6.98 (d, J = 7.5 Hz, 1H), 7.05 (t, J = 7.5 Hz, 1H), 7.14–7.19 (m, 2.0H), 7.21–7.25 (m, 2.3H), 7.27–7.31 (m, 1.3H), 7.34–7.38 (m, 3.4H), 7.49 (t, J = 7.5 Hz, 1H), 7.59 (d, J = 8.0 Hz, 0.5H), 7.62 (d, J = 7.5 Hz, 3H), 7.81–7.83 (m, 2H); **^{13}C NMR** (125 MHz; $CDCl_3$): δ = 21.1, 21.2, 61.7, 61.8, 125.3, 126.2, 126.8, 126.9, 128.1, 128.4, 128.5, 128.5, 128.6, 128.7, 128.9, 129.0, 129.3, 129.4, 132.2, 132.3, 133.8, 133.9, 134.8, 134.9, 135.5, 138.6, 138.9, 140.5, 194.3, 194.5. Mass Spectrometry: HRMS (ESI-TOF) (m/z): Calcd for $C_{21}H_{19}NNaO_3S$, ([M + Na]$^+$), 388.0978, found 388.0979.

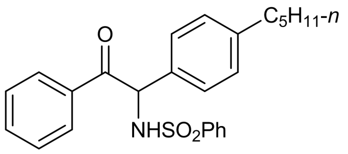

3l

***N*-(2-oxo-1-(4-pentylphenyl)-2-phenylethyl)benzenesulfonamide 3l**

The title compound was prepared according to the synthesis of **2a** to afford **3l** (52%).

For **3l**, white solid, mp. 156–158 °C. NMR Spectroscopy: **^{1}H NMR** (500 MHz; $CDCl_3$): δ = 0.88 (t, J = 7.0 Hz ,3H), 1.24–1.33 (m, 4H), 1.48–1.52 (m, 2H), 2.45 (t, J = 8.0 Hz, 2H), 6.00 (d, J = 7.5 Hz, 1H), 6.24 (d, J = 7.0 Hz, 1H), 6.95 (d, J = 7.5 Hz, 2H), 7.06 (d, J = 7.5 Hz, 2H), 7.23–7.26 (m, 2H), 7.36–7.37 (m, 3H), 7.49 (t, J = 7.5 Hz, 1H), 7.62 (d, J = 7.5 Hz, 2H), 7.81 (d, J = 7.5 Hz, 2H); **^{13}C NMR** (125 MHz; $CDCl_3$): δ = 13.9, 22.4, 30.9, 31.4, 35.4, 61.5, 126.9, 128.0, 128.6, 128.7, 128.9, 129.0, 132.1, 132.5, 133.8, 140.6, 143.5, 194.4. IR (in KBr): 3312, 3030, 2922, 2852, 1676, 1339, 1158, 829, 752, 685 cm^{-1}. Mass Spectrometry: HRMS (ESI-TOF) (m/z): Calcd for $C_{25}H_{28}NO_3S$, ([M + H]$^+$), 422.1784, found 422.1792.

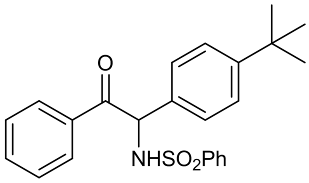

3m

***N*-(1-(4-(*tert*-butyl)phenyl)-2-oxo-2-phenylethyl)benzenesulfonamide 3m**

The title compound was prepared according to the synthesis of **2a** to afford **3m** (71%).

For **3m,** white solid, mp. 182–184 °C. NMR Spectroscopy: **^{1}H NMR** (500 MHz; $CDCl_3$): δ = 1.20 (s, 9H), 6.03 (d, J = 7.5 Hz, 1H), 6.24 (d, J = 7.0 Hz, 1H), 7.07 (d, J = 8.0 Hz, 2H), 7.14 (d, J = 8.0 Hz, 2H), 7.22 (t, J = 7.5 Hz, 2H), 7.33–7.38 (m, 3H), 7.50 (t, J = 7.5 Hz, 1H), 7.60 (d, J = 8.0 Hz, 2H), 7.83 (d, J = 7.5 Hz, 2H); **^{13}C NMR** (125 MHz; $CDCl_3$): δ = 31.2, 34.5, 61.4, 126.0, 126.9, 127.8, 128.6, 128.7, 129.0, 132.1, 133.8, 133.9, 140.6, 151.5, 194.3. IR (in KBr): 3321, 3067, 2958, 2867, 1681, 1400, 1163, 803, 756, 740 cm^{-1}. Mass Spectrometry: HRMS (ESI-TOF) (m/z): Calcd for $C_{24}H_{26}NO_3S$, ($[M + H]^+$), 408.1628, found 408.1637.

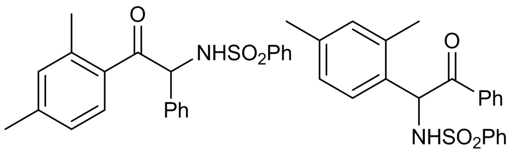

2n **3n**

***N*-(2-(2,4-dimethylphenyl)-2-oxo-1-phenylethyl)benzenesulfonamide 2n**

***N*-(1-(2,4-dimethylphenyl)-2-oxo-2-phenylethyl)benzenesulfonamide 3n**

The title compounds were prepared according to the synthesis of **2a** to afford **2n** and **3n** (48%). The products could not readily be separated by silica gel chromatography, so they were characterized as a mixture. The ratio of the isomor was determined by ^{1}H NMR Spectroscopy. The ^{1}H NMR spectrum of the product showed a 5:1 mixture of **3n** and a compound tentatively assigned as **2n** based on the methine peak at δ 5.89 for **2n** and at δ 6.07 for **3n**.

For **2n** and **3n**, NMR Spectroscopy: **^{1}H NMR** (500 MHz; $CDCl_3$): δ = 2.18 (s, 0.6H), 2.19 (s, 3H), 2.27 (s, 0.6H),2.44 (s, 3H), 5.89 (d, J = 7.0 Hz, 0.2H), 6.04 (d, J = 8.0 Hz, 1H), 6.08 (d, J = 7.5 Hz, 1H), 6.32 (d, J = 7.0 Hz, 0.2H), 6.76–6.80 (m, 2H), 6.90 (s, 1H), 6.92 (s, 0.2H), 6.95–6.97 (m, 0.3H), 7.06–7.07 (m, 0.6H), 7.11–7.12 (m, 0.7H), 7.27–7.34 (m, 4H), 7.39–7.43 (m, 1.5H), 7.47 (t, J = 7.5 Hz, 1H), 7.66(t, J = 7.0 Hz, 4H); **^{13}C NMR** (125 MHz; $CDCl_3$): δ = 19.2, 20.8, 59.2, 126.8, 127.2, 127.9, 128.3, 128.5, 128.6, 130.4, 132.1, 132.2, 133.6, 134.1, 136.2, 138.6, 140.0, 195.2. Mass Spectrometry: HRMS (ESI-TOF) (m/z): Calcd for $C_{22}H_{21}NNaO_3S$, ($[M + Na]^+$), 402.1134, found 402.1142.

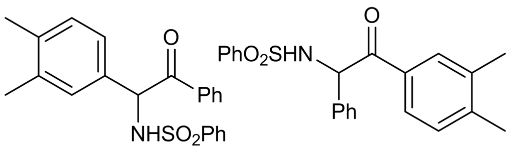

3o **2o**

***N*-(1-(3,4-dimethylphenyl)-2-oxo-2-phenylethyl)benzenesulfonamide 3o**

N-(2-(3,4-dimethylphenyl)-2-oxo-1-phenylethyl)benzenesulfonamide 2o

The title compounds were prepared according to the synthesis of **2a** to afford **3o** and **2o** (56%). The ratio of the isomer was determined by ^{1}H NMR Spectroscopy. The ^{1}H NMR spectrum of the product showed a 20:1 mixture of **3o** and a compound tentatively assigned as **2o** based on the methyl peak at δ 2.22 for **2o** and at δ 2.11 for **3o**.

For **3o**, white solid, mp. 171–174 °C. NMR Spectroscopy: **^{1}H NMR** (500 MHz; $CDCl_3$): δ = 2.06 (s, 3H), 2.11 (s, 3H), 5.95 (d, J = 7.5 Hz ,1H), 6.19 (d, J = 7.0 Hz, 1H), 6.85 (s, 1H), 6.92 (s, 2H), 7.24–7.26 (m, 2H), 7.37 (q, J = 8.0 Hz, 3H), 7.49 (t, J = 7.5 Hz, 1H), 7.61 (d, J = 7.5 Hz, 2H) , 7.81 (d, J = 7.5 Hz, 2H); **^{13}C NMR** (125 MHz; $CDCl_3$): δ =19.3, 19.5, 61.5, 125.6, 126.8, 128.4, 128.6, 128.9, 129.0, 130.1, 132.0, 132.5, 133.7, 133.8, 137.1, 137.3, 140.4, 194.3. IR (in KBr): 3261, 3069, 2966, 2926, 1686, 1385, 1167, 882, 814, 753, 741 cm^{-1}. Mass Spectrometry: HRMS (ESI-TOF) (m/z): Calcd for $C_{22}H_{21}NNaO_3S$, ([M + Na]$^+$), 402.1134, found 402.1137.

Aminative multifunctionalization of terminal alkynes with NFSI derivatives. (take 4b as an example).

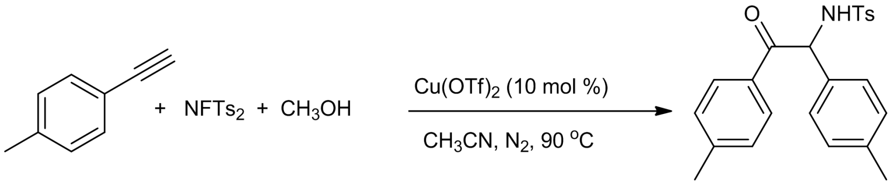

1j **4b**

To a solution of the $NFTs_2$ (0.3 mmol, 102.9 mg) in CH_3CN (2.0 mL) was added the CH_3OH (0.6 mmol, 24 μL), TFA (0.2 mmol, 15 μL), p-methyl phenylethyne (**1j**, 0.2 mmol, 22 μL) and $Cu(OTf)_2$ (0.02 mmol, 7.2 mg) in screw-cap test tube under N_2 atmosphere. The test tube was then sealed off with a screw-cap and the reaction was stirred at 90 °C for the 8.0 h. After the reaction finished, the reaction mixture was cooled to room temperature and quenched by water. The mixture was extracted with CH_2Cl_2 (3 × 5.0 mL), the combined organic phases were dried over anhydrous Na_2SO_4 and the solvent was evaporated under vacuum. The residue was purified by column chromatography (petroleum ether/ethyl acetate 10:1 (v/v)) to give the corresponding product **4b** (56.6 mg, 72%).

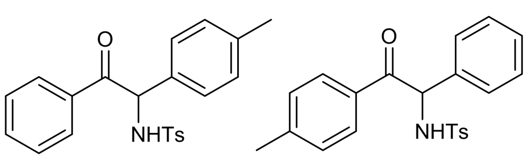

4a **5a**

4-methyl-N-(2-oxo-2-phenyl-1-(p-tolyl)ethyl)benzenesulfonamide 4a

4-methyl-N-(2-oxo-1-phenyl-2-(p-tolyl)ethyl)benzenesulfonamide 5a

The title compounds were prepared according to the synthesis of **4b** to afford **4a** and **5a** (84%). The ratio of the isomer was determined by ^{1}H NMR Spectroscopy. The ^{1}H NMR spectrum of the product showed a 8:1 mixture of **4a** and a compound tentatively assigned as **5a** based on the methyl peak at δ 2.22 for **4a** and at δ 2.33 for **5a**. **4a** was further purified by silica gel chromatography (petroleum ether/ diethyl ether 10:1 (v/v)).

For **4a**, white solid, mp. 160–162 °C. NMR Spectroscopy: **^{1}H NMR** (500 MHz; $CDCl_3$): δ = 2.22 (s, 3H), 2.30 (s, 3H), 5.96 (d, J = 8.0 Hz, 1H), 6.24 (d, J = 7.5 Hz, 1H), 6.96 (d, J = 8.0 Hz, 2H), 7.05 (d, J = 6.5 Hz, 4H), 7.35 (t, J = 7.5 Hz, 2H), 7.47–7.53 (m, 3H), 7.52 (d, J = 8.0 Hz, 2H), 7.79 (d, J = 8.0 Hz, 2H); **^{13}C NMR** (125 MHz; $CDCl_3$): δ = 21.0, 21.4, 61.4, 126.9, 127.9, 128.6, 128.9, 129.2, 129.7, 132.5, 133.7, 133.8, 137.3, 138.4, 143.0, 194.5. IR (in KBr): 3273, 3064, 2923, 1686, 1376, 1161, 813, 754 cm^{-1}. Mass Spectrometry: HRMS (ESI-TOF) (m/z): Calcd for $C_{22}H_{22}NO_3S$, ([M + H]$^+$), 380.1315, found 380.1318.

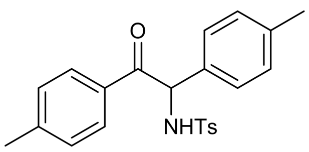

4b

4-methyl-*N*-(2-oxo-1,2-di-*p*-tolylethyl)benzenesulfonamide 4b

For **4b,** White solid, mp. 160–162 °C. NMR Spectroscopy: **^{1}H NMR** (500 MHz; $CDCl_3$): δ = 2.22 (s, 3H), 2.31 (s, 3H), 2.33 (s, 3H), 5.92 (d, J = 7.0 Hz, 1H), 6.20 (d, J = 7.5 Hz, 1H), 6.96 (d, J = 8.0 Hz, 2H), 7.04–7.08 (m, 4H), 7.15 (d, J = 8.0 Hz, 2H), 7.51 (d, J = 8.5 Hz, 2H), 7.70 (d, J = 8.0 Hz, 2H); **^{13}C NMR** (125 MHz; $CDCl_3$): δ = 21.0, 21.4, 21.7, 61.3, 126.9, 127.9, 129.0, 129.2, 129.3, 129.6, 131.2, 132.9, 137.4, 138.3, 142.9, 145.0, 194.0. IR (in KBr): 3278, 3061, 1687, 1591, 1506, 1364, 1161, 823 cm^{-1}. Mass Spectrometry: HRMS (ESI-TOF) (m/z): Calcd for $C_{23}H_{24}NO_3S$, ([M + H]$^+$), 394.1471, found 394.1480.

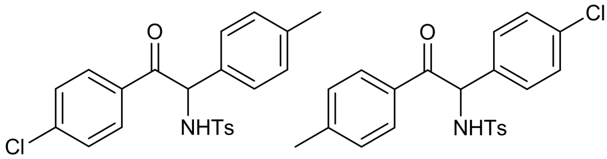

4c **5c**

***N*-(2-(4-chlorophenyl)-2-oxo-1-(*p*-tolyl)ethyl)-4-methylbenzenesulfonamide 4c**

***N*-(1-(4-chlorophenyl)-2-oxo-2-(*p*-tolyl)ethyl)-4-methylbenzenesulfonamide 5c**

The title compounds were prepared according to the synthesis of **4b** to afford **4c** and **5c** (67%). The ratio of the isomer was determined by ^{1}H NMR Spectroscopy. The ^{1}H NMR spectrum of the product showed a > 20:1 mixture of **4c** and a compound tentatively assigned as **5c** based on the methyl peak at δ 2.24 for **4c** and at δ 2.35 for **5c**. **4c** was futher purified by silica gel chromatography (petroleum ether/ diethyl ether 10:1 (v/v)).

For **4c**, white solid, mp. 164–167 °C. NMR Spectroscopy: **^{1}H NMR** (500 MHz; $CDCl_3$): δ = 2.24 (s, 3H), 2.32 (s, 3H), 5.89 (d, J = 7.5 Hz, 1H), 6.14 (d, J = 7.5 Hz, 1H), 6.98 (d, J = 8.0 Hz, 2H), 7.02 (d, J = 8.5 Hz, 2H), 7.07 (d, J = 8.0 Hz, 2H), 7.33 (d, J = 8.5 Hz, 2H), 7.51 (d, J = 8.0 Hz, 2H), 7.73 (d, J = 8.5 Hz, 2H); **^{13}C NMR** (125 MHz; $CDCl_3$): δ = 21.1, 21.4, 61.5, 126.9, 127.9, 129.0, 129.3, 129.8, 130.3, 132.1, 132.3, 137.4, 138.7, 140.4, 143.1, 193.4. IR (in KBr): 3283, 3092, 3048, 2956, 2921, 1687, 1591, 1510, 1449, 1337, 1161, 1090, 828, 811 cm^{-1}. Mass Spectrometry: HRMS (ESI-TOF) (m/z): Calcd for $C_{22}H_{21}ClNO_3S$, ([M + H]$^+$), 414.0925, found 414.0918.

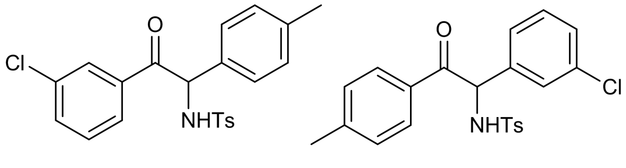

4d **5d**

***N*-(2-(3-chlorophenyl)-2-oxo-1-(*p*-tolyl)ethyl)-4-methylbenzenesulfonamide 4d**
***N*-(1-(3-chlorophenyl)-2-oxo-2-(*p*-tolyl)ethyl)-4-methylbenzenesulfonamide 5d**

The title compounds were prepared according to the synthesis of **4b** to afford **4d** and **5d** (81%). The ratio of the isomer was determined by ^{1}H NMR Spectroscopy. The ^{1}H NMR spectrum of the product showed a 12:1 mixture of **4d** and a compound tentatively assigned as **4d** based on the methyl peak at δ 2.23 for **3d** and at δ 2.34 for **5d**. **4d** was further purified by silica gel chromatography (petroleum ether/ diethyl ether 10:1 (v/v)).

For **4d**, white solid, mp. 178–181 °C. NMR Spectroscopy: **^{1}H NMR** (500 MHz; $CDCl_3$): δ = 2.23 (s, 3H), 2.32 (s, 3H), 5.91 (d, J = 7.5 Hz, 1H), 6.20 (d, J = 7.5 Hz, 1H), 6.98 (d, J = 8.0 Hz, 2H), 7.03–7.09 (m, 4H), 7.30 (d, J = 7.5 Hz, 1H), 7.44–7.46 (m, 1H), 7.52 (d, J = 8.5 Hz, 2H), 7.65 (d, J = 8.0 Hz, 1H), 7.74 (s, 1H); **^{13}C NMR** (125 MHz; $CDCl_3$): δ = 21.0, 21.4, 61.6, 126.9, 127.0, 127.9, 128.8, 129.3, 129.8, 129.9, 131.9, 133.7, 135.0, 135.3, 137.2, 138.7, 143.1, 193.5. IR (in KBr): 3269, 3062, 2924, 1694, 1600, 1510, 1446, 1387, 1164, 1093, 876, 808, 674 cm^{-1}. Mass Spectrometry: HRMS (ESI-TOF) (m/z): Calcd for $C_{22}H_{21}ClNO_3S$, ([M + H]$^+$), 414.0925, found 414.0933.

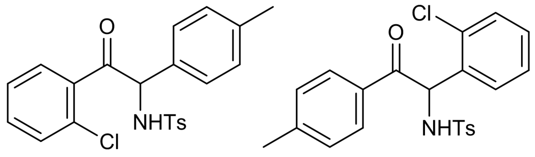

4e **5e**

***N*-(2-(2-chlorophenyl)-2-oxo-1-(*p*-tolyl)ethyl)-4-methylbenzenesulfonamide 4e**
***N*-(1-(2-chlorophenyl)-2-oxo-2-(*p*-tolyl)ethyl)-4-methylbenzenesulfonamide 5e**

The title compounds were prepared according to the synthesis of **4b** to afford **4e** and **5e** (78%). The ratio of the isomer was determined by ^{1}H NMR Spectroscopy. The ^{1}H NMR spectrum of the product showed a > 20:1 mixture of **4e** and a compound tentatively assigned as **5e** based on the methyl peak at δ 2.22 for **4e** and at δ 2.29 for **5e**. **4e** was futher purified by silica gel chromatography (petroleum ether/ diethyl ether 10:1 (v/v)).

For **4e**, white solid, mp. 152–154 °C. NMR Spectroscopy: **^{1}H NMR** (500 MHz; $CDCl_3$): δ = 2.22 (s, 3H), 2.35 (s, 3H), 5.87 (d, J = 6.5 Hz, 1H), 6.24 (d, J = 8.5 Hz, 1H), 6.92–6.93 (m, 4H), 7.09 (d, J = 7.5 Hz, 1H), 7.15 (d, J = 8.0 Hz, 3H), 7.26–7.30 (m, 2H), 7.60 (d, J = 8.0 Hz, 2H); **^{13}C NMR** (125 MHz; $CDCl_3$): δ = 21.1, 21.4, 64.5, 126.6, 127.1, 127.9, 129.3, 129.4, 129.5, 130.5, 131.0, 131.1, 132.3, 135.6, 137.2, 138.5, 143.2, 196.6. IR (in KBr): 3282, 3091, 3025, 2948, 2917, 1710, 1592, 1510, 1383, 1161, 1091, 838, 812, 733, 707 cm^{-1}. Mass Spectrometry: HRMS (ESI-TOF) (m/z): Calcd for $C_{22}H_{21}ClNO_3S$, ([M + H]$^+$), 414.0925, found 414.0927.

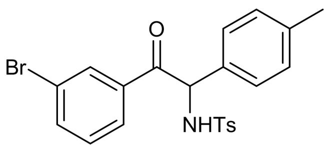

4f

***N*-(2-(3-bromophenyl)-2-oxo-1-(*p*-tolyl)ethyl)-4-methylbenzenesulfonamide 4f**

The title compound was prepared according to the synthesis of **4b** to afford **4f** (73%).

For **4f**, white solid, mp. 188–191 °C. NMR Spectroscopy: **^{1}H NMR** (500 MHz; $CDCl_3$): δ = 2.25 (s, 3H), 2.32 (s, 3H), 5.88 (d, J = 7.5 Hz, 1H), 6.09 (d, J = 7.5 Hz, 1H), 6.99 (d, J = 7.5 Hz, 2H), 7.03 (d, J = 7.5 Hz, 2H), 7.08 (d, J = 8.0 Hz, 2H), 7.23 (t, J = 8.0 Hz, 1H), 7.51 (d, J = 8.5 Hz, 2H), 7.61 (d, J = 7.5 Hz, 1H), 7.69 (d, J = 7.5 Hz, 1H), 7.90 (s, 1H); **^{13}C NMR** (125 MHz; $CDCl_3$): δ = 21.1, 21.4, 61.6, 123.0, 127.0, 127.3, 127.9, 129.3, 129.9, 130.2, 131.7, 131.9, 135.5, 136.6, 137.2, 138.7, 143.2, 193.5. IR (in KBr): 3267, 3058, 2923, 1691, 1596, 1510, 1449, 1413, 1336, 1166, 1095, 881, 855, 813, 773, 709 cm^{-1}. Mass Spectrometry: HRMS (ESI-TOF) (m/z): Calcd for $C_{22}H_{21}BrNO_3S$, ([M + H]$^+$), 458.0420, found 458.0424.

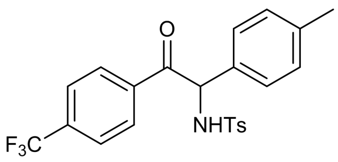

4g

4-methyl-*N*-(2-oxo-1-(*p*-tolyl)-2-(4-(trifluoromethyl)phenyl)ethyl)benzenesulfonamide 4g

The title compound was prepared according to the synthesis of **4b** to afford **4g** (81%).

For **4g**, white solid, mp. 185–187 °C. NMR Spectroscopy: **^{1}H NMR** (500 MHz; $CDCl_3$): δ = 2.24 (s, 3H), 2.32 (s, 3H), 5.95 (d, J = 7.0 Hz, 1H), 6.12 (d, J = 7.5 Hz, 1H), 6.99 (d, J = 7.5 Hz, 2H), 7.03 (d, J = 7.5 Hz, 2H), 7.08 (d, J = 8.0 Hz, 2H), 7.52 (d, J = 8.5 Hz, 2H), 7.62 (d, J = 8.5 Hz, 2H), 7.90 (d, J = 8.5 Hz, 2H); **^{13}C NMR** (125 MHz; $CDCl_3$): δ = 21.1, 21.4, 61.9, 123.2 (q, J = 271 Hz), 125.7 (q, J = 4 Hz), 126.9, 128.0, 129.2, 129.3, 129.9, 131.8, 134.9 (q, J = 33 Hz), 136.5, 137.3, 138.9, 143.2, 193.7. IR (in KBr): 3286, 3052, 2954, 1693, 1597, 1582, 1512, 1492, 1383, 1253, 1161, 835, 810 cm^{-1}. Mass Spectrometry: HRMS (ESI-TOF) (m/z): Calcd for $C_{23}H_{21}F_3NO_3S$, ([M + H]$^+$), 448.1189, found 448.1196.

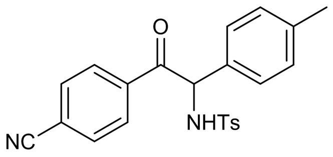

4h

***N*-(2-(4-cyanophenyl)-2-oxo-1-(*p*-tolyl)ethyl)-4-methylbenzenesulfonamide 4h**

The title compound was prepared according to the synthesis of **4b** to afford **4h** (48%).

For **4h**, white solid, mp. 184–186 °C. NMR Spectroscopy: **^{1}H NMR** (500 MHz; $CDCl_3$): δ = 2.24 (s, 3H), 2.33 (s, 3H), 5.93 (d, J = 7.5 Hz, 1H), 6.10 (d, J = 7.5 Hz, 1H), 6.98–7.02 (m, 4H), 7.08 (d,

$J = 8.0$ Hz, 2H), 7.51 (d, $J = 8.5$ Hz, 2H), 7.66 (d, $J = 8.5$ Hz, 2H), 7.88 (d, $J = 8.5$ Hz, 2H); ^{13}C NMR (125 MHz; $CDCl_3$): δ = 21.1, 21.4, 62.0, 116.9, 117.5, 126.9, 128.0, 129.2, 129.3, 130.0, 131.4, 132.4, 136.9, 137.3, 139.0, 143.2, 193.4. IR (in KBr): 3263, 2922, 2853, 2330, 1695, 1596, 1508, 1450, 1331, 1161, 809 cm^{-1}. Mass Spectrometry: HRMS (ESI-TOF) (m/z): Calcd for $C_{23}H_{21}N_2O_3S$, ([M + H]$^+$), 405.1267, found 405.1275.

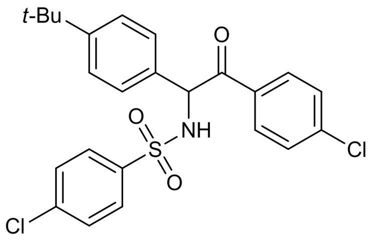

5i

***N*-(1-(4-(*tert*-butyl)phenyl)-2-(4-chlorophenyl)-2-oxoethyl)-4-chlorobenzenesulfonamide 5i**

The title compound was prepared according to the synthesis of **4b** to afford **5i** (46%).

For **5i**, white solid, mp. 182–184 °C. NMR Spectroscopy: **^{1}H NMR** (500 MHz; $CDCl_3$): δ = 1.21 (s, 9H), 6.01 (d, $J = 7.0$ Hz, 1H), 6.37 (d, $J = 7.0$ Hz, 1H), 7.01 (d, $J = 8.0$ Hz, 2H), 7.08–7.18 (m, 4H), 7.35 (d, $J = 7.0$ Hz, 2H), 7.46 (d, $J = 7.0$ Hz, 2H), 7.81 (d, $J = 7.0$ Hz, 2H); ^{13}C NMR (125 MHz; $CDCl_3$): δ = 31.1, 34.5, 61.6, 126.1, 127.8, 128.3, 128.7, 129.1, 130.4, 131.3, 131.8, 138.4, 139.2, 140.6, 152.0, 192.7. IR (in KBr): 3259, 3096, 2962, 1688, 1590, 1323, 1155, 1091, 836, 817 cm^{-1}. Mass Spectrometry: HRMS (ESI-TOF) (m/z): Calcd for $C_{24}H_{23}Cl_2NNaO_3S$, ([M + Na]$^+$), 498.0668, found 498.0663.

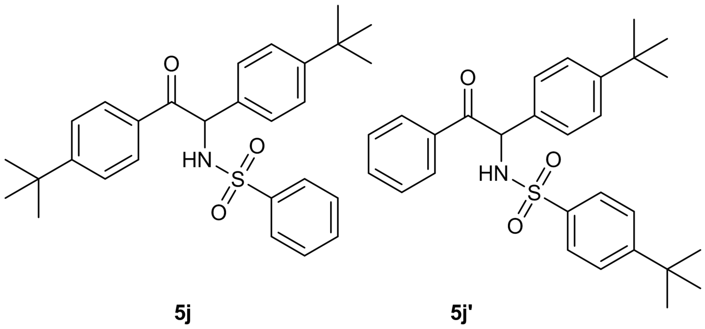

5j **5j'**

***N*-(1,2-bis(4-(*tert*-butyl)phenyl)-2-oxoethyl)benzenesulfonamide 5j**

4-(*tert*-butyl)-*N*-(1-(4-(*tert*-butyl)phenyl)-2-oxo-2-phenylethyl)benzenesulfonamide 5j'

The title compounds were prepared according to the synthesis of **4b** to afford **5j** and **5j'** (72%).

The ratio of the isomer was determined by ^{1}H NMR Spectroscopy. The ^{1}H NMR spectrum of the product showed a 10:1 mixture of **5j** and a compound tentatively assigned as **5j'** based on the imine peak at δ 6.29 for **5j** and at δ 6.22 for **5j'**. **5j** was futher purified by silica gel chromatography (petroleum ether / diethyl ether 10:1 (v/v)).

For **5j**, white solid, mp. 98–100 °C. NMR Spectroscopy: **^{1}H NMR** (500 MHz; $CDCl_3$): δ = 1.21 (s, 9H), 1.28 (s, 9H), 6.01 (d, $J = 7.5$ Hz, 1H), 6.26 (d, $J = 7.5$ Hz, 1H), 7.08 (d, $J = 8.5$ Hz, 2H), 7.14 (d, $J = 8.5$ Hz, 2H), 7.22 (d, $J = 7.5$ Hz, 2H), 7.34 (t, $J = 7.5$ Hz, 1H), 7.38 (d, $J = 8.5$ Hz, 2H), 7.59 (d, $J = 7.5$ Hz, 2H), 7.79 (d, $J = 8.5$ Hz, 2H); ^{13}C NMR (125 MHz; $CDCl_3$): δ = 30.9, 31.2, 34.5, 35.2, 61.3, 125.7, 125.9, 126.8, 127.8, 128.5, 129.0, 131.1, 132.1, 140.6, 151.4, 157.9, 193.4. IR (in KBr): 3296, 3064, 2964, 2869, 1675, 1683, 1602, 1509, 1340, 1164, 1089, 824 cm^{-1}. Mass

Spectrometry: HRMS (ESI-TOF) (m/z): Calcd for $C_{28}H_{34}NO_3S$, ([M + H]$^+$), 464.2254, found 464.2250.

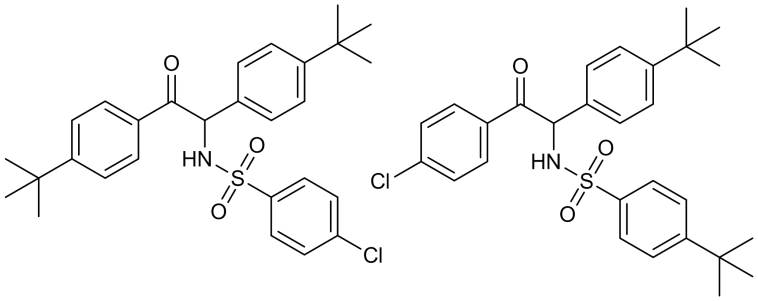

5k **5k'**

***N*-(1,2-bis(4-(*tert*-butyl)phenyl)-2-oxoethyl)-4-chlorobenzenesulfonamide 5k**
4-(*tert*-butyl)-*N*-(1-(4-(*tert*-butyl)phenyl)-2-(4-chlorophenyl)-2-oxoethyl)benzenesulfonamide 5k'

The title compounds were prepared according to the synthesis of **4b** to afford **5k** and **5k'** (67%). The ratio of the isomer was determined by ^{1}H NMR Spectroscopy. The ^{1}H NMR spectrum of the product showed a >20:1 mixture of **5k** and a compound tentatively assigned as **5k'** based on the imine peak at δ 6.32 for **5k** and at δ 6.15 for **5k'**.

For **5k**, white solid, mp. 164–166 °C. NMR Spectroscopy: **^{1}H NMR** (500 MHz; $CDCl_3$): δ = 1.22 (s, 9H), 1.25 (s, 9H), 6.01 (d, J = 7.0 Hz, 1H), 6.32 (d, J = 7.0 Hz, 1H), 7.06 (d, J = 8.0 Hz, 2H), 7.11–7.18 (m, 4H), 7.40 (d, J = 8.5 Hz, 2H), 7.46 (d, J = 8.5 Hz, 2H), 7.81 (d, J = 8.5 Hz, 2H); **^{13}C NMR** (125 MHz; $CDCl_3$): δ = 30.9, 31.2, 34.5, 35.2, 61.3, 125.7, 125.9, 127.8, 128.3, 128.6, 129.1, 130.9, 131.9, 138.3, 139.3, 151.7, 158.1, 193.3. IR (in KBr): 3296, 3065, 2966, 2868, 1675, 1606, 1566, 1510, 1338, 1165, 1089, 839, 822 cm^{-1}. Mass Spectrometry: HRMS (ESI-TOF) (m/z): Calcd for $C_{28}H_{33}ClNO_3S$, ([M + H]$^+$), 498.1864, found 498.1860.

General procedure for aminative multifunctionalization of internal alkynes with NFSI (take 8a as an example).

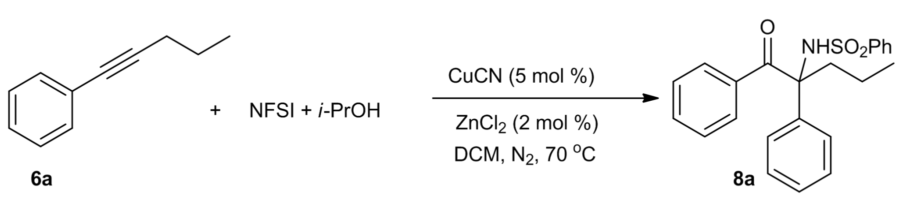

To a solution of the NFSI (0.75 mmol, 236.5 mg) in CH_2Cl_2 (2.0 mL) was added the isopropanol (0.75 mmol, 57 μL), but-1-yn-1-ylbenzene (**6a**, 0.5 mmol, 80 μL), $ZnCl_2$ (0.01 mmol, 1.4 mg), and CuCN (0.025 mmol, 2.2 mg) in screw-cap test tube under N_2 atmosphere. The test tube was then sealed off with a screw-cap and the reaction was stirred for the 12.0 h at 70 ℃. After the reaction finished, the reaction mixture was cooled to room temperature and quenched by water. The mixture was extracted with CH_2Cl_2 (3 × 5.0 mL), the combined organic phases were dried over anhydrous Na_2SO_4 and the solvent was evaporated under vacuum. The residue was purified by column chromatography (petroleum ether/ethyl acetate 10:1 (v/v)) to give the corresponding product **8a** (133.7 mg, 68%).

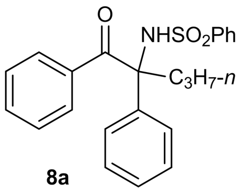

8a

N-(1-oxo-1,2-diphenylpentan-2-yl)benzenesulfonamide 8a

The title compound was prepared according to the synthesis of **8a** to afford **8a** (68%).

For **8a**, white solid, mp. 211–213 °C. NMR Spectroscopy: **^{1}H NMR** (500 MHz; $CDCl_3$): δ = 0.83–0.90 (m, 4H), 1.69–1.76 (m, 1H), 2.23–2.32 (m, 1H), 3.11–3.17 (m, 1H), 6.84 (s, 1H), 7.02 (s, 2H), 7.09–7.14 (m, 4H), 7.18–7.24 (m, 4H), 7.26–7.29 (m, 1H), 7.36–7.41 (m, 3H); ^{13}C NMR (125 MHz; $CDCl_3$): δ = 14.1, 16.6, 37.7, 70.7, 126.1, 127.7, 128.1, 128.2, 128.4, 128.6, 129.8, 131.1, 132.9, 133.5, 136.3, 142.3, 197.1. IR (in KBr): 3248, 3064, 1679, 1594, 1496, 1448, 1359, 1164, 766, 747, 690 cm^{-1}. Mass Spectrometry: HRMS (ESI-TOF) (m/z): Calcd for $C_{23}H_{23}NNaO_3S$, ([M + Na]$^+$), 416.1291, found 416.1284.

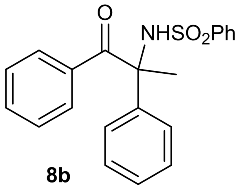

8b

N-(1-oxo-1,2-diphenylpropan-2-yl)benzenesulfonamide 8b

The title compound was prepared according to the synthesis of **8a** to afford **8b** (72%).

For **8b,** white solid, mp. 217–220 °C. NMR Spectroscopy: **^{1}H NMR** (500 MHz; $CDCl_3$): δ = 2.18 (s, 3H), 6.91 (s, 1H), 7.05 (t, J = 7.5 Hz, 2H), 7.12–7.16 (m, 5H), 7.19–7.22 (m, 2H), 7.26–7.31 (m, 3H), 7.38–7.41 (m, 3H); **^{13}C NMR** (125 MHz; $CDCl_3$): δ = 25.0, 67.3, 126.2, 127.5, 128.2, 128.4, 128.6, 130.1, 131.2, 132.9, 133.1, 136.3, 142.2, 197.4. IR (in KBr): 3298, 3058, 1684, 1595, 1495, 1447, 1367, 1169, 759, 703 cm^{-1}. Mass Spectrometry: HRMS (ESI-TOF) (m/z): Calcd for $C_{21}H_{19}NNaO_3S$, ([M + Na]$^+$), 388.0978, found 388.0980.

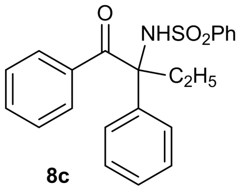

8c

N-(1-oxo-1,2-diphenylbutan-2-yl)benzenesulfonamide 8c

The title compound was prepared according to the synthesis of **8a** to afford **8c** (73%).

For **8c,** white solid, mp. 221–224 °C. NMR Spectroscopy: **^{1}H NMR** (500 MHz; $CDCl_3$): δ = 0.89 (t, J = 7.5 Hz, 3H), 2.32–2.39 (m, 1H), 3.20–3.27 (m, 1H), 6.83 (s, 1H), 7.02 (s, 2H), 7.09–7.14 (m, 4H), 7.20–7.22 (m, 4H), 7.27 (t, J = 7.5 Hz, 1H), 7.37 (d, J = 7.5 Hz, 2H), 7.40 (t, J = 7.0 Hz, 1H); **^{13}C NMR** (125 MHz; $CDCl_3$): δ = 7.7, 28.6, 71.1, 126.1, 127.8, 128.1, 128.2, 128.4, 128.6, 129.9, 131.1, 133.0, 133.5, 136.1, 142.3, 197.2. IR (in KBr): 3247, 3062, 1677, 1591, 1448, 1359, 1163, 757, 709 cm^{-1}. Mass Spectrometry: HRMS (ESI-TOF) (m/z): Calcd for $C_{22}H_{21}NNaO_3S$, ([M + Na]$^+$), 402.1134, found 402.1138.

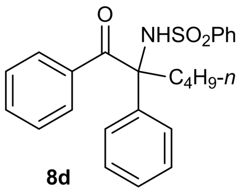

8d

***N*-(1-oxo-1,2-diphenylhexan-2-yl)benzenesulfonamide 8d**

The title compound was prepared according to the synthesis of **8a** to afford **8d** (64%).

For **8d**, white solid, mp. 194–196 °C. NMR Spectroscopy: **^{1}H NMR** (500 MHz; $CDCl_3$): δ = 0.74–0.83 (m, 4H), 1.13–1.20 (m, 1H), 1.27–1.35 (m, 1H), 1.65–1.72 (m, 1H), 2.25–2.31 (m, 1H), 3.13–3.19 (m, 1H), 6.83 (s, 1H), 7.02 (s, 2H), 7.11–7.13 (m, 4H), 7.18–7.24 (m, 4H), 7.26–7.29 (m, 1H), 7.36–7.41 (m, 3H); **^{13}C NMR** (125 MHz; $CDCl_3$): δ = 13.7, 22.6, 25.3, 35.2, 70.7, 126.2, 127.7, 128.1, 128.2, 128.3, 128.6, 129.8, 131.1, 132.9, 133.6, 136.4, 142.4, 197.2. IR (in KBr): 3245, 3065, 2959, 2929, 2871, 1696, 1610, 1493, 1447, 1379, 1171, 752, 700 cm^{-1}. Mass Spectrometry: HRMS (ESI-TOF) (m/z): Calcd for $C_{24}H_{25}NNaO_3S$, ([M + Na]$^+$), 430.1447, found 430.1440.

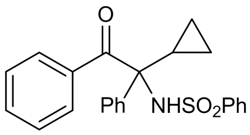

8e

***N*-(1-cyclopropyl-2-oxo-1,2-diphenylethyl)benzenesulfonamide 8e**

The title compound was prepared according to the synthesis of **8a** to afford **8e** (50%).

For **8e,** white solid, mp. 178–180 °C. NMR Spectroscopy: **^{1}H NMR** (500 MHz; $CDCl_3$): δ = 0.25–0.28 (m, 2H), 0.91–0.97 (m, 1H), 1.50–1.55 (m, 1H), 1.59–1.64 (m, 1H), 6.64(s, 1H), 6.99 (s, 2H), 7.12–7.21 (m, 8H), 7.32 (t, *J* = 7.0 Hz, 1H), 7.39 (t, *J* = 8.0 Hz, 1H), 7.43 (d, *J*=8.0 Hz, 2H); **^{13}C NMR** (125 MHz; $CDCl_3$): ^{13}C NMR (125 MHz; $CDCl_3$): δ = -0.4, 4.9, 18.2, 70.4, 126.2, 128.0, 128.1, 128.3, 128.4, 129.5, 130.6, 131.3, 133.0, 133.4, 135.2, 142.5, 195.9. IR (in KBr): 3286, 3063, 2965, 2926, 2855, 1673, 1596, 1491, 1447, 1377, 1167, 752, 704 cm^{-1}. Mass Spectrometry: HRMS (ESI-TOF) (m/z): Calcd for $C_{23}H_{21}NNaO_3S$, ([M + Na]$^+$), 414.1134, found 414.1130.

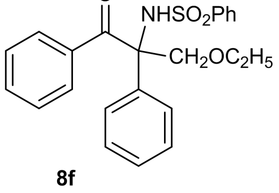

8f

***N*-(3-ethoxy-1-oxo-1,2-diphenylpropan-2-yl)benzenesulfonamide 8f**

The title compound was prepared according to the synthesis of **8a** to afford **8f** (70%).

For **8f,** white solid, mp. 215–218 °C. NMR Spectroscopy: **^{1}H NMR** (500 MHz; $CDCl_3$): δ = 0.95 (t, *J* = 7.0 Hz, 3H), 3.17–3.20 (m, 1H), 3.40–3.44 (m, 1H), 4.29 (d, *J* = 9.0 Hz, 1H), 4.75 (d, *J* = 8.5 Hz, 1H), 6.80 (s, 1H), 7.02 (d, *J* = 7.5 Hz, 2H), 7.08 (t, *J* = 7.5 Hz, 2H), 7.14–7.20 (m, 5H),

7.26–7.28 (m, 2H), 7.31 (t, J = 7.5 Hz, 3H), 7.38 (t, J = 7.5 Hz, 1H); **^{13}C NMR** (125 MHz; $CDCl_3$): δ = 14.6, 67.1, 70.9, 72.4, 126.3, 127.8, 128.0, 128.2, 128.7, 129.3, 131.3, 132.3, 134.0, 134.6, 142.4, 196.1. IR (in KBr): 3256, 3067, 1690, 1597, 1491, 1447, 1359, 1159, 1090, 770, 750, 702 cm^{-1}. Mass Spectrometry: HRMS (ESI-TOF) (m/z): Calcd for $C_{23}H_{23}NNaO_4S$, ([M + Na]$^+$), 432.1240, found 432.1244.

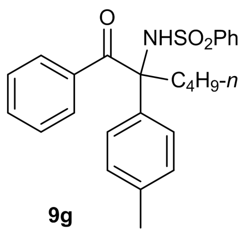

9g

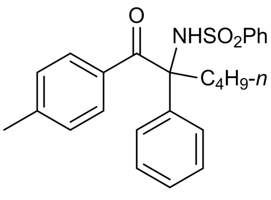

8g

***N*-(1-oxo-1-phenyl-2-(*p*-tolyl)hexan-2-yl)benzenesulfonamide 9g**

***N*-(1-oxo-2-phenyl-1-(*p*-tolyl)hexan-2-yl)benzenesulfonamide 8g**

The title compounds were prepared according to the synthesis of **8a** to afford **9g** and **8g** (51%).

The ratio of the isomer was determined by ^{1}H NMR Spectroscopy, the ^{1}H NMR spectrum of the product showed a 11:1 mixture of **9g** and a compound tentatively assigned as **8g** based on the imine peak at δ 6.85 for **9g** and at δ 6.88 for **8g**.

For **9g**, white solid, mp. 185–187 °C. NMR Spectroscopy: **^{1}H NMR** (500 MHz; $CDCl_3$): δ = 0.75–0.82 (m, 4H), 1.12–1.19 (m, 1H), 1.27–1.34 (m, 1H), 1.65–1.71 (m, 1H), 2.24 (s, 3H), 2.24–2.30 (m, 1H), 3.10–3.16 (m, 1H), 6.78 (s, 2H), 6.85 (s, 1H), 6.98–6.70 (m, 2H), 7.10 (t, J = 7.5 Hz, 2H), 7.19–7.30 (m, 5H), 7.38–7.41 (m, 3H); **^{13}C NMR** (125 MHz; $CDCl_3$): δ = 13.7, 21.0, 22.6, 25.3, 35.2, 70.4, 126.3, 127.6, 128.0, 128.2, 129.1, 129.9, 130.8, 132.8, 133.6, 133.7, 138.2, 142.4, 197.4. IR (in KBr): 3226, 2954, 2925, 1673, 1448, 1366, 1166, 750, 688 cm^{-1}. Mass Spectrometry: HRMS (ESI-TOF) (m/z): Calcd for $C_{25}H_{27}NNaO_3S$, ([M + Na]$^+$), 444.1604, found 444.1614.

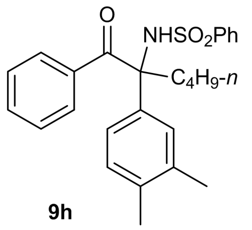

9h

***N*-(2-(3,4-dimethylphenyl)-1-oxo-1-phenylhexan-2-yl)benzenesulfonamide 9h**

The title compound was prepared according to the synthesis of **8a** to afford **9h** (54%).

For **9h**, white solid, mp. 173–176 °C. NMR Spectroscopy: **^{1}H NMR** (500 MHz; $CDCl_3$): δ = 0.74–0.80 (m, 4H), 1.12–1.19 (m, 1H), 1.25–1.35 (m, 1H), 1.67–1.74 (m, 1H), 1.83 (s, 3H), 2.11 (s, 3H), 2.21–2.27 (m, 1H), 3.10–3.16 (m, 1H), 6.55 (s, 1H), 6.85–6.90 (m, 2H), 7.08 (t, J = 7.5 Hz, 3H), 6.20–7.28 (m, 5H), 7.39–7.43 (m, 3H); **^{13}C NMR** (125 MHz; $CDCl_3$): δ = 13.7, 19.4, 19.5, 22.6, 25.3, 35.1, 70.3, 124.7, 126.3, 127.7, 128.2, 129.7, 129.9, 130.8, 132.8, 133.8, 136.6, 136.9, 142.2, 197.5. IR (in KBr): 3263, 3065, 2960, 2862, 1679, 1595, 1503, 1447, 1358, 1159, 863, 830, 740, 694 cm^{-1}. Mass Spectrometry: HRMS (ESI-TOF) (m/z): Calcd for $C_{26}H_{29}NNaO_3S$, ([M + Na]$^+$), 458.1760, found 458.1769.

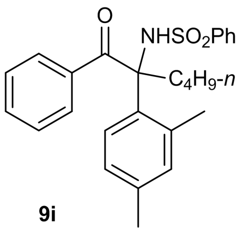

9i

***N*-(1-(2,4-dimethylphenyl)-1-oxo-2-phenylhexan-2-yl)benzenesulfonamide 9i**

The title compound was prepared according to the synthesis of **8a** to afford **9i** (55%).

For **9i,** white solid, mp. 168–171 °C. NMR Spectroscopy: **^{1}H NMR** (500 MHz; $CDCl_3$): δ = 0.73–0.82 (m, 4H), 1.08–1.17 (m, 1H), 1.26–1.31 (m, 1H), 1.68–1.74 (m, 1H), 2.17–2.23 (m, 4H), 2.42 (s, 3H), 3.02–3.08 (m, 1H), 6.48 (d, J = 8.0 Hz, 1H), 6.60 (d, J = 8.0 Hz, 1H), 6.84 (s, 1H), 7.01–7.04 (m, 3H), 7.12 (t, J = 8.0 Hz, 5H), 7.25 (d, J = 8.0 Hz, 2H), 7.28 (t, J = 7.5Hz, 1H); **^{13}C NMR** (125 MHz; $CDCl_3$): δ = 13.7, 21.2, 21.6. 22.7, 25.3, 35.0, 71.2, 125.5, 126.1, 127.3, 128.2, 128.5, 129.0, 130.6, 131.1, 133.2, 136.5, 140.3, 142.0, 142.3, 199.4. IR (in KBr): 2955, 2924, 2869, 1711, 1449, 1376, 1169, 855, 805, 754, 689 cm^{-1}. Mass Spectrometry: HRMS (ESI-TOF) (m/z): Calcd for $C_{26}H_{29}NNaO_3S$, ($[M + Na]^+$), 458.1760, found 458.1768.

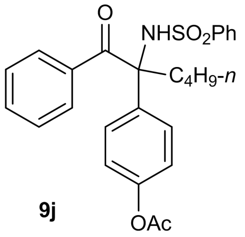

9j

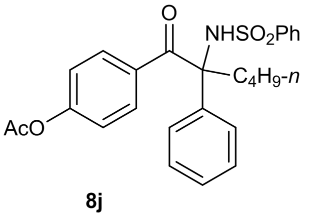

8j

4-(1-oxo-1-phenyl-2-(phenylsulfonamido)hexan-2-yl)phenyl acetate 9j

4-(2-phenyl-2-(phenylsulfonamido)hexanoyl)phenyl acetate 8j

The title compounds were prepared according to the synthesis of **8a** to afford **8j and 9j** (48%).

The products could not readily be separated by silica gel chromatography, so they were characterized as a mixture. The ratio of the isomer was determined by ^{1}H NMR Spectroscopy. The ^{1}H NMR spectrum of the product showed a 4:1 mixture of **9j** and a compound tentatively assigned as **8j** based on the methyl peak at δ 2.26 for **9j** and at δ 2.22 for **8j**.

For **9j** and **8j**, NMR Spectroscopy: **^{1}H NMR** (500 MHz; $CDCl_3$): δ = 0.72–0.78 (m, 4H), 1.11–1.21 (m, 1H), 1.25–1.32 (m, 1H), 1.66–1.72 (m, 1H), 2.22–2.31 (m, 4H), 3.08–3.18 (m, 1H), 6.76 (s, 1.2H), 6.81–6.82 (m, 1.1H), 6.91–6.99 (m, 1.4H), 7.06–7.10 (m, 2H), 7.14–7.18 (m, 2H), 7.19–7.15 (m, 3.6H), 7.29 (t, J = 7.5 Hz, 0.8H), 7.35–7.43 (m, 2.8H); **^{13}C NMR** (125 MHz; $CDCl_3$): δ = 13.6, 13.9, 21.0, 21.1, 22.5, 22.6, 25.3, 35.3, 35.6, 70.2, 70.6, 121.3, 121.6, 126.0, 126.1, 128.2, 128.3, 128.4, 128.6, 128.8, 129.8, 130.8, 131.1, 131.3, 131.6, 133.0, 133.3, 133.9, 136.3, 142.2, 142.3, 150.6, 154.0, 168.5, 168.6, 195.8, 196.9. Mass Spectrometry: HRMS (ESI-TOF) (m/z): Calcd for $C_{26}H_{27}NNaO_5S$, ($[M + Na]^+$), 488.1502, found 488.1508.

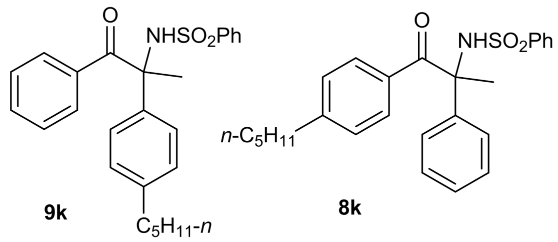

***N*-(1-oxo-2-(4-pentylphenyl)-1-phenylpropan-2-yl)benzenesulfonamide 9k**

***N*-(1-oxo-1-(4-pentylphenyl)-2-phenylpropan-2-yl)benzenesulfonamide 8k**

The title compounds were prepared according to the synthesis of **8a** to afford **9k** and **8k** (68%).

The products could not readily be separated by silica gel chromatography, so they were characterized as a mixture. The ratio of the isomer was determined by ^{1}H NMR Spectroscopy. The ^{1}H NMR spectrum of the product showed a 6:1 mixture of **9k** and a compound tentatively assigned as **8k** based on the methyl peak at δ 0.91 for **9k** and at δ 0.84 for **8k**.

For **9k** and **8k**, NMR Spectroscopy: **^{1}H NMR** (500 MHz; CDCl$_3$): δ = 0.84 (t, J = 7.0 Hz, 0.4H), 0.91 (t, J = 7.0 Hz, 2.7H), 1.25–1.38 (m, 4H), 1.51–1.57 (m, 2H), 2.16 (s, 2.5H), 2.19 (s, 0.4H), 2.46–2.53 (m, 1.8H), 6.84 (d, J = 7.5 Hz, 2H), 6.93 (s, 0.9H), 7.00-7.05 (m, 2.3H), 7.12 (t, J = 7.5 Hz, 2.6H), 7.21 (t, J = 7.5 Hz, 2H), 7.26–7.30 (m, 2.6H), 7.33 (d, J = 8.5 Hz, 0.3H), 7.37–7.41 (m, 2.6H); **^{13}C NMR** (125 MHz; CDCl$_3$): δ = 13.3, 14.1, 22.4, 22.5, 25.1, 25.2, 30.5, 30.9, 31.3, 31.5, 35.4, 35.8, 67.1, 67.2, 126.1, 126.3, 127.4, 127.5, 128.0, 128.1, 128.2, 128.3, 128.5, 128.7, 130.1, 130.4, 131.1, 131.2, 132.8, 133.1, 133.3, 133.4, 140.6, 142.2, 142.3, 143.2, 196.8, 197.5. Mass Spectrometry: HRMS (ESI-TOF) (m/z): Calcd for $C_{26}H_{30}NO_3S$, ([M + H]$^+$), 436.1941, found 436.1942.

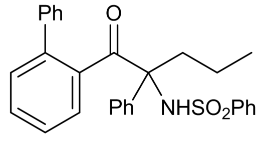

***N*-(1-([1,1'-biphenyl]-2-yl)-1-oxo-2-phenylpentan-2-yl)benzenesulfonamide 8l**

The title compound was prepared according to the synthesis of **8a** to afford **8l** (66%).

For **8l,** white solid, mp. 189–192 ^{o}C. NMR Spectroscopy: **^{1}H NMR** (400 MHz; CDCl$_3$): δ = 0.69 (t, J = 7.5 Hz, 3H), 1.31–1.49 (m, 2H), 2.47–2.51 (m, 2H), 7.13–7.16 (m, 2H), 7.19–7.22 (m, 3H), 7.34 (t, J = 7.5 Hz, 2H), 7.45–7.50 (m, 6H), 7.60 (t, J = 7.5 Hz, 1H), 7.85–7.89 (m, 4H); **^{13}C NMR** (100 MHz; CDCl$_3$): δ = 14.6, 22.6, 37.1, 71.4, 120.5, 126.7, 126.9, 127.0, 127.8, 127.9, 128.0, 128.7, 128.8, 132.5, 141.3, 141.5, 141.6, 145.6, 192.3. IR (in KBr): 3288, 3057, 1691, 1604, 1445, 1386, 1156, 747, 693, cm^{-1}. Mass Spectrometry: HRMS (ESI-TOF) (m/z): Calcd for $C_{29}H_{27}NNaO_3S$, ([M + Na]$^+$), 492.1604, found 492.1600.

General procedure for sythesis of α-alkyloxyl-α,α-diaryl imines (take 7a as an example).

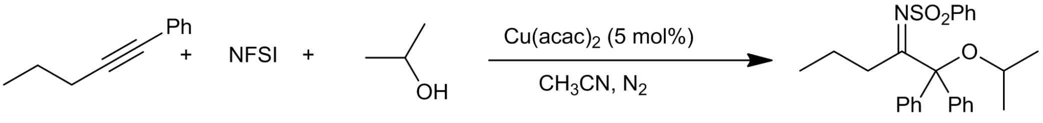

To a solution of the NFSI (1.0 mmol, 314.3 mg) in CH$_3$CN (2.0 mL) was added isopropanol (1.5

mmol, 114 μL), but-1-yn-1-ylbenzene (**6a**, 0.5 mmol, 80 μL), and Cu(acac)$_2$ (0.025 mmol, 6.5 mg) in screw-cap test tube under N_2 atmosphere. The test tube was then sealed off with a screw-cap and the reaction was stirred for the 48.0 h at ice base. After the reaction finished, the reaction mixture was cooled to room temperature and quenched by water. The mixture was extracted with CH_2Cl_2 (3 × 5.0 mL), the combined organic phases were dried over anhydrous Na_2SO_4 and the solvent was evaporated under vacuum. The residue was purified by column chromatography (petroleum ether/diethyl ether 25:1 (v/v)) to give the corresponding product **7a** (154.5 mg, 71%).

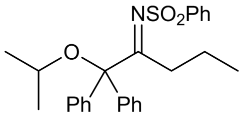

7a

***N*-(1-isopropoxy-1,1-diphenylpentan-2-ylidene)benzenesulfonamide 7a**

The title compound was prepared according to the synthesis of **7a** to afford **7a** (71%).

For **7a,** white solid, mp. 124–126 °C. NMR Spectroscopy: **^{1}H NMR** (500 MHz; $CDCl_3$): δ = 0.78 (s, 3H), 0.79 (s, 3H), 0.90 (t, J = 7.5 Hz, 3H), 1.41–1.50 (m, 2H), 2.95–2.98 (m, 2H), 3.50–3.61 (m, 1H), 7.26–7.29 (m, 6H), 7.36–7.38 (m, 4H), 7.44 (t, J = 7.5 Hz, 2H), 7.52–7.55 (m, 1H), 7.81–7.83 (m, 2H); **^{13}C NMR** (125 MHz; $CDCl_3$): δ = 14.9, 22.5, 23.7, 36.4, 67.9, 90.1, 126.8, 127.7, 127.9, 128.6, 129.9, 132.5 140.3, 141.1, 194.0. IR (in KBr): 3088, 3064, 1618, 1490, 1446, 1381, 1161, 1120, 1089, 770, 757, 703 cm^{-1}. Mass Spectrometry: HRMS (ESI-TOF) (m/z): Calcd for $C_{26}H_{29}NNaO_3S$, ([M + Na]$^+$), 492.1760, found 492.1777.

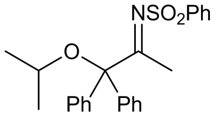

7b

***N*-(1-isopropoxy-1,1-diphenylpropan-2-ylidene)benzenesulfonamide 7b**

The reaction was carried out at 10 °C to afford **7b** (68%).

For **7b,** white solid, mp. 112–114 °C. NMR Spectroscopy: **^{1}H NMR** (500 MHz; $CDCl_3$): δ = 0.78 (s, 3H), 0.79 (s, 3H), 2.52 (s, 3H), 3.62–3.71 (m, 1H), 7.27–7.28 (m, 6H), 7.37–7.43 (m, 4H), 7.49 (t, J = 7.0 Hz, 2H), 7.57 (t, J = 7.5 Hz, 1H), 7.92–7.97 (m, 2H); **^{13}C NMR** (125 MHz; $CDCl_3$): δ = 21.4, 23.5, 68.2, 89.2, 126.8, 127.8, 127.9, 128.7, 129.4, 132.7, 140.3, 141.0, 190.0. IR (in KBr): 3089, 3062, 3035, 1626, 1491, 1447, 1367, 1163, 1126, 1088, 748, 749, 702 cm^{-1}. Mass Spectrometry: HRMS (ESI-TOF) (m/z): Calcd for $C_{24}H_{25}NNaO_3S$, ([M + Na]$^+$), 430,1447, found 430.1451.

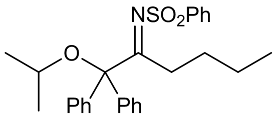

7c

***N*-(1-isopropoxy-1,1-diphenylhexan-2-ylidene)benzenesulfonamide 7c**

The title compound was prepared according to the synthesis of **7a** to afford **7c** (63%).

For **7c,** yellow liquid. NMR Spectroscopy: **^{1}H NMR** (500 MHz; $CDCl_3$): δ = 0.80 (s, 3H), 0.81 (s, 3H), 0.87 (t, J = 7.0 Hz, 3H), 1.31–1.38 (m, 2H), 1.43–1.49 (m, 2H), 3.00–3.03 (m, 2H),

3.57–3.62 (m, 1H), 7.28–7.30 (m, 6H), 7.39–7.40 (m, 4H), 7.46 (t, *J* = 7.5 Hz, 2H), 7.56 (t, *J* = 7.5 Hz, 1H), 7.83 (d, *J* = 7.5 Hz, 2H); **^{13}C NMR** (125 MHz; $CDCl_3$): *δ* = 13.4, 23.4, 23.7, 30.5, 34.2, 67.8, 90.2, 126.8, 127.7, 127.9, 128.6, 130.0, 132.5, 140.3, 141.2, 194.3. IR (in KBr): 3060, 3032, 1620, 1491, 1447, 1368, 1163, 1116, 1091, 771, 703 cm^{-1}. Mass Spectrometry: HRMS (ESI-TOF) (m/z): Calcd for $C_{27}H_{31}NNaO_3S$, ([M + Na]$^+$), 472.1917, found 492.472.1918.

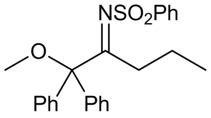

7d

***N*-(1-methoxy-1,1-diphenylpentan-2-ylidene)benzenesulfonamide 7d**

The reaction was carried out at 10 °C to afford **7d** (52%).

For **7d,** yellow liquid. NMR Spectroscopy: **^{1}H NMR** (500 MHz; $CDCl_3$): *δ* = 0.93 (t, *J* = 7.5 Hz, 3H), 1.54–1.60 (m, 2H), 2.95–3.00 (m, 5H), 7.27–7.32 (m, 10H), 7.42 (t, *J* = 7.5 Hz, 2H), 7.53(t, *J* = 7.5 Hz, 1H), 7.71–7.79 (m, 2H); **^{13}C NMR** (125 MHz; $CDCl_3$): *δ* = 15.0, 22.4, 35.9, 52.9, 90.8, 126.8, 127.8, 128.0, 128.6, 129.6, 132.5, 138.7, 141.0, 193.4. IR (in KBr): 3061, 3032, 1622, 1492, 1447, 1320, 1158, 1091, 768, 745, 702 cm^{-1}. Mass Spectrometry: HRMS (ESI-TOF) (m/z): Calcd for $C_{24}H_{25}NNaO_3S$, ([M + Na]$^+$), 430.1447, found 430.1456.

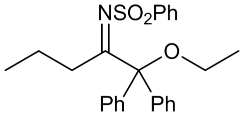

7e

***N*-(1-ethoxy-1,1-diphenylpentan-2-ylidene)benzenesulfonamide 7e**

The reaction was carried out at 10 °C to afford **7e** (62%).

For **7e,** white solid, mp. 108–110 °C. NMR Spectroscopy: **^{1}H NMR** (500 MHz; $CDCl_3$): *δ* = 0.95 (t, *J* = 7.5 Hz, 3H), 1.15 (t, *J* = 7.0 Hz, 3H), 1.58–1.65 (m, 2H), 2.97–3.02 (m, 4H), 7.27–7.31 (m, 10H), 7.41 (t, *J* = 7.5 Hz, 2H), 7.51 (t, *J* = 7.5 Hz, 1H), 7.77 (d, *J* = 7.5 Hz, 2H); **^{13}C NMR** (125 MHz; $CDCl_3$): *δ* = 15.0, 15.1, 22.3, 35.8, 60.5, 90.3, 126.8, 127.7, 127.9, 128.6, 129.4, 132.5, 139.3, 141.1, 193.7. IR (in KBr): 3061, 3032, 1621, 1492, 1447, 1320, 1158, 1118, 1090, 769, 746, 702 cm^{-1}. Mass Spectrometry: HRMS (ESI-TOF) (m/z): Calcd for $C_{25}H_{27}NNaO_3S$, ([M + Na]$^+$), 444.1604, found 444.1610.

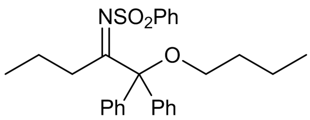

7f

***N*-(1-butoxy-1,1-diphenylpentan-2-ylidene)benzenesulfonamide 7f**

The reaction was carried out at 10 °C to afford **7f** (58%).

For **7f,** white solid, mp. 118–120 °C. NMR Spectroscopy: **^{1}H NMR** (500 MHz; $CDCl_3$): *δ* = 0.84 (t, *J* = 7.5 Hz, 3H), 0.96 (t, *J* = 7.5 Hz, 3H), 1.25–1.35 (m, 2H), 1.50–1.54 (m, 2H), 1.60–1.65 (m, 2H), 2.94–3.00 (m, 4H), 7.25–7.31 (m, 10H), 7.42 (t, *J* = 7.0 Hz, 2H), 7. 52 (t, *J* = 7.5 Hz, 1H), 7.77 (d, *J* = 7.5 Hz, 2H); ^{13}C NMR (125 MHz; $CDCl_3$): *δ* = 13.9, 15.0, 19.3, 22.2, 32.0, 35.9, 64.5,

90.2, 126.8, 127.8, 127.9, 128.6, 129.5, 132.5, 139.4, 141.2, 193.8. IR (in KBr): 3061, 3031, 1622, 1491, 1447, 1321, 1161, 1091, 767, 747, 703 cm^{-1}. Mass Spectrometry: HRMS (ESI-TOF) (m/z): Calcd for $C_{27}H_{31}NNaO_3S$, ([M + Na]$^+$), 472,1917, found 472,1936.

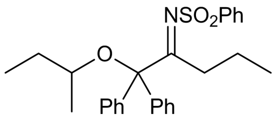

7g

N-(1-(*sec*-butoxy)-1,1-diphenylpentan-2-ylidene)benzenesulfonamide 7g

The title compound was prepared according to the synthesis of **7a** to afford **7g** (65%).

For **7g,** yellow liquid. NMR Spectroscopy: **^{1}H NMR** (500 MHz; $CDCl_3$): δ = 0.64 (t, J = 7.5 Hz, 3H), 0.75 (d, J = 6.0 Hz, 3H), 0.89 (t, J = 7.5 Hz, 3H),1.12–1.23 (m, 2H), 1.31–1.41 (m, 1H), 1.50–1.60 (m, 1H), 2.93–3.00 (m, 2H), 3.30–3.35 (m, 1H), 7.23–7.28 (m, 6H), 7.32–7.38 (m, 4H), 7.45 (t, J = 7.5 Hz, 2H), 7.54 (t, J = 7.5 Hz, 1H), 7.82 (d, J = 7.5 Hz, 2H); ^{13}C NMR (125 MHz; $CDCl_3$): δ = 9.1, 15.0, 20.0, 22.6, 29.8, 36.4, 72.2, 89.9, 126.9, 127.6, 127.7, 127.9, 128.0, 128.7, 129.8, 130.0, 132.5, 140.3, 140.4, 141.1, 194.0. IR (in KBr): 3062, 1620, 1489, 1448, 1319, 1160, 1089, 746, 699 cm^{-1}. Mass Spectrometry: HRMS (ESI-TOF) (m/z): Calcd for $C_{27}H_{31}NNaO_3S$, ([M + Na]$^+$), 472.1917, found 472.1939.

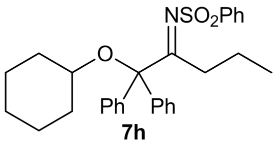

7h

N-(1-(cyclohexyloxy)-1,1-diphenylpentan-2-ylidene)benzenesulfonamide 7h

The reaction was carried out at 10 °C to afford **7h** (70%).

For **7h,** white solid, mp. 124–126 °C. NMR Spectroscopy: **^{1}H NMR** (500 MHz; $CDCl_3$): δ = 0.83–0.91 (m, 5H), 1.05–1.12 (m, 3H), 1.20–1.23 (m, 2H), 1.29–1.32 (m, 1H), 1.42–1.50 (m, 4H), 2.95–2.98 (m, 2H), 3.24–3.28 (m, 1H), 7.25–7.30 (m, 6H), 7.37–7.38 (m, 4H), 7.45 (t, J = 7.5 Hz, 2H), 7.54 (t, J = 7.5 Hz, 1H), 7.82 (d, J = 7.5 Hz, 2H); **^{13}C NMR** (125 MHz; $CDCl_3$): δ = 15.0, 22.5, 24.1, 25.5, 33.6, 36.6, 73.5, 90.1, 126.9, 127.7, 128.0, 128.7, 129.9, 132.5, 140.5, 141.2, 194.0. IR (in KBr): 3060, 1619, 1490, 1448, 1324, 1152, 1094, 765, 737, 702 cm^{-1}. Mass Spectrometry: HRMS (ESI-TOF) (m/z): Calcd for $C_{29}H_{33}NNaO_3S$, ([M + Na]$^+$), 498.2073, found 498.2042.

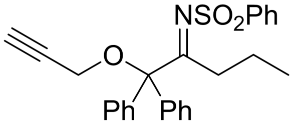

7i

N-(1,1-diphenyl-1-(prop-2-yn-1-yloxy)pentan-2-ylidene)benzenesulfonamide 7i

The reaction was carried out at 25 °C to afford **7i** (54%).

For **7i,** yellow liquid. NMR Spectroscopy: **^{1}H NMR** (500 MHz; $CDCl_3$): δ = 0.93 (t, J = 7.5 Hz, 3H), 1.50–1.57 (m, 2H), 2.37 (t, J = 7.5 Hz, 1H), 2.97–3.00 (m, 2H), 3.67 (d, J = 7.5 Hz, 2H), 7.29–7.32 (m, 10H), 7.44 (t, J = 7.5 Hz, 2H), 7.54 (t, J = 7.5 Hz, 1H), 7.81 (d, J = 7.5 Hz, 2H);

^{13}C NMR (125 MHz; $CDCl_3$): δ = 14.9, 22.4, 36.2, 53.7, 74.1, 79.6, 90.9, 126.9, 128.1, 128.4, 128.7, 129.5, 132.6, 138.2, 141.0, 192,4. IR (in KBr): 3290, 3060, 3028, 1710, 1599, 1492, 1447, 1358, 1172, 1032, 765, 700 cm^{-1}. Mass Spectrometry: HRMS (ESI-TOF) (m/z): Calcd for $C_{26}H_{25}NNaO_3S$, ([M + Na]$^+$), 454.1447, found 454.1458.

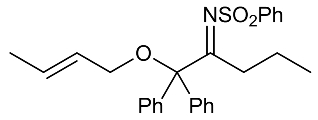

7j

***N*-(1-((*E*)-but-2-en-1-yloxy)-1,1-diphenylpentan-2-ylidene)benzenesulfonamide 7j**

The reaction was carried out at 25 °C to afford **7j** (46%).

For **7j,** yellow liquid. NMR Spectroscopy: **^{1}H NMR** (500 MHz; $CDCl_3$): δ = 0.94 (t, J = 7.5 Hz, 3H), 1.55–1.62 (m, 2H), 1.68–1.69 (m, 3H), 2.97–3.00 (m, 2H), 3.43 (d, J = 5.5 Hz, 2H), 5.45–5.49 (m, 1H), 5.61–5.66 (m, 1H), 7.27–7.32 (m, 10H), 7.43 (t, J = 7.5 Hz, 2H), 7.53 (t, J = 7.5 Hz, 1H), 7.76–7.79 (m, 2H); **^{13}C NMR** (125 MHz; $CDCl_3$): δ = 15.0, 17.9, 22.4, 36.0, 65.9, 90.4, 126.9, 127.1, 127.7, 127.8, 128.0, 128.6, 129.5, 132.5, 139.2, 141.1, 193.4. IR (in KBr): 3061, 3026, 1674, 1622, 1492, 1446, 1321, 1170, 1088, 755, 702 cm^{-1}. Mass Spectrometry: HRMS (ESI-TOF) (m/z): Calcd for $C_{27}H_{29}NNaO_3S$, ([M + Na]$^+$), 470.1760, found 470.1756.

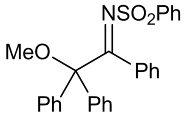

7k

***N*-(2-methoxy-1,2,2-triphenylethylidene)benzenesulfonamide 7k**

To a solution of NFSI (1.0 mmol, 315.3 mg) in CH_2Cl_2 (2.0 mL) was added methanol (1.5 mmol, 61 μL), 1,2-diphenylethyne (**6k**, 0.5 mmol, 89 mg) and CuCN (0.025 mmol, 2.2 mg) in a screw-cap test tube under N_2 atmosphere. The test tube was then sealed off with a screw-cap and the reaction was stirred at 90 ℃ for 48 h. After the reaction finished, the reaction mixture was cooled to room temperature and quenched by water. The mixture was extracted with CH_2Cl_2 (3 × 5.0 mL), the combined organic phases were dried over anhydrous Na_2SO_4 and the solvent was evaporated under vacuum. The residue was purified by column chromatography (petroleum ether/ethyl acetate 20:1 (v/v)) to give the corresponding product **7k** (145.6 mg, 68%).

For **7k,** white solid, mp. 184–186 °C. NMR Spectroscopy: **^{1}H NMR** (500 MHz; $CDCl_3$): δ = 2.79 (s, 3H), 7.10–7.15 (m, 2H), 7.25–7.35 (m, 10H), 7.36–7.38 (m, 1H), 7.40–7.49 (m, 5H), 7.60–7.64 (m, 2H). **^{13}C NMR** (125 MHz; $CDCl_3$): δ = 52.8, 90.2, 127.0, 127.1, 127.4, 127.8, 128.0, 128.6, 129.5, 129.8, 132.5, 135.1, 139.6, 140.5, 187.4. IR (in KBr): 3057, 1631, 1489, 1445, 1333, 1158, 1080, 745, 700 cm^{-1}. Mass Spectrometry: HRMS (ESI-TOF) (m/z): Calcd for $C_{27}H_{23}NNaO_3S$, ([M + Na]$^+$), 464.1291, found 464.1301.

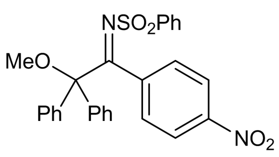

7l

***N*-(2-methoxy-1-(4-nitrophenyl)-2,2-diphenylethylidene)benzenesulfonamide 7l**

The title compound was prepared according to the synthesis of **7k** to afford **7l** (45%)

For **7l,** white solid, mp. 196–198 °C. NMR Spectroscopy: **^{1}H NMR** (500 MHz; $CDCl_3$): δ = 1.04 (t, J = 7.5 Hz, 3H), 1.77–1.84 (m, 2H), 3.06–3.10 (m, 2H), 7.27–7.36 (m, 10H), 7.42 (t, J = 7.5 Hz, 2H), 7.53 (t, J = 7.5 Hz, 1H), 7.71–7.73 (m, 2H). **^{13}C NMR** (125 MHz; $CDCl_3$): δ = 52.9, 90.9, 122.2, 127.1, 128.1, 128.4, 128.5, 128.8, 129.7, 133.1, 138.1, 139.9, 141.4, 148.1, 185.0. IR (in KBr): 3106, 3080, 3057, 1627, 1596, 1516, 1493, 1446, 1357, 1325, 1161, 1089 cm^{-1}. Mass Spectrometry: HRMS (ESI-TOF) (m/z): Calcd for $C_{27}H_{22}N_2NaO_5S$, ([M + Na]$^+$), 509.1142, found 509.1149.

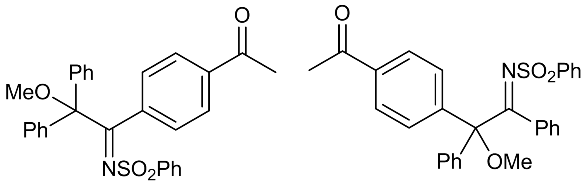

7m **7m'**

***N*-(1-(4-acetylphenyl)-2-methoxy-2,2-diphenylethylidene)benzenesulfonamide 7m**

***N*-(2-(4-acetylphenyl)-2-methoxy-1,2-diphenylethylidene)benzenesulfonamide 7m’**

The title compounds were prepared according to the synthesis of **7k** to afford **7m** and **7m’** (41%). The products could not readily be separated by silica gel chromatography, so they were characterized as a mixture. The ratio of the isomer was determined by ^{1}H NMR Spectroscopy. The ^{1}H NMR spectrum of the product showed a 3:2 mixture of **7m** and a compound tentatively assigned as **7m’** based on the methyl peak at δ 2.59 for **7m** and at δ 2.60 for **7m’**.

For **7m** and **7m’,** NMR Spectroscopy: **^{1}H NMR** (500 MHz; $CDCl_3$): δ = 2.59 (s, 1.8H), 2.60 (s, 1.2H), 2.79 (s, 3H), 7.14–7.16 (m, 2H), 7.28–7.33 (m, 6H), 7.35–7.40 (m, 5H), 7.47–7.52 (m, 1H), 7.53–7.56 (m, 1H), 7.47–7.52 (m, 1H), 7.63–7.66 (m, 2H), 7.87–7.89 (m, 2H). **^{13}C NMR** (125 MHz; $CDCl_3$): δ = 26.6, 26.7, 52.3, 52.9, 89.8, 90.4, 126.7, 126.9, 127.0, 127.2, 127.4, 127.6, 127.7, 127.9, 128.0, 128.2, 128.6, 128.7, 129.3, 129.5, 129.7, 130.1, 132.7, 134.7, 136.3, 137.3, 138.7, 139.1, 139.6, 140.1, 140.2, 145.2, 186.4, 197.4, 197.7. Mass Spectrometry: HRMS (ESI-TOF) (m/z): Calcd for $C_{29}H_{25}NNaO_3S$, ([M + Na]$^+$), 506.1397, found 506.1400.

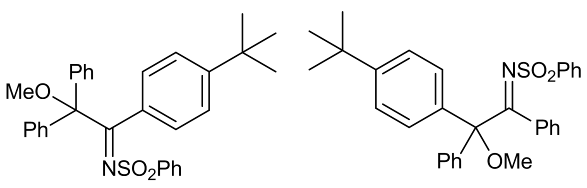

7n **7n'**

***N*-(1-(4-(*tert*-butyl)phenyl)-2-methoxy-2,2-diphenylethylidene)benzenesulfonamide 7n**

***N*-(2-(4-(*tert*-butyl)phenyl)-2-methoxy-1,2-diphenylethylidene)benzenesulfonamide 7n’**

The title compounds were prepared according to the synthesis of **7k** to afford **7n** and **7n’** (54%) The products could not readily be separated by silica gel chromatography, so they were characterized as a mixture. The ratio of the isomer was determined by ^{1}H NMR Spectroscopy. The ^{1}H NMR spectrum of the product showed a 1:2 mixture of **7n’** and a compound tentatively

assigned as **7n** based on the methyl peak at *δ* 2.80 for **7n** and at *δ* 2.81 for **7n'**.

For **7n** and **7n',** NMR Spectroscopy: **^{1}H NMR** (500 MHz; $CDCl_3$): *δ* = 1.34–1.36 (m, 9H), 2.80 (s, 1H), 2.81 (s, 2H), 7.14–7.17 (m, 2H), 7.29–7.36 (m, 10H), 7.38–7.45 (m, 1H), 7.46–7.49 (m, 4H), 7.60–7.65 (m, 2H). **^{13}C NMR** (125 MHz; $CDCl_3$): δ = 31.2, 31.3, 34.5, 34.8, 52.7, 52.8, 90.2, 124.1, 124.7, 127.4, 127.6, 127.8, 127.9, 128.0, 128.5, 128.6, 129.2, 129.3, 129.5, 129.6, 129.8, 132.3, 132.4, 132.5, 125.2, 126.3, 139.8, 140.1, 140.5, 140.6, 150.9, 153.3, 187.8, 187.9. Mass Spectrometry: HRMS (ESI-TOF) (m/z): Calcd for $C_{31}H_{31}NNaO_3S$, ($[M + Na]^+$), 520.1917, found 520.1920.

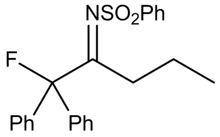

10

N-(1-fluoro-1,1-diphenylpentan-2-ylidene)benzenesulfonamide 10

The title compound was prepared according to the synthesis of **7j** to afford **10** (36%).

For **10,** white solid, mp. 126-128 °C. NMR Spectroscopy: **^{1}H NMR** (500 MHz; $CDCl_3$): *δ* = 2.85 (s, 3H), 7.16 (d, *J* = 8.6 Hz, 2H), 7.35–7.36 (m, 10H), 7.42 (t, *J* = 7.5 Hz, 2H), 7.55 (t, *J* = 7.5 Hz, 1H), 7.67 (d, *J* = 8.0 Hz, 2H), 8.15 (d, *J* = 8.5 Hz, 2H). **^{13}C NMR** (125 MHz; $CDCl_3$): δ = 14.9, 21.3 (d, *J* = 3 Hz), 36.4 (d, *J* = 3 Hz), 102.3 (d, *J* = 182 Hz), 126.7, 127.2 (d, *J* = 7 Hz), 128.1, 128.6, 128.7 (d, *J* = 2 Hz), 132.8, 139.0 (d, *J* = 23 Hz), 140.8, 190.4 (d, *J* = 33 Hz). ^{19}F NMR ($CDCl_3$, 470 MHz): *δ* = 145.9. Mass Spectrometry: HRMS (ESI-TOF) (m/z): Calcd for $C_{23}H_{22}FNNaO_2S$, ($[M + Na]^+$), 418.1247, found 418.1255.

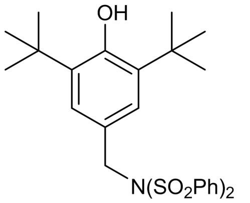

11

N-(3,5-di-*tert*-butyl-4-hydroxybenzyl)-N-(phenylsulfonyl)benzenesulfonamide 11

For **11,** dark yellow solid. NMR Spectroscopy: **^{1}H NMR** (500 MHz; $CDCl_3$): *δ* = 1.35 (s, 18H), 4.90 (s, 2H), 5.21 (s, 1H), 7.19 (s, 2H), 7.38–7.42 (m, 4H), 7.54 (t, *J* = 7.5 Hz, 2H), 7.77–7.79 (m, 4H); **^{13}C NMR** (125 MHz; $CDCl_3$): *δ* = 30.1, 34.2, 53.1, 124.8, 126.2, 127.9, 128.6, 133.3, 135.7, 140.4, 153.6. IR (in KBr): 3595, 2959, 2873, 1480, 1448, 1376, 1169, 881 cm^{-1}. Mass Spectrometry: HRMS (ESI-TOF) (m/z): Calcd for $C_{27}H_{33}NNaO_5S_2$, ($[M + Na]^+$), 538.1692, found 538.1696.

General procedure for semi-pinacol rearrangement.

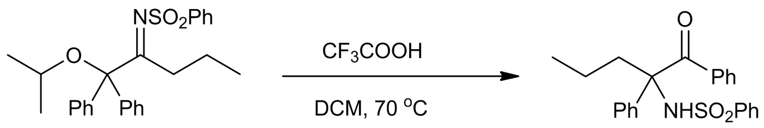

7a **8a**

To a solution of **7a** (0.3 mmol, 130.5 mg) in DCM (2.0 mL) was added TFA (0.3 mmol, 21 μL) in screw-cap a test tube under air. The test tube was then sealed off with a screw-cap and the reaction was stirred at 70 °C for 4.0 h. After the reaction finished, the reaction mixture was cooled to room temperature and quenched by water. The mixture was extracted with CH_2Cl_2 (3 × 5.0 mL), the combined organic phases were dried over anhydrous Na_2SO_4 and the solvent was evaporated under vacuum. The residue was purified by column chromatography (petroleum ether/ethyl acetate 10:1 (v/v)) to give the corresponding product **8a** (102.6 mg, 87%).

Supplementary References

(1) Thorand, S. & Krause, N. Improved procedures for the palladium-catalyzed coupling of terminal alkynes with aryl bromides (sonogashira coupling). *J. Org. Chem.* **63**, 8551 - 8553 (1998).

(2) Wang, F. *et al.* Enantioselective fluorination of 2-oxindoles by structure-micro-tuned *N*-fluorobenzenesulfonamides. *Eur. J. Org. Chem.* **2014**, 3607 - 3613 (2014).
